# Safety, tolerability, viral kinetics, and immune correlates of protection in healthy, seropositive UK adults inoculated with SARS-CoV-2: a single-centre, open-label, phase 1 controlled human infection study

**DOI:** 10.1016/S2666-5247(24)00025-9

**Published:** 2024-05-01

**Authors:** Susan Jackson, Julia L Marshall, Andrew Mawer, Raquel Lopez-Ramon, Stephanie A Harris, Iman Satti, Eileen Hughes, Hannah Preston-Jones, Ingrid Cabrera Puig, Stephanie Longet, Tom Tipton, Stephen Laidlaw, Rebecca Powell Doherty, Hazel Morrison, Robert Mitchell, Rachel Tanner, Alberta Ateere, Elena Stylianou, Meng-San Wu, Timothy P W Fredsgaard-Jones, Judith Breuer, Garth Rapeport, Vanessa M Ferreira, Fergus Gleeson, Andrew J Pollard, Miles Carroll, Andrew Catchpole, Christopher Chiu, Helen McShane, for the COV-CHIM01 study team, Maricel Alparaque, Liisa Anid, Rachel Benamore, Neha Bharti, Bhumika Patel, Adrian Burns, Charlotte Crowther, Trudi Johnstone, Jyolsna Jose, Aiseosa Nehiweze, Sibongile Nyamunda, Maria Orobiyi-Rieba, Bindu Parvelikudy, Dzikamayi Pswarayi, Binnie Elizabeth Samuel, Victoria Sodipo, Preethu Srijith, Helen Stone, Mary Ann Valmores, Alexandru Voaides, Gavindren Vuddamalay, Alessandro Sette, Eleanor Barnes, Susanna J Dunachie, Nicholas Byard, Oliver Conway, Michael Luciw, Abigail Platt, Jack Quaddy, Cheryl Turner, Cushla Cooper, Yama Mujadidi

**Affiliations:** Department of Paediatrics, University of Oxford, Oxford, UK; Department of Paediatrics, University of Oxford, Oxford, UK; The Peter Doherty Institute for Infection and Immunity, The University of Melbourne, Melbourne, VIC, Australia; Department of Paediatrics, University of Oxford, Oxford, UK; The Wellcome Centre for Human Genetics and Pandemic Sciences Institute, Nuffield Department of Medicine, University of Oxford, Oxford, UK; Department of Paediatrics, University of Oxford, Oxford, UK; Institute of Child Health, University College London, London, UK; National Heart and Lung Institute, Imperial College London, London, UK; Radcliffe Department of Medicine, University of Oxford, Oxford, UK; Oxford Radiology Research Unit, University of Oxford, Oxford, UK; Oxford Vaccine Group, Department of Paediatrics, University of Oxford, and the NIHR Oxford Biomedical Research Centre, Oxford, UK; Centre for Human Genetics and Pandemic Sciences Institute, Nuffield Department of Medicine, University of Oxford, Oxford, UK; hVIVO, London, UK; Department of Infectious Disease, Imperial College London, London, UK; Department of Paediatrics, University of Oxford, Oxford, UK; Oxford University Hospitals NHS trust Oxford UK; Centre for Infectious Disease and Vaccine Research, La Jolla Institute for Immunology, La Jolla, CA, USA; Peter Medawar Building for Pathogen Research, Nuffield Department of Clinical Medicine, University of Oxford, Oxford, UK; Jenner Institute, University of Oxford, Oxford, UK; Oxford Experimental Medicine Clinical Research Facility, Oxford University, Oxford, UK; Oxford Vaccine Group, Oxford University, Oxford, UK

## Abstract

**Background:**

A SARS-CoV-2 controlled human infection model (CHIM) has been successfully established in seronegative individuals using a dose of 1×10^1^ 50% tissue culture infectious dose (TCID_50_) pre-alpha SARS-CoV-2 virus. Given the increasing prevalence of seropositivity to SARS-CoV-2, a CHIM that could be used for vaccine development will need to induce infection in those with pre-existing immunity. Our aim was to find a dose of pre-alpha SARS-CoV-2 virus that induced infection in previously infected individuals.

**Methods:**

Healthy, UK volunteers aged 18–30 years, with proven (quantitative RT-PCR or lateral flow antigen test) previous SARS-CoV-2 infection (with or without vaccination) were inoculated intranasally in a stepwise dose escalation CHIM with either 1×10^1^, 1×10^2^, 1×10^3^, 1×10^4^, or 1×10^5^ TCID_50_ SARS-CoV-2/human/GBR/484861/2020, the same virus used in the seronegative CHIM. Post-inoculation, volunteers were quarantined in functionally negative pressure rooms (Oxford, UK) for 14 days and until 12-hourly combined oropharyngeal–nasal swabs were negative for viable virus by focus-forming assay. Outpatient follow-up continued for 12 months post-enrolment, with additional visits for those who developed community-acquired SARS-CoV-2 infection. The primary objective was to identify a safe, well tolerated dose that induced infection (defined as two consecutive SARS-CoV-2 positive PCRs starting 24 h after inoculation) in 50% of seropositive volunteers. This study is registered with ClinicalTrials.gov (NCT04864548); enrolment and follow-up to 12 months post-enrolment are complete.

**Findings:**

Recruitment commenced on May 6, 2021, with the last volunteer enrolled into the dose escalation cohort on Nov 24, 2022. 36 volunteers were enrolled, with four to eight volunteers inoculated in each dosing group from 1×10^1^ to 1×10^5^ TCID_50_ SARS-CoV-2. All volunteers have completed quarantine, with follow-up to 12 months complete. Despite dose escalation to 1×10^5^ TCID_50_, we were unable to induce sustained infection in any volunteers. Five (14%) of 36 volunteers were considered to have transient infection, based on the kinetic of their PCR-positive swabs. Transiently infected volunteers had significantly lower baseline mucosal and systemic SARS-CoV-2-specific antibody titres and significantly lower peripheral IFNγ responses against a CD8^+^ T-cell SARS-CoV-2 peptide pool than uninfected volunteers. 14 (39%) of 36 volunteers subsequently developed breakthrough infection with the omicron variant after discharge from quarantine. Most adverse events reported by volunteers in quarantine were mild, with fatigue (16 [44%]) and stuffy nose (16 [44%]) being the most common. There were no serious adverse events.

**Interpretation:**

Our study demonstrates potent protective immunity induced by homologous vaccination and homologous or heterologous previous SARS-CoV-2 infection. The community breakthrough infections seen with the omicron variant supports the use of newer variants to establish a model with sufficient rate of infection for use in vaccine and therapeutic development.

**Funding:**

Wellcome Trust and Department for Health and Social Care.

## Introduction

Controlled human infection models (CHIMs), also known as human challenge models, involve the deliberate inoculation of healthy volunteers with a pathogen in a carefully controlled clinical environment. Controlled exposure to an organism at a defined timepoint and dose allows an accurate study of incubation period and host immune response, and rapid testing of vaccines and therapeutics. Baseline samples can be used for the identification of immune correlates of protection.

Neutralising antibodies against SARS-CoV-2 have been correlated with protection against symptomatic SARS-CoV-2 infection.^1–3^ However, it is increasingly clear that mechanisms of protection are heterogeneous. The escape of variants from neutralising antibodies illustrates this concept, with protection against severe disease and death preserved^4^ despite a reduction in neutralising antibodies in both convalescent and vaccinated individuals.^5^ Preservation of T-cell responses across variants^6^ as well as non-neutralising Fc-effector function^7^ and memory B-cell breadth^8,9^ might contribute to the protection seen. Mucosal immunity has been less extensively studied than systemic responses, but mucosal IgA might play a role in early defence.^10,11^ Establishing a SARS-CoV-2 CHIM in volunteers with pre-existing immunity would allow investigation of the early host immune response, in the asymptomatic phase of infection, which is difficult to capture in field studies, as well as more precisely defined correlates of protection.

Killingley and colleagues have established a safe SARS-CoV-2 CHIM in seronegative, SARS-CoV-2-naive, healthy volunteers aged 18–29 years; 18 (53%) of 34 volunteers were infected via intranasal inoculation with a pre-alpha SARS-CoV-2 challenge virus at a dose of 1×10^1^ 50% tissue culture infectious dose (TCID_50_).^12^ This study enabled a detailed description of viral kinetics following primary infection.

With over 99% of the UK population and 59% of the global population reported to be seropositive to SARS-CoV-2, through vaccination or natural infection, a CHIM that can be used for therapeutics or vaccine development will need to reflect the dynamic range of immune protection from hybrid immunity (immunity arising from a combination of SARS-CoV-2 infection and vaccination) in the real-world population.^13,14^ Typically, CHIMs in individuals with pre-existing immunity require higher doses of the infectious challenge than needed in naive volunteers.^12^ Respiratory scyncitial virus and influenza CHIMs have safely used doses of 1×10^4^ to 1×10^7^ TCID_50_.^15,16^ We have conducted the first SARS-CoV-2 CHIM in seropositive individuals, with the aim of confirming safety and establishing an appropriate infectious dose to achieve infection in those with pre-existing immunity.

## Methods

### Study design and participants

This was a single-centre, phase 1, open-label study. Screening visits took place at either the Centre for Clinical Vaccinology and Tropical Medicine (CCVTM), Oxford, UK or the Oxford Experimental Medicine Clinical Research Facility, Oxford, UK. A detailed description of screening, eligibility, and recruitment procedures is available in the appendix (study protocol pp 107–117, 129–132). In brief, healthy individuals aged 18–30 years with documented evidence of previous SARS-CoV-2 infection (PCR or lateral flow antigen test) were comprehensively screened after written informed consent. Screening procedures included clinical history, physical examination, routine haematological and biochemical tests, serology for blood-borne viruses, chest x-ray, lung function testing, and cardiac MRI. Volunteers were excluded if they had any clinically significant medical conditions, risk factors for severe COVID-19 disease, or concerns regarding tolerance of study procedures including the quarantine isolation. Volunteers were enrolled at least 3 months after their primary SARS-CoV-2 infection and only if their primary infection had been well tolerated with no evidence of ongoing symptoms, hospitalisation, or end-organ damage. Careful consideration of the ethical and technical complexity of undertaking a SARS-CoV-2 challenge study during the pandemic is described elsewhere.^17^ The study was approved by the UK Health Research Authority Ad Hoc Specialist Ethics Committee (reference: 21/UK/0001) and conducted according to the principles of the Declaration of Helsinki and Good Clinical Practice.

### Dosing groups

Study design was closely aligned with the seronegative CHIM study to maximise cross-comparability.^12^ Different groups of four to eight volunteers were inoculated with increasing titres of virus in a stepwise, sequential dose escalation process. The starting dose of 1×10^1^ TCID_50_ was chosen to match the seronegative study, with subsequent dosing groups given either 1×10^2^, 1×10^3^, 1×10^4^, or 1×10^5^ TCID_50_ SARS-CoV-2 virus. The target infection rate was 50% (plus or minus 10%).

Initially, unvaccinated volunteers with a documented history of natural infection were enrolled. However, with the successful roll-out of the UK SARS-CoV-2 vaccine programme throughout 2021, recruitment of unvaccinated volunteers became increasingly unfeasible. Therefore, higher dose groups included volunteers with a history of previous infection with or without vaccination. Information on variant of primary infection was sought to determine the nature of the volunteers’ pre-existing immunity to the pre-alpha challenge strain. Available vaccines at time of enrolment were univalent and based on wild-type SARS-CoV-2, therefore offering homologous protection against the pre-alpha challenge variant. Previous infection might have been due to wild-type, alpha, delta, or omicron variants based on enrolment dates, and therefore immunity from previous infection might have been heterologous. Historical PCR results from Public Health England (eg, sequencing or S gene target failure data allowing proxy identification of alpha or omicron variants) were acquired where available or epidemiological data on the most prevalent variant at the time of primary infection are given (appendix p 20).

### Procedures

**Figure 1:**
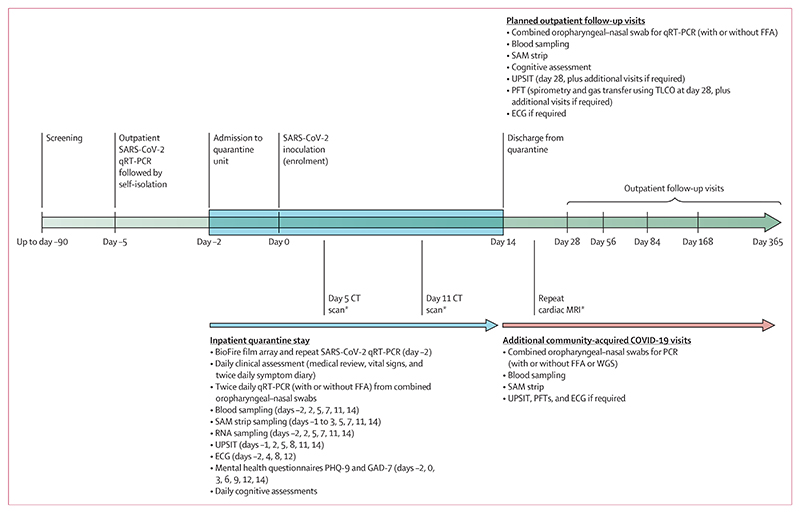
Study timeline and sample collection ECG=electrocardiogram. FFA=focus forming assay. GAD-7=7-item Generalized Anxiety Disorder scale. PHQ-9=9-item Patient Health Questionnaire. PFT=pulmonary function test. qRT-PCR=quantitative RT-PCR. SAM=synthetic absorptive matrix (for sampling of nasal lining fluid). TLCO=transfer capacity of the lung for carbon monoxide. UPSIT=University of Pennsylvania Smell Identification Test. WGS=whole-genome sequencing. *Selected participants as detailed in the appendix (study protocol pp 119–121).

Eligible volunteers were allocated quarantine dates based on their availability. 5 days before inoculation (day –5), volunteers attended the CCVTM, Oxford, UK, for an outpatient SARS-CoV-2 PCR and then self-isolated at home until admission to the quarantine unit at day –2. On admission, respiratory infection including SARS-CoV-2 was excluded on nasopharyngeal swab PCR (figure 1 and appendix p 2). Volunteers were inoculated with SARS-CoV-2/human/GBR/484861/2020 virus by nasal droplet on day 0 under strict infection control procedures (appendix p 2). Full details on challenge virus isolation, manufacture according to Good Manufacturing Practice (GMP), and release testing are described elsewhere.^12^

From day –2, volunteers were quarantined in functionally negative pressure single-occupancy en-suite rooms (Oxford, UK; appendix p 2) for a minimum of 14 days post-inoculation and until they had two consecutive swabs negative for viable virus using focus-forming assay (FFA).

Intravenou casirivimab–imdevimab monoclonal anti-body cocktail (Regeneron Pharmaceuticals, Tarrytown, NY, USA) and subsequently oral ritonavir-boosted nirmatrelvir (Pfizer, Kent, UK) were available as rescue therapy. Initially, the criteria for use of rescue therapy was two consecutive positive PCR swabs for SARS CoV-2, irrespective of symptoms. Following accumulation of satisfactory safety data from both our study and the seronegative study, rescue therapy was reserved for volunteers who demonstrated any warning features beyond mild upper respiratory tract symptoms and signs (appendix pp 2–3).

Volunteers underwent follow-up for 12 months post-enrolment, including unscheduled visits for community-acquired SARS-CoV-2 infection. These additional visits occured within 5 days of a positive test (PCR or lateral flow antigen test) for SARS-CoV-2, with re-attendance for a second post-COVID-19 visit 4–6 weeks later (figure 1 and appendix p 3).

### Virology

Combined oropharyngeal–nasal swabs (BioServUK, Rotherham, UK) were used for longitudinal detection of SARS-CoV-2 virus by quantitative RT-PCR (qRT-PCR) with N gene primers and probes as previously described (hVIVO, London, UK).^12^ PCR-positive swabs were further analysed by FFA (hVIVO, London, UK) to identify viable virus.^12^ Laboratory staff were masked to clinical status of participant and dosing group. Swabbing was 12-hourly during quarantine from the morning following challenge (day 1) until the morning of day 14 (discharge) and additionally at each outpatient follow-up visit. Henceforth, AM refers to the first swab of the day (morning) and PM refers to the second swab (evening).

Swabs from volunteers classified as transiently infected on the basis of their PCR results underwent whole-genome sequencing on frozen samples. Combined oropharyngeal–nasal swabs collected from volunteers at community-acquired COVID-19 visits were also analysed for whole-genome sequencing (appendix pp 4–5).

### Safety assessments

Following SARS-CoV-2 inoculation, volunteers were subject to comprehensive safety assessments both during their quarantine stay and at follow-up appointments (figure 1 and appendix [study protocol pp 117–124]). These included safety blood tests, symptom diary, pulmonary function testing, University of Pennsylvania Smell Identification Tests, cognitive assessments, electrocardigrams, mental health assessment (9-item Patient Health Questionnaire and 7-item Generalized Anxiety Disorder scale), cardiac MRI, and chest CT imaging.

### Immunological assessments

IFNγ ELISpots were performed on freshly isolated peripheral blood mononuclear cells (PBMC) taken from volunteers 2 days before SARS-CoV-2 inoculation and then at 2, 5, 7, 11, and 14 days and all follow-up visits post-inoculation, including community-acquired COVID-19 visits. SARS-CoV-2 peptide pools tested included the S1 and S2 subunit of the spike protein, membrane protein (M), nucleoprotein (NP), ORF3, ORF6, ORF7, ORF8, ORF10 (Mimotopes [UK], Wirral, UK), and predicted SARS-CoV-2 CD4^+^ and CD8^+^ T-cell epitopes (La Jolla Institute for Immunology, San Diego, CA, USA; appendix p 6). Nasal lining fluid (NLF) was collected via nasosorption using synthetic absorptive matrix (SAM) strips (Nasosorption FX-I-11, Hunt Developments, Midhurst, UK; appendix p 7). Baseline IgG, IgA, and IgM responses to SARS-CoV-2 antigens (spike [S], receptor binding domain [RBD], NP, and N-terminal domain [NTD]) were measured in NLF and serum using a multiplexed Meso Scale Discovery (MSD) immunoassay V-PLEX SARS-CoV-2 Panel 2 (Meso Scale Diagnostics, Rockville, MD, USA; appendix pp 7–8) and reported in arbitrary units (AU) per mL. Negative cutoff was calculated from pre-pandemic samples (median response +3 SDs; appendix p 19). Additionally, V-PLEX SARS-CoV-2 Panel 13 (Meso Scale Diagnostics, Rockville, MD, USA) was used to measure the ability of serum to inhibit angiotensin-converting enzyme 2 (ACE2) binding to SARS-CoV-2 spike variants (appendix pp 8–9). Microneutralisation assays of baseline serum samples were performed using SARS-CoV-2 variants and Vero E6 cells (appendix pp 9–10).

Laboratory staff were masked to infection status for all post-challenge quarantine samples.

### Outcomes

The primary endpoint was to identify a safe dose of pre-alpha SARS-CoV-2 challenge virus that induced infection in 50% (plus or minus 10%) of volunteers. Infection for our primary endpoint was prespecified in the protocol as laboratory detection of SARS-CoV-2 using quantitative RT-PCR (qRT-PCR) on two consecutive 12-hourly combined oropharyngeal–nasal swabs, from 24 h post-inoculation (ie, day 2 AM). SARS-CoV-2 PCR-positive results arising within the first 24 h were considered to represent residual inoculum and were classified as uninfected. Secondary objectives were to assess the viral dynamics of re-infection, and to explore the immune response to SARS-CoV-2 inoculation by correlating immune markers against infection outcome. Here we report our primary endpoint, the viral kinetics of re-infection from our dose escalation groups, initial baseline nasal and systemic immunology data in volunteers by infection status, and post-inoculation IFNγ ELISpot results (as these were undertaken on fresh samples). Since post-hoc analysis demonstrated a different pattern of PCR positivity to that seen in the seronegative study, we defined sustained infection as serial consecutive SARS-CoV-2 positive PCRs with associated serial consecutive viable virus on FFA and transient infection as any SARS-CoV-2 positive PCR outside of the residual inoculum period that did not meet the criteria for sustained infection.

### Statistical analysis

Statistical analysis was performed using GraphPad Prism version 9.4.1 (GraphPad Software, San Diego, CA, USA). This first SARS-CoV-2 CHIM in seropositive volunteers was an experimental medicine study and only our primary virological endpoint was prespecified in the protocol, with other exploratory analyses occurring post-hoc. Data were assessed for normal distribution using a Shapiro-Wilk test. Non-normally distributed quantitative measurements are summarised by the median (IQR). Volunteers were grouped based on infection status (transient infection or uninfected) regardless of dosing cohort. Two-group comparisons were undertaken using two-sided Mann-Whitney test. Antigen specific IFNγ ELISpot results post inoculation were assessed using a two-sided Wilcoxon matched pairs signed rank test to compare results from each post-inoculation timepoint (days 2, 5, 7, 10, 14, and 28) to baseline (day –2) for each dosing group. An area under the curve (AUC) analysis was performed to obtain a cumulative measurement of post-inoculation ELISpot results (days 2–28) for each volunteer, to allow comparison of volunteers grouped by infection status using two-sided Mann-Whitney test. For volunteers who acquired community COVID-19 during follow-up, a two-sided Wilcoxon matched pairs signed rank test was used to compare ELISpot responses during infection with pre-infection responses obtained at their preceding routine follow-up visit. A value of p<0·05 was considered significant for all tests.

This is an exploratory study and no formal sample size calculations were undertaken. However, dose groups of four to eight volunteers were considered sufficient to satisfy our primary endpoint with planned expansion of an additional ten to 20 volunteers in a dose confirmation cohort at the final dose. Before each dose escalation, the safety and virological data were reviewed by an independent Data Safety Monitoring Board (which also oversaw the seronegative study) and the Trial Steering Committee. A cutoff date of Dec 23, 2022, was used for interim analysis of safety data and community-acquired infections, with all volunteers having completed follow-up to at least day 28, and up to 12 months, as of this date. A full dataset analysis is planned after the last volunteer’s final visit of the dose confirmation cohort. The trial was registered with ClinicalTrials.gov (NCT04864548).

### Role of the funding source

The funders of the study had no role in study design, data collection, data analysis, data interpretation, or writing of the report.

## Results

**Figure 2:**
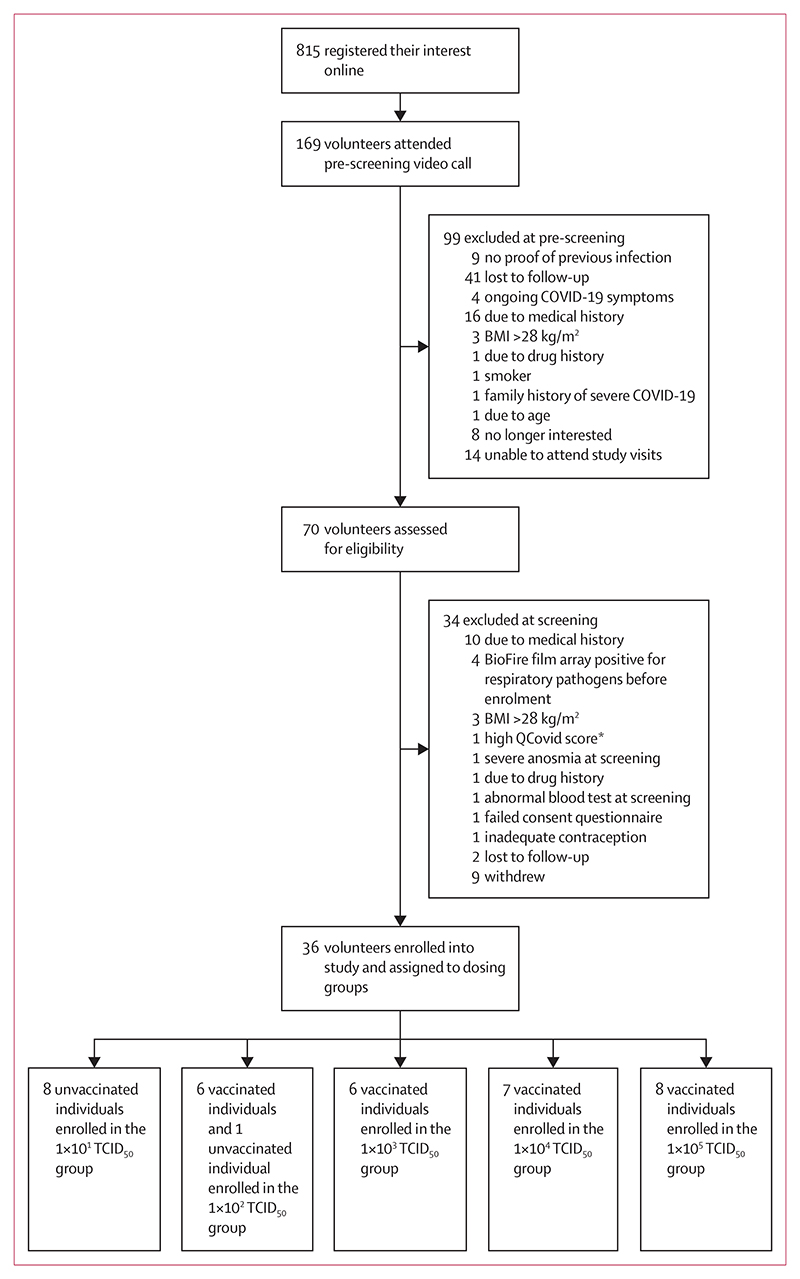
Trial profile TCID_50_=50% tissue culture infective dose. *QCovid score derived using QCovid tool (University of Oxford, Oxford, UK), a living risk prediction algorithm which gives individuals a calculated risk of hospitalisation and death from COVID-19 (appendix, study protocol pp 78–79).

70 volunteers were screened for eligibility and 36 volunteers were enrolled and inoculated with a dose of 1×10^1^ to 1×10^5^ TCID_50_ SARS-CoV-2 (figure 2). Recruitment commenced on May 6, 2021, and the last volunteer was enrolled into the dose escalation cohort on Nov 24, 2022. All enrolled volunteers have completed quarantine and 12 months post-inoculation follow-up.

24 (67%) of enrolled volunteers were male, and 29 (81%) self-identified as White (table 1).

Due to the roll-out of SARS-CoV-2 vaccines as the study progressed, the number of vaccines received at baseline increased with challenge dose. No volunteers in the first dose group, 1×10^1^ TCID_50_, had been vaccinated, whereas seven of eight volunteers in the final group, 1×10^5^ TCID_50_, had received three SARS-CoV-2 vaccinations at enrolment (table 1). Vaccinated individuals had significantly higher anti-spike antibody titres at baseline than did unvaccinated individuals (p<0·0001, Mann-Whitney test; table 1).

Two volunteers in the 1×10^1^ TCID_50_ group received casirivimab–imdevimab despite being clinically well, as they met the protocol-defined criteria of two consecutive PCR-positive swabs. No volunteers received rescue therapy on the grounds of clinical concern. None of the 36 enrolled volunteers developed sustained infection.

**Figure 3:**
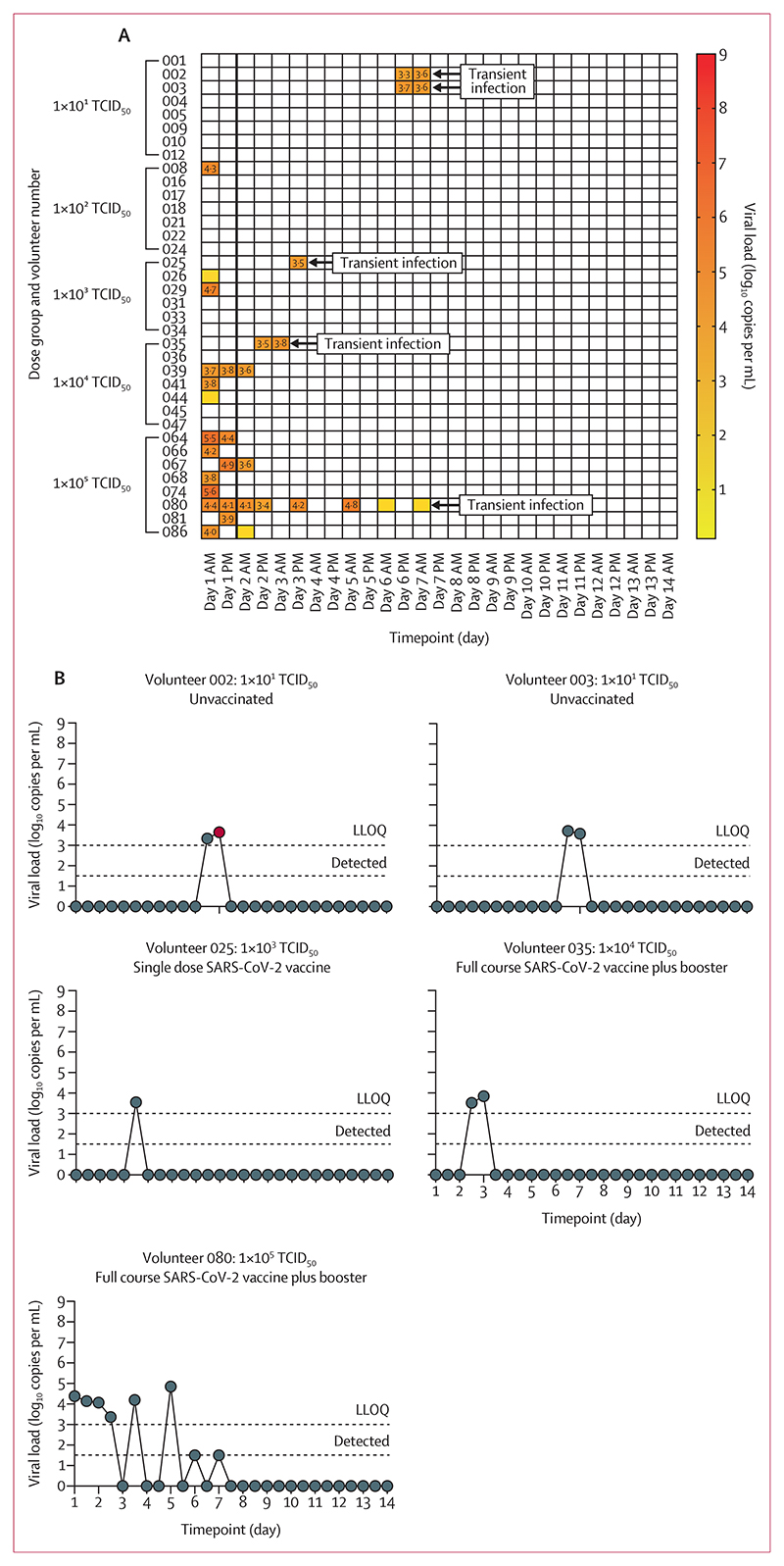
Positive PCRs during quarantine The LLOQ for quantitative RT-PCR (qRT-PCR) was 3 log_10_ copies per mL, with positive detections less than the LLOQ assigned a value of 1·5 log_10_ copies per mL and undetectable samples assigned a value of 0 log_10_ copies per mL. For the focus-forming assay (FFA), the LLOQ was 1·57 focus-forming units (FFU) per mL; viral detection less than the LLOQ was assigned 1 log_10_ FFU per mL; and undetectable samples were assigned 0 log_10_ FFU per mL. (A) All swabs taken during the quarantine period. Coloured squares denote detection of SARS-CoV-2 by qRT-PCR with viral load values presented; positive detections below the LLOQ are highlighted in yellow with no associated value for viral load. The five volunteers considered to demonstrate transient infection are labelled. All other SARS CoV-2-positive swabs were considered to represent residual inoculum, with initial viral detection occurring on day 1 (denoted by thick black line). (B) Viral kinetics of transiently infected volunteers by qRT-PCR. Dotted lines denote the LLOQ and positive detections below this value. Red dots denote corresponding FFA positivity of that sample, with only one volunteer (002) demonstrating positivity by FFA at a single timepoint (1·82 log_10_ FFU per mL). LLOQ=lower limit of quantification.

18 (50%) of 36 volunteers had SARS-CoV-2 PCR-positive swabs during quarantine (figure 3A, appendix p 11). 13 of 18 of these were considered to be residual inoculum only (SARS-CoV-2 PCR-positive at day 1). We detected residual inoculum more frequently in volunteers who received higher doses of SARS-CoV-2 (zero of eight at 1×10^1^ TCID_50_; eight of eight at 1×10^5^ TCID_50_, figure 3A). Three volunteers who were PCR-positive at day 1 remained positive at day 2 AM (all received 1×10^4^ or 1×10^5^ TCID_50_). As these swabs demonstrated a falling viral load, these volunteers were included in the residual inoculum only group.

Five (14%) of 36 volunteers demonstrated transient infection; short-lived detection of virus by PCR outside the immediate post-inoculation period and no evidence of persistent viral replication, with all but one PCR-positive swab testing negative for viable virus on FFA (volunteer 002, figure 3B).

Two of these volunteers (025 and 080) did not meet our primary endpoint prespecified definition of infection (positive for two consecutive 12-hourly timepoints starting 24 h post-inoculation). However, for the purpose of secondary endpoint immunological analysis, these volunteers were included in the transient infection cohort based on their viral kinetics, with clear episodes of SARS-CoV-2 PCR positivity distinct from the immediate post-inoculation phase. Volunteer 080 demonstrates likely residual inocula with a falling viral load from day 1 AM to day 2 PM, followed by a rebound rise in viral load and four subsequent non-consecutive positive swabs from day 3 PM to day 7 AM. Volunteer 025 had a single positive PCR at day 3 PM, however, whole genome sequencing performed on this swab demonstrated a synonymous mutation (C23536T; GenBank accession number OR046026) of the S-protein suggestive of active viral replication when compared against the genome from the challenge stock SARS-CoV-2/human/GBR/484861/2020 (Genbank accession number OM294022).

The five transiently infected individuals were from different dosing cohorts (figure 3B). There was no significant difference between time since vaccination, time since last known SARS-CoV-2 infection, or number of previous SARS-CoV-2 vaccine doses in those with transient infection compared with uninfected volunteers (data not shown).

Intranasal inoculation with doses of 1×10^1^ to 1×10^5^ TCID_50_ SARS-CoV-2 was well tolerated. There were no serious adverse events. Adverse events related to SARS-CoV-2 inoculation were mild or moderate, of short duration, and there were no symptoms or signs of post-COVID-19 condition (also known as long COVID) related to inoculation (table 2, appendix pp 16–18, 24). Symptoms did not consistently coincide with PCR positivity (appendix p 25). There were no clinically significant changes in any clinical assessments (figure 1) related to SARS-CoV-2 inoculation.

The vast majority of adverse events in the transiently infected volunteers were mild and had resolved by discharge (day 14), and all adverse events had resolved by day 18 (appendix p 25).

**Figure 4:**
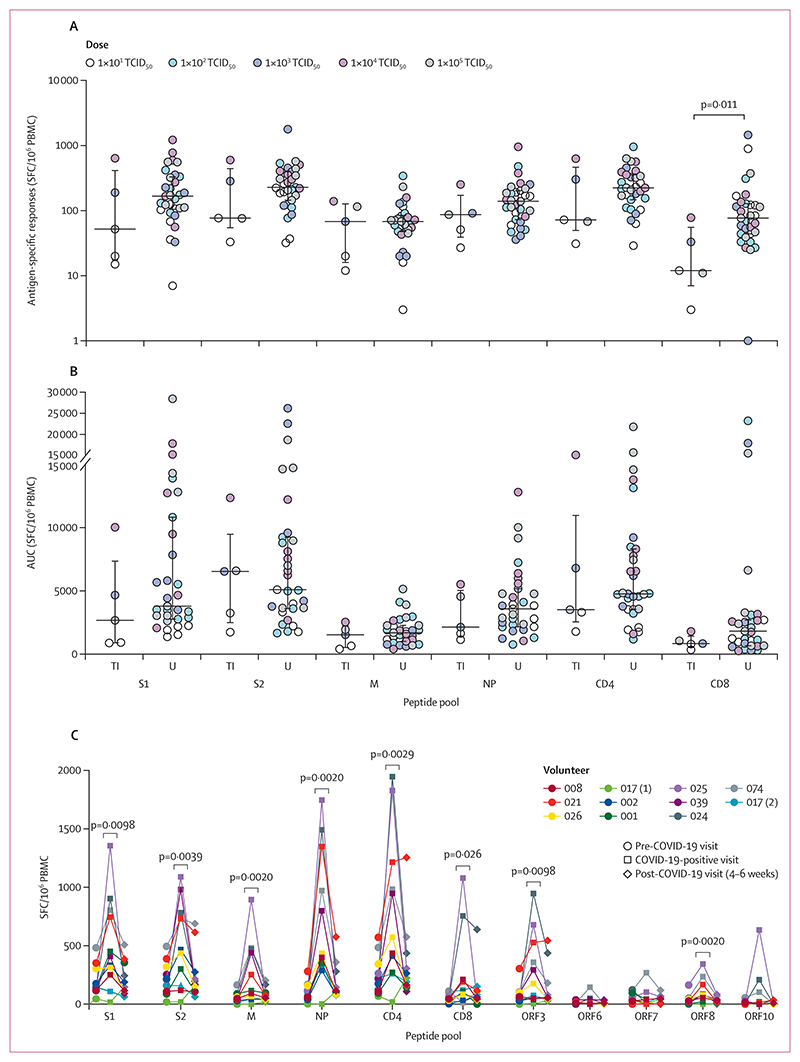
Cellular immunity before and after SARS-CoV-2 infection (A, B) Ex-vivo SARS CoV-2 peptide-specific PBMC IFNγ ELISpot responses by antigen, comparing transiently infected volunteers with uninfected volunteers. Dots represent individuals, colour coded for infection dose; lines show median with IQR. Background subtracted antigen-specific responses are presented as SFC per million PBMC. (A) Baseline ELISpot responses. (B) AUC analysis of ELISpot response over time between baseline and day 28. Dots represent AUC for individual volunteers. Groups were compared using two sided Mann-Whitney and no significant difference was seen. (C) Ex-vivo SARS CoV-2 peptide-specific ELISpot IFNγ responses by antigen performed on freshly isolated PBMC from volunteers with community-acquired COVID-19 infection during the post-quarantine follow-up period. Dots represent each volunteer; volunteer 017 had two community-acquired infections. Background subtracted antigen-specific responses are presented as SFC per million PBMC. AUC=area under the curve. CD4=CD4^+^ peptide pool. CD8=CD8^+^ peptide pool. M=membrane protein. NP=nucleocapsid protein. ORF=open reading frame. PBMC=peripheral blood mononuclear cells. S1=spike protein subunit 1. S2=spike protein subunit 2. SFC=spot-forming cells. TI=transiently infected. U=uninfected.

Volunteers who developed transient infection in quarantine had significantly lower baseline IFNγ ELISpot responses to the CD8^+^ T-cell epitope peptide pool compared with those who remained uninfected, with median responses of 12 (IQR 7–56) and 77 (44–128) spot-forming cells per million PBMC, respectively (p=0·011; figure 4A). There was a similar trend in baseline IFNγ ELISpot responses to the CD4^+^ T-cell epitope peptide pool and the spike protein peptide pools (S1 and S2) which did not reach statistical significance. AUC analysis was performed between baseline and day 28 for each volunteer; no significant difference in AUC was observed between the transiently infected and uninfected groups (figure 4B). No significant increase in IFNγ ELISpot responses was measured post-exposure in either structural or non-structural peptide pools (figure 4B; appendix pp 26–29).

**Figure 5:**
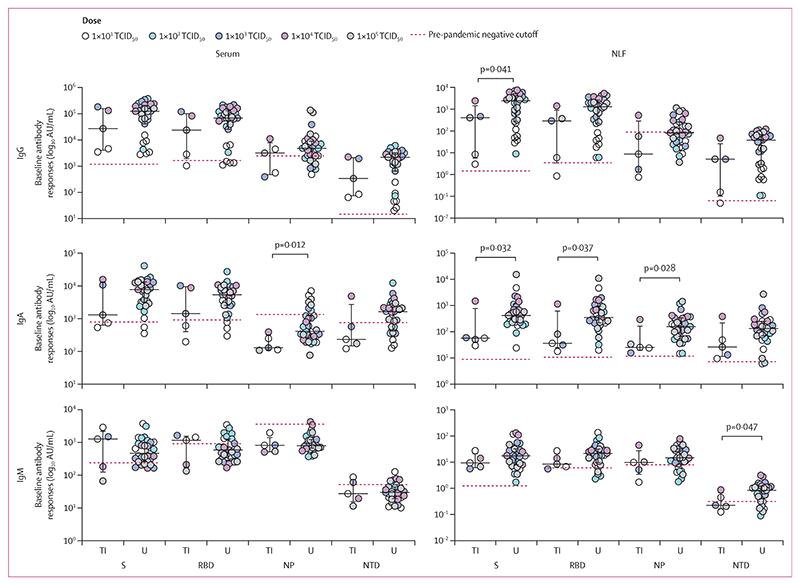
Baseline humoral responses against SARS-CoV-2 Baseline antibody responses (IgG, IgA, and IgM) against four SARS-CoV-2 structural proteins, from serum and NLF, comparing transiently infected volunteers with no transient infection. Dots represent individuals, colour coded for infection dose; black lines show median with IQR; red lines denote the pre-pandemic negative cutoff. Background subtracted antigen-specific responses are presented as SFC per million PBMC. AU=arbitrary units. NLF=nasal lining fluid. NP=nucleocapsid protein. NTD=N-terminal domain. RBD=receptor binding domain. S=spike protein. TI=transiently infected. U=uninfected.

Volunteers who developed transient infection had significantly lower baseline NLF antibodies against SARS-CoV-2 compared with uninfected volunteers (anti-spike IgG: median 411·9 AU/mL [IQR 5·7–1458·0] *vs* 2476·0 AU/mL [276·2–4183·0], p=0·041; anti-NTD IgM: 0·2 AU/mL [0·248–0·7] *vs* 0·8 AU/mL [0·4–1·2], p=0·047; anti-spike IgA: 57·1 AU/mL [40·2–776·1] *vs* 421·6 AU/mL [229·4–905·0], p=0·032; anti-RBD IgA: 36·4 AU/mL [24·8–614·2] *vs* 348·9 AU/mL [218·9–960·3], p=0·037; and anti-NP IgA: 25·5 AU/mL [19·8–164·0] *vs* 157·1 AU/mL [66·5–344·6], p=0·028) and significantly lower serum anti-NP IgA (128·9 AU/mL [IQR 111·3–321·0] *vs* 412·2AU/mL [266·2–1233·0], p=0·012; figure 5). A positive or negative baseline antibody level calculated using pre-pandemic samples (appendix p 7) did not predict transient infection in either serum or NLF.

No significant differences in baseline neutralising serum antibody responses between transiently infected and uninfected volunteers were observed using the ACE-2 inhibition assay or viral microneutralisation assay (appendix p 30). Baseline variant microneutralisation capacity did not consistently correspond with the likely historical infection variant exposure but most volunteers had also been vaccinated at baseline (appendix pp 20–23).

Up to Dec 23, 2022, we detected 14 (39%) volunteers with community-acquired SARS-CoV-2 infection (appendix pp 13–15). 11 of these volunteers were uninfected during quarantine. One of these volunteers (017) subsequently had two community-acquired infections. All infections occurred following the emergence of the omicron variant in the UK (from December, 2021) and all sequenced samples were confirmed as omicron.

IFNγ ELISpot responses at the community-acquired infection visit were significantly higher than those measured at the immediately preceding visit, in all peptide pools (S1: median 409 [IQR 251–804] *vs* 141 [119–304], p=0·0098; S2: 467 [159–783] *vs* 211 [89–320], p=0·0039; M: 116 [43–471] *vs* 48 [29–89], p=0·0020; NP: 436 [336–1348] *vs* 109 [51–159], p=0·0020; CD4: 573 [272–1215] *vs* 215 [108–344], p=0·0029; CD8: 108 [33–211] *vs* 44 [29–67], p=0·026; ORF3: 177 [48–528] *vs* 64 [28–99], p=0·0098; ORF8: 81 [20–167] *vs* 24 [4–39], p=0·0020), except ORF-6, ORF-7, and ORF-10 (figure 4C). The median time between the pre-COVID-19 and COVID-19-positive visit was 41 days (IQR 33–71).

## Discussion

Here we report the virological, clinical, and initial immunological results from the first SARS-CoV-2 human challenge study in individuals with pre-existing immunity to SARS-CoV-2. Despite escalating the inoculum dose to the maximum available (1×10^5^ TCID_50_), we were unable to induce sustained infection in seropositive individuals. Transient infection was seen in five of 36 volunteers. Despite this weak virological signal of infection, we detected statistically significant differences in baseline immune responses in transiently infected volunteers compared with uninfected volunteers. This difference was particularly apparent in mucosal binding antibody responses detected against the spike protein (whole, RBD, and NTD) and PBMC IFNγ responses to a CD8^+^ T-cell epitope peptide pool. 39% of our volunteers subsequently developed community breakthrough infections with the omicron variant.

A previous SARS-CoV-2 CHIM in seronegative individuals using the identical SARS-CoV-2 challenge strain resulted in sustained infection in 18 (53%) of 34 participants with an inoculum dose of 1×10^1^ TCID_50_.^12^ Although this current study was not designed to assess vaccine efficacy, this finding suggests that previous infection, together with vaccination with pre-alpha spike vaccines, offers strong homologous protection against a pre-alpha challenge strain up to a dose of 1×10^5^ TCID_50_. Although the natural infectious dose of virus is not known, dose escalation four-log higher than the infectious dose in the seronegative study illustrates the potency of this protection. This observation is consistent with field epidemiological data, which suggest that hybrid immunity offers the strongest resistance to re-infection.^18^ This alignment with field epidemiological data provides important validation of this seropositive CHIM, providing justification for the continued development of SARS-CoV-2 CHIMs as a tool to improve our understanding of host–pathogen immunobiology and SARS-CoV-2 viral kinetics.

The protective effects seen in our study were durable; in our high-dose group, median time from vaccination and primary infection to challenge was approximately 10 months, with these volunteers demonstrating robust resistance to sustained infection. The high-dose group volunteer who had the longest period of transient infection (volunteer 080) had received their last vaccine booster almost 2 years earlier and still demonstrated protection against sustained infection with a homologous strain. Field studies assessing both the effectiveness of vaccines to induce sterilising immunity and duration of induced protection are complicated by the emergence of genetically divergent variants. The omicron variant, in particular, has demonstrated its ability to evade neutralising antibodies and therefore sterilising immunity.^5^ Rapid mutation of the virus led to a reduction in the binding capacity of neutralising antibody to spike within the first year of the pandemic^19^ and by the time of the UK vaccine roll-out in early 2021, the pre-alpha variant was no longer dominant.^20^ By using a homologous challenge strain in our CHIM we are able to demonstrate durability of protection against infection. Our findings are consistent with data that suggest persistence of antibodies following both infection and vaccination for at least 12 months, with modelling suggesting persistence for years.^21,22^ We note it is possible that asymptomatic undetected infection might have boosted immune responses in our study volunteers, contributing to this protection.

Typically, field efficacy studies will use a single positive PCR as proof of transmissible infection. This might additionally lead to an underestimate of vaccine effectiveness as the detailed longitudinal viral kinetics from this experimental challenge model illustrate that despite some volunteers demonstrating transient episodes of PCR positivity, they did not represent sustained productive infection as evidenced by the live viral titres.

A major advantage of a CHIM is the ability to collect pre-challenge samples to identify correlates of protection. The lower baseline mucosal antibody responses against the spike protein seen in the transient infection group support a biologically plausible hypothesis that mucosal antibody responses provided sterilising immunity in this model. Antibodies against RBD and NTD have both shown neutralising capacity against the SARS-CoV-2 virus.^23,24^ Mucosal antibodies are induced following both natural infection and vaccination, although it is as yet unclear whether the source of mucosal antibody following a systemic vaccination is passive circulation transfer or tissue-resident B cells.^25–27^ Mucosal cellular immune responses were not investigated in this study; however, we saw significantly lower baseline PBMC IFNγ responses to a CD8^+^ T-cell epitope peptide pool in volunteers with transient infection, compared with uninfected participants. Challenge studies in macaques have previously highlighted the importance of antigen-specific CD8^+^ T cells in protection against re-infection.^28^

We saw no boosting of antigen-specific PBMC IFNγ responses post-inoculation, in contrast to the significant increases seen following community-acquired infection. This finding provides further support for the hypothesis that post-inoculation, mucosal immune responses are clearing the virus locally before activation of a systemic response. In field studies, serum antibody neutralisation has been shown to correlate with protection against infection and disease.^1–3^ We saw no difference in baseline serum micro-neutralisation capacity between transiently infected and uninfected volunteers, despite differences in total specific antibody titres. This observation might have been due to the small sample size, or it might be that serum antibody neutralisation is associated with protection against productive infection or disease, rather than transient infection, which might correlate with mucosal neutralisation capacity. Cervia and colleagues have identified SARS-CoV-2 specific mucosal IgA with neutralisation capacity in exposed health-care workers, who demonstrated no corresponding SARS-CoV-2 specific serum antibodies.^11^ In this study we were unable to perform microneutralisation on mucosal samples.

Interpretation of the virology data would have been aided by use of PCR methods that enable identification of viral replication, such as primers for negative-sense genomic and subgenomic RNA, the latter of which subsequently transcribes into positive-sense subgenomic RNA. This would have allowed us to better understand the significance of the unexpected viral kinetic seen in this exploratory study. It is possible that early positive samples that we have considered residual inoculum could represent replicating virus. Commercial positive-sense primers were chosen during study design to align with the seronegative study and with the expectation that we would see a similar viral kinetic in this study.

Undertaking this study during the changing landscape of the pandemic meant that as we progressed through the dosing groups, the number of pre-enrolment COVID-19 vaccines received increased. This might explain why transient infection was seen across several different dosing cohorts. Pragmatically, identifying a dose that induces infection in a study population representative of the current real-world population is important for the model’s generalisability. As transient infection was observed across different dosing groups, this variation in pre-enrolment vaccine status does not hamper our interpretation of correlates of protection as uninfected individuals within each dosing group (enrolled contemporaneously in time) serve as a control for the transiently infected volunteers within that group. The small number of transient infections seen is, however, an important limitation, and although the correlates of protection identified are biologically plausible, further investigation in a model that is able to produce a higher proportion of infected volunteers is necessary. Additionally, as the first seropositive SARS-CoV-2 CHIM, an appropriately cautious approach to recruitment restricted our study population to those at lowest risk of severe disease, which does limit the generalisability of the model.

Ongoing work includes expansion of the highest dose group to increase our sample size both providing further confidence in the infection rate and kinetic seen in this model and testing the significance of the putative correlates of protection. Further interrogation of both baseline correlates of protection, such as the contribution of immune memory to the durability of protection seen, as well as detailed characterisation of the post-exposure immune response is planned. A comprehensive analysis of the post-inoculation samples from this study is underway and will yield further important information. The ability to study early post-exposure mucosal responses in a CHIM yields information not easily obtained from other study designs.

A CHIM that could be used to test interventions will need to induce a stronger virological signal of infection than was seen in this study in those with pre-existing immunity. Doses of up to 1×10^7^ TCID_50_ have been required in influenza CHIM studies where subjects have pre-existing immunity.^15,16^ In this model, it was not feasible to increase the challenge dose beyond the neat Master Seed Virus concentration of 1×10^5^ TCID_50_, as this would have necessitated larger volumes of inoculum, which would have been a potential confounder when considering infection rate. Although many variables such as dose and duration of exposure might confound the community infections seen in this study, the substantial number of breakthrough infections with the omicron variant in volunteers who remained uninfected after inoculation with the pre-alpha strain justifies evaluating other variants in this model. Use of prevalent variants would be more relevant for vaccine and therapeutic evaluation and work is underway to use variants known to demonstrate immune escape, such as delta (ISRCTN94747181) or omicron.^5,29^ Comparison of results from a CHIM undertaken with later evolutionary variants against this study could provide valuable insights into the mechanisms underpinning this immune escape. However, the inclusion of actively circulating variants of concern in a CHIM are hampered by both the time taken to manufacture viral challenge stock under GMP standards and collection of sufficient field safety data on a new variant of concern to justify its ethical use in a CHIM. With the ongoing high global incidence of COVID-19 resulting in continued variant evolution, keeping pace with current variants of concern in a CHIM might be difficult. Commencing GMP manufacture at risk, as soon as a variant is identified and while clinical and epidemiological data are being accumulated, would facilitate the development of a CHIM with a relevant circulating strain.

Immune correlates of protection are likely to be conserved, at least in part, across variants of concern. Therefore, assessing the safety and feasibility of a homologous SARS-CoV-2 CHIM, for which the most field safety data were available, was an important initial step. This study has provided the framework, processes, and confidence that SARS-CoV-2 challenge can be undertaken safely in seropositive individuals. It provides assurance that future studies can consider careful widening of eligibility criteria, thus enabling the findings to be more applicable to the real-world population. Demonstrating the feasibility, safety, and tolerability of CHIMs undertaken in a pandemic setting to both the public and scientific community will set a precedent for use in future pandemics. Use of CHIMs has recently been acknowledged as one means of accelerating pandemic vaccine development in CEPI’s 100-day mission to respond to future threats.^30^ Developing SARS-CoV-2 CHIMs using a selection of variants from different evolutionary lineages would facilitate evaluation of vaccines and therapeutics, and the definition of immune signatures of protection.

## Supplementary Material

Suppl

## Figures and Tables

**Table 1:** Participant baseline physical and demographic characteristics and vaccination and virology history, by study group

	Total (n=36)	1×10^1^ TCID_50_ (n=8)	1×10^2^ TCID_50_ (n=7)	1×10^3^ TCID_50_ (n=6)	1×10^4^ TCID_5__0_ (n=7)	1×10^5^ TCID_50_ (n=8)
Enrolment dates	May 27, 2021–Nov 24, 2022	May 27–July 22, 2021	Aug 19–Nov 4, 2021	Nov 4, 2021–Jan 6, 2022	March 10–March 31, 2022	Aug 11–Nov 24, 2022
Characteristics						
Age, years						
Median (IQR)	25 (21–27)	27 (25–28)	25 (23–27)	22 (20–24)	21 (21–26)	26 (25–27)
Range	19–30	19–30	22–27	20–27	20–29	20–28
Biological sex						
Male	24 (67%)	7 (88%)	5 (71%)	4 (67%)	5 (71%)	3 (38%)
Female	12 (33%)	1 (13%)	2 (29%)	2 (33%)	2 (29%)	5 (63%)
Ethnicity						
White	29 (81%)	7 (88%)	6 (86%)	4 (67%)	5 (71%)	7 (88%)
Asian	1 (3%)	1 (13%)	0	0	0	0
Black	1 (3%)	0	1 (14%)	0	0	0
Mixed ethnicity	5 (14%)	0	0	2 (33%)	2 (29%)	1 (13%)
BMI, kg/m^2^						
Median (IQR)	23⋅4 (22⋅4–24⋅7)	24⋅1 (23⋅0–24⋅7)	23⋅7 (23⋅5–25⋅0)	22⋅9 (21⋅5–23⋅0)	22⋅6 (21⋅9–25⋅3)	23⋅3 (20⋅1–24⋅4)
Range	19⋅1–27⋅5	21⋅3–25⋅4	23⋅2–27⋅5	19⋅1–23⋅9	20⋅8–25⋅9	19⋅5–25⋅7
Vaccine status at enrolment						
Unvaccinated	9 (25%)	8 (100%)	1 (14%)	0	0	0
One dose	3 (8%)	0	1 (14%)	2 (33%)	0	0
Two doses (full course)*	13 (36%)	0	5 (71%)	3 (50%)	4 (57%)	1 (13%)
Booster (univalent)	11 (31%)	0	0	1 (17%)	3 (43%)	7 (88%)
Time elapsed since last vaccine dose at enrolment, days						
Median (IQR)	109 (75–235)	NA	64 (47–87)	92 (65–138)	88 (80–213)	296 (248–324)
Range	25–613	NA	37–94	39–161	25–240	178–613
Time elapsed since primary infection at enrolment, days†						
Median (IQR)	262 (216–396)	218 (202–232)	317 (250–341)	300 (197–421)	515 (331–520)	299 (219–389)
Range	100–667	144–258	145–371	120–465	111–539	100–667
Baseline anti-spike antibody titre, AU/mL‡						
Median (IQR)	7472⋅9 (2104⋅7–15 227⋅9)	159⋅8 (118⋅7–314⋅9)	14 653⋅7 (9037⋅9–17 212⋅0)	14 840⋅5 (9665⋅7–27 713⋅0)	8853⋅5 (7665⋅5–34 258⋅9)	5575⋅7 (4598⋅7–8044⋅6)
Range	78⋅4–40000⋅0	78⋅4–668⋅2	242⋅4–36 604⋅2	3276⋅9–40 000⋅0	4418⋅7–40 000⋅0	2583⋅5–8189⋅2
Baseline anti-nucleocapsid antibody titre‡						
Positive	13 (36%)	0	2 (29%)	2 (33%)	4 (57%)	5 (63%)
Negative	12 (33%)	3 (38%)	1 (14%)	4 (67%)	3 (43%)	1 (13%)
Equivocal	11 (31%)	5 (63%)	4 (57%)	0	0	2 (25%)
Primary infection: known variant type or assumed variant based on S gene target failure data and variant prevalence§						
Unknown	26 (72%)	5 (63%)	4 (57%)	3 (50%)	6 (86%)	8 (100%)
Victoria	4 (11%)	1 (13%)	2 (29%)	1 (17%)	0	0
Alpha	4 (11%)	2 (25%)	1 (14%)	0	1 (14%)	0
Delta	2 (6%)	0	0	2 (33%)	0	0

Data are n (%) unless otherwise stated. In some categories, percentages do not sum to 100% due to rounding. AU=arbitrary units. TCID_50_=50% tissue culture infective dose.

* Two doses is equivalent to full course of primary vaccination against COVID-19 (ie, also includes single Janssen vaccine). †Where volunteers have had more than one confirmed COVID-19 infection preceding enrolment, time since most recent infection has been used.

‡ Baseline serum status performed at screening visit, up to 90 days before enrolment. Anti-spike and anti-nucleocapsid antibody titres were measured using Abbott ARCHITECT assay (Abbott Laboratories, Green Oaks, IL, USA). Upper limit of anti-spike assay is 40 000 AU/mL. Qualitative result for anti-nucleocapsid with equivocal representing low-level anti-N antibody status or non-specific reactivity in the assay (0·4–1·46 AU/mL). §Public Health England confirmed primary infection of enrolled volunteers. Data obtained included sequencing of variant or S gene target failure data where available. Where S gene target failure data were available, the underlying variant was assumed based on the known prevalence of different variants in the UK at the time of primary infection.

**Table 2:** Adverse events in quarantine

	Number of adverse events in all volunteers (n=36)	Number of adverse events by infection status			Number of adverse events by dose group				
		Transiently infected (n=5)	Uninfected (n=31)		1×10^1^ TCID_50_ (n=8)	1×10^2^ TCID_50_ (n=7)	1×10^3^ TCID_50_ (n=6)	1×10^4^ TCID_50_ (n=7)	1×10^5^ TCID_50_ (n=8)
Fatigue									
Mild	16 (44%)	2 (40%)	14 (45%)		6 (75%)	3 (43%)	3 (50%)	2 (29%)	2 (25%)
Moderate	1 (3%)	1 (20%)	0		0	0	0	1 (14%)	0
Stuffy nose									
Mild	16 (44%)	3 (60%)	13 (42%)		3 (38%)	2 (29%)	3 (50%)	5 (71%)	3 (38%)
Headache									
Mild	10 (28%)	1 (20%)	9 (29%)		4 (5%)	1 (14%)	1 (17%)	4 (57%)	0
Moderate	2 (6%)	0	2 (6%)		0	2 (29%)	0	0	0
Sore throat									
Mild	9 (25%)	2 (40%)	7 (23%)		1 (13%)	3 (43%)	2 (33%)	3 (43%)	0
Moderate	1 (3%)	0	1 (3%)		0	0	1 (17%)	0	0
Runny nose									
Mild	8 (22%)	1 (20%)	7 (23%)		1 (13%)	1 (14%)	1 (17%)	2 (29%)	3 (38%)
Sneezing									
Mild	8 (22%)	3 (60%)	5 (16%)		2 (25%)	1 (14%)	0	3 (43%)	2 (25%)
Muscle pains									
Mild	7 (19%)	0	7 (23%)		1 (13%)	1 (14%)	2 (33%)	1 (14%)	2 (25%)
Sore eyes									
Mild	5 (14%)	1 (20%)	4 (13%)		1 (13%)	2 (29%)	2 (33%)	0	0
Moderate	1 (3%)	0	1 (3%)		0	0	1 (17%)	0	0
Tickly throat									
Mild	5 (14%)	1 (20%)	4 (13%)		0	0	2 (33%)	2 (29%)	1 (13%)
Cough									
Mild	4 (11%)	0	4 (13%)		0	1 (14%)	1 (17%)	2 (29%)	0
Change in sense of smell or taste									
Mild	4 (11%)	0	4 (13%)		1 (13%)	0	2 (33%)	1 (14%)	0
Toothache									
Severe	1 (3%)*	0	1 (3%)*		0	0	0	1 (14%)*	0

Symptom scores were collected using self-reported symptom diaries twice daily from all volunteers and graded as mild, moderate, or severe. Number of adverse events during the quarantine period (within 14 days of SARS-CoV-2 inoculation) by the maximum grade reported, showing all adverse events reported by ≥ 10% of volunteers and all severe adverse events regardless of frequency. *Unsolicited adverse event deemed unrelated to SARS-CoV-2 inoculation: dental infection in an uninfected participant successfully treated with oral antibiotics in quarantine.

## Data Availability

De-identified participant data will be made available upon requests directed to the chief investigator. Proposals will be reviewed by the sponsor, study team, chief investigator, and collaborators on the basis of scientific merit and a response can be expected within 14 days. Requests should be made to the corresponding author (helen.mcshane@ndm.ox.ac.uk). After approval of a proposal, data can be shared through a secure online platform after signing a data access agreement.

## Research in context

### Evidence before this study

To identify other SARS-CoV-2 controlled human infection models, we searched PubMed with the following search terms: (controlled human infection model) OR (human challenge model) AND (SARS-CoV-2) OR (COVID-19) AND (Clinical trial) on May 11, 2023, from database inception up to this date. There were no language restrictions.

Our search identified one published study, a SARS-CoV-2 human infection model undertaken in healthy, seronegative individuals aged 18–29 years in the UK using intranasal inoculation with a pre-alpha SARS-CoV-2 virus. Using an inoculation dose of 1×10^1^ 50% tissue culture infectious dose (TCID_50_), the study showed a 53% infection rate (18 of 34 volunteers). Viral kinetics post-inoculation demonstrated shedding of SARS-CoV-2 within 2 days of exposure, peaking at 5 days. Infectious virus persisted for an average of 10 (maximum 12) days. Challenge was well tolerated with no evidence of lower respiratory tract involvement.

### Added value of this study

With increasing global seroprevalence to SARS-CoV-2 via natural infection and vaccination, identifying seronegative volunteers for controlled human infection models (CHIMs) is no longer feasible. A model in seropositive volunteers will be necessary for a SARS-CoV-2 CHIM to remain a viable tool in the development of vaccines and therapeutics. To our knowledge, our study is the first SARS-CoV-2 CHIM in seropositive volunteers, showing that challenge up to a dose of 1×10^5^ TCID_50_ was well tolerated but did not reproduce the same sustained infection kinetic seen in the seronegative study. Our inability to reproduce sustained infection is in keeping with emergent field epidemiological data on re-infection in seropositive individuals, thus demonstrating the biological validity of our model.

Our ability to longitudinally assess volunteers post-exposure has identified an unexpected transient infection kinetic in seropositive individuals, raising the question of whether asymptomatic or mild breakthrough infections in the field could follow a similar kinetic.

The identification and comprehensive characterisation of immune correlates of protection is possible in a SARS-CoV-2 CHIM in seropositive volunteers. We compared our transiently infected cohort to those who remained uninfected and identified credible correlates of protection against susceptibility to re-infection including SARS-CoV-2 specific mucosal and serum antibodies and systemic CD8^+^ T cells. Such a model allows the detailed interrogation of mucosal immunity in particular, which is currently poorly defined in SARS-CoV-2 immunobiology.

### Implications of all the available evidence

Both SARS-CoV-2 models have provided a framework for the safe and ethical conduct of SARS-CoV-2 CHIMs and demonstrated the broad utility of CHIMs, including providing supporting evidence for public health measures and understanding correlates of protection. The identification of this unexpected infection kinetic highlights that serial testing, rather than testing at a single timepoint, might be useful at characterising breakthrough infection in the community. Additionally, our findings suggest that baseline mucosal immunity in particular could be an important factor in aborting infection at the point of entry, thus preventing onward viral transmission. Strategies that boost mucosal immunity therefore warrant further investigation.

More work is needed on optimising a seropositive SARS-CoV-2 CHIM to produce sustained and reproducible infection if CHIMs are to be used for vaccine and therapeutic assessment. The community breakthrough infections secondary to omicron seen in this model suggest that use of genetically divergent variants as a challenge agent should be evaluated.
