# Supplementary material for: Safety, tolerability, viral kinetics, and immune correlates of protection in healthy, seropositive UK adults inoculated with SARS-CoV-2: a single-centre, open-label, phase 1 controlled human infection study
Source: Lancet Microbe. Author manuscript; Available in PMC 2024 Sep 26. (PMC7616636; doi:10.1016/S2666-5247(24)00025-9)
Supplement: Suppl [file EMS198676-supplement-Suppl.pdf]

THE LANCET
Microbe

Supplementary appendix

This appendix formed part of the original submission and has been peer reviewed. We post it as supplied by the authors.

Supplement to: Jackson S, Marshall JL, Mawer A, et al. Safety, tolerability, viral kinetics, and immune correlates of protection in healthy, seropositive UK adults inoculated with SARS-CoV-2: a single-centre, open-label, phase 1 controlled human infection study. *Lancet Microbe* 2024. https://doi.org/10.1016/S2666-5247(24)00025-9

Supplementary Appendix

Contents

Supplementary Appendix 1

Methods 2

Procedures 2

Virology 4

Challenge Virus 4

Assays methods 5

Whole Genome Sequencing of volunteers with community acquired COVID-19 5

Immunological assessments 6

IFN-γ Enzyme-Linked Immunospot (ELISpot) 6

Nasal lining fluid collection (NLF) for mucosal immunology 7

Meso Scale Discovery (MSD®) IgG, IgA and IgM binding Assays 7

Meso Scale Discovery (MSD®) ACE2 inhibition surrogate neutralisation assay 8

Microneutralisation assay 9

Supplementary Tables 11

Supplementary Table 1: All positive quarantine PCR results 11

Supplementary Table 2: Summary of community acquired COVID infections 13

Supplementary Table 3: All laboratory AEs deemed possibly, probably or definitely related to SARS-CoV-2 inoculation 16

Supplementary Table 4: MedDRA coded AEs following discharge from quarantine 17

Supplementary Table 5: Positive cut off values for A. serum and B. nasal lining fluid (NLF) calculated from pre-pandemic samples 19

Supplementary Table 6: Volunteer vaccine status and primary infection variant at screening compared with microneutralisation assay (MNA) using baseline serum 20

Supplementary Figure 1: Solicited AEs in quarantine 24

Supplementary Figure 2: Solicited symptoms in transiently infected volunteers 25

Supplementary Figure 3: Ex-vivo SARS-CoV-2 peptide-specific PBMC IFN-*γ* ELISpot responses after SARS-CoV-2 challenge by dose escalation group 26

Supplementary Figure 4: Baseline serum antibody inhibition of SARS-CoV-2 30

References 31

COVCHIM study Protocol 32

Methods

Procedures

Full study timeline with procedures is given in Figure 1. Prior to enrolment into the study, both Biofire film array (Respiratory 2.1 Plus Panel) (Biofire diagnostics, Utah, USA) and SARS-CoV-2 PCR (Thermo Fisher TaqPath assay, Thermofisher scientific, UK or CEPHEID GeneXpert, Cepheid UK Ltd, High Wycombe, UK) on a nasopharyngeal swab in viral transport media (Sigma Virocult, Medical wire equipment, Wiltshire, UK) were performed by Oxford University Hospitals NHS Trust (Oxford, UK) to exclude co-infection with common respiratory viruses and bacteria prior to inoculation (Figure 1). An additional SARS-CoV-2 PCR (page 9, main manuscript) on combined oro-pharyngeal-nasal swabs (Bioserv, Rotherham, UK) was undertaken at day -2 by hVIVO,UK. Volunteers were asked to self-isolate at home after the SARS-CoV-2 PCR on Day minus 5 until admission to the quarantine unit at Day minus 2. Intranasal inoculation on Day 0 occurred with the participant lying supine. Two drops of up to 50µL each of the SARS-CoV-2/human/GBR/484861/2020 virus were delivered to each nostril using a 100 µL pipette (Gilson, Dunstable, UK). Following inoculation volunteers remained supine for 10 minutes, followed by 20 minutes upright at 90 degrees.

Quarantine stays occurred in two isolation units; the first 4 volunteers enrolled into group 1A were quarantined at the John Radcliffe Hospital, Oxford. All subsequent enrolled volunteers were quarantined at the EMCRF, Oxford.

Quarantined volunteers had 24-hour monitoring, nursing and medical care. Mitigation measures defined in the protocol against the development of COVID disease and associated sequelae included the routine use of thromboembolism deterrent stockings and access to a pre-emptive rescue therapy. This was initially intravenous Regeneron (casirivimab and imdevimab monoclonal antibody cocktail) but this was changed to Paxlovid (Ritonavir boosted Nirmatrelvir), with the advent of oral anti-virals (Substantial Amendment (SA) 7). Initially for safety reasons, the criteria for use of rescue

therapy were deliberately very low and was to be given to individuals with “two consecutive positive PCR swabs for SARS CoV-2” irrespective of symptoms. Following accumulation of satisfactory safety data from the first 4 volunteers in our study and 10 volunteers in the seronegative study, the criteria were amended in the protocol with rescue therapy reserved for volunteers who demonstrated any warning features beyond mild signs and symptoms confined to the upper respiratory tract (i.e. persistent fever, persistent tachypnoea, persistent severe cough, greater than mild CT imaging changes or SaO2<94%).

CT chest imaging was performed post SARS-CoV-2 inoculation to identify any potential lower respiratory tract involvement. For $1x10^1$-$1x10^4$ dosing, all participants had a CT chest at day 5 regardless of infection status but a subsequent day 11 CT chest was only performed in infected participants or at clinician discretion. After the maximum dose escalation of $1x10^5$TCID50 was reached, following successful enrolment and quarantine of the first 5 participants at this dose, routine CT scanning at Day 5 was removed from the protocol (SA8) and replaced with optional CT scans (clinical concern for significant lower respiratory tract involvement only).

Cardiac MRI was performed at baseline in all participants and initially repeated between Day 14 and 28 in all participants for dosing groups $1x10^1$, $1x10^2$ and $1x10^3$TCID50, but after a protocol amendment (SA6) repeat cardiac MRI was only undertaken in those who were PCR positive outside the first 24 hours post- inoculation for dosing groups $1x10^4$ and $1x10^5$TCID50.

In addition to protocol defined follow up visits, volunteers were asked to attend an additional study visit within 5 days of a positive test for SARS-CoV-2 (PCR or lateral flow antigen test) in order to capture community acquired infection. Viral detection, safety assessment and samples for immunology were collected at this additional visit (Figure 1). A further post infection follow-up visit was scheduled 4-6 weeks after a community infection for immunological sampling. After the cessation of the NHS Lateral flow antigen and PCR testing programmes, volunteers were supplied

lateral flow antigen testing kits (Flowflex, Acon Laboratories, San Diego, USA) for use if they developed symptoms associated with COVID infection or were a household contact.

Virology

Flocked swabs (Bioserv, Rotherham, UK) from a combined oropharyngeal/nasal sample were used for detection of SARS-CoV-2 virus and placed in 3ml viral transport media (Bioserv, Rotherham UK). Swabs were kept refrigerated (2-8°C) after collection and transported with temperature monitoring to hVIVO, London, UK for analysis by qRT-PCR +/- FFA as previously described in Killingley et al(1). Swabbing was twelve-hourly during quarantine from the morning following challenge (Day 1) until the morning of Day 14 (discharge) and additionally at each outpatient follow up visit. For qRT-PCR each sample is tested in triplicate on the assay plate and the CT reported is the mean CT of the positive wells. All of the results that are reported as "detected" were required to have at least 2 of the 3 replicate wells on the PCR plate positive for virus detection. PCR positive swabs were further analysed by focus forming assay (FFA). CT values derived from PCR positive swabs were converted to copies per milliliter by comparison with a standard curve generated from linearized plasmid of known concentrations, containing sequences of the SARS-CoV-2 virus nucleocapsid and spike, run concurrently within the assay. Additional nasal swabs were collected on volunteers at community COVID-19 positive visits for whole genome sequencing.

Challenge Virus

The challenge virus; SARS-CoV-2/human/GBR/484861/2020 (a D614G-containing pre-alpha wild-type virus; Genbank Accession number OM294022) is the same as that used in the seronegative study(1). Details of isolation, GMP standard manufacture and release testing are outlined in Killingley et al(1). The virus was stored in a secure 24-hour monitored -80°C freezer and thawed at point of use. Volunteers were inoculated intranasally with virus from the supplied vial (200ul virus at a concentration between $5x10^{1}$-$5x10^{4}$ TCID50/ml or 178ul of the master virus batch at a concentration

of 5·618x10^5 TCID50). Challenge vials were provided pre-diluted with sucrose by the manufacturer and each vial was used for no more than 2 successive inoculations within 2 hours of thawing.

Assays methods

Whole Genome Sequencing of volunteers with community acquired COVID-19

Additional combined oropharyngeal/ nasal swabs were collected on volunteers at community COVID-19 positive visits and placed in viral transport medium (Sigma Virocult, Medical wire, Wiltshire, UK). Samples positive for SARS-CoV-2 were inactivated by adding an equal volume of TNA lysis buffer (Cat. OMEGTNA-1000, Omega Bio-tek, Inc., Nocross, Georgia, USA). 400µL of inactivated lysate was extracted manually using the Omega Biotek Mag-Bind Viral DNA/RNA Kit (Cat. M6246; Omega Bio-tek, Inc., Nocross, Georgia, USA). RNA was converted to cDNA using NEB LunaScript RT (Cat. E3010L, New England Biolabs, Ipswich, Massachusetts, USA) and 400bp amplicons were produced using the ARTIC method using NEB Q5 Hot Start High-Fidelity Master Mix (Cat. M0494X, New England Biolabs, Ipswich, Massachusetts, USA) and ARTIC V4·1 primers (Merck, Burlington, Massachusetts, USA)(2, 3), and a 63°C annealing/extension temperature. Amplicons were library prepped following the CoronaHiT-Illumina library preparation protocol(4) using IDT® for Illumina® DNA/RNA UD Indexes (Cat. 20027213, Illumina, San Diego, California, USA). The pooled final library was sequenced on an Illumina MiSeq (Illumina, San Diego, California, USA) generating ~1·3M read pairs/sample.

Samples were processed using the Artic SARS-CoV-2 nextflow pipeline(5). Consensus sequences generated at 10X minimum depth were used to call lineages using the latest Pangolin webtool(6, 7).

Immunological assessments

IFN-γ Enzyme-Linked Immunospot (ELISpot)

IFN-γ ELISpots were performed on freshly isolated PBMC two days prior to inoculation with SARS-CoV-2 and then at 2, 5, 7, 11 and 14 days post-inoculation and at all routine follow up visits. ELISpots were also performed on samples taken at community positive COVID visits and at the follow-up visit 4-6 weeks later. Briefly, ELISpot plates (Millipore, Merck Life Sciences Dorset, UK) were coated with 15 µg/ml IFN-γ capture mAb (Mabtech, Nacka Strand, Sweden) and incubated overnight at 4 °C. Plates were then washed 5 times with phosphate-buffered saline (PBS) and blocked for 2-5 h with RPMI (Sigma, Merck Life Sciences Dorset, UK) supplemented with 10% heat inactivated foetal calf serum, 1% penstrep (Sigma, Merck Life Sciences Dorset, UK), L-glutamin and Sodium Pyruvate (R10). 2·5 x 10^5 PBMC in 80 µl R10 were added to each well and 20 µl of SARS-CoV-2 peptide pools were added to triplicate wells. Peptide pools tested included the S1 and S2 subunit of the spike protein, membrane protein (M), nucleoprotein (NP), ORF3, ORF6, ORF7, ORF8 and ORF10 (Mimotopes, (UK) Ltd. Wirral, UK). The above peptide pools were tested at a final concentration of 10 µg/ml per peptide per well. SARS-CoV-2 CD4-E and CD8-E peptide pools were also tested. These are experimentally defined SARS-CoV-2 epitopes for the most immunodominant SARS-CoV-2 proteins, identified in CD4+ and CD8+ T cells respectively (La Jolla Institute for Immunology, CA, USA) and were used at a concentration of 1 µg/ml per peptide per well. Anti-human CD3 mAb (Mabtech, Nacka Strand, Sweden) was used as a positive control (10 ng/ml) and unstimulated PBMC were used as a measure of background IFN-γ production.

ELISpot plates were incubated at 37°C, 5% CO2 for 18-20 h before developing and reading on an AID ELISpot reader with ELISpot7·0iSpot software. Results are presented as spot forming cells (SFC) per million PBMC, calculated by subtracting the mean of triplicate unstimulated wells from the mean of triplicate test wells and correcting for number of PBMC in the well.

Nasal lining fluid collection (NLF) for mucosal immunology

Nasal lining fluid was collected via nasosorption using synthetic absorptive matrix (SAM) strips (Nasosorption™ FX-I-11, Hunt Developments, UK). Samples were collected by placing the SAM strips adjacent to the inferior nasal turbinate for 1 minute, 1 strip in each nostril, with a nose clip attached. SAMs were collected at baseline, 12 hourly for the first 72 hours and then at Days 5, 7, 11, 14 and at follow up visits and community-acquired COVID-19 positive visits. Strips were snap frozen and stored at -80°C prior to batch elution. Elution was as previously described, using 300µl Diluent 100 (Meso Scale Diagnostics, Rockville, USA) and filter tube inserts without a membrane (Corning, New York, USA)(8). Results from baseline pre-inoculation samples only are presented here. Analysis of later timepoints is ongoing.

Meso Scale Discovery (MSD®) IgG, IgA and IgM binding Assays

IgG, IgA and IgM responses to SARS-CoV-2 were measured using a multiplexed MSD® immunoassay: The V-PLEX COVID-19 Coronavirus Panel 2 Kit (cat. no. K15369U, K15370U, K15371U) from Meso Scale Diagnostics, Rockville, MD USA. A MULTI-SPOT® 96-well, 10 spot plate was coated with four SARS-CoV-2 antigens (Spike (S), Receptor-Binding Domain (RBD), Nucleoprotein (NP), N-terminal Domain (NTD)) and SARS-CoV-1 spike trimer, as well as spike proteins from seasonal human coronaviruses, OC43, HKU1, 229E and NL63, and bovine serum albumin. SARS-CoV-1 and the seasonal coronavirus responses will be reported in a subsequent manuscript alongside post-inoculation analyses. Antigens were spotted at 200–400 µg/mL (MSD® Coronavirus Plate 2). Multiplex MSD® assays were performed as per the instructions of the manufacturer. To measure IgG, IgA and IgM binding antibodies, 96-well plates were blocked with MSD® Blocker A for 30 minutes. Following washing with washing buffer, plasma samples diluted 1:1,000-10,000 and NLF samples diluted 1:25-1:200 in diluent buffer were added to wells, along with MSD® standard or undiluted MSD® internal controls. After a 2-hour incubation and a washing step, detection antibody (MSD SULFO-TAG™ Anti-Human IgG, IgA or IgM Antibody, 1/200) was added. Following washing, MSD

GOLD™ Read Buffer B was added and plates were read using a MESO® SECTOR S 600 Reader. The standard curve was established by fitting the signals from the standard using a 4-parameter logistic model. Concentrations of samples were determined from the electrochemiluminescence signals by back-fitting to the standard curve and multiplied by the dilution factor. Concentrations are expressed in Arbitrary Units/ml (AU/ml). Cut-offs for serum samples were determined for each SARS-CoV-2 antigen (S, RBD, N, NTD) based on 64 pre-SARS-CoV-2 pandemic sera (median concentration + 3xStandard Deviation for IgG, IgA and IgM binding). Pre-pandemic serum samples were sourced from the Scottish National Blood Transfusion Service (SNBTS) in 2019. Ethical approval was obtained from the SNBTS anonymous archive, IRAS project number 18005, and fully informed consent for virological testing was obtained from the donors. Cut-offs for NLF samples were calculated in the same way as the serum samples using 5 pre-SARS-CoV-2 pandemic NLF samples (Supplementary Table 5B, page 18), supplied by Dr Ryan Thwaites from Imperial College London, UK.

Meso Scale Discovery (MSD®) ACE2 inhibition surrogate neutralisation assay

V-PLEX SARS-CoV-2 Panel 13 Kit (cat. no. K15466U) was used to measure the ability of serum or SAM samples to inhibit angiotensin-converting enzyme 2 (ACE2) binding to different variants of SARS-CoV-2 spike. Assays were performed as per manufacturer's instructions with sera and SAM samples. To measure ACE2 inhibition, 96-well MSD® plates were blocked with MSD® Blocker for 30 minutes. Plates were then washed in MSD® washing buffer, and 25 µl of diluted serum or SAM samples (1:10-1:100) were added to the plate. After 1-hour incubation, recombinant human ACE2-SULFO-TAG™ was added to all wells. After a further 1-hour, plates were washed and MSD GOLD™ Read Buffer B was added, plates were then immediately read using a MESO® SECTOR S 600 Reader. Neutralising activity was determined by measuring the presence of antibodies able to block the binding of ACE2 to SARS-CoV-2 spike proteins from Wuhan-Hu-1 spike, B.1.1.7/Alpha, B.1.351/Beta, P.1/Gamma and B.1.617.2/Delta. An ACE2 calibration curve which consists of a monoclonal antibody with equivalent activity against spike variants was used to interpolate results as arbitrary units (AU).

NLF samples were below the limit of detection for the assay and hence only serum results are presented here.

Microneutralisation assay

Sera were serially diluted in DMEM supplemented with 1% FBS from an initial dilution of 1:10 to 1:10,000. Equal volumes of diluted sera and SARS-CoV-2 virus (approximately 100 focus forming units) were combined and incubated for 30 minutes. Viruses used in this assay were Victoria, Alpha (B.1.1.7), Delta (B.1.617.2) and Omicron (B.1.1.529 BA.1). Following incubation 100µl Vero E6 cells (supplied by Public Health England (PHE) Porton Down) (4·5x10^5/ml) was added to each well and virus was allowed to infect the cells for 2 hours at 37°C, 5% CO_2 followed by the addition of 100 µl Carboxymethyl cellulose (1·5%) to each well. The plates were incubated for a further 20 hours at 37°C, 5% CO_2. All assays were carried out in duplicate.

Cells were washed with 200 µl DPBS and then fixed with paraformaldehyde 4% v/v (100 µl/well) for 30 minutes at room temperature. Cells were permeabilised with TritonX100 (1% in PBS) and then stained for SARS-CoV-2 nucleoprotein using a human monoclonal antibody (FB9B)(9). Bound antibody was detected following incubation with a goat anti-human IgG HRP conjugate (Sigma, UK) and following TrueBlue™ Peroxidase substrate (Insight Biotechnology, London, UK) addition imaged using an ELISPOT reader. The half-maximal inhibitory concentration (IC50) was defined as the concentration of sera that reduced the Foci forming unit (FFU) by 50% compared to the control wells, as calculated using Graphpad Prism 10.

Prototype isolate (Pango lineage B) was Victoria/01/2020, received at P3 from Public Health England (PHE) Porton Down (after being supplied by The Peter Doherty Institute for Infection and Immunity, University of Melbourne).

Alpha (B.1.1.7) was supplied by Public Health England (PHE) Porton Down.

Delta (B.1.617.2) isolate 83DJ-1 was provided by Piet Maes, Laboratory of Clinical and Epidemiological Virology (Rega Institute), KU Leuven, 3000 Leuven, Belgium.

OMICRON (BA.1.1.529), Crick 99 (hCoV/England/FCI-099/2021) was provided by the Crick Institute, London.

Supplementary Tables

Supplementary Table 1: All positive quarantine PCR results

Dose (TCID50)	Vol no.	Vaccinated	Time between last vaccination and enrolment (days)	Infection to enrolment (days)	Anti S Abs (AU/ml)	Anti N Abs	Timepoint	Viral load (Log_{10} copies/mL)	Ct Value	FFA (Log_{10} FFU/mL)
$1x10^1$	00101002	No	N/A	225	78·40	Negative	Day 6 PM	3·331	34·6	Not detected
							Day 7 AM	3·640	33·6	1.82
	00101003	No	N/A	221	221·0	Equivocal	Day 6 PM	3·717	33·3	Not detected
							Day 7 AM	3·585	33·8	Not detected
$1x10^2$	00101008	x 2	37	331	36,604·20	Equivocal	Day 1 AM	4·296	31·2	Not detected
$1x10^3$	00101026	x 1	62	120	>40,000	Positive	Day 1 AM	Detected	37·1	Not detected
	00101025	x 1	39	216	17,959·20	Negative	Day 3 PM	3·545	33·9	Not detected
	00101029	x 2	75	433	11,721·80	Negative	Day 1 AM	4·687	30·1	Not detected
$1x10^4$	00101035	x 3	88	444	28,981	Negative	Day 2 PM	3·512	34·2	Not detected
							Day 3 AM	3·838	32·8	Not detected
	00101039	x 2	84	217	39,536	Positive	Day 1 AM	3·703	34·3	Not detected
							Day 1 PM	3·803	33·6	Not detected
							Day 2 AM	3·576	34·6	Not detected
	00101041	x 2	240	520	6,949	Positive	Day 1 AM	3·839	33·9	Not detected
	00101044	x 3	75	111	>40,000	Equivocal	Day 1 AM	Detected	36·8	Not detected
$1x10^5$	00101064	x 3	179	225	5275·70	Positive	Day 1 AM	5·519	27·6	Not detected
							Day 1 PM	4·357	31·8	Not detected
	00101068	x 3	231	668	8189·20	Negative	Day 1 AM	3·810	33·5	Not detected
	00101066	x 3	254	219	4742·40	Detected	Day 1 AM	4·207	32·4	Not detected
	00101067	x 3	274	100	7996·40	Detected	Day 1 PM	4·923	29·6	Not detected

							Day 2 AM	3·643	34·4	Not detected
	00101074	x 3	296	538	4167·50	Equivocal	Day 1 AM	5·556	27·5	Not detected
	00101080	x 3	614	300	2583·50	Equivocal	Day 1AM	4·378	32·2	Not detected
							Day 1PM	4·139	32·5	Not detected
							Day 2AM	4·065	32·7	Not detected
							Day 2PM	3·361	34·2	Not detected
							Day 3PM	4·194	32·8	Not detected
							Day 5AM	4·846	29·8	Not detected
							Day 6AM	Detected	36·4	Not detected
							Day 7AM	Detected	37·1	Not detected
	00101081	x 2	319	340	8239·30	Detected	Day 1PM	3·944	33·1	Not detected
	00101086	x 3	341	200	5875·70	Detected	Day 1AM	3·952	33·7	Not detected
							Day 2AM	Detected	35·221	Not detected

Transient infection volunteers highlighted in blue. The LLOQ for qPCR was 3 log10 copies per milliliter, with positive detections less than the LLOQ assigned a value of 1·5 log10 copies per milliliter and undetectable samples assigned a value of 0 log10 copies per milliliter

Supplementary Table 2: Summary of community acquired COVID infections up to 23rd December, 2022.

G1 =Grade 1, Mild symptoms; G2= Grade 2, moderate symptoms, G3= Grade 3, severe symptoms.

Dose (TCID50)	Volunteer	Vaccine status at onset of COVID infection	Infection status during quarantine	Date of onset of symptoms	Date of positive test (PCR or LFT)	COVID positive visit	Viral load (Log_{10} copies/mL)	CT value	FFA (Log_{10} FFU/mL)	Variant Sequencing	Symptoms?
$1x10^1$	001	Boosted: BNT162b2 28/07/21, 22/09/21, 16/12/21	Uninfected	20/02/22 (Day 241)	21/2/22	24/02/22	6·506	23·6	Detected	Omicron BA.1	G1 Cough<br>G1 Tickly throat<br>G1 Runny nose<br>G1 Fatigue/ malaise
	002	Boosted: mRNA-1273 20/09/21, 07/10/21 BNT162b2 06/01/22	Transient Infection	27/01/22 (Day 245)	27/1/22	01/02/22	Detected	35·6	Not detected	Sequencing failed*	G1 Productive cough<br>G1 Sore throat<br>G1 Fatigue<br>G1 Headache<br>G1 Malaise
$1x10^2$	008	Full course: BNT162b2 03/7/21, 31/8/21	Uninfected (Day 1AM residual inoculum only)	16/12/21 (Day 70)	16/12/21	21/12/21	4·340	31·6	Not detected	Omicron BA.1	G1 fatigue<br>G1 joint pain<br>G1 ear ache<br>G1 SOB (exertion only)<br>G1 rash (reported 7/1/22
	016**	Boosted: ChAdOx1 nCoV-19 13/02/21, 22/05/21 & mRNA-1273 20/11/21	Uninfected	Asymptomatic infection, picked up at routine Day 365 study visit (18/8/22)	18/08/22	N/A	Detected	35·2	Not detected	N/A	Asymptomatic
	017 (1 of 2)	Full course: BNT162b2 29/06/21, 18/10/21	Uninfected	17/01/22 (Day 123)	18/01/22	19/01/22	7·760	18·6	Not detected	Omicron BA.1	G1 cough (with phlegm)<br>G1 SOB<br>G1 sore throat<br>G1 tickly throat<br>G1 stuffy nose<br>G1 hoarse voice<br>G1 sneezing<br>G1 fatigue<br>G1 headache<br>G1 malaise
	017 (2 of 2)	Full course: BNT162b229/06/21, 18/10/21	Uninfected	12/08/22 (Day 336)	16/08/22	18/08/22	Detected	35·8	Not detected	Omicron BF.7	G1 cough (dry)<br>G1 SOB<br>G1 sore throat/hoarse voice<br>G1 feverishness<br>G1 fatigue<br>G1 Headache<br>G1 Malaise
	021	Full course: BNT162b2 11/6/21, 20/8/21	Uninfected	30/12/21 (Day 84)	30/12/21	04/01/22	5·875	25·9	Not detected	Omicron BA.1	G1 cough<br>G1 SOB<br>G1 tickly throat<br>G1 chest tightness

Dose (TCID50)	Volunteer	Vaccine status at onset of COVID infection	Infection status during quarantine	Date of onset of symptoms	Date of positive test (PCR or LFT)	COVID positive visit	Viral load (Log_{10} copies/mL)	CT value	FFA (Log_{10} FFU/mL)	Variant Sequencing	Symptoms?
											G1 runny nose<br>G1 hoarse voice
	024	Unvaccinated	Uninfected	21/09/22<br>(Day 318)	21/9/22	22/09/22	6·288	25·3	Not detected	Omicron BA.5	G1 stuffy nose<br>G1 runny nose<br>G1 hoarse voice<br>G2 sneezing<br>G1 fatigue<br>G1 malaise<br>G1 diarrhoea
$1x10^3$	025	Single: BNT162b2<br>26/09/21	Transient Infection (D3PM)	06/03/22<br>(Day 122)	08/03/22	12/03/22	6·715	22·7	Not detected	Omicron BA.2	G1 Cough productive of phlegm<br>G2 Sore throat<br>G1 Tickly throat<br>G1 runny and stuffy nose<br>G1 Fever<br>G2 Feverishness<br>G2 joint and muscle aches<br>G2 Fatigue and malaise<br>G1 Headache<br>G1 Nausea<br>G1 Hyposmia<br>G1 Red eyes (from feeling unwell, no discharge)
	026	Single: mRNA-1273<br>03/09/21	Uninfected (Day 1AM residual inoculum only)	09/12/21<br>(Day 35)	11/12/21	15/12/21	5·818	26·0	Not detected	Omicron BA.1	G1 stuffy nose<br>G1 runny nose<br>G1 rash<br>G1 fatigue<br>G1 aguesia<br>G1 anosmia (reported post visit)
	029**	Full course: BNT162b2<br>8/6/21, 23/10/21	Uninfected (Day 1AM residual inoculum only)	23/02/22<br>(Day 48)	23/2/22	09/03/22<br>(Day 56 visit)	Negative (volunteer at home LFD was positive on 23/02/22)	Negative	Negative	N/A	G2 Cough<br>G1 Phlegm<br>G1 SOB<br>G1 stuffy nose<br>G1 runny nose<br>G1 hoarse voice<br>G1 Sneezing<br>G1 Feverishness<br>G1 Muscle aches<br>G1 Fatigue<br>G1 Earache

Dose (TCID50)	Volunteer	Vaccine status at onset of COVID infection	Infection status during quarantine	Date of onset of symptoms	Date of positive test (PCR or LFT)	COVID positive visit	Viral load (Log_{10} copies/mL)	CT value	FFA (Log_{10} FFU/mL)	Variant Sequencing	Symptoms?
											Reduction in sense of smell (smell test was unchanged from baseline at visit)<br>Alteration in sense of taste
$1x10^4$	035**	Boosted: BNT162b2 15/01/21, 11/03/21, 12/21/21	Transient Infection (D2PM, D3AM)	26/07/22 (Day 133)	26/07/22 (LFD)	N/A participant had positive LFD whilst abroad		N/A	N/A	N/A	G1 Cough<br>G1 Phlegm<br>G1 Sore throat<br>G1 Stuffy nose<br>G1 Headache<br>G1 Diarrhoea<br>G1 Thirstiness
	039	Boosted: mRNA-1273 15/06/21, 01/09/21, 16/12/21	Uninfected (Day D1AM, D1PM, D2AM residual inoculum only)	25/05/22 (Day 76)	26/5/22	31/05/22 (Day 84)	6·036	25·7	Not detected	Omicron BA.5	G1 Sore throat
	045**	Full course: BNT162b2 24/06/21, 23/08/21	Uninfected	09/06/22 (Day 70)	9/6/22	N/A (only had positive LFD in the community and did not contact study team)	N/A	N/A	N/A	N/A	G1 Cough<br>G1 Phlegm<br>G1 Sore throat<br>G1 Stuffy nose<br>G1 Runny nose<br>G1 Sneezing<br>G1 Fever
$1x10^5$	074	Boosted: BNT162b2 10/06/21, 05/08/21; mRNA-1273 20/12/21	Uninfected (Day 1AM residual inoculum only)	03/12/22 (Day 52)	13/12/22	N/A incidental finding at routine Day 56 visit (13/12/22). Serial LFD negative at home.	3·716	33·6	Not detected	Omicron BR.2.1	G2 cough<br>G2 sore throat<br>G2 nasal congestion<br>G2 sneezing<br>G2 fever<br>G2 headache

Full course refers to two vaccinations or one Ad26.COV2.S (Johnson & Johnson). Boosted refers to full course plus booster dose.

G = Grade. Grade 1 = mild discomfort, does not interfere with regular activities, Grade 2 = moderate discomfort with mild to moderate limitation in activity, Grade 3 = Severe or significant discomfort with marked limitation in activity.

Vaccines: BNT162b2 (Pfizer-BioNTech), mRNA-1273 (Moderna), ChAdOx1 nCoV-19 (Oxford-AstraZeneca)

* Sequencing failed due to low viral loads

***Volunteers 016, 029,035,045 did not attend for formal COVID positive visits due to logistical issues noted in the table above and therefore had no sampling for IFNγ ELIspot responses (figure 4D*

Supplementary Table 3: All laboratory AEs deemed possibly, probably or definitely related to SARS-CoV-2 inoculation by dose group *showing AEs reported by individual volunteers by grade and timepoint and the total number of volunteers demonstrating a lab AE of a given grade in each dose group. The grade 1 lymphocytes at D28 in the 10^1TCID50 group resolved at Day 84.*

	TIMEPOINT						Number of individual volunteers
	D2	D5	D7	D11	D14	D28	
**10^1TCID50**							
**Decreased Lymphocytes**							
Grade 1	-	-	-	1	1	1	2
**Decreased Neutrophils**							
Grade 1	-	1	1	-	1	-	1
**Decreased Platelets**							
Grade 1	-	-	-	-	1	-	1
Grade 2	-	-	-	1	-	-	1
**Decreased WBC**							
Grade 1	-	1	1	1	1	-	1
**Elevated Bilirubin**							
Grade 1	1	1	-	-	-	-	2
**10^3TCID50**							
**Elevated Bilirubin**							
Grade 1	-	-	1	1	-	-	1
Grade 2	-	1	-	-	-	-	1
**10^5TCID50**							
**Decreased Absolute Hb**							
Grade 1	1	-	-	-	-	-	1
**Elevated Bilirubin**							
Grade 1	1	-	-	1	-	-	1

Supplementary Table 4: **MedDRA coded AEs following discharge from quarantine** Showing a*ll AEs reported by ≥10% of volunteers following discharge from quarantine by maximum grade and all severe AEs regardless of frequency up to a data analysis cut-off date of 23rd December 2022. The number of AEs thought to be possibly, probably or definitely related to SARS-CoV-2 inoculation are shown in the right-hand column. All volunteers followed up to at least Day 28. Percentages are shown in brackets.*

All AEs post discharge reported by ≥10% of volunteers by maximum grade and all severe AEs regardless of frequency	nAEs by dose group (%)						No. possibly, probably, definitely related to SARS-CoV-2 inoculation
	10^1 (N=8)	10^2 (N=7)	10^3 (N=6)	10^4 (N=7)	10^5 (N=8)	Total (N=36)	
**General disorders and administration site conditions**							
**Fatigue**							
Mild	3	2	1	2	—	8 (22)	1[a]
Moderate	—	1	—	1	—	2 (6)	0
**Influenza like illness**							
Mild	—	2	—	—	1	3 (8)	0
Moderate	1	—	1	5	—	7 (19)	0
**Infections and infestations**							
**COVID-19**							
Mild	1	3	1	1	—	6 (17)	0
Moderate	1	1	2	2	1	7 (19)[b]	0
**Gastroenteritis**							
Moderate	—	1	—	—	—	1 (3)	0
Severe	—	—	1	—	—	1 (3)	0
**Infection**							
Severe	1	—	—	—	—	1 (3)	0
**Influenza**							
Severe	—	—	—	—	1	1 (3)	0
**Tonsillitis**							
Severe	1	—	—	—	—	1 (3)	0
**Injury, poisoning and procedural complications**							
**Post vaccination syndrome**							

Mild	3	—	1	—	—	4 (11)	1
Moderate	2	1	—	—	—	3 (8)	0
**Musculoskeletal and connective tissue disorders**							
**Myalgia**							
Mild	—	1	1	1	1	4 (11)	0
Moderate	1	—	—	—	—	1 (3)	0
**Pain in extremity**							
Mild	—	1	—	—	—	1 (3)	0
Moderate	—	3	—	—	—	3 (8)	0
**Nervous system disorders**							
**Headache**							
Mild	—	—	—	1	1	2 (6)	0
Moderate	2	3	1	2	—	8 (22)	0
**Psychiatric disorders**							
**Depression**							
Moderate	—	—	—	1	—	1 (3)	0
Severe	—	—	1	—	—	1 (3)	0
**Respiratory, thoracic and mediastinal disorders**							
**Cough**							
Mild	—	—	1	2	2	5 (14)	0
Moderate	2	—	1	—	—	3 (8)	0
**Nasal congestion**							
Mild	—	—	1	1	1	3 (8)	0
Moderate	1	—	—	—	—	1 (3)	0
**Upper respiratory tract infection**							
Mild	3	4	2	2	—	11 (31)	0
Moderate	—	1	—	—	—	1 (3)	0

[a]Mild fatigue possibly related to SARS-CoV-2 inoculation was reported at Day 19 and lasted for 1 day only

[b]14 participants contracted COVID-19 post discharge, 1 is not reported here as they were asymptomatic.

Supplementary Table 5: Positive cut off values for A. serum and B. nasal lining fluid (NLF) calculated from pre-pandemic samples*

A. serum	Cov-2 NTD	Cov-2 RBD	Cov-2N	Cov-2S
**IgG**	32·55316	1865·637	2957·245	1120·589
**IgA**	565·7463	999·2172	1358·686	505·3715
**IgM**	53·94288	977·4923	4958·536	344·734

B. NLF	CoV-2 NTD	Cov-2 RBD	Cov-2N	Cov-2S
**IgG**	0·043205	4·082876	60·74194	1·696057
**IgA**	8·254826	13·11191	13·4347	10·45779
**IgM**	0·385779	5·950349	10·06802	1·285882

*Pre-pandemic serum samples were sourced from the Scottish National Blood Transfusion Service (SNBTS) in 2019.and pre-pandemic NLF samples were provided by Dr Ryan Thwaites, Imperial College London, UK. Ethical approval was obtained from the SNBTS anonymous archive, IRAS project number 18005, and fully informed consent for virological testing was obtained from the donors.

Supplementary Table 6: Volunteer vaccine status and primary infection variant at screening compared with microneutralisation assay (MNA) using baseline serum

IC50: Half maximal inhibitory concentration; SGTF: S-Gene target Failure. Orange denotes volunteers with subsequent transient infection in quarantine. Sera from volunteers who subsequently received either 1x10[1] or 1x10[2] TCID50 was not tested against omicron infection given it was not in circulation at the time of serum collection. Full course refers to two vaccinations or one Ad26.COV2.S (Johnson & Johnson). Boosted refers to full course plus booster dose. Vaccines: BNT162b2 (Pfizer-BioNTech), mRNA-1273 (Moderna), ChAdOx1 nCoV-19 (Oxford-AstraZeneca, Ad26.COV2.S (Johnson &Johnson). Variant of primary infection based on UK prevalence data at time of volunteer infection(10) and SGTF or sequencing data from PHE (where available), with SGTF used as a proxy identification for Alpha & Omicron variants.

Dose (TCID50)	Vol no.	Date of primary infection	Date of enrolment	Time from primary infection to D0 (days)	Anti-S antibodies (AU/ml)	Anti-N antibodies	Vaccine status at time of enrolment (baseline)	Variant of primary infection	MNA: IC50			
									B.1 (Victoria)	Alpha	Delta	Omicron
**1x10[1]**	**02**	**14/10/2020**	**27/05/21**	**225**	**78·40**	**Negative**	**Unvaccinated**	**Wuhan-like strain**	**72·1**	**88·3**	**<20**	
	**03**	**18/10/2020**	**27/05/21**	**221**	**221·00**	**Equivocal**	**Unvaccinated**	**Wuhan-like strain (No SGTF)**	**67·8**	**157·0**	**46·8**	
	01	27/12/2020	24/06/21	179	668·20	Negative	Unvaccinated	Alpha/ Wuhan-like strain	247·6	434·8	121·7	
	09	31/01/2021	24/06/21	144	143·70	Equivocal	Unvaccinated	Alpha/ Wuhan-like strain	29·7	<20	<20	
	04	06/11/2020	22/07/21	258	175·80	Equivocal	Unvaccinated	Wuhan-like strain/Alpha	60·1	<20	<20	
	05	11/11/2020	22/07/21	253	121·30	Negative	Unvaccinated	Wuhan-like strain/Alpha	26·8	<20	<20	
	10	20/12/2020	22/07/21	214	110·80	Equivocal	Unvaccinated	Alpha (SGTF)	24·0	134·6	<20	
	12	25/12/2020	22/07/21	209	596·70	Equivocal	Unvaccinated	Alpha (SGTF)	174·4	361·0	389·6	
**1x10[2]**	016	27/12/2020	19/08/2021	235	5,028·10	Equivocal	Full course: ChAdOx1 nCoV-19	Alpha/ Wuhan-like strain	832	1035	339	

Dose (TCID50)	Vol no.	Date of primary infection	Date of enrolment	Time from primary infection to D0 (days)	Anti-S antibodies (AU/ml)	Anti-N antibodies	Vaccine status at time of enrolment (baseline)	Variant of primary infection	MNA: IC50			
									B.1 (Victoria)	Alpha	Delta	Omicron
							03/21 & 22/05/21					
	017	30/9/2020	16/09/2021	351	16,950·40	Negative	Single: BNT162b2 29/06/21	Wuhan-like strain (No SGTF)	1492	884	443	
	008	10/11/2020	07/10/2021	331	36,604·20	Equivocal	Full course; BNT162b2 3/7/21 & 31/8/21	Wuhan-like strain or Alpha	4487	2895	1111	
	018	24/11/2020	07/10/2021	317	13,047·70	Positive	Full course: BNT162b2 13/6/21 & 21/8/21	Wuhan-like strain (No SGTF)	2728	1157	706	
	021	01/10/2020	07/10/2021	371	17,473·50	Equivocal	Full course: BNT162b2 18/6/21 & 20/8/21	Wuhan-like strain	2748	1902	630	
	022	15/01/2021	07/10/2021	265	14,653·70	Equivocal	Full course: BNT162b2 13/5/21 & 5/7/21	Alpha or Wuhan-like strain	1117	1873	642	
	024	12/06/21	04/11/2021	145	242·40	Positive	Unvaccinated	Alpha (SGTF)	<20	<20	<20	
**1x10^3**	**025**	**02/04/21**	**04/11/21**	**216**	**17,959·20**	**Negative**	**Single: BNT162b2 26/09/21**	**Alpha/ Delta**	**1553·1**	**663·0**	**209·3**	**175·5**
	026	07/07/21	04/11/21	120	>40,000	Positive	Single: mRNA-1273 3/9/21	Delta (sequenced)	1937·3	928·4	919·5	126·1
	031	13/11/20	02/12/21	384	3,276·90	Negative	Full course: BNT162b2 16/6/21 & 8/7/21	Wuhan-like strain or Alpha	462·0	177·2	56·1	<20
	029	30/10/20	06/01/22	433	11721·80	Negative	Full course: BNT162b2 8/6/21 & 23/10/21	Wuhan-like strain	1393·0	665·5	217·7	62·5
	033	29/06/21	06/01/22	191	30,964·30	Positive	Full course: mRNA-1273 23/7/21 & 19/9/21	Delta (No SGTF)	1335·9	746·0	552·3	143·8
	034	28/09/20	06/01/22	465	8980·30	Negative	Boosted: BNT162b2 19/06/21,	Wuhan-like strain (No SGTF)	884·1	590·3	285·2	32·5

Dose (TCID50)	Vol no.	Date of primary infection	Date of enrolment	Time from primary infection to D0 (days)	Anti-S antibodies (AU/ml)	Anti-N antibodies	Vaccine status at time of enrolment (baseline)	Variant of primary infection	MNA: IC50			
									B.1 (Victoria)	Alpha	Delta	Omicron
							Ad26.COV2.S 29/7/21					
**1x10^4**	**035**	**21/12/20**	**10/03/22**	**444**	**28,981**	**Negative**	**Boosted: BNT162b2 15/1/21, 11/3/21 & 12/12/21**	**Alpha or Wuhan-like strain**	**2538·3**	**2449·1**	**800·9**	**579·0**
	039	05/08/21	10/03/22	217	39,536	Positive	Boosted: mRNA-1273 15/6/21, 01/09/21 & 16/12/21	Delta (No SGTF)	2031·6	2892·2	1240·1	644·7
	041	6/10/20	10/03/22	520	6,949	Positive	Full course: BNT162b2 20/6/21 & 13/7/21	Wuhan-like strain	1166·2	1012·1	533·7	218·9
	036	01/11/20	31/03/22	515	8,381	Negative	Full course: mRNA-1273 13/7/21 & 7/9/21	Wuhan-like strain or Alpha	745·1	1011·7	403·4	191·8
	044	10/12/21	31/03/22	111	>40,000	Equivocal	Boosted: BNT162b2 25/6/21, 3/8/21, & 15/1/22	Omicron (BA.1) or Delta	3017·6	3734·9	1004·2	1148·8
	045	08/10/20	31/03/22	539	8,853	Negative	Full course: 21 BNT162b2 24/6/21 & 23/8/21	Wuhan-like strain	1129·9	1017·6	438·6	254·0
	047	27/10/20	31/03/22	520	14,783	Equivocal	Full course: mRNA-1273 15/12/21, 6/3/22	Wuhan-like strain	1902·9	1168·7	567·5	140·0
**1x10^5**	064	30/12/21	11/08/22	224	5275·70	Positive	Boosted: ChAdOx1 nCoV-19 28/03/21, 17/06/21; mRNA-1273 14/02/22	Omicron (BA.1) or Delta	687·2	461·4	158·9	397·8
	066	07/01/21, 10/02/22	22/09/22	623, 224	4742·40	Detected	Boosted: BNT162b2	Alpha or Wuhan-like strain,	800·1	365·2	111·3	476·4

Dose (TCID50)	Vol no.	Date of primary infection	Date of enrolment	Time from primary infection to D0 (days)	Anti-S antibodies (AU/ml)	Anti-N antibodies	Vaccine status at time of enrolment (baseline)	Variant of primary infection	MNA: IC50			
									B.1 (Victoria)	Alpha	Delta	Omicron
							13/06/21, 04/08/21, 11/01/22	Omicron (BA.1 or BA.2)				
	067	04/07/22	12/10/22	100	7996·40	Detected	Boosted: BNT162b2 25/05/21, 21/07/21, 21/12/21	Omicron (BA.2, BA.4 or BA.5)	2145·0	1471·7	233·2	411·1
	068	03/11/20	01/09/22	667	8189·20	Negative	Boosted: BNT162b2 24/06/21, 20/07/21, 14/01/22	Wuhan-like strain or Alpha	1285·9	589·7	120·7	269·8
	074	22/04/21	12/10/22	538	4167·50	Equivocal	Boosted: BNT162b2 10/06/21, 05/08/21; Modena 20/12/21	Delta (No SGTF)	640·8	565·6	119·1	129·1
**080**	**29/01/22**	**24/11/22**	**299**	**2583·50**	**Equivocal**	**Boosted: ChAdOx1 nCoV-19 30/04/20, 23/08/20, 21/03/21**	**Omicron (BA.1 or BA.2)**	**371·8**	**345·1**	**103·0**	**191·0**
	081	20/12/21	24/11/22	339	8239·30	Detected	Full course: BNT162b2 10/09/21, 10/01/22	Delta	1272·3	1101·3	292·0	484·1
	086	06/10/20, 29/01/22	24/11/22	779, 299	5875·70	Detected	Boosted: BNT162b2 23/06/21, 31/07/21, 19/12/21	Wuhan-like strain, Omicron (BA.1 or BA.2)	881·7	1071·8	248·0	591·8

Supplementary Figure 1: Solicited AEs in quarantine

Percentage of all volunteers reporting solicited symptoms during the quarantine period by the maximum grade reported.

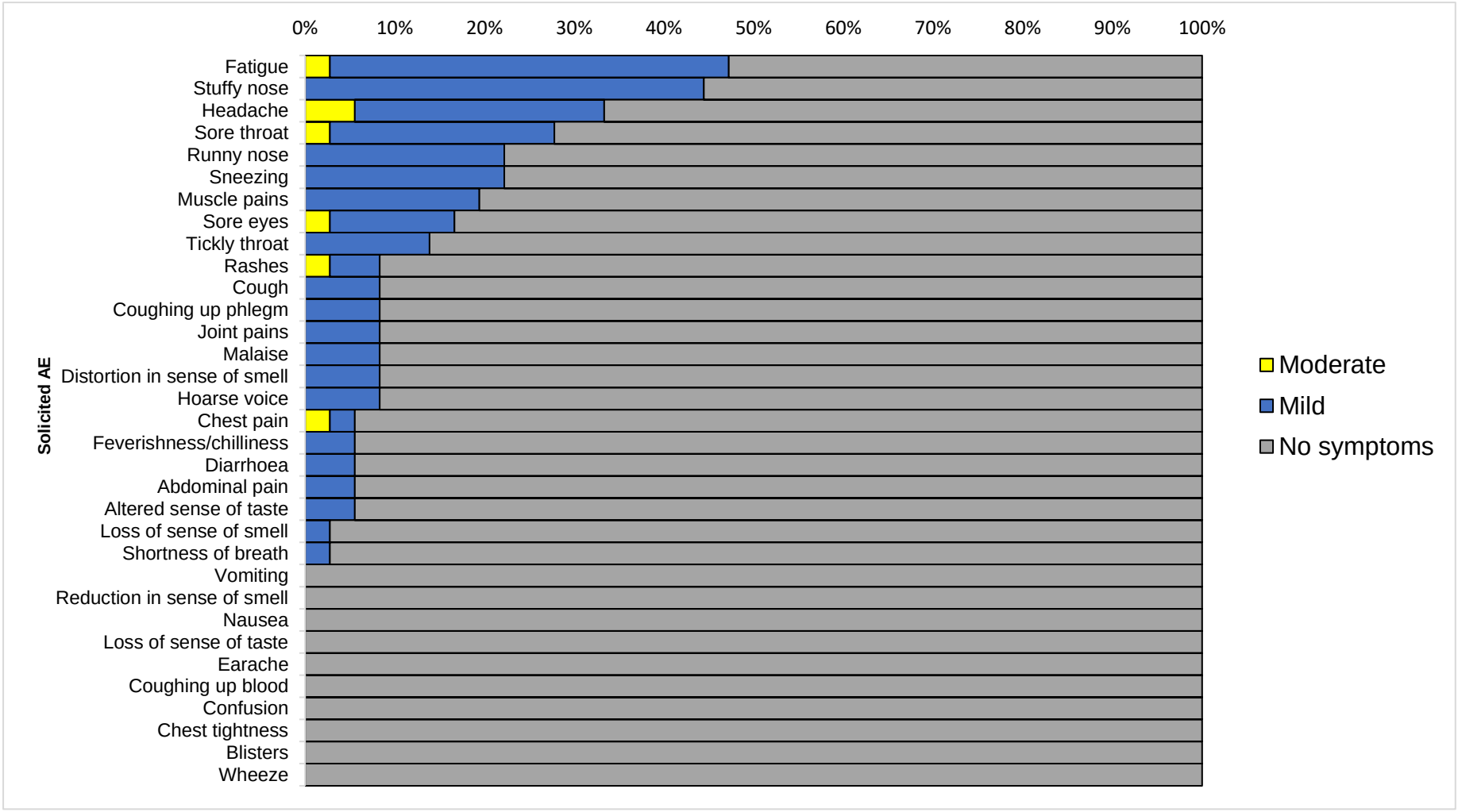

Supplementary Figure 2: Solicited symptoms in transiently infected volunteers

Solicited symptoms reported by transiently infected volunteers in relation to SARS CoV-2 detection on PCR and use of rescue therapy (intravenous casirivimab and imdevimab monoclonal antibody cocktail) for volunteers 002 and 3. Volunteer 080 reported mild AEs (rash and stuffy nose) just prior to discharge but these had resolved by Day 18.

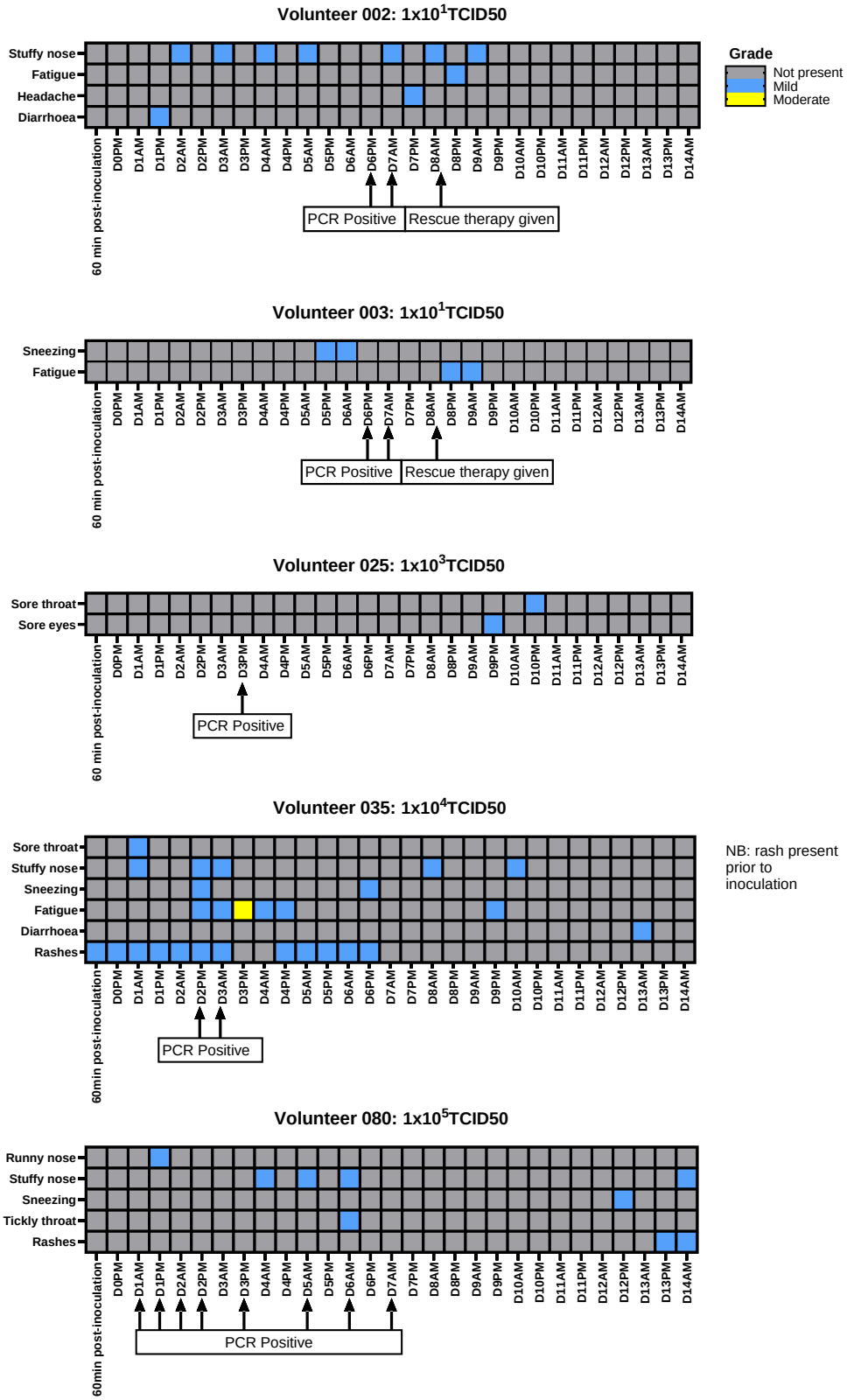

Supplementary Figure 3: Ex-vivo SARS-CoV-2 peptide-specific PBMC IFN- *γ* ELISpot responses after SARS-CoV-2 challenge by dose escalation group

Ex-vivo SARS CoV-2 peptide-specific PBMC IFN-γ ELISpot responses of volunteers in dose escalation groups inoculated with wildtype SARS-CoV-2/human/GBR/484861/2020 virus at a dose of 1 x 10^1 to 1 x 10^5 TCID50 using SARS CoV-2 peptide pools of structural or non-structural proteins. Dots represent individuals, open circles represent volunteers who were unvaccinated at baseline, red dots and lines show volunteers who were transiently infected in quarantine. Background subtracted antigen-specific responses are presented as Spot Forming Cells (SFC)/1x10^6 PBMC. S1: Spike protein subunit 1; S2: Spike protein subunit 2; M: Membrane protein; NP: Nucleocapsid protein; CD4: SARS -COV-2 CD4+ peptide pool; CD8: SARS -COV-2 CD8+ peptide pool; ORF: Open Reading Frame. Post-inoculation samples up to Day 28 were compared to baseline (Day-2) using Wilcoxon matched pairs signed rank. Significance seen for 1x10^4 TCID50 between baseline and Day 2 in S1, S2, M, NP, CD4, CD8 and ORF3, p= 0·016 for all values.

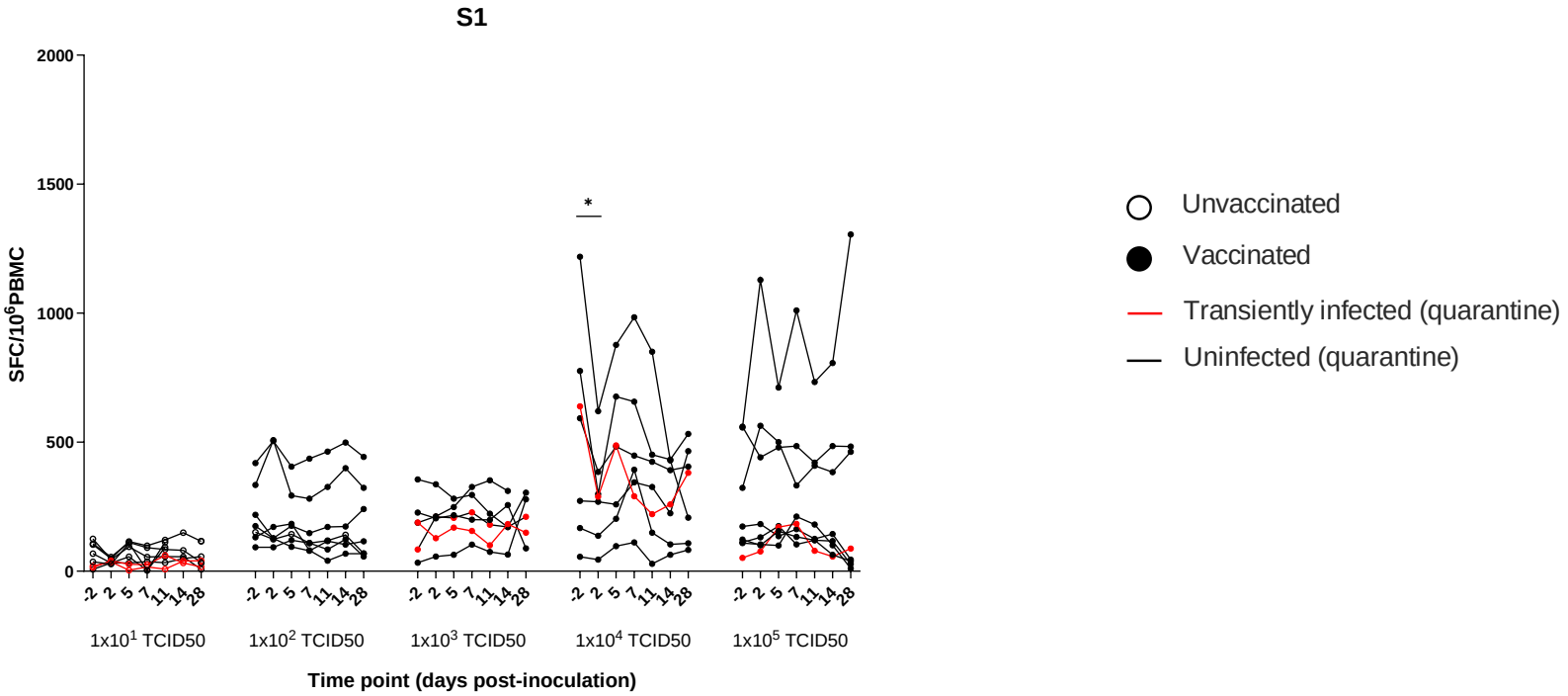

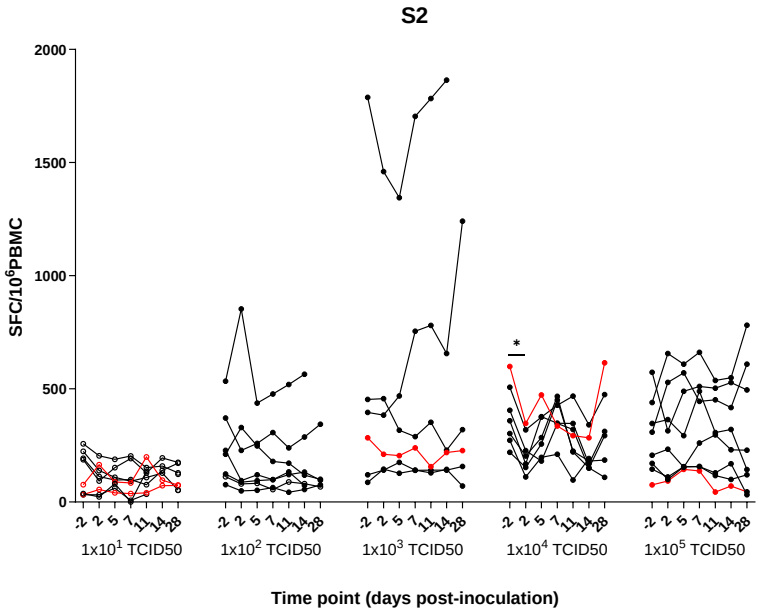
S2
SFC/10^6PBMC
2000
1500
1000
500
0
*
-2 2 5 7 11 14 28
1x10^1 TCID50
1x10^2 TCID50
1x10^3 TCID50
1x10^4 TCID50
1x10^5 TCID50
Time point (days post-inoculation)

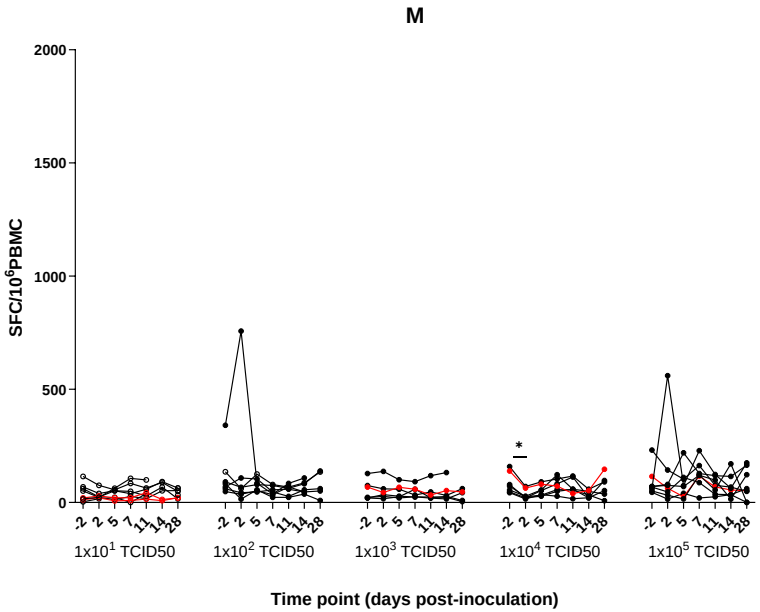
M
SFC/10^6PBMC
2000
1500
1000
500
0
*
-2 2 5 7 11 14 28
1x10^1 TCID50
1x10^2 TCID50
1x10^3 TCID50
1x10^4 TCID50
1x10^5 TCID50
Time point (days post-inoculation)

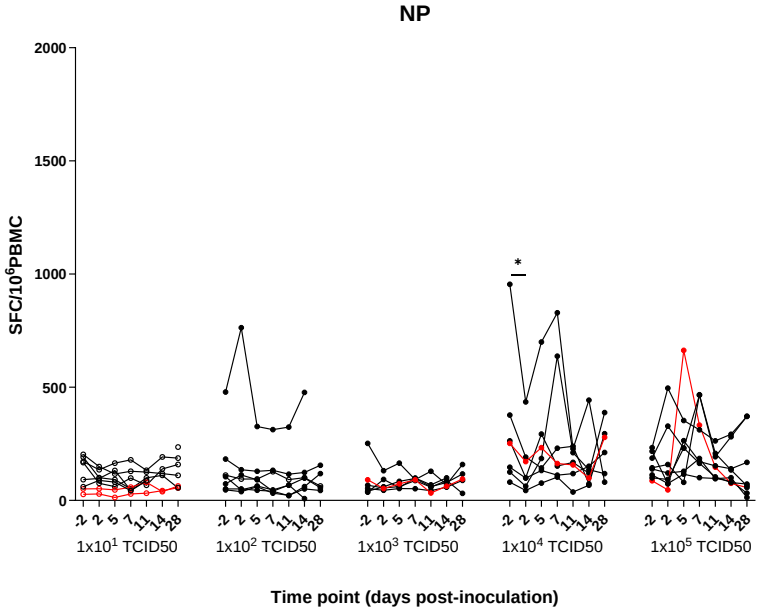
NP
SFC/10^6PBMC
2000
1500
1000
500
0
*
-2 2 5 7 11 14 28
1x10^1 TCID50
1x10^2 TCID50
1x10^3 TCID50
1x10^4 TCID50
1x10^5 TCID50
Time point (days post-inoculation)

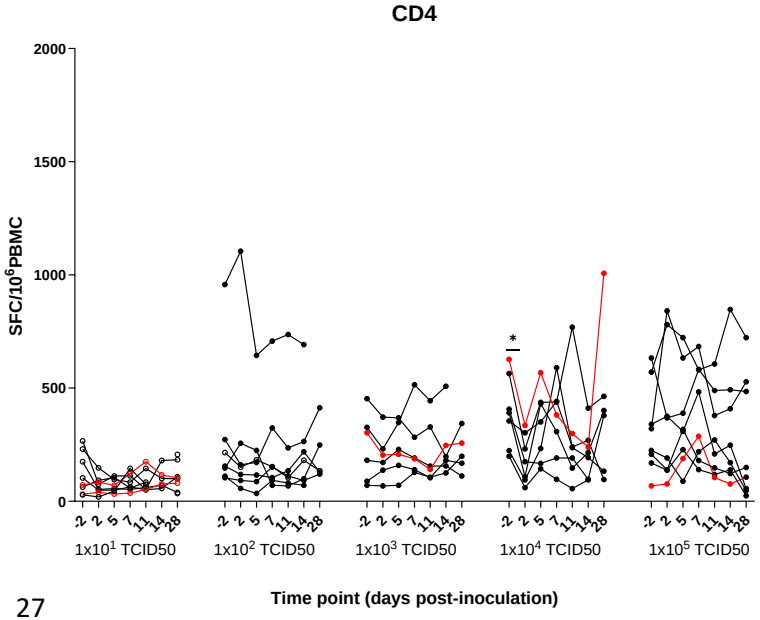
CD4
SFC/10^6PBMC
2000
1500
1000
500
0
*
-2 2 5 7 11 14 28
1x10^1 TCID50
1x10^2 TCID50
1x10^3 TCID50
1x10^4 TCID50
1x10^5 TCID50
Time point (days post-inoculation)

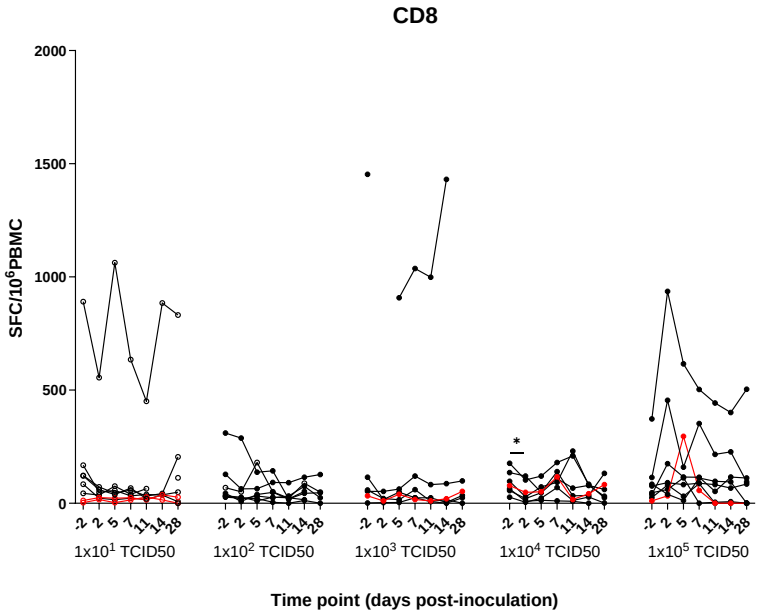
CD8
SFC/10^6PBMC
2000
1500
1000
500
0
*
-2 2 5 7 11 14 28
1x10^1 TCID50
1x10^2 TCID50
1x10^3 TCID50
1x10^4 TCID50
1x10^5 TCID50
Time point (days post-inoculation)

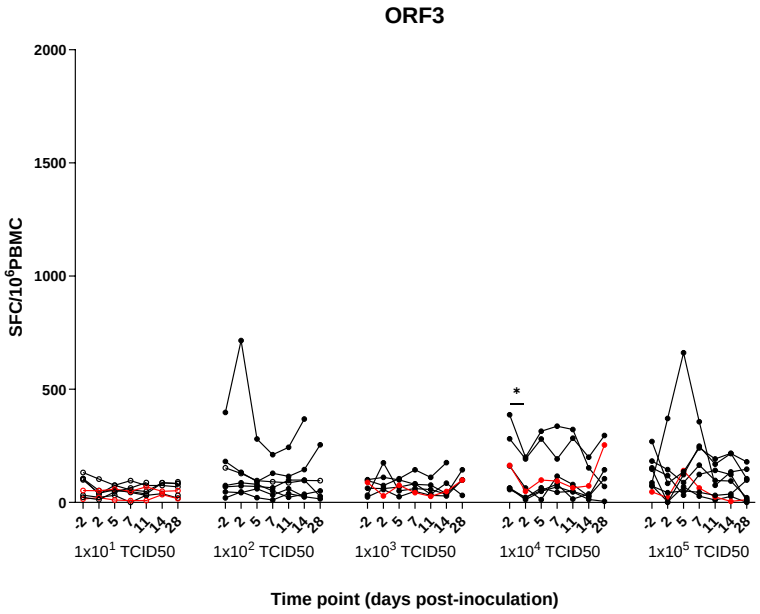
ORF3
SFC/10^6PBMC
2000
1500
1000
500
0
*
-2 2 5 7 11 14 28
1x10^1 TCID50
1x10^2 TCID50
1x10^3 TCID50
1x10^4 TCID50
1x10^5 TCID50
Time point (days post-inoculation)

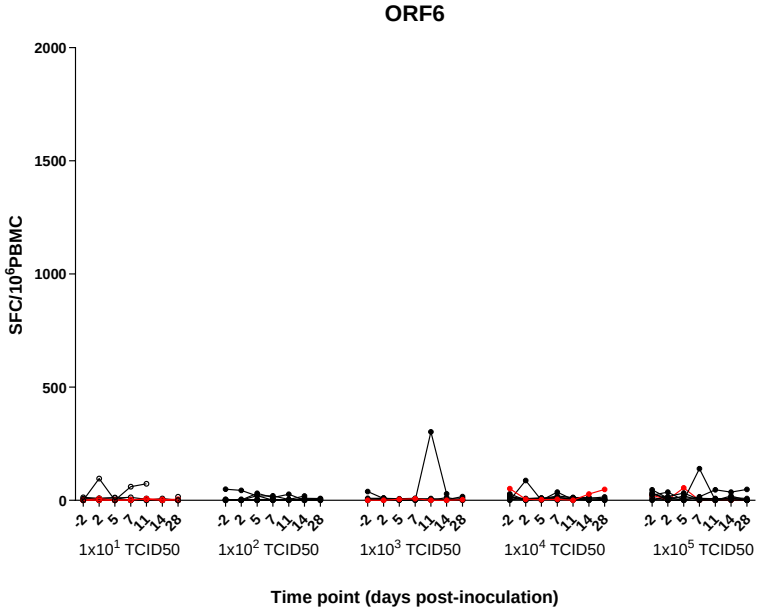
ORF6
SFC/10^6PBMC
2000
1500
1000
500
0
-2 2 5 7 11 14 28
1x10^1 TCID50
1x10^2 TCID50
1x10^3 TCID50
1x10^4 TCID50
1x10^5 TCID50
Time point (days post-inoculation)

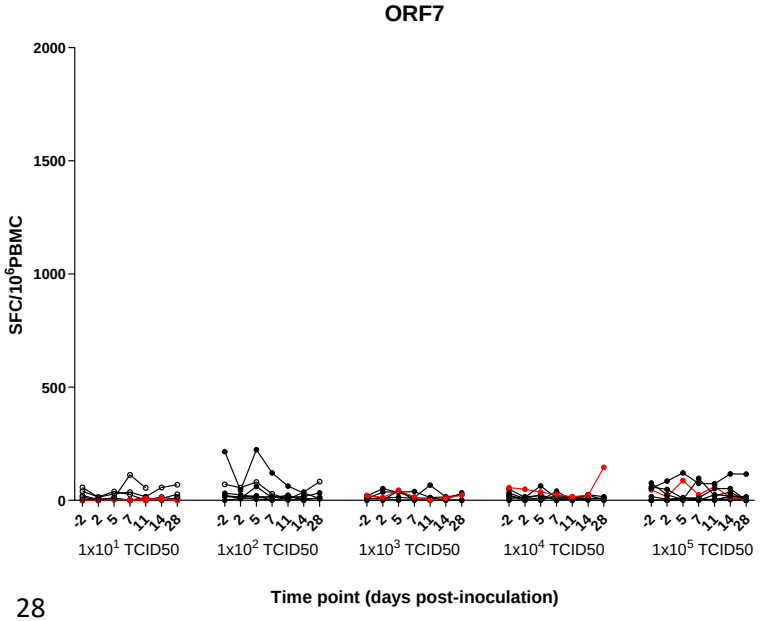
ORF7
SFC/10^6PBMC
2000
1500
1000
500
0
-2 2 5 7 11 14 28
1x10^1 TCID50
1x10^2 TCID50
1x10^3 TCID50
1x10^4 TCID50
1x10^5 TCID50
Time point (days post-inoculation)

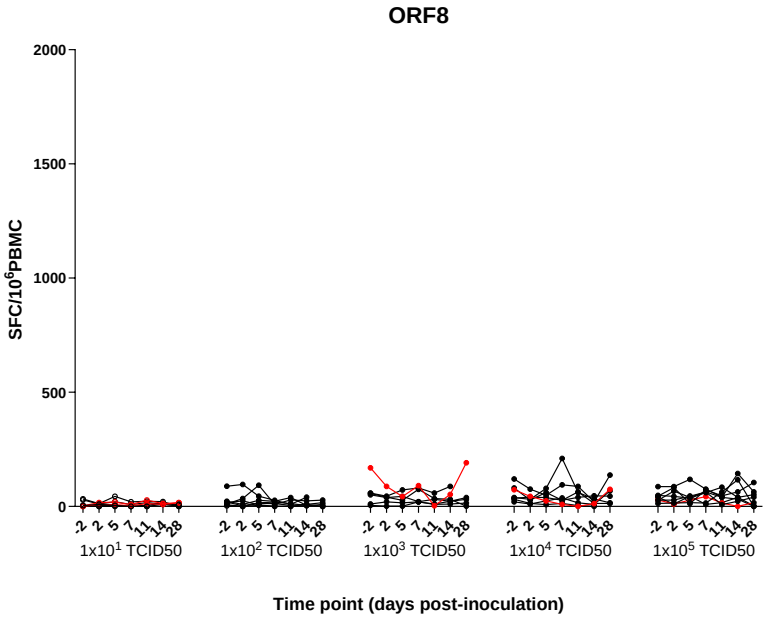
ORF8
SFC/10^6PBMC
2000
1500
1000
500
0
-2 2 5 7 11 14 28
1x10^1 TCID50
1x10^2 TCID50
1x10^3 TCID50
1x10^4 TCID50
1x10^5 TCID50
Time point (days post-inoculation)

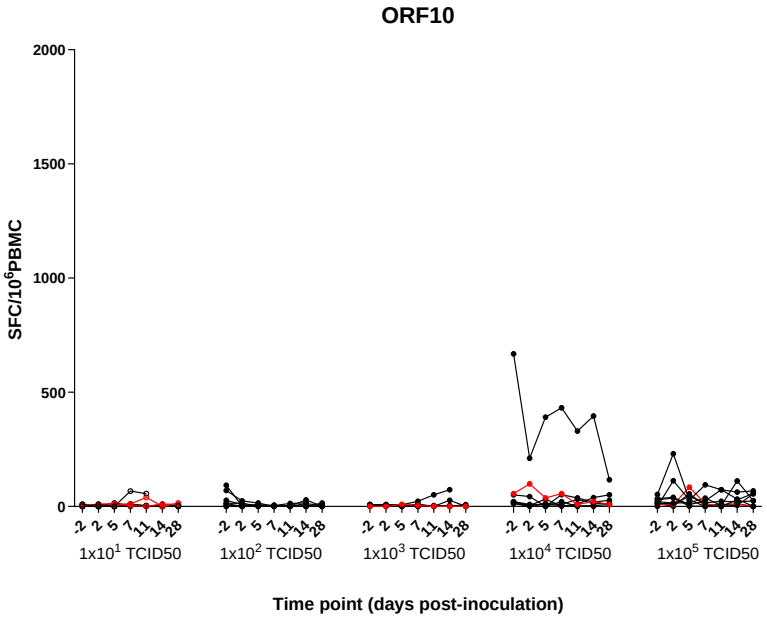
ORF10
SFC/10^6PBMC
2000
1500
1000
500
0
-2 2 5 7 11 14 28
1x10^1 TCID50
1x10^2 TCID50
1x10^3 TCID50
1x10^4 TCID50
1x10^5 TCID50
Time point (days post-inoculation)

Supplementary Figure 4: Baseline serum antibody inhibition of SARS-CoV-2

A. *Surrogate neutralisation assay measuring the ability of baseline serum to inhibit angiotensin-converting enzyme 2 (ACE2) binding to different variants of SARS-CoV-2 spike.* ***B****. Microneutralisation assay using serum and different variants of SARS-CoV-2 virus. Sera from volunteers who subsequently received either 1x10^{1} or 1x10^{2} TCID50 was not tested against Omicron infection given it was not in circulation at the time of serum collection.* ***A+B****. Shapes represent individuals, colour coded for infection dose, black line median with interquartile range, red line WHO positive cut off for Victoria strain (****A****). No significance on Mann-Whitney comparing transiently infected volunteers (TI) with no transient infection (U). AU: Arbitrary units. IC50: half-maximal inhibitory concentration.*

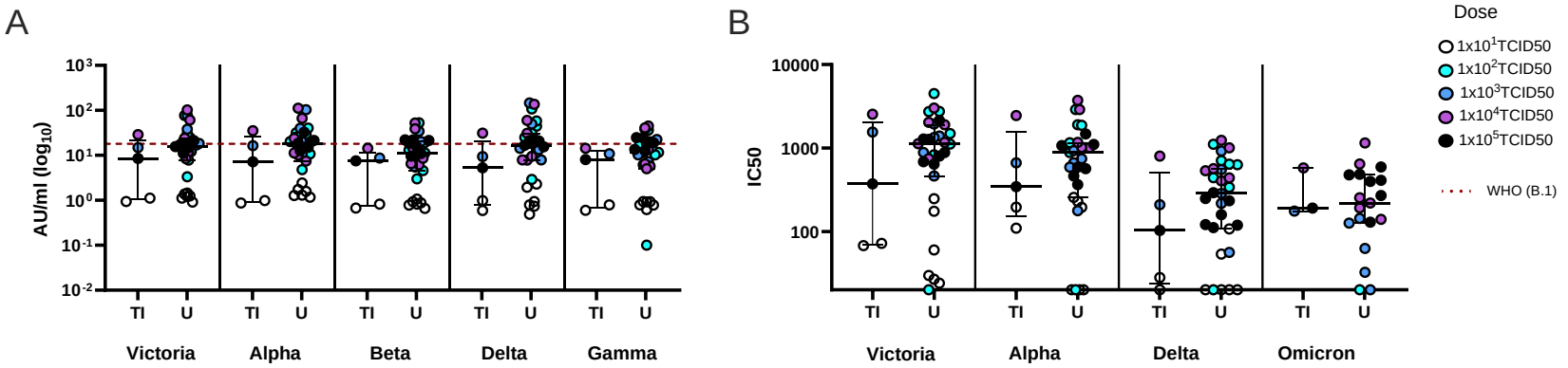

References

1. Killingley B, Mann AJ, Kalinova M, Boyers A, Goonawardane N, Zhou J, et al. Safety, tolerability and viral kinetics during SARS-CoV-2 human challenge in young adults. Nature Medicine. 2022;28(5):1031-41.
2. Tyson JR, James P, Stoddart D, Sparks N, Wickenhagen A, Hall G, et al. Improvements to the ARTIC multiplex PCR method for SARS-CoV-2 genome sequencing using nanopore. bioRxiv. 2020:2020.09.04.283077.
3. Josh Quick 2020. nCoV-2019 sequencing protocol v3 (LoCost). protocols.io. https://protocols.io/view/ncov-2019-sequencing-protocol-v3-locost-bh42j8ye]
4. Baker DJ, Aydin A, Le-Viet T, Kay GL, Rudder S, de Oliveira Martins L, et al. CoronaHiT: high-throughput sequencing of SARS-CoV-2 genomes. Genome Medicine. 2021;13(1):21.
5. https://github.com/connor-lab/ncov2019-artic-nf
6. Rambaut A, Holmes EC, Hill V, O'Toole Á, McCrone JT, Ruis C, et al. A dynamic nomenclature proposal for SARS-CoV-2 to assist genomic epidemiology. bioRxiv. 2020:2020.04.17.046086.
7. https://pangolin.docs.cog-uk.io/ [
8. Thwaites RS, Jarvis HC, Singh N, Jha A, Pritchard A, Fan H, et al. Absorption of Nasal and Bronchial Fluids: Precision Sampling of the Human Respiratory Mucosa and Laboratory Processing of Samples. J Vis Exp. 2018(131).
9. Huang KA, Tan TK, Chen TH, Huang CG, Harvey R, Hussain S, et al. Breadth and function of antibody response to acute SARS-CoV-2 infection in humans. PLoS Pathog. 2021;17(2):e1009352.
10. Wright DW, Harvey WT, Hughes J, Cox M, Peacock TP, Colquhoun R, et al. Tracking SARS-CoV-2 mutations and variants through the COG-UK-Mutation Explorer. Virus Evol. 2022;8(1):veac023.

CLINICAL STUDY PROTOCOL

A dose finding human experimental infection study with SARS-CoV-2 in healthy volunteers immunologically sensitised with either previous SARS-CoV-2 infection and/or vaccination against SARS-CoV-2.

Short title: COV-CHIM01: SARS-CoV-2 dose finding infection study

**Study Reference:**	COV-CHIM01
**Protocol Version:**	12.1
**Date:**	23 Mar 2023
**REC Reference:**	21/UK/0001
**IRAS Reference:**	296569
**Chief Investigator:**	Professor Helen McShane
**Sponsor:**	University of Oxford
**Funder:**	Wellcome Trust & Department of Health and Social Care (DHSC)

Authors: Dr Ingrid Cabrera Puig, Dr Susan Jackson, Dr Julia Marshall, Raquel Lopez Ramon, Dr Hazel Morrison, Dr Andrew Mawer, Hannah Scott, Dr Timothy Fredsgaard-Jones, Dr Meng-San Wu

Key Trial Contacts:

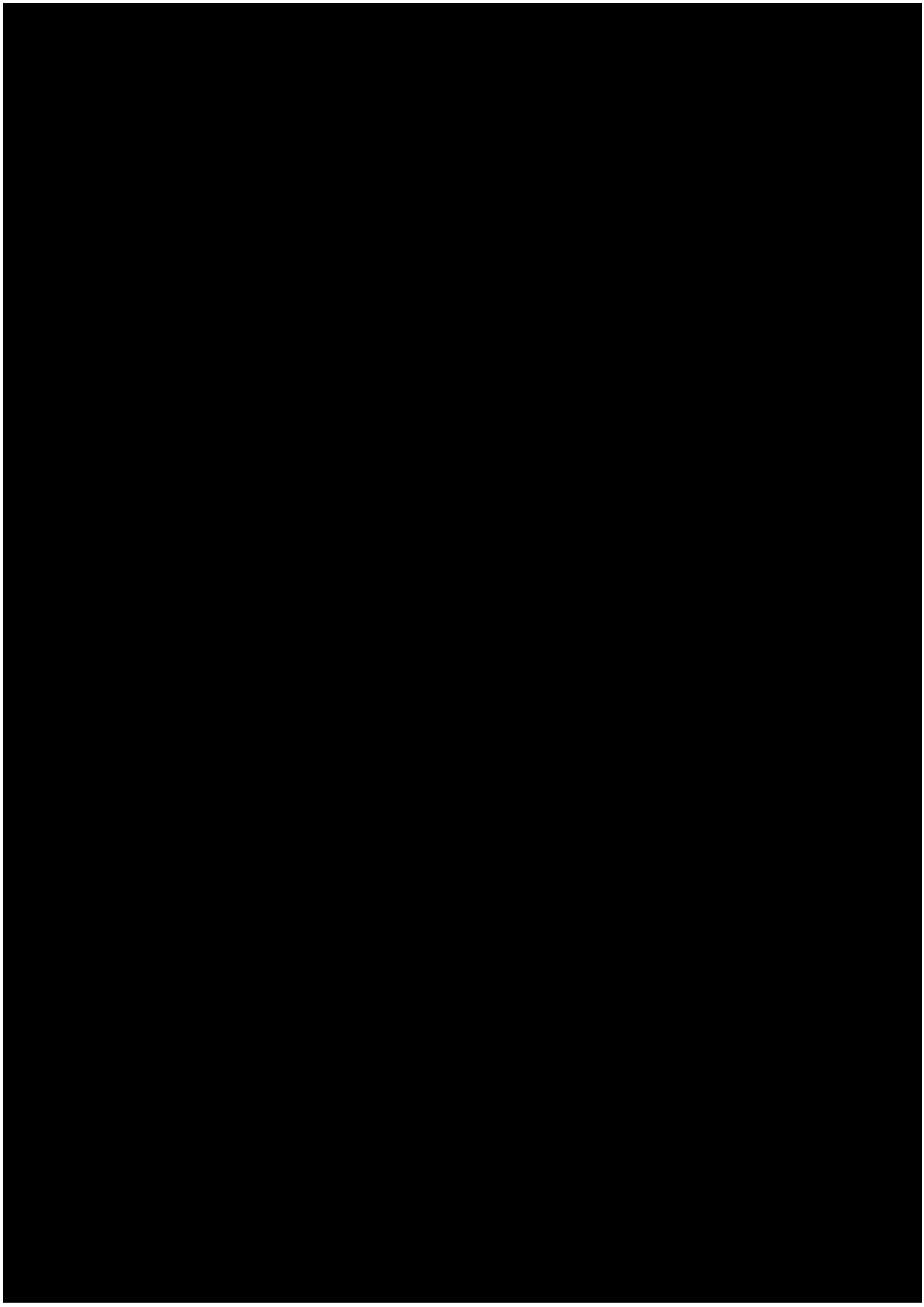

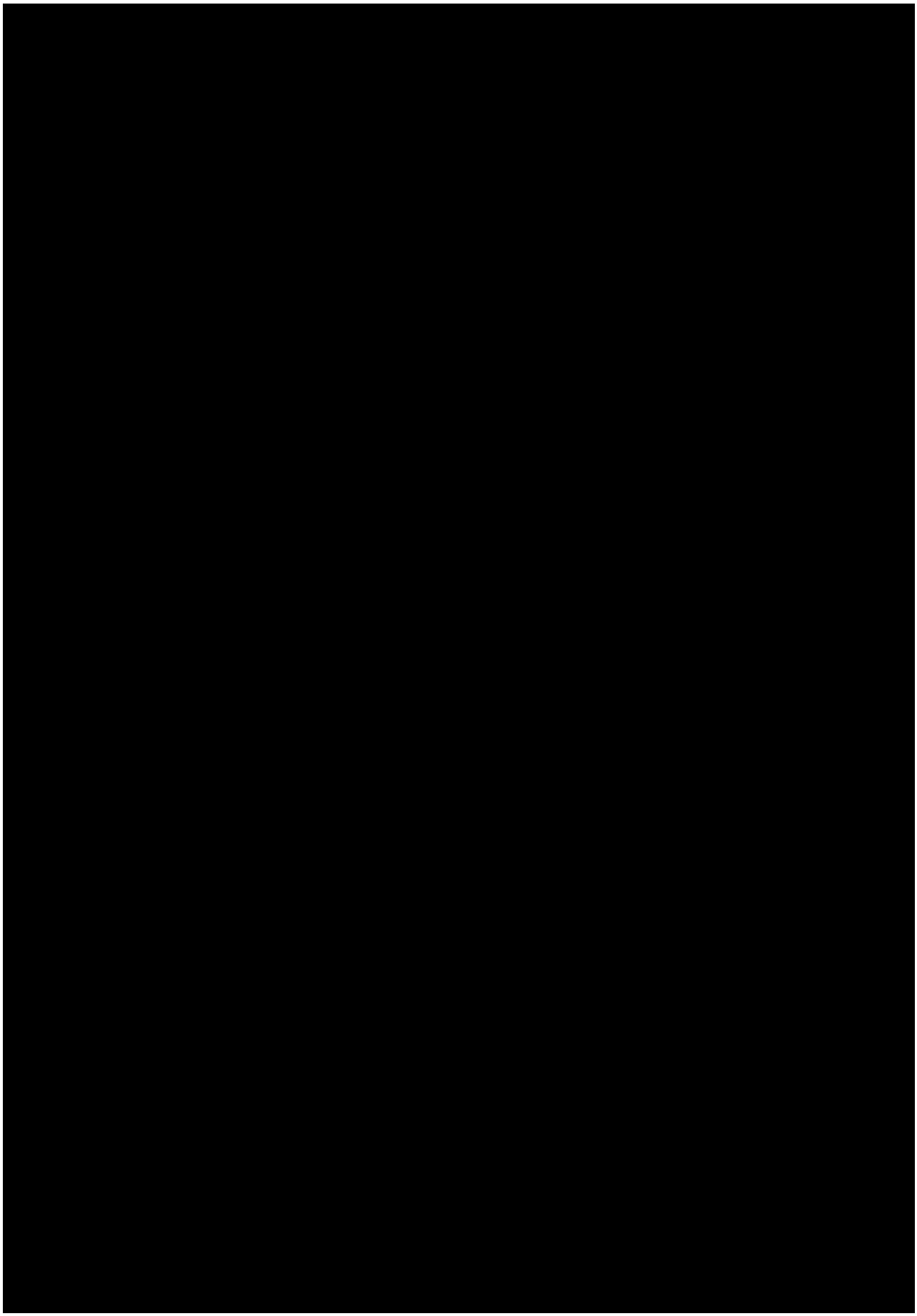

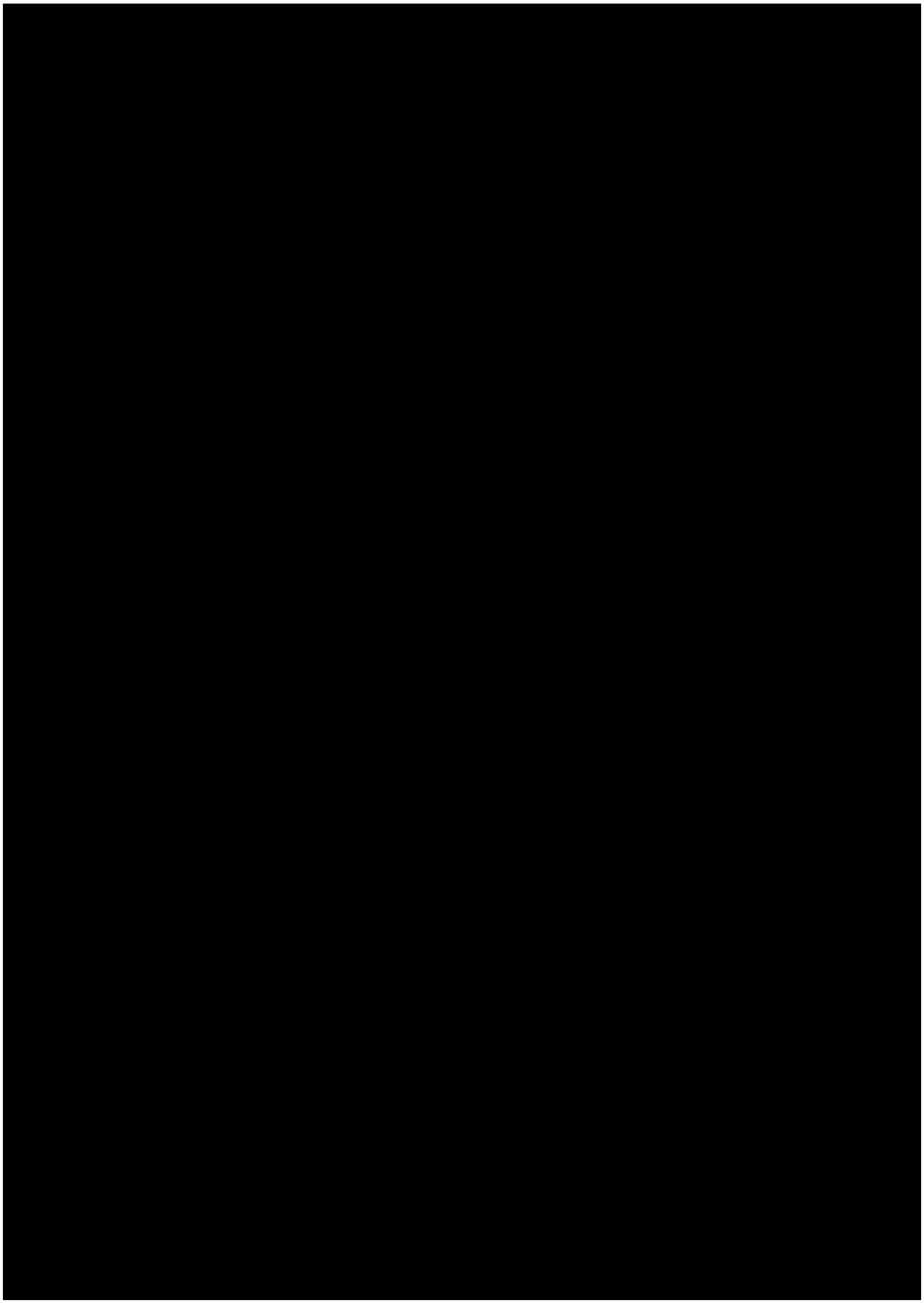

[redacted]

Confidentiality Statement

This document contains confidential information that must not be disclosed to anyone other than the Sponsor, the investigator team, HRA, host organisation, and members of the Research Ethics Committee and other regulatory bodies. This information cannot be used for any purpose other than the evaluation or conduct of the clinical investigation without the prior written consent of Professor Helen McShane.

Statement of Compliance

The study will be conducted in compliance with the protocol, the principles of Good Clinical Practice, Medicines for Human Use (Clinical Trial) Regulations 2004 (as amended) and all other applicable regulatory requirements.

Chief Investigator approval and agreement

"I have read this protocol and agree to abide by all provisions set forth therein. I agree to comply with the International Conference on Harmonisation Tripartite Guideline on Good Clinical Practice."

Professor Helen McShane

[redacted]

"According to the Declaration of Helsinki, 2008, I have read and hereby approve this version of the protocol. I declare no conflict of interest"

Conflict of Interest Details:

_____

Professor Helen McShane

[redacted]

Table of Contents

1 SYNOPSIS....56

SUMMARY....60

2 ABBREVIATIONS....61

3 BACKGROUND AND RATIONALE....63

3.1 Background....63

3.2 Why is the COVID-19 challenge model so important?....64

3.3 How is this research relevant to patients?....65

3.4 Immune response to SARS-CoV-2....65

3.5 Rationale for addition of groups 3 & 4 (previously uninfected, vaccinated participants)....67

3.6 Research Strategy....68

3.7 Virology of SARS-CoV-2....69

3.8 Virological Correlates of Infection....72

3.9 Re-infection....74

3.10 Harmful Immune responses....74

3.11 Risks and Clinical outcomes of SARS-CoV-2 infection....75

3.12 Treatment of Severe COVID-19....82

3.13 Pre-clinical and clinical experience with REGN-COV2 (Regeneron Monoclonal Antibody cocktail Or Ronapreve) and Paxlovid for COVID-19:....82

3.14 Potential long-term complications of COVID-19....86

3.15 Mitigating Risk in Experimental Human SARS-CoV-2 Infection....90

3.16 Our group is well placed to perform human respiratory pathogen challenge studies....91

3.17 Hypothesis....92

4 OBJECTIVES AND ENDPOINTS....92

5 COMMITTEES....94

5.1 Trial Steering Committee (also known as Medical Oversight Committee) 94

5.2 Data Safety Monitoring Board 94

6 STUDY DESIGN 95

6.1 Study groups 96

6.2 Study volunteers 98

6.3 Definition of Start and End of Study 98

6.4 Potential Risks for volunteers 98

6.5 Known Potential Benefits 107

7 RECRUITMENT AND WITHDRAWAL OF STUDY VOLUNTEERS 107

7.1 Identification of Study Volunteers 107

7.2 Informed consent 108

7.3 Inclusion and exclusion criteria 110

7.4 Prevention of 'Over Volunteering' 115

7.5 Withdrawal of Volunteers 115

7.6 Compliance with Dosing Regime 117

7.7 Pregnancy 117

8. CLINICAL PROCEDURES 117

8.1 Schedule of Attendance 117

8.2 Observations 117

8.3 Study Procedures 117

8.4 Treatments administered 124

8.5 Diary card 128

8.6 Study visits 129

8.7 Sequence of Enrolment and challenge of volunteers 134

8.8 Blinding 134

8.9 Dose escalation, de-escalation and confirmation 135

8.10 Rescue treatment with REGN COV2 (Ronapreve) or Paxlovid 140

8.11 Discharge from confinement 141

8.12 Subsequent follow up visits: Day 28, 56, 84, 168 and 365 141

8.13 Unscheduled additional visits for individuals with possible or confirmed COVID-19 in post quarantine follow up period 142

8.14 Schedule of Attendances 144

9. STUDY AGENTS AND DEVICES 150

9.1 SARS-CoV-2 virus 150

9.2 REGN COV2 and Paxlovid 151

9.3 Dalteparin 152

9.4 Duckbill face mask 152

10. ASSESSMENT OF SAFETY 152

10.1 Definitions 153

10.2 Foreseeable adverse reactions 154

10.3 Unforeseen Serious Adverse Reactions 154

10.4 Causality 155

10.5 Assessment of severity 156

10.6 Reporting Procedures for Serious AEs 157

10.7 Reporting of events related to REGN-COV2 157

10.8 Procedures to be followed in the event of abnormal findings 158

10.9 Interim Safety Reviews 158

10.10 Safety Group Holding Rules 158

11 STATISTICS 160

12 DATA MANAGEMENT 160

12.1 Data Handling 160

12.2 Record Keeping 161

12.3 Source Data and Case Report Forms (CRFs) 161

12.4 Data Protection 161

12.5 Data Quality 162

13 QUALITY CONTROL AND QUALITY ASSURANCE PROCEDURES 162

13.1 Investigator procedures 162

13.2 Monitoring 162

13.3 Protocol deviation 162

13.4 Audit & inspection 162

14 SERIOUS BREACHES 162

15 ETHICS AND REGULATORY CONSIDERATIONS 163

15.1 Declaration of Helsinki 163

15.2 Guidelines for Good Clinical Practice 163

15.3 Ethical Approvals 163

15.4 Volunteer Confidentiality 163

16 FINANCING AND INSURANCE 164

16.1 Financing 164

16.2 Insurance 164

16.3 Contractual Arrangements 164

16.4 Compensation 164

17 PUBLICATION POLICY 164

AMENDMENT HISTORY

Protocol v12.1 Non-Substantial Amendment 4

Section	From	Changed To	Rationale for change
1. Synopsis	34 months (06 May 2021 – 06 March 2024)	38 months (06 May 2021 – 06 July 2024)	Due to reduced enrolment in the last cohorts, the duration has been extended to enable additional time to meet enrolment targets.

8.1 Schedule of Attendance<br>8.14 Schedule of Attendances<br>Table 7. Risks associated with other Study procedures	A total volume of blood collected over 12 months is 865.5-870.5ml.	A total volume of blood collected over 12 months is 838.5ml.	Reduction in the maximum blood volume taken due to a change in tubes used by the biochemistry laboratory. A single 5ml tube may now be used in lieu of 1 x 5ml and 1 x 3ml tube.<br>The schedule has also been updated to align presentation of the maximum exploratory immunology volume during the quarantine period in both cases.

Protocol v12.0 Substantial Amendment 9

Section	From	Changed To	Rationale for change
Senior immunologists	Public Health England	UK Health Security Agency	Revision missed in pervious amendment to capture name change of Public Health England
1. Synopsis	Planned Study Duration 30 Months	Planned Study Duration 34 months (06 May 2021 to 06 March 2024)	Estimate for study duration revised based on rate of enrolment in study groups to date.<br>Dates added for clarity.
1. Synopsis<br>2. Abbreviations	-	Typographical errors corrected	Correction of typographical errors throughout.

3.7 Virology of SARS-CoV-2<br><br>3.15 Mitigating risk in experimental human SARS-Cov2 infection<br>6.4 Potential risks for volunteers			Sub section numbering correction in 3.7.
1. Synopsis6.2 Study volunteers<br>6.4.3 Cardiovascular Magnetic Resonance (CMR) imaging and Gadolinium contrast use<br>6.5 Known Potential Benefits<br>7.3 Inclusion and exclusion criteria<br>8.3.10 Cardiovascular Magnetic Resonance (CMR)<br>8.3.11 Echocardiography<br>8.6 Study visits<br>8.11 Discharge from confinement<br>8.14 Schedule of Attendances<br>16.4 Compensation	Cardiac MRI (CMR) or echocardiogram (echo) at screening and discharge (for infected volunteer only)	Removal of CMR/echo	To date, CMR/echo has neither demonstrated any clinically significant changes for any participants, nor has an abnormal CMR/echo result at screening excluded any volunteer. However, it imposes a significant burden for the volunteers, which is not justified by its very marginal benefits. In agreement with the DSMB, CMR and echo will therefore be removed for all participants, especially considering robust and intensive cardiac monitoring by serial troponins and ECGs already in place.<br><br>The minimum compensation has been reduced to £4845 in line with this change.
Summary	As of 3rd March 2022, there have been over 4 million confirmed COVID-19 cases and over 120,000 deaths in the United Kingdom	As of 9th November 2022, there have been over 20 million confirmed COVID-19 cases and over 170,000 deaths in the United Kingdom	Updated data included
Summary<br>7.2 Informed consent	a) individuals with microbiologically proven	a) individuals with previous SARS-CoV-2 infection (+/- vaccination)	Removal of microbiologically proven in line with SA008

	previous SARS-CoV-2 infection (+/- vaccination)		
7.3.2 Exclusion criteria	Volunteers with any history of physician diagnosed and/or objective test confirmed asthma, chronic obstructive pulmonary disease, pulmonary hypertension, reactive airway disease, or chronic lung condition of any aetiology or who have experienced:<br><br>i) Significant/severe wheeze in the past<br>ii) Respiratory symptoms, including wheeze, which has ever resulted in hospitalisation<br>iii) Known bronchial hyper reactivity to viruses	Volunteers with any history of physician diagnosed and/or objective test confirmed asthma, chronic obstructive pulmonary disease, pulmonary hypertension, reactive airway disease, or chronic lung condition of any aetiology or who have experienced:<br><br>iv) Significant/severe wheeze in the past<br>v) *Clinically significant* respiratory symptoms, including wheeze, which has ever resulted in hospitalisation<br>vi) Known bronchial hyper reactivity to viruses	Addition of *clinically significant* to respiratory symptoms to allow for recruitment of volunteers who have been hospitalised for respiratory symptoms which would not put them at increased risk of SARS-CoV-2 inoculation. E.g. a participant admitted overnight to await a computed tomography pulmonary angiogram (CTPA) which was subsequently normal.
8.3.8 Pulmonary Function Tests (PFTs)<br><br>8.14 Schedule of Attendances	Spirometry and Transfer Factor repeated at D28 visit	Removal of routine D28 PFTs, replacement with PFTS in participants demonstrating PCR positivity beyond D1 or at clinician discretion	Pulmonary function testing is of limited value in participants who are not successfully infected in quarantine. All safety data thus far, including CT scans, AE reporting and PFTs have been reassuring and suggest that intra-nasal inoculation with SARS-CoV-2 is not associated with significant lower respiratory tract involvement. However, we have yet to see sustained infection in any participant. Therefore, repeat PFTs at D28 will be

			restricted to participants who demonstrate PCR positivity beyond D1 and may be performed in other participants at clinician discretion.
8.3.9 Radiology<br>8.8 Blinding<br>8.14 Schedule of Attendances	*Capacity for CT scanning will be pre-arranged for D5 and D11 with the local radiology department. However, if a participant meets the criteria outlined above and there is capacity to carry out a CT scan prior to D5 or D11 a CT scan may be performed at an earlier timepoint.	*Capacity for CT scanning will be pre-arranged for D5 and D11 with the local radiology department. However, if a participant meets the criteria outlined above and service capacity permits, a CT scan may be performed at another timepoint during the quarantine stay.<br><br>Following DSMB review on 31st October 2022 routine CT scans were discontinued for remaining participants in groups 2, 3 & 4 and instead restricted to participants with symptoms or signs of lower respiratory tract involvement as above	Clarification: in the unlikely event that a participant were to meet criteria for a CT scan beyond D11 this could be carried out if radiology capacity allows.<br>Confirmation of DSMB outcome added and associated revision to blinding wording and schedule of attendance table.
8.6.2 Consent & Screening visit	The volunteer will be required to isolate	The volunteer will be advised to isolate	Revised in line with changes approved as part of SA07

Protocol v11.0 Substantial Amendment 8

Section	From	Changed To	Rationale for change
Title<br>1. Synopsis	A dose finding human experimental infection study with SARS-CoV-2 in healthy volunteers immunologically sensitised with either previous,	A dose finding human experimental infection study with SARS-CoV-2 in healthy volunteers immunologically sensitised with either previous SARS-CoV-2	Updated to reflect inclusion of serologically confirmed SARS-CoV-2 infection.

	microbiologically confirmed, SARS-CoV-2 infection and/or vaccination against SARS-CoV-2.	infection and/or vaccination against SARS-CoV-2.	
1. Synopsis<br>6.2 Study volunteers<br>7.3.1 Inclusion Criteria<br>8.6.2 Consent & Screening Visit	Groups 1 & 2: Previous microbiological confirmation of SARS-CoV-2 infection > 3 months prior to enrolment (Proof of positive PCR or lateral flow antigen test confirmed via medical notes/ or PHE)	Groups 1 & 2: Previous microbiological confirmation of SARS-CoV-2 infection > 3 months prior to enrolment (Proof of positive PCR or lateral flow antigen test confirmed via medical notes/ or UK HSA or a history from a volunteer consistent with SARS-CoV-2 infection with other evidence of this infection such as a photograph of a positive lateral flow test on the volunteer's phone or similar). OR serological confirmation such as positive anti-nucleocapsid IgG serology (unless this is explainable by prior vaccination) with the most recent history of symptoms or exposure likely to represent SARS-CoV-2 infection having occurred > 3 months prior to enrolment*.<br><br>*Where no history of symptoms or exposure can be identified to determine the timing of SARS-CoV-2 infection, volunteers may be enrolled >7 weeks from the identification of anti-nucleocapsid positivity and >3 months from their	As the government no longer offers confirmatory PCR and given the 24 hour time limit on logging positive lateral flows on https://www.gov.uk/report we have found that many volunteers no longer reach this burden of proof depsite having a convincing history of SARS-CoV-2 infection and alternative proof of this infection, such as a photograph of a positive lateral flow test or similar. This change will facilitate the recruitment of these volunteers.<br><br>The shorter acceptable time interval between anti-nucleocapsid postivity and enrolment in asymptomatic volunteers reflects the finding that anti-nucleopsid antibodies usually remain positive for several months after infection [1]and a positive result is unlikely to represent the actual time of infection. Furthermore, Ig G antibodies peak 3-7 weeks post symptom onset, with IgM peaking at 2-5 weeks and neutralising antibodies peaking and declining at 14-22 days. [2] Accepting the limitiations of these assays, 7 weeks from anti-N positivity likely places participants beyond

		last negative anti-nucleocapsid antibody test	the peak of their immune response to their community acquired infection.
3.15 Mitigating Risk in Experimental Human SARS-CoV-2 Infection	iv. Participants in the dose escalation phase will be treated with either a targeted combined monoclonal therapy (REGN-COV2) or oral antiviral (paxlovid), depending on timing of enrolment in the study, if they develop any warning symptoms or signs of COVID-19 disease (see section 8.9). The need to continue this for our dose confirmation phase (Group 2) will be assessed after review by the DSMB. Full details regarding the two phases of the study can be found in section 6.1.	iv. Participants in the dose escalation phase will be treated with either a targeted combined monoclonal therapy (REGN-COV2) or oral antiviral (paxlovid), depending on timing of enrolment in the study, if they develop any warning symptoms or signs of COVID-19 disease (see section 8.9). The need to continue this for our dose confirmation phase (Group 2 and Group 4) will be assessed after review by the DSMB. Full details regarding the two phases of the study can be found in section 6.1.	Group 4 listed along with group 2 as part of the dose confirmation stage of the study.
5.2 Data Safety Monitoring Board	After the dose finding Group 1 is complete and prior to Group 2 enrolment to determine the dose for Group 2 and the use of the following<br>• Rescue therapy<br>• CT scans at D5 and 11 post infection	After the dose finding Group 1 is complete and prior to Group 2 enrolment to determine the dose for Group 2 and the use of the following in groups 2, 3, and 4 as appropriate as detailed in this protocol:<br>• Rescue therapy<br>• CMR scans<br>• Routine use of CT scans at D5 and 11 post infection	Rescue therapy, CMR scans and routine use of CT scans at D5 and D11 will be reviewed with the DSMB at the end of both dose confirmation groups (1 and 3). If the DSMB recommends changes to the use of rescue therapy and CMR scans a substantial amednment will be sought. SA08 provides provision within the protocol to cease routine use of CT scans and

			replace this with CT scans for participants with confirmed COVID and symptoms and/or signs of lower respiratory tract involvement as outlined in section 8.3.9 with DSMB approval. Any other changes to the use of CT scans would require a further substantial amendment
	After the dose finding Group 3 is complete and prior to Group 4 enrolment to determine the dose for Group 4 and the use of the following<br>• Rescue therapy<br>• CT scans at D5 and 11 post infection	After the dose finding Group 3 is complete and prior to Group 4 enrolment to determine the dose for Group 4 and the use of the following, if not already confirmed following review of Group 1:<br>• Rescue therapy<br>• CMR scans<br>• Routine use of CT scans at D5 and 11 post infection	Updated to clarify that the DSMB may review the use of rescue therapy, CMR scans and routine use of CT scans in groups 3 and 4 at completion of group 1 and group 3.
7.1 Identification of study volunteers	Addition	Text messaging services to potentially eligible participants when agreed with the owner of the database (i.e. local GP practices)	Additional advertising method to further enhance recruitment
7.3.2 Exclusion criteria	Clinically significant smoking history. Defined as: Current smoker (any smoking including e-cigarettes in the last 3 months) or > 2 pack year smoking history at any time (2 pack years is equivalent to 20 cigarettes daily for 2 years), or use of any nicotine containing products within the last 3 months	Clinically significant smoking history. Defined as: Current smoker (any smoking including e-cigarettes in the last 3 months) or > 2 pack year smoking history at any time (2 pack years is equivalent to 20 cigarettes daily for 2 years), or use of any nicotine containing products *on more than one occasion* within the last 3 months	A single isolated use of a nicotine containing product (e.g. a cigarette at a wedding) in someone who is a non-smoker dose not pose increased risk to volunteers and therefore participants with this kind of history should not be excluded.

	History or evidence of autoimmune disease or known immunodeficiency of any cause (including HIV).	History or evidence of clinically significant autoimmune disease or known immunodeficiency of any cause (including HIV).	History or evidence of autoimmune disease encompasses a wide range of symptoms and signs. Where this history or evidence is not concerning for an underlying disease that might place a participant at increased risk or afffect the integrity of the data participants may be enrolled into the study. .
7.3.4 Challenge Postponement criteria	Evidence of recent SARS CoV-2 infection (<3 months) based on a suggestive clinical history or investigations such as repeat serology, lateral flow antigen tests or PCR. Volunteers in group 3 & 4 could be re-enrolled after 3 months if appropriate into group 1 or 2. Volunteers in group 1 or 2 would have to defer participation for 3 months.	Evidence of recent SARS CoV-2 infection (<3 months) based on a suggestive clinical history or investigations such as repeat serology, lateral flow antigen tests or PCR. Volunteers in group 3 & 4 could be re-enrolled, if appropriate, into group 1 or 2 as per the inclusion criteria.	Challenge postponement criteria updated in line with inclusion criteria
8.3.5 Nasosorption (immune assay)	Up to two strips of synthetic absorptive matrix (SAM™) will be used	Up to four strips of synthetic absorptive matrix (SAM™) will be used…	Up to this point the yield from two synthetic absorptive matrix (SAM™) strips has not been sufficient for some cytokine assays, therefore the upper limit has been increased to allow four strips to be taken. This procedure is painless and well tolerated by participants.
8.3.9 Radiology<br>8.14 Schedule of Attendances	Prior to commencement of dose confirmation groups (2 and 4), the DSMB will review the safety data and the need for CT scans in both cohorts. Based on the data from the corresponding dose	After completion of group 1 the DSMB will review the need for routine CT scans in all participants for groups 2, 3 and 4. The DSMB will consider the safety data as a whole including whether routine CT scans	Having dose escalated up to and including 1 x 10^4 TCID50 in previously infected volunteers (group 1) CT scan findings thus far have been reassuring However, CT scans are not without risk given the radiation exposure

	escalation group e.g. group 1 (previously infected volunteers) for Group 2 and Group 3 (uninfected vaccinated volunteers) for group 4 they may advise against continued use of CT scans in the corresponding dose confirmation group. If that is the case we will seek approval from the REC to amend the protocol accordingly	have demonstrated evidence of clinically significant lower respiratory tract involvement and/or altered the management of any participants enrolled in the study. If it is felt that the risks of radiation exposure outweigh the potential benefits of CT scans they will no longer be offered routinely. Instead, a low dose CT chest will be carried out at day 5 and/or day 11* in any participant with COVID-19 infection and any of the following<br><br>• Ongoing severe and persistent cough – Grade 3 reports of coughing via symptom diary cards that is largely persistent over 48 hours (Grade 3: Significant discomfort with marked limitation in activity).<br>• Any event of confirmed hypoxia (≤ 94%, usually confirmed over a 1-hour period)<br>• Ongoing signs of lower respiratory tract involvement on auscultation (e.g. crepitations)<br>• At CI discretion<br><br>*Capacity for CT scanning will be pre-arranged for D5 and D11 with the local radiology department. However, if	associated with their use. Therefore, the study team will review their use with the DSMB at the completion of dose escalation group 1. If emergent data suggests that participants in groups 2, 3 or 4 are likely to derive more harm than benefit from routine use of CT scans these may be discontinued with the approval of the DSMB. We will instead carry out CT scans in participants where clinically indicated as per section 8.3.9

		a participant meets the criteria outlined above and there is capacity to carry out a CT scan prior to D5 or D11 a CT scan may be performed at an earlier timepoint.<br><br>Importantly CI discretion will be used at all times in the decision to carry out a CT chest. If a participant meets the criteria for CT chest but is felt to be unwell enough to require referral for hospital care, CT chest will not be performed within the study in order to avoid delaying admission and/or treatment. Where a participant undergoes a CT chest at D5 this may or may not be repeated at D11 at CI discretion.<br><br>After completion of group 1 the DSMB will advise from the following options:<br><br>• Continue use of routine CT scans throughout the study with the option for re-review after enrolment of further participants<br>• Discontinue use of routine CT scans in all groups (2,3 and 4)<br>• Discontinue use of routine CT scans in group 2 with continued use of routine CT scans in group 3 and re-review of the use of	

		CT scans prior to progression to group 4<br><br>This change will take effect immediately after the DSMB have provided their opinion.	
Throughout document	PHE	References to PHE replaced with UK HSA, or clarified with UK HSA (formerly PHE) where appropriate	The UK Health Security Agency is a government agency that has replaced Public Health England. The protocol has therefore been updated for accuracy of the current agency.<br><br>Where appropriate to past events, references to PHE remain.

Protocol v10.2 Non-Substantial (Minor) Amendment 2

Section	From	Changed To	Rationale for change
Title Page	-	Additional author added	Authorship not recognised in previous amendments
7.1 Identification of Study Volunteers	-	• In public places, including buses and trains, with the agreement of the owner/ proprietor<br>• In literature for circulation<br>• Banner adverts online (e.g. Google advertising)<br>• Postage mailouts<br>• The above methods may also be used	Due to a slowing of recruitment, additional streams of recruitment will be beneficial to the study.<br><br>All methods will utilise previously approved advertising and/or participant facing materials; no new documents are

		within GP practices in England	introduced as part of this amendment.

Protocol v10.1 Minor Amendment 1

Section	From	Changed To	Rationale for change
9.1.3 Dispensing and administration	The inoculum dose will be rapidly defrosted by warming in the gloved hand. Lying down, volunteers will be inoculated with the 200ul inoculum by 4 x 50ul drops into the nostrils (100ul per naris) using filter tips and pipette.	The inoculum dose will be rapidly defrosted by warming in the gloved hand. Lying down, volunteers will be inoculated with a maximum 200ul inoculum, depending on concentration of the vial, by 4 drops into the nostrils (maximum 100ul per naris) using filter tips and pipette.	The vials of virus are slightly above the required 10^5 dose level, therefore a slightly reduced volume (under 200ul) is needed to achieve the maximum dose approved in SA007.<br>This was not known at the time of writing SA007.<br>The exact volume required will be calculated for inclusion in internal SOPs.

Protocol v10.0 Substantial Amendment 7

Section	From	Changed To	Rationale for change
1. Synopsis	Planned Study Duration: 24 months	Planned Study Duration: 30 months	Estimate for study duration revised based on rate of enrolment in study groups to date.
1. Synopsis<br>6.1 Study Groups<br>8.9 Dose escalation, de-escalation and confirmation	Groups 3a and 3b, n=6-8	Groups 3a and 3b, n=4-8	A minimum of 4 individuals will need to be enrolled at each dose with none having been infected at that dose prior to dose escalation. If 1 or more of the first 4 volunteers develops infection, the group will be expanded.<br>We have adjusted our group size from 6-8 down to 4-8 for Groups 3A and 3B. This brings it in line with the

			current group size in Group 3C. Our target attack rate remains unchanged at 50% +/- 10%. A minimum of 4 individuals will need to be enrolled at each dose, with *none* having been infected at that dose, in order to dose escalate after only 4 volunteers. If 1 or more of the first 4 volunteers develops infection the group will be expanded.<br><br>To date, all volunteers in Group 1 have been well. We have safely dose escalated to $1x10^4$ TCID50 in previously infected, vaccinated volunteers (Group 1), with low rates of infection. Therefore, in the uninfected vaccinated volunteers (Group 3), we believe inoculating a minimum of 4 (rather than 6) volunteers in each dose escalation group will enable efficient dose escalation, whilst giving us the option to expand group size should we successfully infect any volunteer in Group 3A or 3B.
1. Synopsis<br>6. Study Design<br>8.9 Dose escalation, de-escalation and confirmation	Maximum dose of $1x10^4$ $TCID_{50}$	Addition of group 1E and 3D at the $1x10^5$ $TCID_{50}$ dose level	Having not met our target attack rate using doses of $1x10^4$ $TCID_{50}$ of SARS-CoV-2 inoculum we plan to dose escalate to a dose of $1x10^5$ $TCID_{50}$. Doses of up to 10^4 $TCID_{50}$ have been well tolerated thus far in our study .<br>This escalation has been fully approved by both the study TSG and DSMB and is in line with reccomendations from the WHO advisory group, proposing stepwise dose escalation from ~10^2 to ~10^5 $TCID^{50}$ in first-in-human challenge studies with SARS-CoV-2[3].
1. Synopsis<br>8.9 Dose escalation, de-escalation and confirmation	Maximum group size in Group 2 and 4 is 40 respectively.	Reduction in maximum group size of Groups 2 and 4 to 30	Based on current recruitment, additional groups and study timelines, Groups 2 and 4 have

			been reduced for more feasible completion.<br><br>For funding purposes, this also ensures that the maximum participant number for the study remains in the previous level, without impacting the validity of the study.
1. Synopsis<br>6. Study Design<br>8.9 Dose escalation, de-escalation and confirmation<br>11. Statistics	Up to 136 participants total	Up to 132 participants total	Maximum participant number amended in line with the above changes.
1. Synopsis<br>3.13 Pre-clinical and clinical experience with REGN-COV2 (Regeneron Monoclonal Antibody cocktail Or Ronapreve) and Paxlovid for COVID-19:<br>3.15 Mitigating Risk in Experimental Human SARS-CoV-2 Infection<br>5.2 Data Safety Monitoring Board<br>6 Study Design<br>7.3 Inclusion and exclusion criteria<br>7.5 Withdrawal of volunteers<br>8.3 Study Procedures<br>8.4 Treatments administered<br>8.6. Study Visits<br><br>Table7. Risks associated with	Rescue therapy: 1200mg intravenous infusion of REGN COV2 (A monoclonal antibody cocktail against the SARS-CoV-2 spike protein)	Rescue therapy: Paxlovid (300mg PF 07321332 with 100mg ritonavir) taken twice orally daily for 5 days[1] or 1200mg intravenous infusion of REGN COV2 (A monoclonal antibody cocktail against the SARS-CoV-2 spike protein)<br><br>1. Once paxlovid is fully approved following SA007, REGN COV2 will be discontinued as rescue therapy	Supply of Paxlovid from local NHS pharmacy has been approved by the Programme Board of the Department of Health and Social Care's Antivirals and Therapeutics Taskforce. We continue to feel that there is need for an efficacious rescue medication for the prevention of severe COVID-19 disease for the COV-CHIM01 study. We would support the provision of medicinal products with the greatest evidence for efficacy and the lowest risk of complication. We feel that the current evidence base would currently support the use of Paxlovid in this capacity and therefore have replaced REGN COV2 with Paxlovid in this ammendment. See 3.13 for full justification.<br><br>Following full approval of paxlovid (by both ethics and internal green lighting procedures), use of REGN COV2 will be discontinued in the study.<br><br>Wording for REGN-COV2 remains in the protocol for clarity of past use.

other Study procedures<br>8.8 Blinding<br>8.10 Rescue treatment with REGN COV2 (Ronapreve) **or Paxlovid**<br>8.14 Schedule of Attendances<br>9.2 REGN COV2 **and Paxlovid**<br>10.2 Foreseeable adverse reactions<br>10.4 Causality			
6.4 Potential Risks for volunteers<br>10.2 Foreseeable adverse reactions	Manufacturer IB and SmPC listed	Reference to SmPC only	As the rescue therapy is now marketed, reference to the IB is no longer relevant and has been replaced with reference to the SmPC throughout the document.
7.3 Inclusion and exclusion criteria	17. Shares a household/ is in a support bubble with someone with clinically significant	*Inclusion criteria*: b. For women of child bearing potential (WOCBP) taking the combined oral contraceptive pill, a willingness to use barrier contraception with spermicide for 30 days after completing treatment with paxlovid, should they receive it.<br><br>*Exclusion criteria*: Concurrent use of medication contraindicated for use with Paxlovid rescue therapy<br><br>17.Shares a household with someone with clinically significant immunodeficiency (due to underlying medical	Updated in view of potential for drug interactions with paxlovid<br><br>Updated due to change in government guidellines

	immunodeficiency (due to underlying medical condition, medication or pregnancy); or who is extremely clinically vulnerable as per Public Health England guidelines	condition, medication or pregnancy); or who is extremely clinically vulnerable (previously shielding under Public Health England guidelines)	
7.2 Informed Consent<br>7.5 Withdrawal of Volunteers<br>8.11 Discharge from confinement<br><br>and<br><br>8.13 Unscheduled additional visits for individuals with possible or confirmed COVID-19 in post quarantine follow up period	The participant will be counselled about the risk of onward transmission of SARS-CoV-2 and the legal requirement to self-isolate according to PHE and government rules at the time.	The participant will be counselled about the risk of onward transmission of SARS-CoV-2 and advised to self-isolate according to PHE and government guidance at the time	There is no longer a legal requirement to isolate in England, however the study team will continue to advise participants to self-isolate should they withdraw post-enrolment as an additional public safety measure.
8.13 Unscheduled additional visits for individuals with possible or confirmed COVID-19 in post quarantine follow up period	Following discharge, participants will be asked to contact the study team should they develop any new* symptoms consistent with possible COVID-19 infection. If this occurs within 90 days following a positive SARS CoV-2 PCR from their quarantine stay they will be asked to attend a "suspected COVID-19 visit"<br>*and*<br>If symptoms occur more than 90 days	[Volunteers] will be asked to undergo testing for SARS-CoV-2 via the usual routes available to them e.g. occupational health testing. Volunteers will also be provided with lateral flow tests by the study team to ensure they have access to SARS-CoV-2 testing. Any swab results positive for SARS-CoV-2 will prompt a follow up "Positive COVID-19 test visit" …. volunteers with symptoms deemed by the investigator to be highly suspicious for COVID-19 (e.g. loss of sense of	Volunteers demonstrating PCR positivity in quarantine will now be treated the same as other volunteers with respect to suspected/confirmed COVID-19 visits and may undertake initial testing for COVID-19 in the community, regardless of infection status in the quarantine unit, as the primary means of testing available to most people is now LFTs. The risk of a false positive test is therefore low. A provision has been added to invite volunteers for review where their symptoms are highly suspicious of COVID-19 regardless of test result due to the higher false negative rate of LFT vs PCR

	after a positive PCR on the quarantine unit or if individuals do not test positive during their quarantine stay, then they will be asked to undergo testing for SARS-CoV-2 via the usual routes available to them e.g. NHS test & trace service or occupational health testing. The study team will follow up the swab results.<br><br>*New symptoms that warrant SARS-CoV-2 testing will be those outlined by current government requirements for testing. At the time of writing these are:<br>• Fever >37.8°C (excluding post vaccination fevers that are exempted from self-isolation/SARS-CoV-2 testing)<br>• New onset persistent cough	smell) may prompt a "suspected COVID-19 test visit".<br><br>*New symptoms that warrant SARS-CoV-2 testing will include the following:<br>• Fever >37.8°C (excluding post vaccination fevers)<br>• New onset persistent cough<br>• New onset change or loss in sense of smell and/or taste<br>Volunteers may also be asked to undertake a COVID test at clinician discretion (e.g. developing symptoms compatible with COVID not included in the above list).	Due to the change in current government guidelines with no further free PCR testing nor free LFTs, volunteers will be provided with LFTs by the study team in order to ensure ongoing surveillance of community-acquired SARS-CoV-2 infection.
8.6 Study Visits	Detailed explanation of study information included in the pre-screening video appointment	Prospective participants will be provided a pre-recorded video with a detailed explanation of study information.<br>The pre-screening video appointment will be	Pre-screening video calls to date have taken a significant amount of time. To most effectively utilise clinician time and screen prospective volunteers more efficiently, the same study information will be conveyed in a pre-recorded video, while at the

		shorter in length, to cover any questions.	same time keeping the pre-screening video call with the Doctor to continue to allow time for questions and clarification. This will also ensure greater standardisation of wording used.
8.14 Schedule of attendances	Discharge MRI will only be undertaken in those volunteers who demonstrate evidence of PCR positivity during the quarantine stay. This does not **exclude** those volunteers who have evidence of residual inoculum at the Day 1 timepoint only.	Discharge MRI will only be undertaken in those volunteers who demonstrate evidence of PCR positivity during the quarantine stay. This does not **include** those volunteers who have evidence of residual inoculum at the Day 1 timepoint only.	Typographical error corrected
9.1 SARS-CoV-2 virus	One GMP vial will therefore be used for each volunteer inoculation with no further manipulation or dilution required outside of the GMP facility	As per communication with MHRA the virus is a non-CTIMP, therefore neither the master stock nor dilutions are subject to conditions set out in Article 13(1) Directive 2001/20/EC. The undiluted challenge virus (Master Virus Bank) has undergone extensive quality testing… Selected individual inoculum vials that have been prepared at the various dilutions required for the study may also be tested<br><br>All challenge virus vials will be supplied by hVIVO	Provision to use a single vial for 2 or more volunteers was added as part of SA006, however revisions of this sentence missed. This has now been revised in line with SA006.<br><br>Clarification of production, supply and testing of the SARS-CoV-2 virus<br><br>.
9.4 Duckbill face mask	Collection matrix samples at each time point will be sent to our collaborators at the University of Leicester for viral assays.	Collection matrix samples at each time point will be sent to our collaborators at the University of Leicester for viral assays, for infected volunteers only.	To date, all masks have been shipped to Leicester however beneficial data is more likely to be gained from infected volunteers. To reduce unnecessary shipments, masks from infected volunteers will be sent to Leicester for analysis, whilst masks from uninfected volunteers may be sent as required. E.g. for negative controls.

10.2 Foreseeable adverse reactions	The expected reactions to REGN COV-2 are defined as those outlined in the reference safety information section of the manufacturer's current Investigational Brochure.	The expected reactions to REGN COV-2 and Paxlovid rescue therapies are defined as those outlined in the SmPC (www.gov.uk).	Paxlovid added, reference to IB removed
10.4 Causality	For every AE, an assessment of the relationship of the event to the administration of the challenge agent SARS-CoV-2, REGN-COV2, or other study procedure, will be undertaken	For every AE, an assessment of the relationship of the event to the administration of the challenge agent SARS-CoV-2, REGN-COV2, **Paxlovid**, or other study procedure, will be undertaken	Paxlovid added in AE assessment

Protocol v9.0 Substantial Amendment 6

Section	From	Changed To	Rationale for change
1. Synopsis,<br>6. Study design		Addition of group 1D and 3C	Having not met our target attack rate using doses of 10^3 $TCID_{50}$ of SARS-CoV-2 inoculum we plan to dose escalate to a dose of 10^4 $TCID_{50}$. Doses of up to 10^3 $TCID_{50}$ have been well tolerated thus far in our study and there is precedent for the use of doses of 10^4 $TCID_{50}$ and higher in other viral human challenge models[4] Specifically a study using doses of $10^{3.7}$ and $10^{4.7}$ $TCID_{50}$ RSV in human volunteers found no symptoms or signs of lower airway disease and a study using 10^7 $TCID_{50}$ influenza A identified no severe complications[5].<br><br>See cover letter for full justification
1. Synopsis	Planned Study Duration: 18 months	Planned Study Duration: 24 months	Estimate for study duration revised on the basis of the addition of 2 new groups in SA06 and our current progress.

6. Study design		Clarification that dose escalation will be capped at 10^4 TCID50 and we will proceed to dose confirmation, following DSMB safety review, even if we do not meet our target attack rate of 50% +/- 10%, to enable a larger sample size to assess the dynamic range of protection at that dose and ensure confidence in the negative infection rate at that dose.	In line with DSMB discussion that enrolling into our dose confirmation group even if target attack rate is not reached is important to broaden the sample size and ensure confidence in the negative infection rate.
7.3.1 Inclusion criteria	For Groups 1 & 2: Previous microbiological confirmation of SARS-CoV-2 infection > 3 months prior to enrolment (Proof of positive PCR confirmed via medical notes/ or PHE).	For Groups 1 & 2: Previous microbiological confirmation of SARS-CoV-2 infection > 3 months prior to enrolment (Proof of positive PCR *or lateral flow antigen test* confirmed via medical notes/ or PHE).	In view of the government's suspension of the need for confirmatory PCR following a positive lateral flow as of January 11th 2022 we will accept documentary evidence of a positive lateral flow device.
7.3.2 Exclusion Criteria,<br>8.3.13 Patient Health Questionnaire (PHQ-9) and Generalised Anxiety Disorder (GAD-7) Questionnaire	Psychiatric illness including volunteers with a history of depression and/or anxiety with associated severe psychiatric comorbidities, for example psychosis. Specifically,<br>i) Volunteers with history of anxiety-related symptoms of any severity within the last 2 years if the Generalized Anxiety Disorder-7 score is ≥5<br>ii) Volunteers with a history of depression of any severity within the	History of severe psychiatric illness at any time (e.g. inpatient stay, psychosis) or current significant active symptoms of anxiety and/or depression or significant claustrophobia. Consider exclusion in the following cases:<br>i) Volunteers with history of anxiety-related symptoms of any severity within the last 2 years if the Generalized Anxiety Disorder-7 score is ≥5<br>ii) Volunteers with a history of depression of any severity within	Wording changed to clarify that a volunteer with any psychiatric condition necessitating an inpatient stay or causing psychosis would be excluded rather than just depression/anxiety. This had been flagged as unclear in a recent monitoring visit.<br><br>'Consider exclusion' added to allow for discretion in screening participants.<br><br>Whilst a GAD-7 score ≥5 or a PHQ-9 score ≥4 will trigger a review and discussion with volunteers there are instances where participants may score highly due to confounding factors such as e.g. shift work causing sleep disruption in a volunteer who otherwise has mild symptoms of depression, has tolerated self-isolation well previously and feels their mood is stable.

	last 2 years if the Patient Health Questionnaire-9 score is ≥4	the last 2 years if the Patient Health Questionnaire-9 score is ≥4<br>iii) Significant claustrophobia<br>This change is further elaborated on in 8.3.13	
7.3.2 Exclusion Criteria	Participation in another research study involving receipt of an investigational product in the 30 days preceding enrolment, or planned use during the study period	Participation in any other research trial which involves receipt of an investigational product or drawing of blood. Additionally participants must not have had an investigational product within the 30 days preceding enrolment into COV-CHIM01.	To clarify that participants may not be co-enrolled in a research trial (CTIMP) or study involving drawing of bloods throughout COV-CHIM01 enrolment
7.3.4 Challenge Postponement Criteria		Addition of: Evidence of recent SARS CoV-2 infection (<3 months) based on a suggestive clinical history or investigations such as repeat serology, lateral flow antigen tests or PCR. Volunteers in group 3 & 4 could be re-enrolled after 3 months if appropriate into group 1 or 2. Volunteers in group 1 or 2 would have to defer participation for 3 months.	Clarification in line with study inclusion/ exclusion criteria that recent COVID infection would lead to challenge postponement.
8.4.4 Prohibited medication		Any medication or product (prescription or over-the-counter), for symptoms of nasal congestion or respiratory tract infections including nasal steroids. Washout changed from 7 days to 30 days	Typographical error noted. Changed from 7 to 30 days to bring in line with exclusion criteria and also the 30 day wash out period specified in the PIS and quarantine PIS
8.13 Unscheduled additional visits for		Volunteers who have attended for a 'positive	Collecting samples from participants who have been

individuals with possible or confirmed COVID-19 in post quarantine follow up period		COVID-19' visit or a 'suspected COVID-19' visit where they were found to have been infected with SARS-CoV-2 will attend for a further '4 weeks post COVID-19 infection' follow up visit at 4 weeks (-1 week/+2 weeks) from their suspected/positive COVID-19 visit…These visits may coincide with pre-planned follow up visits. If this is the case swabs and bloods will not be duplicated.	infected in the community 4 weeks following re-infection enables valuable information to be gathered about the maturation of the immune respone to natural infection.<br><br>Additional blood taken for exploratory immunology at unscheduled visits will not total more than 125mls throughout the whole duration of the study. This has not been changed in this amendment.
8.14 Schedule of attendances	Additional unscheduled suspected COVID/COVID positive visit exploratory immunology blood volume: 30ml	exploratory immunology blood volume: *62.5ml*	Additional blood volume for exploratory immunology previously described as 30mls, but with provision for additional bleeds where the total blood drawn for immunology should not exceed 125mls at unscheduled visits across the whole study. 62.5mls as a default is safe and allows for the collection of additional valuable immunology data beyond that which could be obtained with 30mls. The maximum volume for additional bleeds for exploratory immunology across the whole study remains 125mls
		Addition of an optional 5mls serology for SARS-CoV-2 antibodies prior to enrolment (footnote O)	An additional 5ml of blood may be taken for serology prior to enrolment if clinical suspicion of asymptomatic infection, in order to ensure the volunteer continues to meet the inclusion/exclusion criteria (see 8.6.2)
	Combined mid-turbinate and oropharyngeal swab for qPCR +/- live viral assays [b]	Combined mid-turbinate and oropharyngeal swab(s) for detection of SARS-CoV-2 virus. [b]	Changed to clarify that additional swabs may be taken for e.g virus sequencing at suspected COVID/COVID positive visit as originally stated in our exploratory endpoint.
		Additional footnote: M Whilst every effort will be made to ensure vital signs are taken as close to a specified time point as	Allows for prioritisation of workload.

		possible minor deviations will be acceptable to allow for prioritisation of work load e.g. vitals taken 12 minutes post challenge where 10 minutes is the specified time point.	
8.3.10 Cardiovascular Magenetic Resonance, 8.11 Discharge from confinement, 8.14 schedules of attendances	CMR will be performed at baseline (before being confined), and when the participant is discharged from the clinical research unit	CMR will be performed at baseline (before being confined) in all volunteers, and at discharge from the clinical research unit only in individuals who demonstrate PCR positivity after 24 hours during the quarantine stay (brief PCR positivity in first 24 hours is considered to represent residual inoculum rather than true infection) when the participant is discharged from the clinical research unit	Discharge Cardiac MRI/ ECHO is a safety end point to assess the effect of SARS CoV-2 infection on the heart. Subjecting volunteers who demonstrate no evidence of PCR positivity to contrast is unnecessary and subjects them to the additional risk/ burden of the cardiac MRI or ECHO procedure.
8.3.11 Echocardiography, 8.11 Discharge from confinement, 8.14 schedules of attendances	An additional Echocardiogram will be obtained when they are discharged from the quarantine unit if CMR cannot be performed.	An additional Echocardiogram will be obtained when they are discharged from the quarantine unit if CMR cannot be performed (again, only in individuals who demonstrate PCR positivity during the quarantine stay, from 24 hours after inoculation).	As above
8.3.2 Mid turbinate (nose) and Oropharyngeal (throat) swabs for virus detection:		Addition: "Sequencing for variant typing may also be carried out at suspected COVID/COVID positive visits."	To clarify our intention to perform sequencing on samples for SARS-CoV-2 virus as per our exploratory endpoint.
8.6.2 Consent & Screening Visit		Addition of: Furthermore, if there is clinical suspicion of asymptomatic infection (e.g. high rates of infection locally) an additional 5ml of blood may be taken for serology prior to enrolment, in order to detect changes in antibody titre or anti-	As per the body of the text, evidence of asymptomatic infection may inform a decision to defer enrolment or change the assigned group from 1/2 to 3/4<br><br>The potential additional blood volume is trivial

		nucleocapsid antibody positivity which may indicate asymptomatic infection between screening and enrolment. Evidence of asymptomatic infection prior to entering quarantine may inform a decision to defer (+/- change groups) for participants with possible recent infection…	
8.8 Blinding	In addition, where research investigations require interpretation such as the discharge MRI	In addition, where research investigations require interpretation such as the *D5 CT scan*	Discharge MRI will only take place in successfully infected volunteers therefore blinding will not be possible.
8.9 Dose escalation, de-escalation and confirmation		Addition of group 1D and 3C<br><br>Figures revised in line with above changes	Rationale for dose escalation to 10^4 $TCID_{50}$ outlined above.<br><br>Dose escalation will be capped at 10^4 TCID50 regardless of attack rate in Group 1D. Therefore, following inoculation of at least 4 individuals in this group (regardless of attack rate). The study may proceed to group 2 following safety review from the DSMB. We will never enrol more than 5 individuals at one time due to capacity on the quarantine unit.
		Addition: <u>Given the evolving situation with Omicron it may become increasingly difficult to recruit to group 4 due to limited numbers of eligible participants who have not been previously infected. If it becomes clear that it is not feasible to recruit 10-20 subjects into Group 4 we may combine Groups 2 and 4 for analysis purposes aiming for up to 30 participants across both groups.</u>	Updated in light of high infection rates with Omicron that may hamper reruitment into Groups 3 and 4.

9.1.3 Dispensing and administration		Additional provision made for using a single vial of inoculum to dose 2 or more volunteers	At doses of 10^4 $TCID_{50}$ it may be desirable to use a single vial of SARS-CoV-2 inoculum to dose 2 or more volunteers to preserve stock. This will be carried out with careful handling of the inoculum as per the SOP.
16.1 Financing, Title Page		Addition of Department of Health and Social care (DHSC).	Final dose level and dose confirmation groups being funded by DHSC.
16.4 Compensation	The total amount compensated will be £4995 for full study participation. Additional compensation will be provided for any additional days in quarantine beyond day 17, and any unscheduled additional visits.	The total amount compensated will be between £4925 and £4995 for full study participation (depending on requirement for discharge cardiac MRI/echocardiogram). Additional compensation will be provided for any additional days in quarantine beyond day 17, COVID positive visits and any unscheduled additional visits.	With removal of discharge cardiac MRI/echo for PCR negative volunteers total compensation amount changes.

Protocol v8.0 Substantial Amendment 5

Section	From	Changed To	Rationale for change
Title page, 1. Synopsis	A dose finding human experimental infection study with SARS-CoV-2 in healthy volunteers with previous, microbiologically confirmed, SARS-CoV-2 infection.	A dose finding human experimental infection study with SARS-CoV-2 in healthy volunteers immunologically sensitised with either previous, microbiologically confirmed, SARS-CoV-2 infection and/or vaccination against SARS CoV-2.	The title has been amended in line with the addition of groups 3 & 4, vaccinated without prior infection
1.Synopsis, 3.5 Rationale for addition of groups 3 & 4		Addition of group 3 (dose finding group) & group 4 (dose confirmation) in previously uninfected	Full rationale for change outlined in section 3.5. A successful SARS-CoV-2 human challenge model may be utilised in

5.2 Data safety monitoring board,<br>6. Study design,<br>6.1 study groups,<br>6.2 study volunteers,<br>7.3 Inclusion and exclusion criteria,<br>8.3.9 Radiology,<br>8.6.2 Consent & screening visit,<br>8.9 dose escalations, de-escalations and confirmation,<br>8.10 Rescue treatment with REGN COV2 (Ronapreve),<br>10.9 Interim safety reviews,<br>11 Statistics		vaccinated volunteers to study groups.<br>Associated increase in sample size.	the future to allow assessment of new therapeutics. A safe model needs to account for the dynamic nature of protective immunity seen in the target population. This includes a range of immunological phenotypes including those who have previously been infected with SARS-CoV-2 (with or without vaccination) and those who are uninfected (with or without vaccination). Uninfected, unvaccinated volunteers are not included in this study as this sub-population is included in an ongoing, parallel study (NCT04865237). The addition of an uninfected vaccinated group to this study will allow us to compare and contrast both the dose required for infection and the immune response in these groups. Natural infection exposes individuals to a broader range of viral epitopes than vaccination. Furthermore, the route of natural infection may induce mucosal immunity that intramuscular vaccination may not. Challenge of vaccinated individuals in a controlled human infection model also provides the opportunity to assess both the dynamic range and duration of protective immunity post vaccination.
1.Synopsis,<br>Summary,<br>3. Background and rationale,<br>4. Objectives and endpoints,<br>8.4.4 Prohibited medication	Infected individuals only	Reference added to uninfected vaccinated throughout	As above
5.2 Data Safety Monitoring Board	"The DSMB is expected to meet approximately	"The DSMB is expected to meet as per the above or more frequently if required."	Clarification on frequency of DSMB meetings now study is underway. Due to recruitment and the length of cycle of quarantines, meetings every 4 weeks are not necessary as

	every 4 weeks during the period in which quarantines are taking place or more frequently if required."	Meetings will occur between dosing groups; to review the data from 4 unvaccinated volunteers at a given dose; prior to group 2 & 4 and if any SAEs or safety group holding rules are met.	there may be insufficient new data to review.
6.4.2 Chest radiography and lung CT scan,<br>8.3.9 Radiology	"An additional CT will occur at Day 11 in all individuals with evidence of SARS CoV 2 infection (defined by positive PCR or viral culture or deemed by investigator to have symptom, signs of investigations suspicious of SARS CoV-2 infection)."	Clarification of the criteria for when to undertake an additional CT scan at day 11 "in infected volunteers if deemed necessary by the investigators e.g. due to sequential positive PCR, ongoing live viral culture or ongoing symptoms, signs or investigations suspicious for SARS CoV-2 infection). In order to limit unnecessary radiation exposure scans will not occur for well volunteers with isolated non-consecutive PCR positivity who have demonstrated a normal CT scan at day 5."	To minimize unnecessary radiation exposure, clarity has been added regarding performance of Day 11 CT scans. Scans will not be performed isolated non consecutive PCR positivity in well volunteers.
7.3.1 Inclusion Criteria<br>8.6.2Consent & screening visit	Presence of either anti-spike antibodies or anti-nucleocapsid antibodies to SARS CoV-2 at screening visit for enrolment into Groups 1 &2.	Written confirmation of prior SARS CoV-2 infection is sufficient for proof of prior infection. Baseline antibodies will continue to be measured at screening.	To increase the dynamic range of protective immunity assessed in our study population, proof of antibody status at screening will no longer be required (however will continue to be measured).
8.4.4 Prohibited medication	'All medications other than those noted above are to be stopped before the planned date of viral challenge unless in the opinion of the chief	Removal of "herbal supplements and chronically used medications, vitamins or dietary supplements, including any medication known to be an inducer or inhibitor of cytochrome P450 enzymes" from Table 10.	Strict wash out period for herbal supplements and vitamins removed from Table 10. Their use will however be assessed by study team prior to admission to the quarantine unit and a decision made regarding whether or not they will affect safety or integrity of study data.

	Investigator the medication will not interfere with the study procedures or compromise subject safety'	Addition of:<br><br>'All medications (including over the counter and herbal supplements), other than those noted above are to be stopped before the planned date of viral challenge unless in the opinion of the Chief Investigator/ Lead Clinician the medication will not interfere with the study procedures or compromise subject safety.'	
8.9 dose escalations, de-escalations and confirmation	Progression to Group 2 after the attack rate has been met	Details of progression to group 2 if the target attack rate is not met.<br><br>"If a dose meeting our target attack rate is not found in group 1, then group 2 will continue at the last highest safe dose identified after discussion with the DSMB, to enable a larger sample size to assess the dynamic range of protection at that dose and ensure confidence in the negative infection rate at that dose."	Details added regarding group 2 following DSMB discussion if target attack rate not met and DSMB decision is not to escalate doses further. In this situation we would challenge group 2 at the last highest dose to enable a larger sample size to assess the full dynamic range of protective immunity at that dose and provide a larger sample size to fully assess ability to cause infection at that last dose.
8.3.4 Nasopharyngeal (nose) for RNA sequencing<br>8.13 Unscheduled additional visits for individuals with possible or confirmed COVID-19 in post quarantine follow up period<br>8.14 Schedule of Attendances	Follow up Nasopharyngeal swabs for RNA sequencing completed for all volunteers	Follow up Nasopharyngeal swabs for RNA sequencing will only be performed in volunteers who have demonstrated evidence of SARS CoV-2 positivity during the study. This includes volunteers seen with suspected or confirmed COVID post discharge at unscheduled visits.	Removal of deep nasal (nasopharyngeal swabs) from follow up visits for volunteers who have remained PCR negative throughout as provides no additional data of interest and leads to an additional procedure for study participants.

8.3.3 Nasopharyngeal (nose) for respiratory pathogen detection<br>8.13 Unscheduled additional visits for individuals with possible or confirmed COVID-19 in post quarantine follow up period<br>8.14 Schedule of Attendances	Nasopharyngeal swabs for identification of respiratory pathogens performed at Day -2 only.	Addition of nasopharyngeal swabs for identification of respiratory pathogens at discretion of study clinicians at post quarantine follow up visits (both scheduled and unscheduled visits for individuals with possible or confirmed COVID-19).	If volunteers disclose symptoms in keeping with a respiratory infection, a nasopharyngeal swab for PCR will be taken to assess exposure to a panel of respiratory pathogens to look for a cause for participants symptoms or look for evidence of co-infection if COVID positive.

Protocol v7.0 Substantial Amendment 4

Section	From	Changed To	Rationale for change
Title page	Dr Ingrid Cabrera Puig	Addition of new Project Managers Dr Rebecca Powell Doherty and Hannah Scott	New staff members to replace Dr Ingrid Cabrera Puig who has left study team.
1 synopsis,<br>6.1 study groups,<br>8.9 dose escalation<br>10.9 Interim Safety Reviews	Group sizes for group 1a, 1b and 1c of 8 participants.	Changed to 6-8 participants with dose escalation once attack rate of <3 participants has been confirmed as unachievable i.e. no infections after 6 participants.	Now that the study has started and we have good safety data we would like to be able to progress with enrolment if it becomes obvious our attack rate target at a dose level will not be met (i.e. 6 volunteers with no evidence of infection).
2 Abbreviations	CTRG Clinical Trials and Research Governance	CTRG removed, RGEA Research Governance, Ethics and Assurance added	In line with new name
5.2 Data Safety Monitoring Board,<br>10.9 Interim Safety Review		Addition of "Review of data from a minimum of 4 unvaccinated individuals prior to dose escalation of unvaccinated individuals".	Addition to duties for DSMB. Changed to account for new dosing strategy of unvaccinated individuals which recognises the increasing evidence that individuals with prior infection plus vaccination have superior immune protection from SARS CoV-2 infection
6.1 Study groups	44 participants	64 participants	Error in total participants corrected.
6.1 study groups,		Addition of caveat that if unvaccinated volunteers are enrolled; a minimum of 4	As the UK vaccination schedule progresses, it is unlikely we will be able to enrol unvaccinated

6.4.3 Cardiovascular Magnetic Resonance (CMR) imaging and Gadolinium contrast use<br>8.4.4 Prohibited Medication<br>8.9 dose escalation		unvaccinated individuals will need to be enrolled at each dose with no evidence of infection prior to dose escalation of any unvaccinated volunteers. This may mean enrolling unvaccinated volunteers at a lower dose group after dose escalation has occurred. If an unvaccinated volunteer did develop infection then we would create an unvaccinated subgroup at that dose and dose escalation in unvaccinated individuals would only be able to occur after confirmation that the target attack rate of 50% (+/-10%) in up to 8 participants will not be met.	volunteers into higher dosing groups. However, there is an increasing body of evidence demonstrating the immune benefit conferred by vaccination of convalescent individuals over natural immunity alone. For safety purposes, if an unvaccinated volunteer is recruited to enrol in an intermediate or higher dosing group (i.e. 1B or 1C) then they will only be vaccinated at that dose if at least 4 unvaccinated individuals have already received the immediate lower dose and demonstrated no evidence of infection or safety concerns.
7.3.2 Exclusion Criteria	History of use of drugs of misuse, with evidence of a negative drugs of misuse urine test required at screening and quarantine admission	Clinically significant history of use of drugs of misuse, with evidence of a negative drugs of misuse urine test required at screening and quarantine admission	Addition of "clinically significant" to criteria in line with other studies performed by the group. This allows investigator discretion for instances where a participant may have experimented but is not a regular drug user and has no plans to participate in use of recreational drug misuse in the future.
7.3.4 Challenge Postponement criteria	The CI has been granted access to the regional ICU network data for Thames Valley and Wessex. She will be in contact with the Network Manager (Adult Critical Care), currently Kujan Paramanantham, regarding regional capacity. In addition, the CI is in contact with the Oxford ICU Senior Clinical and Nursing team. The CI, or someone from her team will alert the Network Manager and the Oxford team a week prior to any planned infections, and again 2-3	The CI has been granted access to the regional ICU network data for Thames Valley and Wessex. If this demonstrates concern regarding capacity issues (i.e. Critcon above usual winter pressure levels) then she will be in contact with both the Network Manager (Adult Critical Care), currently Kujan Paramanantham, regarding regional capacity and the Oxford ICU Senior Clinical and Nursing team to confirm capacity prior to enrolment. The CI, or someone from her team will assess capacity at 7 days and 2-3 days prior to enrolment. Volunteers will be	Now that the NHS is no longer facing the pressures seen during the peak of the COVID pandemic seen in January 2021 we have adapted our protocol to reduce the workload on our ICU contacts. We will continue to check ICU capacity using the regional network data both 7 days and 2-3 days prior to inoculation.<br>If this flags any concerns beyond the usual winter bed pressures then we will liaise with our contacts on ICU (both regional and local) prior to enrolment of participants.

	days before, to ensure that there is local and regional capacity, should this be required. Volunteers will be deferred if there is not sufficient capacity. This is a precautionary measure in the unlikely event a participant requires hospitalisation due to a severe unforeseen adverse reaction. Bed capacity is not anticipated to be a recurring issue however this policy has been put in place as a safe guarding measure due to the current fluctuating bed pressures with the pandemic.	deferred if there is not sufficient capacity. This is a precautionary measure in the unlikely event a participant requires hospitalisation due to a severe unforeseen adverse reaction. Bed capacity is not anticipated to be a recurring issue however this policy has been put in place as a safe guarding measure due to the current fluctuating bed pressures with the pandemic.	
8.13 Unscheduled additional visits for individuals with possible or confirmed COVID-19 in post quarantine follow up period	Symptoms within or after 90 days of enrolment	Revised to symptoms within or after 90 days of a positive SARS CoV-2 PCR from their quarantine stay	The rationale for seeing participants with <u>suspected</u> COVID-19 within 90 days of enrolment is due to the fact that after infection SARS CoV-2 PCR can remain positive for up to 90 days due to persistent dead viral material. By seeing the participant we can investigate for live viral material to differentiate between new infection or persistent material from prior infection. This will only be true of individuals who develop infection during the quarantine period and we have therefore amended our protocol to reflect that.

Protocol v6.0 Substantial Amendment 3

Section	From	Changed To	Rationale for change

Title page	Oxford Clinical Research Facility (OxCRF)	Addition of new sub-investigator and co-author of protocol Dr Andrew Mawer<br>Experimental Medicine Clinical Research Facility (EMCRF)	New staff member<br><br>Updated in line with current policy
1 Synopsis<br>And<br>4 Objectives and endpoints	Once daily during quarantine period with a pre-challenge baseline measurement (D-1 to D14+/ until discharge criteria met).<br><br>Genome sequencing	Antigen detection via lateral flow tests added to exploratory endpoints<br><br>Face masks: Once daily during quarantine period with a pre-challenge baseline measurement (D-1 to D14+/ until discharge criteria met).<br>Other methods of virus detection will occur on selected samples.<br><br>Whole genome sequencing	To reflect updated plans to perform lateral flow tests. This will be performed on selected samples from swabs already taken for PCR, and no additional swabs or procedures will be done on participants for these tests.<br>While RT-PCR remains gold standard, point-of-care rapid lateral flow tests (LFTs) for SARS-CoV-2 antigen detection are an integral part of the current Public Health England strategy to control the pandemic. Studies have demonstrated high sensitivity of lateral flow for samples with active viral replication. With one study demonstrating a sensitivity of 94.7% in samples with detectable viral growth[6]. The addition here as an exploratory end point for viral detection will allow investigation of the utility of this tool to detect participants who are actively infectious, thus providing additional information on the utility of this tool for pandemic control.<br><br>Clarification
1.0 Synopsis, 2.Abbreviations, 6.1 Study groups, 8.6. study visits	Oxford University Hospitals NHS Trust or Oxford Clinical Research Facility	Experimental Medicine Clinical Research Facility	Changed to reflect permanent relocation of quarantine unit to the new Experimental Medicine Clinical Research Facility (EMCRF) with new name.
3.10.3 Personalised risk assessment using the QCOVID tool	By setting a risk threshold, the potential risks associated with particular participant features (such as	The potential risks associated with particular participant features (such as ethnicity, sex or BMI) may be balanced holistically and certain risk	As per section 3.14 rationale below

	ethnicity, sex or BMI) may be balanced holistically and certain risk factors mitigated by other characteristics such as younger age (Table 1). Thus, a healthy White British 30 year old man or woman; a Black African 22 year old man; a 26 year old Indian man; a 24 year old Chinese man; and a 27 year old Chinese woman of BMI 23.5 would all have an absolute risk of death of 1 in 250,000 (0.0004%), thus establishing parity and eliminating any potentially increased risk associated with ethnicity that currently remains unexplained.	factors mitigated by other characteristics such as younger age (Table 1). Thus, a healthy White British 30 year old man or woman; a Black African 22 year old man; a 26 year old Indian man; a 24 year old Chinese man; and a 27 year old Chinese woman of BMI 23.5 would all have an absolute risk of death of 1 in 250,000 (0.0004%), thus establishing parity and eliminating any potentially increased risk associated with ethnicity that currently remains unexplained. The QCOVID tool will be utilised in this setting to inform a global assessment of eligibility as well as providing further information as part of the participant's informed consent process. Absolute cut offs have been removed, however, scores will be considered as part of a holistic assessment of risk, including information on primary infection.	
3.12 Pre-clinical and clinical experience with REGN-COV2 (Regeneron Monoclonal Antibody cocktail) for COVID-19		Addition of:<br>Recent results from phase 3 studies has led to the licensure of REGN-COV-2 on 20th August 2021 (also known as Ronapreve) in the UK for both the prophylaxis and treatment of COVID-19 disease[7]. It has been administered to a total of 7116 subjects (approximately 4666 via IV administration and 2450 via subcutaneous administration.<br>In COV-2067 a randomised, double blinded, placebo-controlled phase 1-3 study in non-hospitalised participants. Individuals with at least one risk factor for severe COVID-19 received a single intravenous infusion of REGN-COV2 within 3 days of having a positive	With MHRA licensure of REGN COV-2 on 20th August 2021 the protocol and patient facing documents have been amended to reflect new information and recent licensure.

		SARS-CoV-2 PCR test. 4567 adult participants were randomized to receive one of three doses of REGN COV2 combination, either the 1,200mg dose (n=838), 2,400 mg dose (n = 1529) or the 8,000mg dose (n =700), or placebo (n = 1500). The primary end point of this study was the proportion of subjects with COVID related hospitalisation or all cause death through to Day 29. In the 1,200mg dose this end point was met in 7 individuals (1.0%) treated versus 24 in placebo (3.0%), demonstrating a 70% relative risk reduction (p=0.0024). Interim safety results released from this study demonstrated a tolerable safety profile, with serious adverse events being numerically more frequent with placebo than REGN-COV2 treatment (0.8% high dose REGN-COV2, 1.6% low dose REGN-COV2; 2.3% placebo). None of the SAEs were considered to be related to the study drug. Numerically more infusion reactions occurred with the REGN-COV2 high dose compared to placebo but not with the low dose REGN COV2 regime (1.5% high dose; 0% low dose; 0.4% placebo). This study demonstrated that overall, treatment resulted in a reduction in the average daily change in viral load through day 7 (mean time-weighted average change from baseline) with -0.71 log10 copies/mL for 1,200 mg dose ($p < 0.0001$) and -0.86 log10 copies/mL for 2,400 mg dose ($p < 0.0001$) compared to placebo. The greatest benefit in viral load reduction was seen in individuals who	

		had higher starting viral loads and those who were seronegative at baseline (I.e. slow immune responders)[7-9]. Self-reported symptom duration was also reduced in treated participants versus placebo, with a median duration of 10 days in 1200mg dose cohort versus 14 days for placebo treated subjects (p=0.0001). Results have demonstrated no significant difference in virologic, clinical efficacy, hypersensitivity reactions or SAEs across dose groups.	
3.14 Mitigating Risk in Experimental Human SARS-CoV-2 Infection	We will utilise the recently published QCOVID living risk prediction tool for an objective determination of risk with a strict absolute risk threshold of COVID-associated death of less than 1 in 250,000 (0.0004%) and COVID-associated hospital admission of less than 1 in 5000 (0.02%)(38).	Prior to enrolment, each potential participant deemed to be suitable for entry into the study (based on inclusion/exclusion criteria and their screening assessments) will undergo a personalised risk assessment using the QCOVID tool in combination with emergent results from individuals experimentally infected with SARS-CoV-2 in this study. The risk assessment will be reviewed by a member of the study medical team, documented and discussed with the participants before enrolment.	Whilst the individual risk factors of the QCOVID are important to consider and will continue to be used to ensure safety, the study team and DSMB have not found that the score per se adds additional safety value. The DSMB review and the study team have determined that a QCOVID risk assessment will be included and communicated with potential participants but that an absolute cut off will not be used as part of the inclusion/exclusion criteria at present. i.e. the QCovid risk score will be taken into account by the investigator, along with other possible risk factors, to decide final eligibility.<br>In addition, this change allows our inclusion/exclusion criteria to align with the SARS-CoV-2 naive challenge study running at Imperial College London
5.2 Data Safety Monitoring Board		Deleted planned review of the inclusion of QCovid tool in inclusion/exclusion criteria	
6.2 Study Volunteers	Study population will be microbiologically confirmed previous	Study population will be microbiologically confirmed previous SARS-CoV-2 infected	As above

	SARS-CoV-2 infected males and non-pregnant or lactating females between 18 and 30 years with no underlying co-morbidities. Eligibility will depend on review of medical and social history, physical examination, QCOVID risk score and the results of comprehensive testing including; laboratory tests, cardiac magnetic resonance imaging (or Echocardiogram), ECG, smell tests, chest x ray and pulmonary function testing. Volunteers will be considered enrolled immediately after receiving the intranasal viral challenge.<br><br>Note: At the end of Group 1, to broaden the diversity of volunteer recruitment, we will ask the DSMB to review our safety data and determine whether we should continue with the QCOVID risk assessment for enrolment into Group 2. The assessment will consider whether we continue with the criteria used in Group 1, relax the criteria for death and severe disease used as a cut off or stop using QCOVID altogether. This review will consider safety data from the Oxford and the Imperial study and will also include review of any relevant publications on risk and ethnicity.	males and non-pregnant or lactating females between 18 and 30 years with no underlying co-morbidities. Eligibility will depend on review of medical and social history, physical examination, and the results of comprehensive testing including; laboratory tests, cardiac magnetic resonance imaging (or Echocardiogram), ECG, smell tests, chest x ray and pulmonary function testing. In addition, a QCOVID risk score will be calculated and the risk discussed with the volunteer prior to enrolment. Although an absolute cut-off will not be used to determine eligibility, the QCOVID score will be used as part of a holistic assessment by study investigators when considering suitability of participants.<br>Volunteers will be considered enrolled immediately after receiving the intranasal viral challenge.	

7.3 Inclusion and exclusion criteria		Deleted: 3. Assessed using the QCOVID risk tool to have an absolute risk of COVID-associated death of less than 1 in 250,000 (0.0004%) and COVID-associated hospital admission risk of less than 1 in 5000 (0.02%).	As above
Table 5: Mitigating risk	Strict inclusion and exclusion criteria, combined with stringent health screening, will apply to ensure only healthy young adults with no known risk factors for severe COVID-19 are enrolled in this study. Use of QCOVID living risk prediction tool for risk determination.	Strict inclusion and exclusion criteria, combined with stringent health screening, will apply to ensure only healthy young adults with no known risk factors for severe COVID-19 are enrolled in this study. Use of QCOVID living risk prediction tool as a guide to inform risk determination.	
Table 6. Risks associated with other Study procedures		Text added: In addition please refer to the SmPC found at Summary of Product Characteristics for Ronapreve - GOV.UK (www.gov.uk)	Changed with recent licensure of REGN COV-2 by MHRA.
8.3.2 Midturbinate (nose) and oropharyngeal (throat) swabs for virus detection		Addition of following text:<br>Antigen detection (lateral flow tests) may also be performed on selected samples as part of our exploratory end points.	As above re: rationale for addition of lateral flow for exploratory end points.
8.6.2 Consent & screening		Text added:<br>QCovid score will be calculated with the patient present and discussed as part of a holistic assessment of risk.	In line with change to use of QCOVID tool as outlined previously. This highlights its use in the screening visit.
8.6.3 D-2 admission to quarantine unit (and commencement of inpatient period)		Text added:<br>During the inpatient quarantine period care will be delivered in line with Oxford University Hospital NHS inpatient policies and study standard operating procedures.	To add clarity that Oxford University Hospital NHS inpatient policies will be followed whilst on the EMCRF quarantine unit.
8.6.3 and 8.14 Schedule of attendance		Addition of weight recording at D-2 and every 7 days whilst in quarantine	OUH policy, which is being followed whilst in quarantine is to measure weight at

			admission and then weekly to ensure safe prescribing practices and monitoring of individuals. We will therefore measure weight at these timepoints in line with this policy. We have updated the protocol in order to also record this parameter as a research safety output.
8.9 Dose escalation, de-escalation and confirmation		Deleted text: assessing ongoing need for QCOVID risk assessment at screening	
9.2 REGN COV-2	REGN COV2 (combined REGN10933 and REGN10987) is currently unlicensed in the UK and being provided as a "special"	REGN COV2 (combined casirivimab and imdevimab) is recently licensed in the UK (20th August 2021).<br>REGN10933 and REGN10987 changed to casirivimab and imdevimab throughout.<br>REGN COV2 also referred to as Ronapreve	Changed with recent licensure of REGN COV-2 by MHRA.

Protocol v5.0 Substantial Amendment 2

Section	From	Changed To	Rationale for change
Exploratory endpoints		Addition of text: Cardiac MRI exploratory research images	To make more explicit that some cardiac images referred to in 8.3.10 Cardiovascular Magnetic Resonance (CMR) are not part of the clinical report and come under exploratory research
Background and Rationale		Small changes to text to bring it in line with the fact that we are now also enrolling volunteers who have been previously vaccinated with SARS-CoV-2	
7.3.2 Exclusion criteria<br><br>& 7.3.4 Challenge postponement criteria	Plans to receive any vaccination 30 days prior to enrolment and/or 30 days following enrolment	Plans to receive a live vaccination 30 days prior to enrolment, or any vaccination (i.e. non-live, including a SARS-CoV-2 vaccine) 21 days prior to enrolment. And/or plans to take any vaccination 30 days following enrolment	With increasing vaccine roll out across all ages including 18-30 year olds, it will be increasingly difficult to enrol unvaccinated subjects and importantly, it would not be appropriate for us to ask potential subjects to defer their vaccination.

		Deleted the following exclusion criteria: Has received a vaccination for SARS-CoV-2 either as part of a clinical study or as part of licensed vaccine rollout.	As our study aims to define protective immunity, the study aims remain intact. We will continue to require evidence of past infection as an inclusion criteria and will measure the effect of that past infection, +/- effect of vaccination, on our reinfection rates. We will be able to define non-spike immunogenicity to evaluate immunity induced by infection, as well as measure vaccine and infection induced immunogenicity against the spike antigen.
8.3.10 Cardiovascular Magnetic Resonance (CMR):	CMR will be performed at baseline (before being confined) and when the participant is discharged from the clinical research unit. The CMR protocol will include assessment of cardiac structure, function and myocardial tissue characterisation, including intravenous injection of a gadolinium-based contrast agent (e.g. Dotarem or Gadovist) per clinically-accepted CMR protocols. Participants will be screened for magnetic safety and any contraindications to CMR. Participants who successfully complete a baseline CMR scan will be invited for a follow-up CMR at or soon after discharge from the quarantine unit, for both safety and exploratory purposes. All scans will be reported based on cine and LGE imaging by an appropriately qualified healthcare professional.	CMR will be performed at baseline (before being confined) and when the participant is discharged from the clinical research unit. The CMR protocol will include assessment of cardiac structure, function and myocardial tissue characterisation. If intravenous access can be obtained, intravenous injection of a gadolinium-based contrast agent (e.g. Dotarem or Gadovist) per clinically-accepted CMR protocols, will be performed for late gadolinium enhanced imaging (LGE). Participants will be screened for magnetic safety and any contraindications to CMR. Participants who successfully complete a baseline CMR scan will be invited for a follow-up CMR at or soon after discharge from the quarantine unit, for both safety and exploratory purposes. All scans will be reported based on cine, +/- LGE imaging where available, by an appropriately qualified healthcare professional.	Addition of text to more explicitly state which images make up part of the clinical report and which images form part of research only (the parametric mapping images haven't been clinically validated and hence are not included in the clinical report.)

	CMR results will also be used for research purposes by both the study team and Professor Keith Channon group, as part of a collaboration within the framework of the Oxford Acute Myocardial Infarction (OxAMI) Study.	The CMR scan will include parametric mapping (e.g. T1/T2/Extracellular volume), which will not be included in the formal clinical report, but rather will form part of our exploratory research endpoints. Images will be used for research purposes by both the study team and Professor Keith Channon group, as part of a collaboration within the framework of the Oxford Acute Myocardial Infarction (OxAMI) Study.	
8.3.15 University of Pennsylvania Smell Identification Test (UPSIT)	…will be performed at baseline, daily during quarantine and study visit if there has been any evidence of anosmia/ parosmia in the 28 days after challenge.	..will be performed at baseline, every three days during quarantine, at the day 28 follow up visit and any suspected Covid-19 visit. We will also perform the smell test at any study visit if clinically indicated e.g. if UPSIT abnormal at previous timepoint or the volunteer reports subjective symptoms of anosmia/parosmia.	Performing the smell test every day during quarantine creates a learned response and may reduce the accuracy of the result. We are therefore changing the UPSIT test to every three days during quarantine. But if any clinical suspicion of anosmia/parosmia the UPSIT will be performed at any timepoint.
8.3.2 Mid Turbinate (nose) and Oropharyngeal (throat) swabs for virus detection		Addition of the following text: Briefly, qPCR will be performed on each swab and, to fulfil discharge criteria, immunofluorescence for detection of live virus will be performed on pre-discharge samples. In addition, to meet the secondary objectives describing the SARS-CoV-2 viral dynamics in upper respiratory samples, further live culture analysis of swab elution will occur on selected samples collected over the course of the study.	To make more explicit the details of the analysis
8.4.1 REGN COV2 (Regeneron monoclonal antibody cocktail)	Treatment with a single intravenous infusion of 1200mg REGN-COV2 will commence immediately after symptoms, signs or investigations suggestive of COVID-19 disease.	Treatment with a single intravenous infusion of 1200mg REGN-COV2 will be given to volunteers confirmed to be infected and who demonstrate any warning features of COVID-19 disease beyond mild signs and	As evidence accrues from the Imperial College Study of the safety of this model without any pre-emptive therapy, we would like to align our criteria for use of the Regeneron monoclonal antibody cocktail to the Imperial College study. In

		symptoms that are confined to the upper respiratory tract.	addition, our first two subjects in our study were transiently PCR positive (positive swabs on D6 pm and D7 am). Both were treated as per our protocol with Regeneron monoclonal antibody cocktail. However subsequent swabs on D7 pm and D8 am, taken prior to treating with Regeneron, were negative. We would like to avoid treating so early in future.
8.6.2 Consent & Screening Visit	This visit may take place up to 60 days prior to the challenge.	This visit may take place up to 90 days prior to the challenge.	Changed to bring the study protocol in line with the standard practice of clinical studies occurring in our Oxford Vaccine Centre. These are healthy young volunteers at low risk of their health condition changing over this time. We also already subject all volunteers to a repeat screen of pertinent tests at D-2 which should pick up any changes to medical criteria significant to eligibility criteria prior to confirming eligibility and enrolment at D0.
8.10 Rescue treatment with REGN COV2<br><br>And throughout to line up with changes to threshold criteria to deliver REGN-COV2		A single 60 minute intravenous infusion of 1200mg of REGN COV2 will commence in any volunteer confirmed to be infected with SARS-CoV-2 and with any of the following:<br><br>Persistent tachypnoea – Respiratory Rate ≥ 21 for ≥ 8 hours<br>Persistent Fever – Fever (≥ 37.9) from a time point 5 days post symptom onset and present for at least once each day for ≥ 72 hours<br>Severe and persistent cough – Grade 3 reports of coughing via symptom diary cards that is largely persistent over 48 hours (Grade 3: Significant discomfort with marked limitation in activity).	As above

		For CT changes related to SARS CoV-2 infection, anything more than mild changes (based on the British Society of Thoracic Imaging COVID reporting scale) represents a standalone trigger and mild changes in combination with other concerning clinical features could also be considered.<br>Any event of confirmed hypoxia (≤ 94%, usually confirmed over a 1-hour period)<br><br>Importantly CI discretion will be used at all times in the decision to start REGN-COV2. Biochemical markers (e.g. elevated CRP/D-dimer) will not be used in isolation, though a combination of factors outside of the triggers above could still lead to a decision to start therapy. Once a decision to commence rescue therapy has been made by the CI/PI, the NHS Infectious Diseases team at the Oxford University Hospital, will be made aware. The subject will remain within the quarantine unit to receive REGN COV2 therapy unless protocol stated criteria for transfer to NHS care are met.	
Schedule of Attendance		Updated to reflect changes to UPSIT test timeline, extension of screening visit period and to clarify mid-turbinate swab tests	

Protocol v4.0 Substantial Amendment 1

Section	From	Changed To
Schedule of Attendance	818ml blood taken in total	865.5ml (to accommodate a 2.5ml paxgene blood tube at each exploratory timepoint for RNA-sequencing (with the exception of the symptomatic covid unscheduled visits); and up to an additional 20ml baseline blood for exploratory immunology)<br>Addition of mid-turbinate swab for RNA-sequencing

	Baseline nasosorption Day -2<br><br>Baseline naso-pharyngeal swab D0	Moving baseline nasosorption to Day -1 due to space out nasal sampling and clarifying this is two samples (BD)<br>Baseline naso-pharyngeal swab D-2 to avoid disruption to nasal mucosa so close to D0 innoculation<br>Addition of smell test at screening (to help exclude volunteers with ongoing symptoms of long covid from primary infection)
Throughout	Combined nose (Naso-pharyngeal) and throat (oro-pharyngeal) swab for SARS-CoV-2 viral detection	Combined nose (mid turbinate) and (throat) oro-pharyngeal swab for SARS-CoV-2 viral detection<br>(Also referred to as a nasal-oropharyngeal swab in some places)
Key Trial contacts		Updated
Table 6: Risks	818ml (+up to an additional maximum total of 120ml<br><br>Systemic hypersensitivity reactions & acute infusion reactions; symptoms can include fever, chills, nausea, vomiting, abdominal pain, headache, dyspnoea (shortness of breath), hypotension, angioedema, throat irritation, urticarial rash, pruritus, myalgia, and/or dizziness.<br>Infusion & hypersensitivity reactions in published data are rare (0% with 2.4g dose, 1.5% in 8g dose).	865.5ml (+up to an additional maximum total of 125ml)<br><br>Updated in line with current IB (v5.0): Systemic hypersensitivity reactions & acute infusion reactions; symptoms can include fever, chills, nausea, vomiting, flushing, abdominal pain, chest tightness, headache, dyspnoea (shortness of breath), hypotension, angioedema, throat irritation, urticarial rash, pruritus, myalgia, and/or dizziness.<br>Infusion & hypersensitivity reactions in published data are rare (1.8% with 2.4g dose, 3.1% in 8g dose).<br>(Note this is based on known adverse reactions as per IB version 5 safety data cut-off date 27 January 2021)
6.1 Study Groups	Volunteers will be recruited from the Thames Valley region with screening and outpatient follow up visits at the Clinical Centre for Vaccinology and Tropical Medicine.	Volunteers will be recruited who are able and willing to attend regular screening and outpatient follow up visits at the Clinical Centre for Vaccinology and Tropical Medicine.
8.3 Study procedures	8.3.2 Nasopharyngeal (nose) and Oropharyngeal (throat swabs) for virus detection:	8.3.4 Nasopharyngeal (nose) for RNA-sequencing<br>Two nasopharyngeal swabs will be taken on the same days that immunology bloods are taken for RNA-sequencing, as detailed in the SoA<br><br>Change from nasopharyngeal swab for viral detection to mid-turbinate. Text changed as follows:<br>8.3.2 Mid turbinate (nose) and Oropharyngeal (throat) swabs for virus detection:

	Combined nose/throat swabs will be taken for SARS-CoV-2 viral detection and quantitation using study SOPs as detailed in the SoA. At admission to the quarantine unit an additional nasopharyngeal swab will be taken to screen for respiratory pathogens including SARS-CoV-2 prior to enrolment.	Combined mid turbinate nose and oropharyngeal throat swabs will be taken for SARS-CoV-2 viral detection and quantitation using study SOPs as detailed in the SoA.<br><br>8.3.3 Nasopharyngeal (nose) for virus detection:<br>At admission to the quarantine unit a nasopharyngeal swab will be taken to screen for respiratory pathogens including SARS-CoV-2 prior to enrolment.
8.14 Schedule of Attendances		Addition of smell test to first screening visit. This allows the study team to exclude any participants with possible persistent anosmia post primary COVID infection at an earlier stage in the screening process.
8.9 Dose escalation, de-escalation and confirmation<br><br>And throughout	<u>Group 1:</u><br>The starting dose for Group 1A (10^1 TCID50) was selected as the lowest reliably quantifiable amount of virus. At this lowest dose, three individuals will be challenged. If any of the first 3 participants in Group 1A develop infection after 14 days and no pausing rules are met, the remaining 5 participants in the group will be challenged at the group 1A dose (10^1 TCID50). However, if none of the first three sentinel individuals in group 1A develop infection after 14 days and no pausing rules are met (based on severity and frequency of adverse events, see Group Safety Holding Rules), the dose would instead be escalated to Group 1B dose (10^2 TCID50).<br>With each subsequent new dose level, three individuals will be challenged. After 14 days, then either the next 5 participants in the group can be challenged (if any of first three individuals develop infection) or the dose will be escalated.	Table 4 and Figure 6 updated and text changed to allow all 8 volunteers to be enrolled into each sub group regardless of the infection status of first 3 in that group<br><u>Group 1:</u> Groups of up to 8 individuals will be challenged at each dose and dose escalation will proceed as follows.<br>The starting dose for Group 1A (10^1 $TCID_{50}$) was selected as the lowest reliably quantifiable amount of virus. 8 participants in the group will be challenged at the group 1A dose (10^1 $TCID_{50}$).<br>The attack rate threshold for the 8 participants for any given Group 1 sub-group is 50% +/- 10%. i.e we would aim for 5 participants but a minimal threshold as low as 3 participants would be acceptable dependent on other factors such as clinical symptoms as outlined in the note below. Once this attack rate has been met we will move on to Group 2 (details below).<br>If the number of infected participants in the first Group 1 sub-group does not meet this pre-defined attack rate threshold (50% +/- 10%), the next sub-group will be inoculated with a higher dose. ie if 2 or fewer volunteers in the subgroup develop infection after 14 days and no pausing rules are met (based on severity and frequency of adverse events, see Group Safety Holding Rules), the dose would be escalated to the next sub group (following DSMB review of data).
Section 5.2 Data Safety		Addition of the following text as we realised this was missed out in error:

Monitoring Board		After the first 10 volunteers have been enrolled into Group 2, and again (if applicable) after 20 volunteers, to determine if further enrolment is required into Group 2
Section 9.2	6000mg REGN10933 & 6000mg REGN10987	Typographical error corrected<br>600mg REGN10933 & 600mg REGN10987
Section 10.9 Interim Safety Reviews	For the dose escalation, three volunteers will receive a new dose and the study investigators will wait a minimum of 14 days and until live virus negative before a safety review by the CI. If no safety concerns (see group holding rules) and at least one individual has developed SARS-CoV-2 infection then a further 5 volunteers will be enrolled at the same dose. If no volunteers have developed infection then we will refer to the DSMB for consideration of dose escalation. The next group of three volunteers at the next higher dose of SARS-CoV2, will be challenged only following favourable review by the DSMB, and a minimum of 7 days after the last volunteer has been discharged.	For the dose escalation, eight volunteers will receive a new dose and the study investigators will wait a minimum of 14 days and until live virus negative before a safety review by the CI. If less than three volunteers have developed infection then we will refer to the DSMB for consideration of dose escalation. The next group of eight volunteers at the next higher dose of SARS-CoV2, will be challenged only following favourable review by the DSMB, and a minimum of 7 days after the last volunteer has been discharged.

1 SYNOPSIS

**Title**	A dose finding human experimental infection study with SARS-CoV-2 in healthy volunteers immunologically sensitised with either previous SARS-CoV-2 infection and/or vaccination against SARS-CoV-2
**Study Identifier**	COV-CHIM01
**Study Centres**	Oxford University Hospitals NHS Foundation Trust (OUH),<br>Headington, Oxford,<br>OX3 9DU<br><br>Centre for Clinical Vaccinology and Tropical Medicine<br>University of Oxford<br>Churchill Hospital, Old Road, Headington<br>Oxford, OX3 7LE<br><br>Experimental Medicine Clinical Research Facility (EMCRF)<br>Churchill Hospital<br>Old Road, Headington<br>Oxford, OX3 7LE
**Clinical Phase**	Phase I, Experimental
**Design**	Dose finding open label clinical infection, safety and viral detection optimisation in previously SARS-CoV-2 infected (unvaccinated or vaccinated) or uninfected vaccinated volunteers.
**Population**	Healthy volunteers aged 18-30 years either with a) previous SARS COV-2 infection > 7 weeks prior to enrolment or b) previously uninfected vaccinated volunteers.
**Planned Sample Size**	Total: Up to 132 participants<br><br>**Group 1. Dose finding clinical challenge, safety and viral detection optimisation of previously SARS-CoV-2 infected volunteers +/-vaccination**:<br><br>a) Intranasal viral challenge with $1x10^1$ $TCID_{50}$, N=6-8<br>b) Intranasal viral challenge with $1x10^2$ $TCID_{50}$, N=6-8<br>c) Intranasal viral challenge with $1x10^3$ $TCID_{50}$, N=6-8<br>d) Intranasal viral challenge with $1x10^4$ $TCID_{50}$, N=4-8<br>e) Intranasal viral challenge with $1x10^5$ $TCID_{50}$, N=4-8

Group 2. Safety and dose confirmation in previously SARS-CoV-2 infected volunteers +/-vaccination:

Intranasal viral challenge in 10-30 participants with the dose identified from Group 1 (dose finding stage).

Group 3. Dose finding clinical challenge, safety and viral detection optimisation in previously uninfected SARS-CoV-2 vaccinated volunteers.

a) Intranasal viral challenge with $1x10^2$ $TCID_{50}$, N=4-8
b) Intranasal viral challenge with $1x10^3$ $TCID_{50}$, N=4-8
c) Intranasal viral challenge with $1x10^4$ $TCID_{50}$, N=4-8
d) Intranasal viral challenge with $1x10^5$ $TCID_{50}$, N=4-8

Group 4. Safety and dose confirmation in previously uninfected SARS-CoV-2 vaccinated volunteers.
Intranasal viral challenge in 10-30 participants with dose identified from Group 3 (dose finding stage).

Visit Schedule

Screening visit followed by SARS-CoV-2 Challenge (D0) with inpatient admission period (D-2 to approximately D14) in the quarantine unit for close monitoring. Discharge will be when two negative sequential samples for viable SARS-CoV-2 are obtained. Subsequent follow up visits scheduled at days 28, 56, 84, 168 and 365. Unscheduled visits for suspected re-infection with SARS-CoV-2 after discharge from quarantine will occur if necessary.

Planned Study Duration

38 months (06 May 2021 – 06 July 2024)

Objective	Outcome Measure

**Co - Primary**	To assess safety and human clinical response to wild type SARS-CoV-2 intranasal challenge in both previously infected (unvaccinated or vaccinated) and uninfected vaccinated volunteers.	Measured by solicited and unsolicited adverse events and other objective parameters including physical examinations, smell test, cognitive tests, vital signs, pulmonary CT, pulmonary function tests, ECG , cardiovascular imaging (MRI or echocardiogram) and clinical laboratory results.
**Co-Primary**	Selection of the SARS-CoV-2 dose(s) required to induce upper respiratory tract infection in 50% (+/-10%) of previously SARS-CoV-2 infected (vaccinated or unvaccinated) and uninfected vaccinated healthy volunteers following intranasal challenge.	Defined by laboratory identification of SARS-CoV-2, from nasopharyngeal swab, using qPCR and/or quantitative live viral detection at two consecutive 12- hourly time points starting 24 hours post-inoculation and up to discharge from quarantine.
**Secondary**	To assess the SARS-CoV-2 viral dynamics in upper respiratory samples from previously infected or uninfected vaccinated individuals including; determination of the incubation period, peak viral load and the mean duration of infectious viral shedding.	From quantitative virology measured twice daily during quarantine using qPCR and/or quantitative live viral detection.
	To identify laboratory markers of the immune response that correlate with the levels of viral load.	Laboratory markers of innate and adaptive immunity, including but not limited to ex-vivo ELISpot, flow cytometry, ELISAs, transcriptomic analysis, neutralising antibody activity, and cytokine analysis.
**Exploratory**	To explore alternate measures of viral shedding.	e.g. Quantitation and detection of virus in exhaled breath using facemask insert technology; other methods of live virus detection; virus sequencing; antigen detection via CE marked lateral flow tests
	Other exploratory immune endpoints	E.g. HLA and whole genome sequencing<br>Cardiac MRI exploratory research images such as ECV

	Sample analysis for the completion of exploratory endpoints may be performed under the OVC Biobank research tissue bank protocol (REC: 16/SC/0141)
**Challenge agent**	SARS-CoV-2
**Formulation**	Liquid
**Route of Administration**	Intranasal administration
**Dose per Administration**	$1x10^1$ to $1x10^5$ $TCID_{50}$
**Rescue therapy:**	Paxlovid (300mg Nirmatrelvir with 100mg ritonavir) taken twice orally daily for 5 days[1] or<br>1200mg intravenous infusion of REGN COV2 (A monoclonal antibody cocktail against the SARS-CoV-2 spike protein)<br><br>1. Once paxlovid is fully approved following SA007, REGN COV2 will be discontinued as rescue therapy

SUMMARY

In December, 2019, a local outbreak of pneumonia of initially unknown cause was detected in Wuhan (Hubei, China), and was quickly determined to be caused by a novel coronavirus, namely severe acute respiratory syndrome coronavirus 2 (SARS-CoV-2). Since then, the virus has made its way across the globe to affect over 180 countries and was declared a pandemic by the World Health Organisation on 11 March 2020. As of 9th November 2022, there have been over 20 million confirmed COVID-19 cases and over 170,000 deaths in the United Kingdom[10].

Understanding the nature, effectiveness and durability of the human immune response to SARS-CoV-2 is crucial for the long-term management of the disease. At present, we have some knowledge of the pattern and kinetics of the humoral response to natural infection from epidemiological studies but are less certain about all other aspects of the response including the nature of the T cell response, the extent of existing immunity in the population to SARS-CoV-2, the role of innate immunity, the extent of memory B cell creation, the durability of all these elements of protection and how these correlates of immunity change after vaccination.

The primary purpose of this study is to evaluate the safety and human clinical response to SARS-CoV-2 challenge in individuals who are not considered immunologically naïve to SARS-CoV-2. This will be divided into 2 groups; a) individuals with previous SARS-CoV-2 infection (+/- vaccination) or b) individuals with no prior history of SARS-CoV-2 infection who have received vaccination against SARS-CoV-2. We start by establishing the optimal challenge dose that causes infection in both groups. The study hopes to identify the lowest level of infectious dose necessary to produce viral replication in the upper respiratory tract of research volunteers while minimising risk of disease progression. Investigators aim to achieve an infection model which results in no symptoms, or symptoms no more severe than the common mild response of healthy people of the same age within the general population.

Demonstration of a safe SARS-CoV-2 human challenge model in immunologically sensitised individuals will then allow its use, to study in detail how baseline immunity, prior to infectious challenge, confers protection from infection. By comprehensively characterising the baseline immune response in healthy subjects who have either previously been infected with SARS-CoV-2 or received a vaccine against SARS-CoV-2, and then determining infection rates after challenge, we can establish which baseline immune responses, induced by prior infection and/or vaccination, protect against subsequent infection.

We can also use quantitative viral load data post-infectious challenge to further define immune correlates of protection. We expect the use of young, healthy and previously infected and/or vaccinated individuals to demonstrate the most robust immune responses providing the best opportunity to identify potential correlates of protection. Identification of such correlates has for other pathogens allowed targeted vaccine and therapeutic development as well as providing immunological efficacy end points allowing prioritisation of new therapeutic candidates.

A successful SARS-CoV-2 human challenge model may be utilised in the future to allow assessment of new therapeutics. A safe model needs to account for the dynamic nature of protection seen in the target population. This includes a range of immunological phenotypes including those who have previously been infected with SARS-CoV-2 (with or without vaccination) and those who are uninfected (with or without vaccination). Uninfected, unvaccinated volunteers are not included in this study as this sub-population is included in an ongoing, parallel study (NCT04865237). The addition of an uninfected

vaccinated group to this study will allow us to compare and contrast both the dose required for infection and the immune response in these groups. Natural infection exposes individuals to a broader range of viral epitopes than vaccination. Furthermore, the route of natural infection may induce mucosal immunity that intramuscular vaccination may not. Challenge of vaccinated individuals in a controlled human infection model also provides the opportunity to assess both the dynamic range and duration of protective immunity post vaccination.

We can also use this controlled human infection model to define precisely the innate and adaptive immune response after infection in a way that is not feasible in natural infection, where timing infection precisely is not possible. Challenge models have the added benefit over epidemiological studies of allowing detailed investigation of the immune response after a defined time-point infection allowing a greater narrative on both viral kinetics and the nature of protective and non-protective immune responses. The early innate immune response is thought to play a vital role in determining disease phenotype and can be difficult to characterise with both asymptomatic and pre-symptomatic infection often missed in epidemiological studies.

The information gained from these studies can also inform policy makers on issues, such as determining whether to allow return to unfettered travel and normal daily activities, as well as information to determine the need and timing of vaccination and re-vaccination in the context of previous infection and vaccination. We will define viral shedding in the breath in both asymptomatic and mildly symptomatic patients, better informing Public Health infection control policy. Furthermore, with the recent identification of new variants that escape targeted treatments such as monoclonal antibody therapy and convalescent plasma, establishing a safe SARS-CoV-2 CHIM using wildtype virus is a necessary first step prior to future studies using new variants in individuals who are seropositive either from natural infection with different lineages of the virus or vaccination [11]. This could be an essential step in speeding the development pipeline of novel vaccines and therapeutics against new variants.

2 ABBREVIATIONS

**ACE2**	Angiotensin-converting enzyme 2
**ADE**	Antibody dependent enhancement
**AE**	Adverse event
**ALT**	Alanine Aminotransferase
**ALP**	Alkaline Phosphatase
**APTT**	Activated partial thromboplastin time
**ARDS**	Acute Respiratory Distress Syndrome
**BAME**	Black, Asian and Minority Ethnic
**BCC**	Basal cell carcinoma
**BMI**	Body Mass Index
**CCVTM**	Centre for Clinical Vaccinology and Tropical Medicine, Oxford
**CHIM**	Controlled human infection model
**CI**	Chief Investigator
**CIS**	Carcinoma in situ
**CMR**	Cardiac magnetic resonance imaging
**COVID-19**	Coronavirus disease 2019

**CRF**	Case report form
**CT**	Computerised Tomography
**CSP**	Clinical Study Plan
**DLCO/TLCO**	Diffusing capacity of lung for carbon monoxide (synonymous with TLCO)
**DSMB**	Data Safety Monitoring Board
**ECG**	Electrocardiogram
**EUA**	Emergency use authorisation
**ELISA**	Enzyme-linked immunosorbent assay
**ELISPOT**	Enzyme-linked immunospot
**EMCRF**	Experimental Medicine Clinical Research Facility
**FDA**	Food and Drug administration, United States medical regulators
**GCP**	Good Clinical Practice
**GMP**	Good Manufacturing Practices
**GP**	General Practitioner
**HCG**	Human chorionic gonadotrophin
**HBV**	Hepatitis B virus
**HCV**	Hepatitis C virus
**HIV**	Human immunodeficiency virus
**HLA**	Human leukocyte antigen
**HRA**	Health Research Authority
**ICS**	Intracellular Cytokine Staining
**ICL**	Imperial College London
**IFNγ**	Interferon gamma
**IV**	Intravenous
**LMWH**	Low molecular weight heparin
**mAbs**	Monoclonal Antibodies
**MHRA**	Medicines and Healthcare products Regulatory Agency
**NHS**	National Health Service
**NHP**	Non-human primate
**PBMC**	Peripheral blood mononuclear cell
**PCR**	Polymerase chain reaction
**PI**	Principal investigator
**PHE**	Public Health England (now UK Health Security Agency)
**PFTs**	Pulmonary function tests
**QP**	Qualified Person
**RBD**	Receptor binding domain
**REC**	Research Ethics Committee
**RGEA**	Research Governance, Ethics and Assurance, Oxford University
**RNA**	Ribonucleic acid
**SAE**	Serious adverse event
**SARS-CoV/ SARS-CoV-1**	Severe acute respiratory syndrome coronavirus
**SARS-CoV-2**	Severe acute respiratory syndrome coronavirus 2
**SAM™**	synthetic absorptive matrix

**SGTF**	S gene target failure
**SmPc**	Summary of product characteristics
**SoA**	Schedule of Attendances
**SOP**	Standard operating procedure
**SUSAR**	Suspected unexpected serious adverse reaction
**TB**	Tuberculosis
**TCID50**	Median Tissue Culture Infectious Dose
**TEDS**	Thrombo-embolus deterrent stockings
**UK HSA**	UK Health Security Agency
**URT**	Upper respiratory tract
**vp**	Viral particle
**WHO**	World Health Organisation
**WOCBP**	Women of Child Bearing Potential

3 BACKGROUND AND RATIONALE

Understanding the nature, effectiveness and durability of the human immune response to SARS-CoV-2 is crucial for the long-term management of the disease. Currently information is lacking around almost every aspect of the immune response following a SARS-CoV-2 infection. We are unable to establish with certainty whether an individual with a particular titre of antibody from prior infection and/or vaccination is likely to be protected from infection and, if so, for how long. Similarly, we cannot quantitate the level of protection being provided by T cell immunity. These are central questions for establishing evidence-based government policy on issues such as immunity passports and the need and timing of vaccination and re-vaccination in the context of previous infection or vaccination.

3.1 Background

There are several approaches to addressing these challenges and each provides distinct insight into these questions. The first approach is to use epidemiological data to establish whether individuals such as hospital workers or care home workers who have become seropositive because of infection/ vaccination are observed to become subsequently infected. One such study (SIREN study) has recently published an interim analysis demonstrating that seropositive healthcare workers from prior infection had an 83% lower risk of infection, with a median protective effect five months following primary SARS-CoV-2 infection[12, 13]. A similar study observing infection in care home residents and staff, the VIVALDI study is ongoing. These are crucial studies but will not be able to provide definitive correlates of immunity as they will provide little data on the state of the immune system at the precise time of infection; in addition, these studies are slow as they require high enough levels of endemic infection in the target population to generate the numbers necessary to draw scientifically robust conclusions. The asymptomatic nature of the disease means that identifying re-infected people may prove to be extremely challenging. Furthermore, recent licensure and roll out of first-generation vaccines against SARS-CoV-2 will lead to a natural decline in infection levels meaning field efficacy models will become increasingly difficult to undertake. A second type of study using human challenge will provide a more detailed understanding of

the nature of the immune response that provides protection from infection and thus will be very complementary to the epidemiological studies described above. It will provide the opportunity to interrogate the full extent of the immune response at the time of exposure and will also allow the evaluation of the durability of immune responses of all kinds and how they correlate with protection. It has the advantage that it can provide rapid results and rapid turnaround as well as a much more detailed evaluation of immune function. This proposal is to institute such a challenge model in the UK to establish the role of immunity in protection against infection, but also to provide a longer-term capacity to test other questions that will arise largely relating to immune response durability as the epidemic continues. These include defining correlates that could explain the difference in risk of severe disease in certain populations (BAME patients, elderly and those with comorbidities). Similar studies will also enable the evaluation of the longer-term effects of vaccine candidates on protection and immunity as they are developed. A successful SARS-CoV-2 human infection model could also be used to accelerate development of next generation vaccines and novel treatments. This remains necessary to prepare for future coronavirus outbreaks, due to the emergence of new strains or SARS-CoV-2 mutations. Improved vaccines are still necessary to help block transmission, optimally protect at-risk groups and provide cross-strain protection with virus mutation.

3.2 Why is the COVID-19 challenge model so important?

There are many aspects of COVID-19 infection that scientists still don't understand. The human challenge study may be the only way to get this information. We believe that the information obtained in the challenge study will have important public health benefits and play a significant role in the ongoing management of the pandemic.

1. The "incubation period" of COVID-19 is the time from initial coronavirus exposure to the onset of symptoms and when people are most infectious. A human challenge study is the only way to accurately measure how long the incubation period is, and will assist and inform future track and trace efforts.
2. A human challenge study will allow us to accurately measure how long people are infectious, from first exposure to the virus being cleared. This will determine exactly how long self-isolation (quarantine) periods should be after exposure.
3. Re-infection means a person was infected once, recovered fully, and then later became infected for a second time. Based on what we know from similar viruses, some people could catch COVID-19 more than once. A human challenge study will help answer a number of important questions on re-infection, including how long natural immunity can protect people from re-infection; what makes people more at risk of re-infection; and whether re-infected people can spread the virus to others.
4. Emerging data suggests it is possible to become infected despite vaccination against SARS-CoV-2. Challenge of vaccinated individuals will allow us to assess whether induced immunity can protect individuals from infection, what makes people more at risk of infection; and whether infected people can spread the virus to others.

5. A human challenge model will allow us to track the immune response (mucosal and systemic) from a defined time point of viral exposure, this is very difficult to do in epidemiological studies.
6. Developing a safe human challenge study will enable future studies to allow testing of vaccines and antiviral treatments, particularly against new variants. A key question for new vaccines is whether they stop people carrying and spreading the virus as well as preventing symptoms.
7. A re-infection human challenge model can also enable identification of functional correlates of protection and enable roll out of robust tests which confirm protection in individuals
8. Many people infected with coronavirus are asymptomatic. Asymptomatic individuals are a major cause of infection spread because they are unaware they are infected. A human challenge study will allow researchers to learn much more about asymptomatic re-infection and risk of transmission.

The aim of this study is to firstly define the best dose for such a challenge model and secondly to describe viral kinetics and correlates of protection in individuals primed by either prior infection and/or SARS-CoV-2 vaccination. This study is being conducted in parallel with a dose finding challenge model of SARS-CoV-2 naïve volunteers at Imperial College London, UK

3.3 How is this research relevant to patients?

While young healthy adults may not fully recapitulate high risk groups, they provide a benchmark for optimal protective immunity and are highly suitable for antiviral and monoclonal antibody testing. People in this age group are currently the main drivers of the continuing pandemic transmission now older adults are vaccinated and will be the main target for vaccines that aim to prevent asymptomatic transmission by reduction of viral shedding in the upper respiratory tract.

3.4 Immune response to SARS-CoV-2

More than 90% of SARS-CoV-2 infections result in production of antibody by 7 days. The durability of this response seems variable with anti-spike S IgG remaining stable up to 6 months but anti-nucleocapsid N antibody falling from 1 month post infection [14]. A shorter anti-N protein half-life is associated with younger adults and asymptomatic infection.

Recent evidence from animal models showed that antibodies against spike protein administered passively protect against subsequent challenge with SARS-CoV-2 virus [15]. This is especially true when the antibodies are neutralising *in vitro*. Such antibody protects cells from infection by blocking the interaction between the spike receptor binding domain and the ACE2 receptor. However, other antibodies that bind to spike protein but did not neutralise *in vitro* may fail to protect against infection. The success of the passively transferred antibodies in animal models suggests that antibody prophylaxis (and possibly, therapy) either with convalescent plasma or with cloned antibodies could be protective in humans. The optimal therapy might be with polyclonal antibody, since single antibodies might drive virus escape.

Most people make a polyclonal response after virus infection that targets many different proteins and epitopes within them [16].Thus, it might be expected that most people will make neutralising antibodies that are protective against re-infection. However, the specific protective (neutralising) antibodies are not measured in all the different antibody tests that have been developed so far. For example, some tests measure antibodies against N protein only, which while a marker of infection, is not thought to confer protection. Some of the latest serological tests do measure antibodies that target the receptor binding domain specifically, or even measure the antibody's ability to disrupt the interaction between spike and ACE2 receptor.

However, it remains uncertain what titre of neutralising antibody is required for immunity; it is also uncertain how long antibody persists and how effectively it is boosted by re-exposure or vaccination. We also know little about the size of memory B cell populations in convalescent patients. In addition, antibody in the blood may not be reflected by protection against lung disease (although this seems probable) or against infection of the nose and pharynx (less likely).

There is emerging data to suggest that T cells also play an important role in the response to this virus and both CD4 and CD8 T cells specific for viral epitopes are known to be present in individuals who have not had COVID-19, as well as in those who have had all forms of the disease, from asymptomatic infection to moderate or severe disease. Evidence is building to suggest that T cells may form a crucial part of protective immunity against the virus and that widespread T cell immunity may have been present in the population through cross reactivity to other coronaviruses before the pandemic occurred. Observations that agammaglobulinemic patients may not succumb to severe disease supports this theory [17]. Long term immunological studies of individuals infected with SARS-CoV-1 suggest that while serological immunity is short lived T cell immunity may last many years. However, it is not clear whether such immunity provides protection against re-infection with that pathogen or related viruses. It also appears that family members of affected individuals who have not seroconverted may have measurable T cell responses. The study of T cell responses has lagged behind serological studies largely because assays of T cell function are difficult, not standardised and not scalable. Solutions to this problem are currently being generated by groups worldwide. Additionally, it is clear that SARS-CoV-2 also has the ability to evade innate immune responses. For example, it is very effective at suppressing and avoiding interferon responses, likely increasing its pathogenicity.

Our knowledge gaps on issues of protective immunity and its correlates has serious repercussions with regard to policy decisions. We hope that furthering our understanding will inform development of optimal vaccines and enable provision of clear guidance to the public and front-line workers about protection gained from prior infection or vaccination and durability of protection. Insights into protection may arise by following populations epidemiologically over a long period, as has been done with other respiratory viruses but there is some urgency to obtain answers in the setting of a pandemic given the seriousness of the disease in certain individuals and the profound impact it is having on our healthcare system, economy and lifestyle. Accelerated and definitive answers can be obtained using a carefully developed human infection model.

The key immunological questions that might be resolved by human challenge therefore include:

- The protection afforded by current or new vaccines
- The role of antibody vs. cellular immunity in immune protection and disease
- The immune biomarkers that predict variations in symptoms
- The durability of immunity in those previously infected with SARS-CoV-2
- The effects of passive antibody therapy on viral infection and recovery

As our model includes volunteers who are seropositive from natural infection only and also volunteers who are seropositive from natural infection and previous vaccination, once developed, this challenge model could be used for future studies to provide information on durability of vaccine immunity, efficacy of vaccines against new variants and interrogation of the role of immunity directed against non-spike antigens.

3.5 Rationale for addition of groups 3 & 4 (previously uninfected, vaccinated participants)

The magnitude, duration and breadth of the immune response seen against SARS-CoV-2 inoculation will vary between previously infected and uninfected individuals. Those individuals with prior infection will have been exposed to a broader range of viral epitopes than uninfected vaccinated volunteers in a UK population, the majority of whom have received a homologous prime-boost regime with vaccines containing the SARS-CoV-2 spike protein. Current evidence suggests that vaccination may confer a neutralising response of greater magnitude than unvaccinated convalescent individuals; with a phase 1/ 2 trial of BNT162B2 vaccine demonstrating higher neutralising antibody titres following both primary and boost doses compared to convalescent human sera[18]. However, there is increasing evidence that those who have been infected prior to vaccination can mount stronger immune responses than uninfected double vaccinated individuals[19-23]. Compartment specific immune responses may also differ between those who have been naturally exposed to respiratory droplets and uninfected individuals who have received an intramuscular vaccine. Sterlin et al, demonstrated that mucosal IgA contributed to a greater extent to early SARS-CoV-2 viral neutralisation than serum IgG[24]. Addition of uninfected vaccinated volunteers to the study will enable us to do several things:

1. Development of a SARS-CoV-2 challenge model more representative of the full repertoire of prior sensitisation to SARS-CoV-2 likely to be found in the real world. If future models, are to be used to test the efficacy of novel vaccines or therapeutics ensuring that an appropriate dose is selected that covers this breadth of prior immunity is important.
2. Ability to compare and contrast the immune response (both systemic and mucosal) and viral kinetics of infection in those with prior sensitisation from natural infection versus vaccination. The benefit of doing this in a challenge model is the ability to control other confounding variables such as time of infection, dose of virus and variant of SARS-CoV-2.
3. Establishment of this model would allow future assessment of vaccines against new variants to assess virus escape, and also the assessment of durability of protective immunity conferred by vaccination.
4. Interrogation of the differing immune response (mucosal and systemic) seen in convalescent vaccinated individuals and uninfected vaccinated individuals may help to facilitate and focus next generation vaccine development.

Dose escalation for the uninfected vaccinated group (group 3) will commence at a dose of $1x10^2$ TCID50 based on data collected from Group 1a, demonstrating that a target attack rate of 50% (+/-10%) in unvaccinated anti-Spike IgG positive individuals (from prior infection) was not achievable at a dose of $1x10^1$ TCID50 and with no safety concerns identified.

3.6 Research Strategy

The two endpoints in a human challenge model which could provide hard evidence in evaluation of vaccines and other interventions are (i) virological (i.e. quantified evidence of infection of the participant) and (ii) disease-based (i.e. a measure of symptom intensity in the participant). A significant proportion of SARS-CoV-2 infections in nature are asymptomatic, and at the present time the probability of disease given infection, and the host and microbial factors which determine progression from infection to disease are not defined. In view of this, and the wide range of symptoms and potential morbidity/mortality associated with the development of more severe disease, measurement of viral concentrations within the upper respiratory tract (URT) would provide the safest and most easily defined endpoint, rather than a measured symptom/disease-based endpoint. Therefore, the current design is a dose titration study to identify the dose level that is safe and well tolerated while resulting in reproducible levels of SARS-CoV-2 viral infection in the URT of inoculated participants, irrespective of symptoms.

This dose escalation in previously SARS-CoV-2 infected volunteers will be conducted in parallel with a dose escalation in SARS-CoV-2 naive volunteers that will be conducted by hVIVO, in close collaboration with Imperial College London (ICL); Chief Investigator Dr Christopher Chiu. The aim of the hVIVO/ICL study is to establish a SARS-CoV-2 controlled human infection model for vaccine evaluation in SARS-CoV-2 naïve healthy volunteers. The aim of our complementary work, is to establish a SARS-CoV-2 infection model to evaluate key questions about protective immunity. Using the same virological strain in both vaccine and protective immunity studies, together with common virological and adverse event endpoints, will allow us to bridge between the two studies and maximise knowledge gained.

Alongside these challenge studies the crucial scientific studies will be immunological assays that define the extent and strength of immune reactivity so that these can be correlated to the presence of protection against challenge infection. Serological assessment will be performed with a combination of ELISAs to a range of viral proteins and associated assays of neutralising antibodies. Quantitation of memory B cell populations will assess the ability of individuals with serological evidence of immunity to respond on further exposure. T cell assays will be systematically performed using established techniques including ELISpots, intracellular cytokine staining and RNA sequencing. Cell activation markers will provide further insights into T cell populations. Collection of epithelial lining fluid with enable description of cytokine responses relating to innate, cellular and humoral immunity at the local level.

Careful participant selection associated with extensive immunophenotyping and serological assays in this challenge context should provide definitive answers to some of the most important scientific questions facing policy makers in this pandemic.

3.7 Virology of SARS-CoV-2

3.7.1 Virus structure and genetics

SARS-CoV-2 is an enveloped positive sense single-stranded RNA virus with a genome of approximately 29,000 nucleotides in length. SARS-CoV-2, along with SARS-CoV-1 (the cause of SARS in 2002-3), are members of the genus *Betacoronavirus,* subgenus *Sarbecovirus*. SARS-CoV-2 shows 79% nucleotide similarity with SARS-CoV-1, but is suggested to be most closely related to the horseshoe bat Sarbecovirus, RaTG13, having diverged from this around 40-70 years ago.

3.7.2 How SARS-CoV-2 infects humans

It is likely that SARS-CoV-2 has been circulating in bats and intermediate species (possibly the pangolin) before crossing into humans in Hubei province of China in late 2019. SARS-CoV-2 enters a human host cell by binding to the human angiotensin-converting enzyme 2 (ACE2) receptor with the viral spike (S) protein. The spike receptor binding domain (RBD), which is the specific domain within the S protein that is responsible for binding to the human ACE2 receptor, is more divergent from RaTG13 (85% nucleotide similarity). Furthermore, compared to RaTG13, SARS-CoV-2 has also acquired a furin (human protease enzymatic activity which is exploited by numerous viral and bacterial pathogens) cleavage site insertion within the viral envelope (E) protein, which may have led to an increase in human infectivity.

3.7.3 Role of inoculum density in severity of infection

It is intuitive that the concentration of virus to which a participant is exposed is likely to determine the chances of successful infection, but also of severity of the inflammatory response and, potentially, the disease. We are not aware of relevant infecting dose experiments with animal models of COVID-19, but animal models of other viral infections show that variation in the infecting dose determines the severity of the disease. A dose-response has been shown in mouse models with several strains of SARS-CoV-1, which is closely related to the virus causing COVID-19 (SARS-CoV-2). The infectivity varies between different strains of the virus, which modifies the shape of the dose-response curve, but nevertheless consistent dose-response relations are observed with the severity of the infection [25]. Human influenza infection challenge studies have also demonstrated higher infection rates related to dose [26]. In this study, we will therefore adopt a strategy of infecting participants with a low dose of SARS-CoV-2, and then conduct a series of dose increment cycles until we identify the lowest dose which achieves the objective of viral replication in the upper respiratory tract of 50% (+/-10%) of participants.

3.7.4 Significance of high viral loads within the upper airway

Longitudinal studies have not established any correlation between SARS-CoV-2 viral load in the respiratory tract and disease severity in younger patients due to the high frequency of asymptomatic disease. In

symptomatic hospitalised patients, the highest levels of pharyngeal virus shedding occur during the first week of symptoms, with a peak on day 4 [27].

Transient peak viral loads of $7x10^8$ RNA copies/swab have been reported in upper and lower respiratory tract samples but decline rapidly after day 5. Shedding of viral RNA from respiratory tract samples generally outlast the end of symptoms. Viral loads above $log10^7$ RNA copies/mL samples have been independently associated with detection of infectious SARS-CoV-2. In hospitalised patients with mild-severe disease, the median duration of infectious virus shedding is 8 days [28]. Patients with <log 10^6 viral RNA copies/mL are highly unlikely to be infectious [27, 28].

3.7.5 Mutations and viral lineages of SARS-CoV-2

Since January 2020, SARS-CoV-2 has spread globally. Despite a relatively low mutation rate of around 2.5 mutations/genome/month (similar to other coronaviruses), the rapid global expansion has resulted in the accumulation of over 2,000 known mutations within the viral genome. The significance of these mutations to pathogenesis and transmission is still poorly understood. The vast majority of mutations are felt to have no apparent effect on the virus. Ongoing SARS-CoV-2 genome sequencing is being undertaken by the COVID-19 Genomics UK (COG-UK) consortium. This is critical to our understanding of the diversity of the virus and will inform vaccine development. Distinct viral lineages associated with geographical regions have emerged and been classified into viral genotypes. These genotypes will help us better describe how the virus is spreading both locally and globally.

In December 2020, PHE announced lineage B1.1.7 as the first "Variant of Concern" (VOC-202012/01). This variant is notable due to a higher number of mutations in one lineage and is responsible for a growing number of cases in the UK. It is defined by a set of 23 mutations, 14 amino acid changes and 3 deletions. The N501Y mutation, known to be in this variant, affects the receptor binding motif (RBM) of the spike protein, increasing binding affinity to the ACE-2 receptor. Ongoing studies suggest that this variant transmits more readily but it is unclear at point of writing if this is associated with any change in virulence or significant antigenicity changes[29]. One of the observed S gene mutations in this lineage deletes amino acids 69 and 70 (Δ69-70), this mutation has been found to cause a reproducible S gene target failure (SGTF) in the ThermoFisher TaqPath assay used in 3 of the UK lighthouse laboratories that provide SARS-CoV-2 testing for approximately 35% of community tests in England. This coincidental occurrence can be used as a proxy measure for incidence of lineage B1.1.7. SGTF has been compared to sequencing of PCR results and demonstrated that as of 14th December 2020 lineage B1.1.7 has been responsible for over 99% of SGTF [30]. In the tests performed by these laboratories, the incidence of SGTF suggestive of lineage B1.1.7 has been steadily rising from 28.2% for week commencing 30/11/2020 to 89.5% (from 75,092 tests) week commencing 18th January 2021. The proportion of cases tested by these labs per region is demonstrated in figure 1 below.

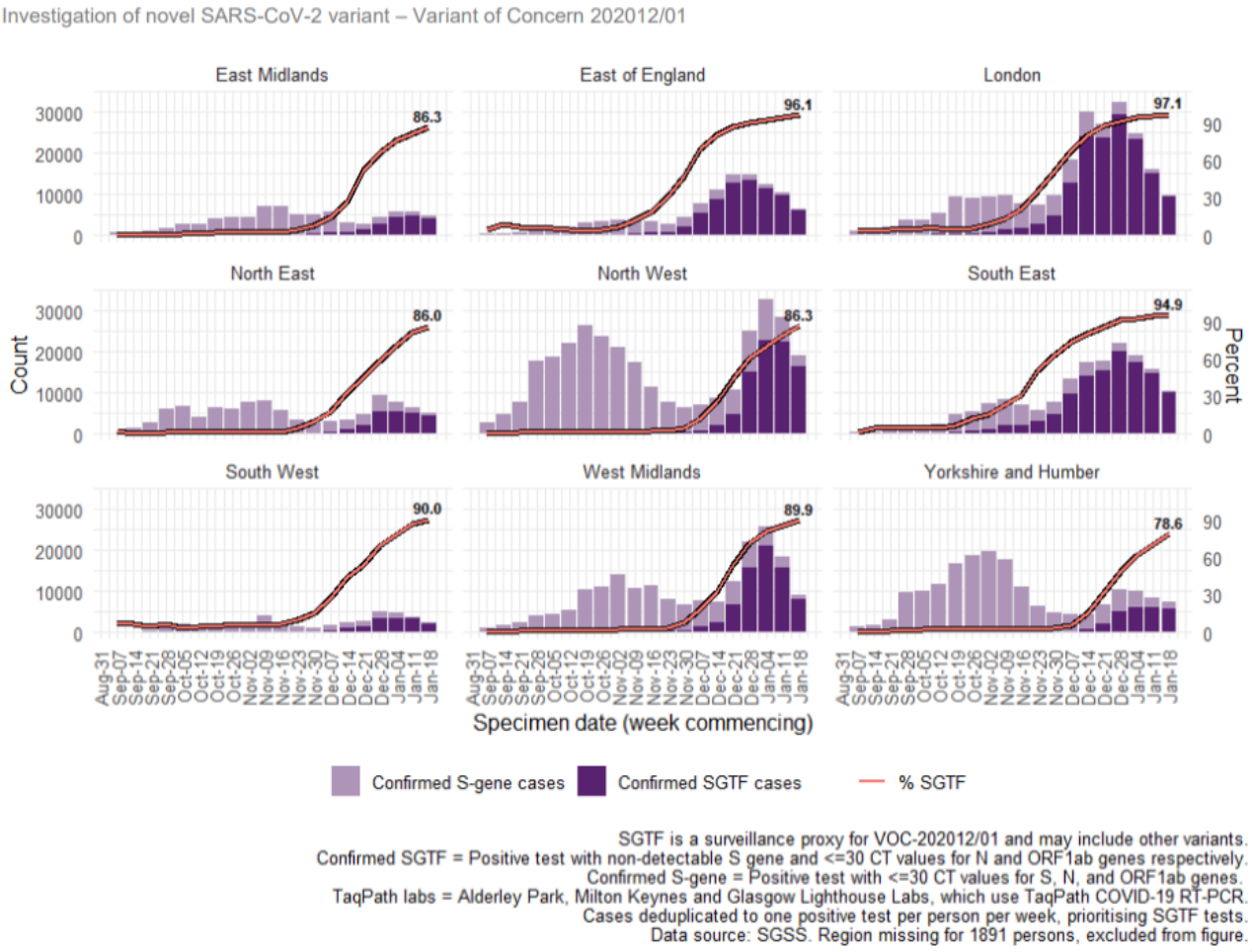

Figure 1: Weekly number (bars) and proportion (red lines) of Pillar 2 COVID-19 cases tested by TaqPath laboratories with SGTF among those with S gene detection results, by region of residence (7 September 2020 to 24 January 2021). *Percent confirmed SGTF for most recent days annotated.[30]*

Other noteworthy global variants of interest include lineage B.1.351 (N501Y.V2) and lineage P.1. Lineage B.1.351, first detected in South Africa, has eight mutations in the spike protein (three in the RBD including N501Y). It is associated with a high number of South African infections and has been shown to have increased transmissibility. Of particular concern, two RBD mutations (K417N, E484K) have been shown to escape monoclonal antibody therapy.[11] Lineage B.1.351 has subsequently been sequenced in samples from several other countries including the UK. Lineage P.1 first detected in Brazil, has also recently been sequenced in a small number of UK samples. This lineage also contains mutations N501Y, K417N and E484K[29].

Of recent concern, the spike protein mutation E484K has also been found in 11 B1.1.7 (UK variant of concern) sequences (of 214,159 analysed) by COG-UK on 26/01/2021 [30].

The emergence of multiple variants with shared mutations in the spike protein raises the concern of the evolution of SARS-CoV-2 to a new phenotype with increased transmissibility. It is not yet fully known

whether this would have the propensity to cause re-infection or infection in vaccinated individuals. A South African group has demonstrated lineage B.1.351 escapes convalescent plasma and monoclonal antibody therapy[11]. Full data is still awaited regarding efficacy of vaccines, however a series of recent press releases and pre-prints have suggested efficacy of licenced and several unlicensed vaccines will remain comparable for wildtype SARS CoV-2 and the UK variant lineage B1.1.7 [31-35]. For lineage B1.351 (South African variant) in vitro studies of Pfizer and Moderna vaccines suggest reduced neutralising antibody titres in vitro against this variant, although still felt to be present at levels that would offer some protection[32, 33]. A recombinant spike protein nanoparticle vaccine candidate NVX-CoV2373 found that efficacy was markedly reduced in their South African study compared to UK data [31]. Post hoc analysis of their UK study demonstrated efficacy of 95.6% for wildtype SARS CoV-2, 85.6% against UK variant B1.1.7, whilst the South African study demonstrated 60% efficacy (CI 19.9-80.1), where the mutated lineage B.1.351 was responsible for over 90% of cases the trial sequenced. Similarly, Johnson & Johnson found reduced efficacy in their South African trial (72% protection in USA, 57% in South Africa) and the Oxford-AstraZeneca vaccine has shown no protection against mild and moderate disease caused by lineage B.1.351 in a small trial of young, healthy volunteers [34, 36]. Efficacy against severe disease from lineage B.1.351, including mortality and hospitalisations for the Oxford-AstraZeneca vaccine is not yet known. In light of these recent findings, multiple vaccine producers are already working on new vaccines or boosters against lineage B.1.351.

Although, it is positive that partial efficacy against lineages with significant spike protein mutations has been demonstrated, it does raise concerns that immunity from prior infection or vaccination will not always confer protection against known or future mutations. Therefore, any mutations to the spike protein need to be carefully monitored. The ability to rapidly test immunity to new variants in seropositive individuals (either from prior infection with different lineages or vaccination) would help guide government policy moving forward. By demonstrating the feasibility and safety of a SARS-CoV-2 challenge using an early SARS-CoV-2 isolate, this model could be used for new variants in the future, allowing rapid understanding of the immune response and assessment of vaccines and therapeutics. It is accepted that translation of this model for use in new variants would have some limitations, including possible need for limited dose escalation studies. However, a proof of concept study using the early isolate, for which we have significantly more clinical data is an important step in this process.

3.8 Virological Correlates of Infection

Figure 2 shows SARS-CoV-2 viral load in the upper respiratory tracts of naturally infected symptomatic patients by qPCR and culture [28]. On infection recovery, many people have viral RNA (detected by swab PCR test on a nasal sample) but do not shed *live* virus that can be grown in a lab or infect others. In a review of 79 studies on SARS-CoV-2, viral RNA shedding from the upper respiratory tract peaked in the first week from symptom onset, with a mean total shedding of 17 days and a maximum at 83 days [37]. However there was no live virus detected after day 9 of illness in any study in immunocompetent people (11 studies). This loss of viral infectivity (live virus) coincides with a declining viral load (detected by PCR) and the appearance of antibody [28].

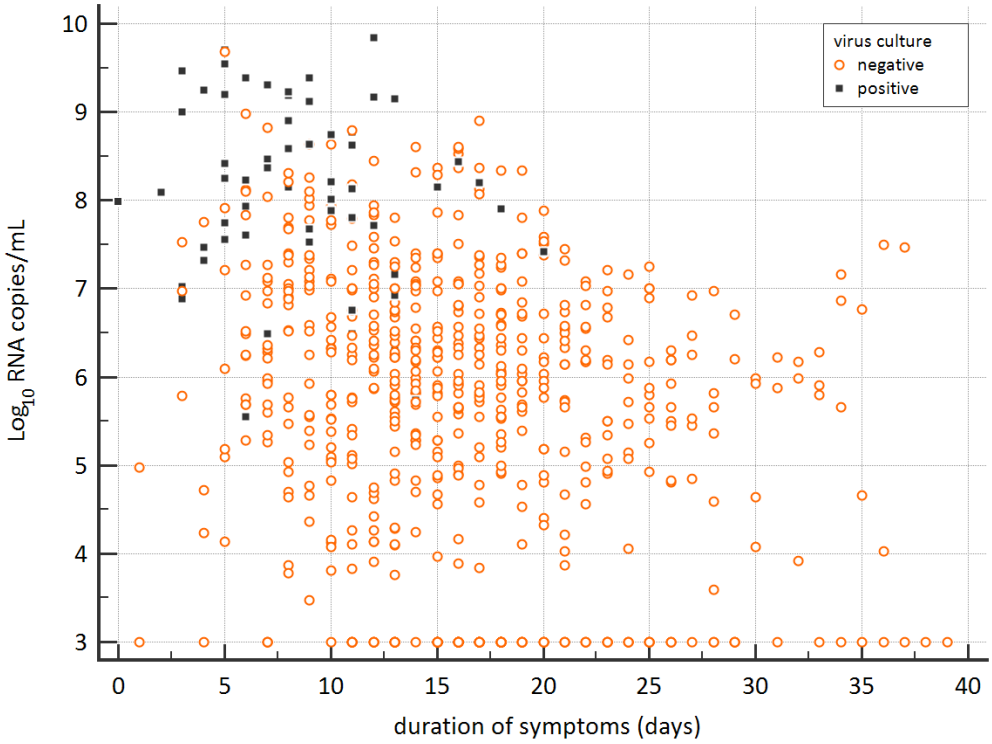

Figure 2 SARS-CoV-2 viral load in naturally infected patients by qPCR and culture.

The Scientific Advisory Group for Emergencies (SAGE) has been advised by NERVTAG that it is unlikely that people who are recovering from SARS-CoV-2 infection and have developed antibody in nasal secretions, blood or serum are still infectious. A qPCR Ct value >35 has been presented to NERVTAG committee as associated with lack of infectivity (Wendy Barclay and Peter Openshaw, Imperial College London, personal communication).

Viral particles can spread from human to human either through surface contamination (by nose/mouth touch or large respiratory droplet deposition) or through breathing in contaminated aerosolised droplets. For aerosol transmission the virus needs to be viable in droplets of <5um to remain suspended in the air. Despite best efforts, it remains unclear how the SARS-CoV-2 virus is spread. While wearing face masks seems to mitigate infection risk, suggesting an aerosol droplet route, this remains controversial[38]. Hospital observational studies and experimental modelling studies have detected viable SARS-CoV-2 in the air for 3 hours and on surfaces for up to 3 weeks [39, 40] , suggesting potential for spread via both mechanisms. In one study, aerosols of <5um were generated with a jet nebuliser resulting in $10^{3.7}$ $TCID_{50}$ per litre of air. The virus remained viable until their 3 hour endpoint and reduced to a concentration of $10^{2.7}TCID_{50}$ per litre of air [39]. It remains unclear what the correlation is between environment contamination and airway viral load of the patient (as measured by viral shedding on nasal swab). Furthermore, exhaled virus from an asymptomatic person may be more or less viable than virus expelled on coughing from a symptomatic patient. Our proposed human re-infection challenge model, where we will track viral kinetics from both oropharyngeal & mid turbinate swab and breathed out virus collected

on face mask (experimental endpoint) is well placed to answer many of these questions. This could inform hospital infection control strategy and public health policies such as quarantine length.

3.9 Re-infection

Following SARS-CoV-2 infection, an immunological response is demonstrated, but despite extensive studies, there is still limited evidence about the type and level of immune response that protects people from re-infection. However, with the efficacy of first-generation vaccines and the number of documented re-infections remaining low despite high incidence rates in some countries, it is clear that immunity after vaccination or natural infection can provide resistance to re-infection, although the duration of protection remains unknown. In a recent multi centre cohort study of UK healthcare workers(SIREN), possible re-infection was detected in 44 people out of a total of 6614 participants, indicating a possible albeit low risk of re-infection (odds ratio 0.17 (95% CI 0.13-0.24) re-infection risk compared with primary infection). Median time to re-infection was more than 160 days [12, 13]. Re-infection was defined as greater than 90 days from primary infection and the authors do acknowledge that some of these possibly infected cases could be primary infection with long term viral shedding as genomic sequencing data is lacking. A further study of 133266 laboratory confirmed cases in Qatar, 243 patients were identified who had had a previous positive PCR more than 6 weeks earlier[41]. Without genomic sequencing it is unclear if all were true re-infections, however 54 cases were deemed likely re-infection due to clinical context. In a SARS-CoV-2 serology study of 12219 UK health care workers, there were no symptomatic infections in those with anti-spike antibodies, (compared with 89 in seronegative participants). However, there were three asymptomatic infections captured in the seropositive cohort[42]. In the SIREN study 40 of the 44 re-infected cases had positive antibodies at the time of re-infection[12]. Taken together, this data suggests that while anti-spike antibodies do confer protection for a period of time in a majority, re-infection can occur despite seropositivity. Why some people remain unprotected following primary infection is unknown. Various theories have been postulated including increased risk following asymptomatic or mild primary infection, length of time since primary infection or inherent variability in immune responses. In a re-infection challenge study in rhesus macaques, an intratracheal dose of SARS-CoV-2 at 1×10^6 $TCID_{50}$ was not sufficient to re-infect these animals 28 days following initial infection [43]. However as our volunteers will have had their primary infection more than 3 month prior to re-infection, the applicability of these findings to the human re-infection model is uncertain. Our study is designed to escalate dose until re-infection occurs in 50% (+/-10%) volunteers. Baseline humoral and cellular immunology of volunteers who remain resistant to re-infection will provide important insight into correlates of protection. Determining the dose threshold required for re-infection of previously exposed volunteers will also be invaluable in modelling risk to inform public health strategies such as social distancing rules, especially as more people in the population are exposed or vaccinated.

3.10 Harmful Immune responses

There has been some concern around antibody dependent enhancement (ADE) of disease and other mechanisms by which re-infection with SARS-CoV-2, in the presence of suboptimal pre-existing immunity,

may lead to more severe disease, for example due to immunopathology[44]. Passive transfer of SARS-CoV-2 antibody to hamsters led to a small signal of increased weight loss compared with control animals [15]. Numbers were too small to conclude this was ADE and no mechanistic study was performed. Conversely, seropositive rhesus macaques were protected from disease on SARS-CoV-2 challenge [43]. While data on re-infection cases in humans is sparse, reassuringly, of the documented cases, the vast majority of these have been mild or asymptomatic[45]. In the recent SIREN cohort study of health care workers, only 15 (34%) of the 44 possible re-infected cases were symptomatic and of these only 2 (23%) reported typical COVID-19 symptoms [12]. There were no hospitalisations. Nevertheless, there have been reports of more severe disease on re-infection such as one recent case report describing a young healthy adult whose first episode of COVID-19 was mild but who was re-infected with a variant strain with more severe disease requiring hospitalisation [46]. There is therefore a theoretical risk that previous immune responses (by infection or vaccination) may contribute to more severe disease on re-infection. However, there is still no robust evidence of this for SARS-CoV-2.

In RSV disease (which shows many similarities to COVID-19 in terms of mechanisms of disease and immunity), T cells may contribute to enhanced disease in the absence of neutralising antibody.

A more common scenario in infectious disease is some pre-existing immunity leads to an abrogation of a second infection. Nevertheless, our careful dose escalation will ensure any risk of immune enhancement is minimised.

3.11 Risks and Clinical outcomes of SARS-CoV-2 infection

This protocol has been designed to minimise, as much as possible, risk to participants and their contacts, whilst providing a robust platform for vaccine efficacy evaluation. For the purpose of protocol design and informed consent, the degree of risk can be semi-quantified using data from observational studies of natural infection, which have also identified factors associated with risk of severe disease such as age, co-morbidities, gender and ethnicity. Animal models provide information correlating severity of pathology with route and dose of inoculation. In addition, there is emerging information concerning the efficacy of antiviral and immunomodulating therapy, which has the potential to attenuate risk to participants.

Much of the information that informs the general public of COVID-19, and indeed the scientific community, is derived from hospital-treated cases, that are, by definition, at the severe end of the spectrum. In conducting a dose ranging study commencing with an inoculum at the lowest measurable dose, investigators aim to achieve a model in which volunteers experience an infection that results in no symptoms, or symptoms no more severe than the common mild response of healthy people of the same age within the general population.

Young healthy adults in the 18-30 year old age are at low risk of severe outcomes following COVID-19. As of 6th November 2020 in England and Wales, 95 individuals in this age range have died due to COVID-19, but most had risk factors. Risk of hospitalisation in this age group (without accounting for co-morbidities) has recently been estimated at 0.08-0.39%. Protracted symptoms (“long COVID”) can occur but are less common in young people with mild disease, almost always resolving within 4 months.

3.10.1 Age as a risk factor for severe COVID-19

Age has been a major factor in severe outcome of COVID-19 in all series published so far [47]. In one large meta-analysis by the Imperial group of data from China, an estimated 20% of hospital-treated infections were severe with an overall infection-mortality rate of approximately 2% [48]. Using this huge dataset, it was estimated that the infection-mortality rate (95% confidence interval) in 20-29 year olds was 0·0309% (0·0138–0·0923), and in 30-39 year olds was 0·0844% (0·0408–0·185). In contrast, the estimated infection-mortality rate in the over-60s was 3·28% (1·82–6·18).

Modelling from France estimated that 0.5% of 20-29 year olds who were infected during the first pandemic wave (including those with co-morbidities) were admitted to hospital, using denominator data from passive surveillance and proportions of asymptomatically infected individuals from the *Diamond Princess* cruise ship to estimate the total number of infections over the same period [49]. These are likely to have been over-estimates, as more recent analysis of severe outcomes from several European countries using denominators estimated by seroprevalence data showed the following in young adults <30 years old [50].

- Risk of death following infection: 1.2-6.1 per 100,000 (0.0012-0.0061%)
- Risk of ICU following infection: 0.9-4.5 in 10,000 (0.009-0.045%)
- Risk of hospitalization following infection: 0.8-3.9 per 1,000 (0.08-0.39%)

Even these are likely to be over-estimates for a healthy young adult group, as the figures do not take risk factors or co-morbidities into account. No published data exist specifically describing the risk of hospitalization in the UK of individuals in the 18-30 year old age group once they are already infected. As of 6th November 2020, 95 adults aged 18-30 have died due to COVID-19, the majority of whom had co-morbidities or other risk factors (Ben Humberstone, Office of National Statistics [ONS], personal communication, [51].

Data from the ONS from the 16 weeks between 7th March and 26th June 2020 [52] covering the peak of the first pandemic wave show an estimated absolute risk of death in those aged 15-24 years of 0.5 in 100,000 (0.0005%) and those aged 25-34 of 1.6 in 100,000 (0.0016%). Additionally, the QCOVID living risk prediction algorithm [53, 54] provides an absolute risk of COVID-associated hospitalization in a White British 30 year old woman with no risk factors as 1 in 5076 (0.0197%).

Extrapolating study findings from young adults to high risk groups such as older people with co-morbidities is not always possible. However, young adults with mature intact immune systems are the benchmark for optimal immune responses. They are therefore the best group in which to identify correlates of protection against which vaccine-induced responses can be compared. So far, phase III studies have shown no major differences between young and older vaccinees, which also reassures about extrapolating in this context. Furthermore, a vaccine candidate tested by infection challenge of young adults that showed no effect on infection rate or viral shedding can reasonably be understood to have little protective efficacy in those with young healthy immune systems, and therefore would be even less likely to show efficacy in more high-risk populations with impaired immune responses. Host factors such

as age are also less likely to impact the efficacy of antiviral drugs and monoclonal antibodies, so extrapolation is more feasible for these types of studies. Finally, with widespread vaccination in the near future, the majority of high-risk individuals will have been vaccinated and almost all efficacy studies, including head-to-head comparisons of drugs and vaccines, will need to take place in younger volunteers. While young adults with no known underlying conditions may still develop unexpectedly severe outcomes following virus or drug administration, these are likely to be rare and can be further mitigated in an early phase clinical trial setting.

Detailed analyses of the existing phase III vaccine trials are not yet available, so although preliminary announcements have suggested no difference in efficacy in older adults and other high-risk groups this has yet to be fully evaluated. In the published Pfizer data, analysis was based on only 170 infections, with only 20 cases in those aged >65 and 5 cases in those aged >75, who are likely to respond least well to vaccination [55]. Similar issues with statistical power apply to subgroups with co-morbidities or immunosuppression, so we would argue that the efficacy of these first-generation vaccines remains uncertain and there remains a strong rationale to continue developing vaccines to improve protection in those who have difficulty mounting good immune responses.

3.10.2 Ethnicity as a risk factor for severe COVID-19

Initial analysis by the Office for National Statistics (ONS) showed that the rate of death involving the coronavirus (COVID-19) among almost all ethnic groups has been significantly higher than that of those of White British ethnicity[56]. Based on an initial statistical model adjusting for age, males of Black African ethnic background were 3.8 times more likely to die from a COVID-19-related death and females of Black African ethnic background were 2.9 times more likely than males and females who self-identified as White British. People of Bangladeshi, Pakistani, Indian and Mixed ethnicities also had statistically significantly raised risk of death involving COVID-19 compared with those of White ethnicity. However, much of this increased risk disappeared after taking into account geography, socio-economic characteristics and health measures such as pre-existing conditions, with Black African males having a 2.5 times higher corrected risk, Black African females having a 2.1 times greater corrected risk. For other ethnic minority groups the increased rate of COVID-19 mortality was also reduced following correction and the ONS concluded that the majority of the difference between ethnic groups in COVID-19 mortality was a result of socio-economic disadvantage and other circumstances such as high rates of employment in essential services including front line health care.

These data have been partially supported by findings from other studies. The OpenSAFELY study showed that after adjustment for other factors, Black and South Asian people were at higher risk with hazard ratios of 1.48 and 1.45 respectively [57]. However, the ISARIC-4C study showed an increased risk only in South Asian people after correction for other factors [58]. Some of this remaining increased risk may be due to unmeasured social and environment factors, although it is not possible to exclude biological risk factors at this stage. Thus, while there is an increased risk of poor outcomes following COVID-19 in people of some ethnic backgrounds, most is due to exposures, socioeconomic and health inequality factors, the data for which are incomplete. Furthermore, while the relative risk of death may be higher even when

certain factors have been taken into account, in young adults this still represents a very low absolute risk of severe outcomes. This is reflected in the QCOVID risk model, where risk of severe outcomes in young adults remains extremely low irrespective of ethnicity [53].

3.10.3 Personalised risk assessment using the QCOVID tool

As detailed above, analysis by the Office of National Statistics and the OpenSAFELY and ISARIC 4C studies of hospitalized patients have all concluded that the majority of the increased risk seen (particularly in Black and South Asian groups) was related to socioeconomic factors including greater exposure due to disproportionately being in front-line jobs. However, there remained some element of increased risk in BAME individuals that was still unexplained, with up to ~2.5 times increased risk of severe outcomes in Black African men, for example. Our public and participant inclusion and engagement as well as reviews by experts in BAME health (including Kevin Fenton, PHE, and Kamlesh Khunti, Centre for BME Health) highlighted two opposing views: (1) that inclusion and diversity should be maximized, and (2) that BAME people should not be subjected to any increased risk before those with no documented risk had been through the study procedures. To take these views into account and since this increase in risk was not identical across ethnicities, it was felt that a personalised risk assessment would be a better way to balance inclusivity with safety. Furthermore, a recruitment approach should be responsive to the most up-to-date data. The QCOVID risk scoring tool (qcovid.org) published by a consortium led by Julia Hippisley-Cox (University of Oxford) is an independent, validated risk assessment algorithm that integrates age, sex, ethnicity, geography, body mass index and co-morbidities to provide an individualised estimate of absolute mortality and hospitalisation risk. This provides an objective absolute risk of death and hospitalization based on the best available UK epidemiologic data. The tool is CE marked and will be recalibrated with up-to-date data every 3-6 months.

The potential risks associated with particular participant features (such as ethnicity, sex or BMI) may be balanced holistically and certain risk factors mitigated by other characteristics such as younger age (Table 1). Thus, a healthy White British 30 year old man or woman; a Black African 22 year old man; a 26 year old Indian man; a 24 year old Chinese man; and a 27 year old Chinese woman of BMI 23.5 would all have an absolute risk of death of 1 in 250,000 (0.0004%), thus establishing parity and eliminating any potentially increased risk associated with ethnicity that currently remains unexplained.

The QCOVID tool will be utilised in this setting to inform a global assessment of eligibility as well as providing further information as part of the participant's informed consent process. While we are not using absolute cut offs, scores will be considered as part of a holistic assessment of risk, including information on primary infection.

Table 1. Representative risk assessments using QCOVID

Absolute risk of death	Absolute risk of hospitalisation	Age	Sex	Ethnicity	BMI
1 in 250,000 (0.0004%)	1 in 4902 (0.020%)	30	F	White British	23.5
1 in 250,000 (0.0004%)	1 in 7143 (0.014%)	30	M	White British	23.5
1 in 250,000 (0.0004%)	1 in 8475 (0.012%)	22	M	Black African	23.5
1 in 250,000 (0.0004%)	1 in 5319 (0.019%)	26	M	Indian	23.5
1 in 250,000 (0.0004%)	1 in 9901 (0.010%)	24	M	Chinese	23.5
1 in 250,000 (0.0004%)	1 in 4587 (0.022%)	27	F	Chinese	23.5
1 in 250,000 (0.0004%)	1 in 4329 (0.023%)	30	F	White British	27.7
1 in 250,000 (0.0004%)	1 in 6211 (0.016%)	30	M	White British	27.7
1 in 250,000 (0.0004%)	1 in 7143 (0.014%)	22	M	Black African	27.7
1 in 250,000 (0.0004%)	1 in 3984 (0.025%)	27	M	Indian	27.7
1 in 250,000 (0.0004%)	1 in 8621 (0.012%)	24	M	Chinese	27.7
1 in 250,000 (0.0004%)	1 in 4000 (0.025%)	27	F	Chinese	27.7
1 in 200,000 (0.0003%)	1 in 2475 (0.04%)	22	F	Black African	23.5
1 in 200,000 (0.0003%)	1 in 2959 (0.034%)	23	F	Indian	23.5
1 in 200,000 (0.0003%)	1 in 4329 (0.023%)	30	F	Chinese	23.5
1 in 166,667 (0.0006%)	1 in 3333 (0.03%)	30	M	Indian	23.5
1 in 125,000 (0.0008%)	1 in 2632 (0.038%)	30	F	Indian	23.5
1 in 111,111 (0.0009%)	1 in 2169 (0.046%)	30	F	Black African	23.5
1 in 100,000 (0.001%)	1 in 4739 (0.021%)	30	M	Chinese	23.5
1 in 83,333 (0.0012%)	1 in 2755 (0.036%)	30	M	Black African	23.5

3.10.4 Consequences of natural infection

Natural infection by SARS-CoV-2 mostly results in no or minor symptoms. In the careful observation of passengers and crew in quarantine on board the cruise ship *Diamond Princess*, 712 of a total of 3711 persons were found to be infected with SARS-CoV-2, and 410 (58%) of those infected were asymptomatic at the time of testing. Amongst the latter asymptomatic cases, a small fraction - 12% - subsequently developed symptoms [59]. Where infection results in symptoms, international published epidemiological surveys indicate a median incubation period of 5 days. People infected with SARS-CoV-2 are most infectious (i.e. exhibit significant viral shedding) from 2 days before, until 7 days after symptom onset [60].

Evidence from observational studies of infected individuals and non-human primates have shown that changes occur in the lungs even sometimes during asymptomatic [61]. These are generally seen as ground-glass shadows on imaging that may be temporary or persistent. They can be early (around 5 days post-infection) or late (around 10 days) and can progress to more severe lung inflammation characterised by widespread changes in both lungs.

A wide range of symptoms can occur from disease onset. In a detailed, multi-site CDC study of 274 people in the United States with symptoms, who were tested as outpatients and found to be positive by RT-PCR and then interviewed in the period 14-21 days after the initial swab, the complaints experienced in descending order of frequency were fatigue, cough, headache, body ache, fever, chills, loss of taste, loss of smell, diarrhoea, congestion, dyspnoea, nausea, sore throat, chest pain, abdominal pain, confusion and vomiting. Among respondents aged 18–34 years with no chronic medical condition, 19% (9 of 48) reported not having returned to their usual state of health. Age ≥50 versus 18–34 years (adjusted odds ratio [aOR] = 2.29) and reporting 3 or more versus no chronic medical conditions (aOR = 2.29) were associated with failure to regain usual health. Obesity (body mass index ≥30 kg per m^2) (aOR 2.31) and a psychiatric condition (aOR 2.32) also were associated with failure to recover completely after adjusting for age, sex, and race/ethnicity.

3.10.5 Consequences of development of disease

In a minority of higher-risk individuals, initial symptoms of natural infection are followed by a dramatic decline in clinical state, characterized by worsening dry cough, severe dyspnoea and profound malaise. This is caused primarily by uncontrolled viral replication and dissemination to the lower respiratory tract followed by onset of a diffuse maladaptive lung inflammation causing failure of gas exchange resulting in low arterial oxygen saturation. This, together with generalised sepsis (including coagulation disorders and cardiac complications) can lead to multi-organ failure. Severe COVID-19 may also lead to acute cardiac, kidney, and liver injury, in addition to cardiac arrhythmias, rhabdomyolysis, coagulopathy, and septic shock. In a small number of children and teenagers, a condition (Paediatric Inflammatory Multisystem Syndrome temporally associated with COVID-19 or PIMS-TS) has also been reported that leads to more severe disease [62, 63]. It is unclear whether this syndrome occurs in adults.

The UK ISARIC study investigated 20,133 people with COVID-19 admitted to hospitals whose median age was 73 years (interquartile range 58-82), the median duration of symptoms before admission was 4 days and the median duration of hospital stay was 7 days [60]. Amongst this cohort, 23% had no documented reported comorbidity. The commonest comorbidities of the remainder were chronic cardiac disease

(31%), diabetes (21%), chronic pulmonary disease (18%), asthma (14%) and Chronic Kidney disease (16%). Increased age and comorbidities including obesity were associated with a higher probability of mortality. The investigators reported 3 major and distinct clusters of symptoms: (i) respiratory (cough, sputum, sore throat, runny nose, ear pain, wheeze, and chest pain) – the commonest cluster; (ii) systemic (myalgia, joint pain and fatigue); and (iii) enteric (abdominal pain, vomiting and diarrhoea). Across the entire cohort, 41% of patients were discharged alive, 32% died and 41% continued to receive care at date of reporting. 17% required admission to High Dependency or Intensive Care Units; of these, 17% were discharged alive, 37% died and 46% continued to receive care at the reporting date. Of those receiving mechanical ventilation, 20% were discharged alive, 53% died and 27% remained in hospital. Overall, younger age, female sex, and lack of co-morbidities or obesity were associated with lower mortality in hospital. However, individuals aged 18-30 made up only a small proportion of cases (with no male preponderance) [60].

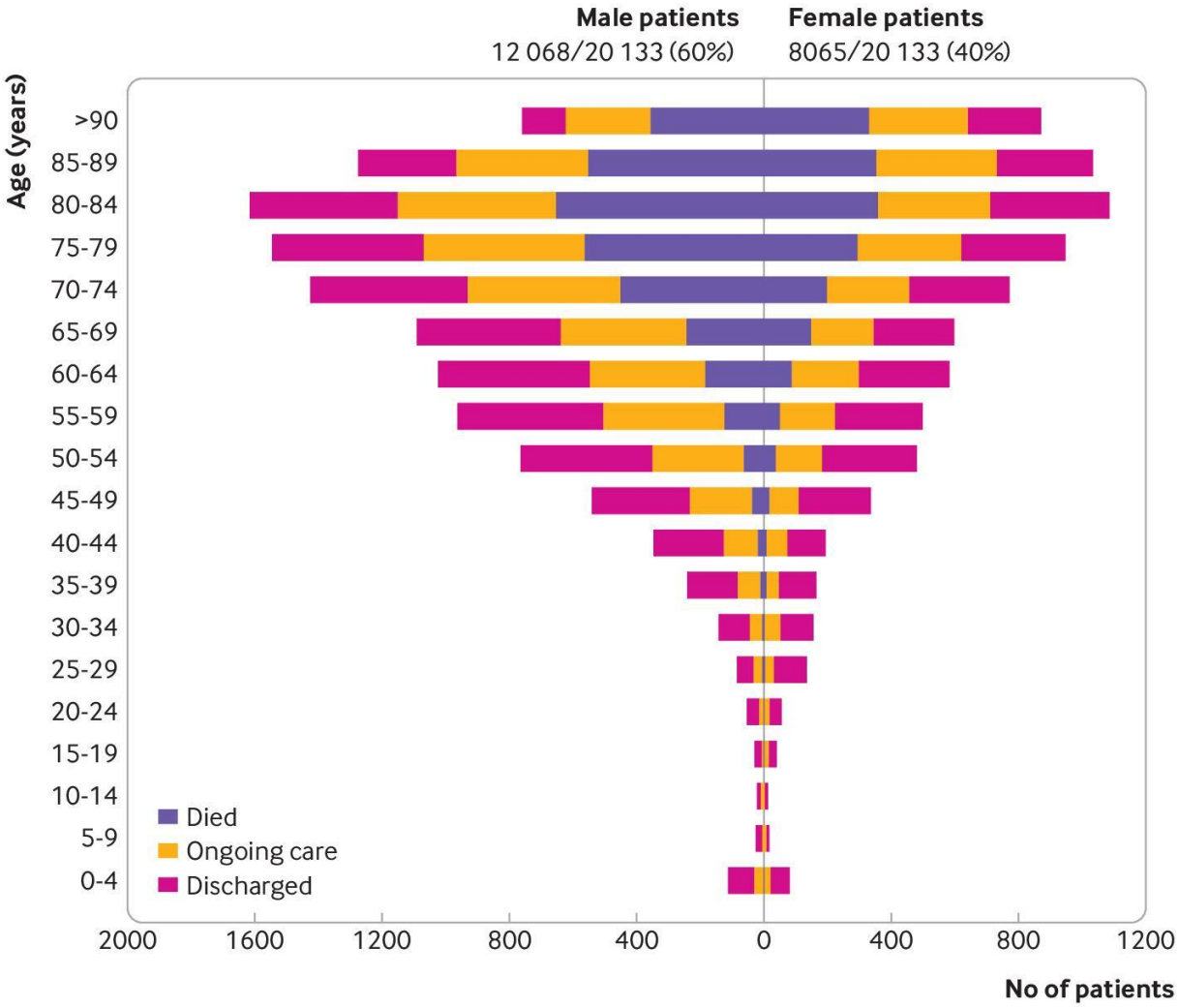

Figure 3. Outcomes of 20,133 hospitalised COVID-19 patients by age

Together, these data suggest that SARS-CoV-2 infection in healthy young adults with no co-morbidities rarely causes more than mild self-limiting symptoms.

3.12 Treatment of Severe COVID-19

Patients with severe COVID-19 often require oxygen and ventilatory support, together with correction of physiological dysfunction usually in intensive care units. The most severely ill usually require mechanical ventilation, plus one or more of haemodynamic support, correction of coagulopathy, treatment of heart failure and correction of acute renal failure. Standard of care of severely ill people in the UK now includes dexamethasone and Remdesivir. In patients hospitalized with COVID-19, the use of dexamethasone resulted in lower 28-day mortality among those who were receiving either invasive mechanical ventilation or oxygen alone at randomization but not among those receiving no respiratory support [64]. While the efficacy differs between studies, treatment with the anti-viral drug Remdesivir 200mg IV daily has been shown to be superior to placebo in shortening the time to recovery in adults hospitalized with COVID-19 and evidence of lower respiratory tract infection [65] In contrast, the recent SOLIDARITY trial [66] suggested no overall improvement in mortality or clinical outcome in hospitalized patients, but this non-placebo controlled international multi-centre study enrolled a wide range of patients at various stages including late disease, where it is expected anti-viral treatment could be less efficacious.

A recent interim clinical commissioning policy by the MHRA has also recommended use of interleukin-6 inhibitors (Toclizumab or Sarilumab) for patients admitted to ICU with COVID-19 pneumonia. This was based on emergent data from the REMAP-CAP trial of an overall mortality reduction of 24% and reduced time on ICU (approximately 1 week less) [67].

Clinical trials looking at efficacy of other treatments are ongoing, including several synthetic monoclonal antibody therapies, convalescent plasma therapy, anti-inflammatory treatments such as colchicine and anti-platelets e.g. aspirin.

3.13 Pre-clinical and clinical experience with REGN-COV2 (Regeneron Monoclonal Antibody cocktail Or Ronapreve) and Paxlovid for COVID-19:

Regeneron™ has developed 2 non-competing, high-affinity human IgG1 anti-SARS-CoV-2 monoclonal antibodies (mAbs), REGN10933 (casirivimab) and REGN10987 (imdevimab)[68, 69]. These mAbs bind specifically to the RBD of the SARS-CoV-2 spike glycoprotein and act as potent neutralising antibodies by blocking interaction with the host ACE2 receptor, which is responsible for viral uptake into host cells. REGN10933 and REGN10987 are intended to be used as a combination therapy (REGN-COV2) as they bind to distinct and non-overlapping regions of the RBD with the view to broaden cover against circulating viral variants(40). Additionally, in-vitro studies have demonstrated that using this non-competing combination minimises the risk of escape mutation development due to selection pressure; with no escape mutants developing with the dual non-competing REGN-COV2 mAB regime whilst, development of escape mutations occurred when single mAB or dual competing mAB therapies were tested in vitro [69].

In vivo efficacy has been assessed in 2 non-human primate (NHP) models and 1 syrian golden hamster model [8]. In NHP studies, REGN-COV2 reduced viral load in upper and lower airways and reduced lung pathology, both when given as prophylaxis and treatment for SARS-CoV-2 infection. In the Syrian golden

hamster study, REGN-COV2 limited weight loss (indicator of morbidity in this model) when used prophylactically and as treatment. No evidence of ADE was observed in any of the studies, as assessed by increased viral load, more severe lung pathology, or enhanced weight loss. No evidence of escape mutant selection was observed in the NHP study where it was assessed.

Recent results from phase 3 studies has led to the licensure of REGN-COV-2 on 20th August 2021 (also known as 'Ronapreve') in the UK for both the prophylaxis and treatment of COVID-19 disease[7]. It has been administered to a total of 7116 subjects (approximately 4666 via IV administration and 2450 via subcutaneous administration.

In COV-2067 a randomised, double blinded, placebo-controlled phase 1-3 study in non-hospitalised participants, individuals with at least one risk factor for severe COVID-19 received a single intravenous infusion of REGN-COV2 within 3 days of having a positive SARS-CoV-2 PCR test. 4567 adult participants were randomized to receive one of three doses of REGN COV2 combination, either the 1,200mg dose (n=838), 2,400 mg dose (n = 1529) or the 8,000mg dose (n =700), or placebo (n = 1500). The primary end point of this study was the proportion of subjects with COVID related hospitalisation or all cause death through to Day 29. In the 1,200mg dose this end point was met in 7 individuals (1.0%) treated versus 24 in placebo (3.0%), demonstrating a 70% relative risk reduction (p=0.0024). Interim safety results released from this study demonstrated a tolerable safety profile, with serious adverse events being numerically more frequent with placebo than REGN-COV2 treatment (0.8% high dose REGN-COV2, 1.6% low dose REGN-COV2; 2.3% placebo). None of the SAEs were considered to be related to the study drug. Numerically more infusion reactions occurred with the REGN-COV2 high dose compared to placebo but not with the low dose REGN COV2 regime (1.5% high dose; 0% low dose; 0.4% placebo). This study demonstrated that overall, treatment resulted in a reduction in the average daily change in viral load through day 7 (mean time-weighted average change from baseline) with -0.71 log10 copies/mL for 1,200 mg dose ($p < 0.0001$) and -0.86 log10 copies/mL for 2,400 mg dose ($p < 0.0001$) compared to placebo. The greatest benefit in viral load reduction was seen in individuals who had higher starting viral loads and those who were seronegative at baseline (I.e. slow immune responders)[7-9]. Self-reported symptom duration was also reduced in treated participants versus placebo, with a median duration of 10 days in 1200mg dose cohort versus 14 days for placebo treated subjects (p=0.0001). Results have demonstrated no significant difference in virologic, clinical efficacy, hypersensitivity reactions or SAEs across dose groups.

Best results in viral load reduction are seen in those with highest starting viral loads, and given our direct inoculation to the respiratory mucosa, our volunteers may well have high viral loads. Post hoc analyses performed by Regeneron have demonstrated a similar trend in individuals seropositive at baseline, however, were not powered for statistical significance in this group.

The applicability of this data to our cohort is limited. We are excluding any individuals with risk factors for severe COVID-19. The seropositive group in this study (38% of total) were individuals who had antibodies at enrolment (within 72 hours of positive microbiological testing for SARS-CoV-2 infection) with the

assumption that this represented a rapid immune response to SARS-CoV-2 after infection. However, data on prior SARS-CoV-2 exposure/ infection and the existence of antibody results prior to symptoms/ positive test result is unavailable so it is unclear whether this group, in part, could represent individuals with pre-existing antibodies due to prior exposure or infection. It can be hypothesised that our cohort may behave like the seropositive group in this study meaning the benefit of REGN-COV2 is not as pronounced based on the current data. Additionally, this study was not powered to look at efficacy in seropositive individuals alone. However, it is clear that this is a safe and well-tolerated treatment with evidence of a significant reduction in hospitalisation or death with COVID-19 and a reduction in median duration of symptoms. Furthermore, giving the treatment to seropositive patients did not increase viral load or demonstrate evidence of ADE, justifying further investigation into its use in seropositive individuals from known historic infection despite a lack of current efficacy data. It would also be expected that a targeted treatment against SARS-CoV-2 infection would have greatest impact when given early in the course of infection. Given that we are deliberately inoculating individuals with SARS-CoV-2, using a targeted rescue therapy is an important ethical consideration recognised by the World Health Organisation in their regulatory framework for challenge models [70]. .

Substantial Amendment 7 – Introduction of Paxlovid

REGN-COV2 (Ronapreve) was included as rescue therapy for part of the dose escalation phase, Groups 1 and 3, and only required for 2 volunteers.Once the preferred rescue therapy, paxlovid, is fully approved as part of SA007, REGN—COV2 will no longer be used. Paxlovid was first authorised for use 31 December 2021; until May 2022 supplies were not available for use in the study, thus REGN-COV2 remained the best option until this time.

Paxlovid is the brand name for Ritonavir boosted Nirmatrelvir (PF 072321332). Of the two active parts of the medication Nirmatrelvir is a protease inhibitor active against all coronavirus which are known to infect humans, and originally developed following the SARS CoV-1 outbreak.[71] Ritonavir is a strong cytochrome P450 3A4 inhibitor which has been used extensively for boosting the activity of HIV protease inhibitors,[72] it has no direct activity against coronaviruses, and its role is in increasing the circulating concentrations of Nirmatrelvir. Phase one clinical trials showed that oral activity in a mouse-adapted SARS-CoV-2 model achieved plasma concentrations exceeding invitro antiviral cell potency at levels. Paxlovid has been approved for use across multiple countries including the UK where it has a current licence for use to reduce the risk of the progression to severe disease from SARS-Cov2 for high risk individuals when given within 5 days of symptom onset.

The EPIC-HR trial[73] was a placebo controlled RCT which randomised 2246 patients deemed to be of high risk of severe COVID-19 to either Paxlovid or placebo within 3 days of symptom onset. The primary endpoint was hospitalisation or death within 28 days of infection, this was met in 5 of 697 patients in the Paxlovid group, and 44 of 682 of the placebo group a relative risk reduction of 88.9% (Absolute risk

reduction 5.84% [95% confidence interval, −7.78 to −3.84; p<0.001]). When mortality was assessed 13 patients died in the placebo group, compared to zero in the Paxlovid group. By day five, viral load was also reduced by an adjusted 0.868±0.105 log_{10} copies per millilitre (95% CI, –1.074 to –0.6615; P<0.001). Adverse event rates were not significantly different between the two arms (22.6% in Paxlovid group v 23.9% in Placebo group). Medication related AE in the Paxlovid group were modest, and were largely dysgeusia or diarrhoea and of which the majority were grade 1 or 2, cessation of the drug occurred in <0.8% of cases. Importantly this study excluded patients who had either been previously infected or vaccinated for SARS-CoV2, which is not the case in the COV-CHIM study. Notably all participants in this study were all deemed to have a risk factor for progression to severe disease due to pre-existing co-morbidities, which would have been exclusion criteria for COV-CHIM. This may mean the beneficial effects of Paxlovid in the volunteers of the COV-CHIM study are more modest.

Comparison between Paxlovid and REGN-COV2 for Rescue Therapy in COV-CHIM

There have be no direct head to head studies comparing the use of REGN-COV with Paxlovid either for hospitalised patients or in the prevention of severe disease. Suitability and recommendation of which should be considered as first choice rescue therapy for COV-CHIM therefore is reliant upon comparing the above efficacy data.

In the COV-CHIM01 study the majority of patients are expected to have baseline sero-positivity to nucleocapsid or spike protein or both. While baseline serum antibody appears to alter the efficacy of these medications, the reduction in efficacy for seropositive patients is clearer for REGN-COV2. The RECOVERY trial of patients hospitalised by COVID-19 did not find a significant effect of REGN-COV2 in patients with pre-existing spike antibodies (RR 1.09 (95% CI (0.94-1.25).[74] While an RCT of REGN-COV in an outpatient setting did find significantly reduced rate of hospitalisation or death in seropositive patients compared to control (p=0.04), it was less efficacious compared to seronegative individuals and the study did not differentiate patients positive for anti-nucleocapsid from anti-spike protein.[75] This pooled group may have shown a difference not present in anti-spike positive patients alone. Although the EPIC-HR study excluded previously vaccinated individuals, subgroup analysis showed that patients with positive SARS-CoV-2 serology at baseline continued to have significant difference in primary outcome, albeit more modest, than that seen in serology negative patients (Absolute risk reduction in primary end point in Paxlovid arm -1.34% [-2.45, -0.23] in serology positive patients -10.25 [-13.28, -7.21] in serology negative patients).[73] Interim analysis has recently been made available for the EPIC-SR study.[76] This study

compared PAXLOVID v Placebo in patients with a standard risk of progression but no previous vaccination or vaccinated individuals with a risk factor for developing severe disease. The primary endpoint was the same as for the EPIC -HR study; hospitalisation or death within 28 days of infection. The interim analysis at 80% of enrolment reported for Paxlovid group of 3/428 events [0.7%] versus 10/426 events [2.4%] in the control group (p=0.051) with no deaths in either arm. The final results are awaiting release.

The oral preparation of Paxlovid also presents further logistical benefits over REGN-CoV2 in terms of administration, with its oral route of administration avoiding the placement of cannula which have associated inconvenience to the volunteer as well as risk of complication from infection, thrombosis and bleeding. Its availability as a standard preparation will also facilitate speed of administration in comparison to REGN-COV2, which currently requires support from the on-call pharmacy team to facilitate preparation and administration, with associated lead times.

The inclusion of Ritonavir and its effects on cytochrome P450 3A4 inhibition, mean that there are potential implications with regards to use of medications, supplements and contraceptive therapies. For the purposes of the COV-CHIM01 study, the majority of participants taking regular medications that have a significant interaction with Paxlovid would be excluded by virtue of the underlying condition necessitating that medication. The main exception being participants taking hormonal contraceptives containing ethinyl estradiol which may have reduced efficacy, due to reduced drug concentration while taking Paxlovid. Women who use the combined oral contraceptive pill as their method of contraception will be required to use barrier contraception with spermicide while taking Paxlovid and for 30 days after stopping Paxlovid. Participants will be assessed for potential interactions between Paxlovid and any regular medications or supplements at screening and in the event that acute medications are prescribed concurrently.

3.14 Potential long-term complications of COVID-19

Currently, it is still too early to assess the long-term complications of COVID-19. However, it is possible that severe ARDS may be associated with long-term pulmonary pathology including fibrosis. In the case of the SARS outbreak of 2003, a 2-year study of a selected population of SARS survivors, showed significant impairment of DLCO, exercise capacity and health status had persisted [77]. In the wake of the SARS epidemic, some survivors experienced chronic fatigue syndrome for several years after illness [78]. Currently, large cohort studies (such as the UK sponsored PHOSP-Covid) have been set up to collect this information. However, to date, there has been no evidence that long-term sequelae are significantly associated with asymptomatic or mild SARS-CoV-2 infection. “Long COVID” is currently being widely

discussed [79] and refers to a post-infective state that has been experienced by COVID-19 patients including health care workers, characterised by fatigue and lassitude, extending for weeks and even months. The incidence and nature of this illness is currently being investigated by several groups internationally [80].

Data from the COVID Symptom Study (led by Tim Spector and Claire Steves at King's College London) is using self-reported symptom data from a mobile phone app to analyse the frequency and duration of symptoms related to COVID-19. In that dataset, preliminary data were obtained of 629 individuals in the 18-30 year old age group with PCR-confirmed SARS-CoV-2 infection and who were non-smokers, had a BMI<25, had no co-morbidities and consistently logged into the app (data extracted September 2020, personal communication, Claire Steves, King's College London). In this cohort, 78% were women but the frequencies and duration of symptoms were similar between men and women (Table 2 and Table 3). The most frequent symptoms were fatigue (78%), headache (74%), loss of smell (61%), sore throat (59%) and cough (48%) as shown in Table 2. On average, these symptoms lasted no more than 5 days although some rare individuals experienced loss of smell and fatigue for up to 4 months before resolution (Table 3, figure 4). Nevertheless, fatigue had resolved in 75% of individuals after 11 days or fewer and 90% of individuals after 19 days or fewer, and loss of smell in 75% after 9 days or fewer and 90% after 14 days or fewer (Figure 4). Further analysis of the overall dataset has allowed the development of a risk prediction system for "long COVID", which has shown relatively lower risk in younger individuals with <5 symptoms [81]. Due to the scarcity of data on re-infection, little is known about the consequences of re-infection and risk of developing long COVID symptoms. However, importantly, of the approximately 300 cases of re-infection, to date there have been *no* reported cases of long COVID following re-infection in this study (data reviewed February 2021, personal correspondence, Claire Steves, King's College London). Further, data on the SIREN study suggests re-infection leads to development of COVID-19 disease with fewer symptoms compared to the primary episode, which, based on our limited knowledge of risks for long-COVID would imply a reduced risk for protracted symptoms following a secondary infection.

Recent data published from the COVID symptom study also suggests that immune sensitisation conferred by vaccination is protective against long COVID. Once adjusted for age, BMI and gender, a full course of vaccination halved the risk of symptoms persisting beyond 28 days (OR 0·51, 95% CI 0·32–0·82; p=0·0060) as well as reducing the total number of symptoms reported when compared to unvaccinated individuals[82].

Table 2. Frequency of COVID-19 related symptoms in healthy 18-30 year olds with PCR-confirmed infection

Frequency	Overall	Male	Female
Fatigue	0.783784	0.791045	0.780933
Headache	0.739269	0.671642	0.756592
Loss of smell	0.605723	0.544776	0.62069
Sore Throat	0.586645	0.514925	0.604462
Persistent cough	0.481717	0.492537	0.476673
Shortness of breath	0.36725	0.343284	0.371197
Fever	0.36407	0.425373	0.344828
Chest pain	0.357711	0.291045	0.375254
Skipping meals	0.349762	0.298507	0.363083
Unusual muscle pains	0.343402	0.358209	0.338742
Hoarse voice	0.298887	0.246269	0.312373
Diarrhoea	0.233704	0.186567	0.245436
Dizziness	0.197138	0.156716	0.208925
Abdominal pain	0.18124	0.156716	0.184584
Eye soreness	0.151033	0.141791	0.15213
Confusion, disorientation or drowsiness	0.138315	0.164179	0.127789
Rashes	0.028617	0.014925	0.032454
Blisters	0.009539	0.007463	0.010142

Table 3. Duration of COVID-19 related symptoms in healthy 18-30 year olds with PCR-confirmed infection

Duration (days)					
Symptom	Median	Min	Max	75th centile	90th centile
Loss of smell	5	1	119	9	14
Fatigue	5	1	102.5	11	19
Persistent cough	4	1	46	9	14.8
Headache	3.5	1	72	8	15
Shortness of breath	3	1	92	9	17
Chest pain	3	1	92	7	17
Skipping meals	3	1	66	6	10
Sore Throat	3	1	52.5	7	12.2
Hoarse voice	2.5	1	46	6	11
Confusion, disorientation or drowsiness	2.5	1	34	6	11.4
Dizziness	2.5	1	32.5	7	18
Rashes	2.25	1	9.5	4.75	6.9
Unusual muscle pains	2	1	92	5	9.5
Abdominal pain	2	1	66	5.75	12
Fever	2	1	46	4	8
Diarrhoea	2	1	32.5	4	9
Eye soreness	2	1	29	4	9.2
Blisters	1.25	1	9	1.75	5.5

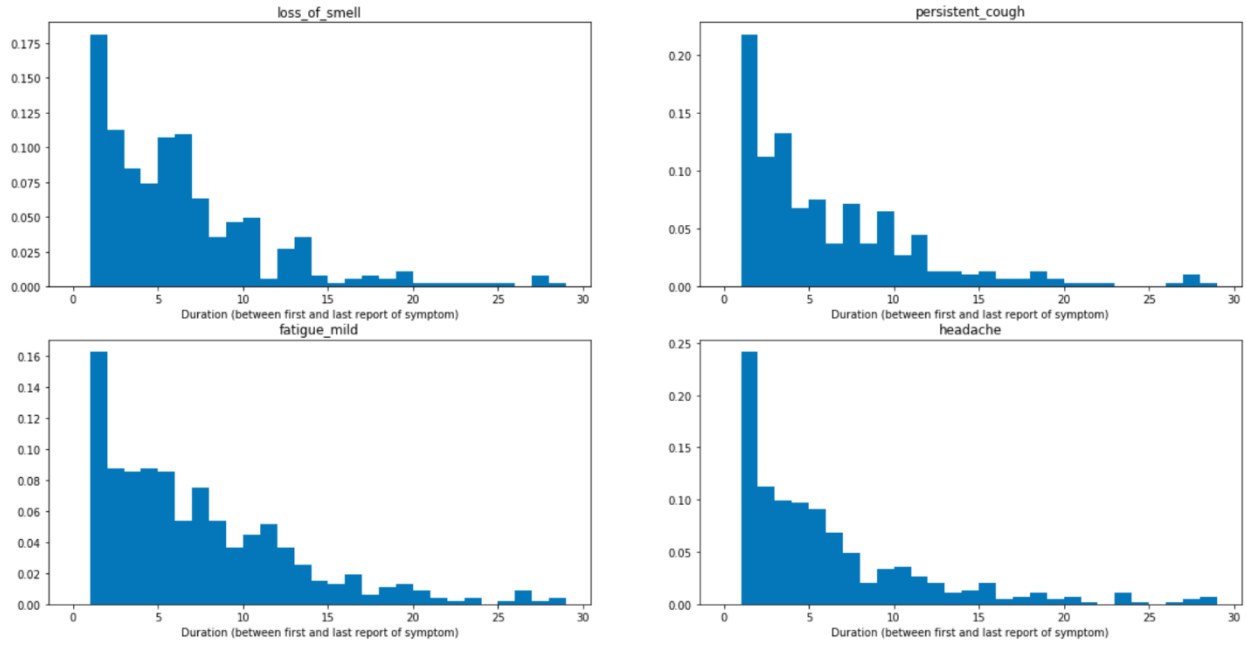

Figure 4 Frequency of individuals with the most common COVID-19 associated symptoms by duration.

From these data and considering the intranasal inoculation of SARS-CoV-2 in this study, loss and alteration of smell is a potential specific risk, even in healthy young adults and may persist in rare cases for weeks to months. Objective testing has shown that anosmia may be even more common than when assessed by self-report and may be the only symptom, particularly in young adults. In addition, parosmia (alteration in the sense of smell that can severely impair appetite due to familiar foods triggering a foul smell) has previously been reported in a large proportion of those with post-viral loss of smell. It is now also being reported during the COVID-19 pandemic and may persist for longer than anosmia. While anosmia is most common in those aged 40-45, parosmia may occur with similar frequency across the age ranges. Some data exist that suggests the usefulness of treatments including steroid rinses, topical vitamin A and omega-3 supplements that may reduce these symptoms or speed recovery. A placebo-controlled randomized controlled trial accepted for publication has shown that oral and intranasal steroids given at 4 weeks in those with persistent anosmia achieves better recovery rates (Claire Hopkins, ENT consultant, Guy's and St Thomas' Hospital, personal communication).

3.15 Mitigating Risk in Experimental Human SARS-CoV-2 Infection

The data outlined above has been used to inform the study design to minimise risk to participants. We will include individuals aged 18-30 years only. Exclusion criteria will include any risk factors for severe COVID-19 including previous hospitalisation with COVID-19, obesity and *any* comorbidity detected on screening. Prior to enrolment, each potential participant deemed to be suitable for entry into the study (based on inclusion/exclusion criteria and their screening assessments) will undergo a personalised risk assessment using the QCOVID tool in combination with emergent results from individuals experimentally

infected with SARS-CoV-2 in this study. The risk assessment will be reviewed by a member of the study medical team, documented and discussed with the participants before enrolment.

Other ways to reduce risk as much as possible include (i) use of a low infecting dose (ii) avoiding direct instillation to the lung, (iii) early recognition of features associated with progression to severe disease and (iv) rescue therapy for infected participants (see section 3.12).

i) Dose finding will occur in a cautious and staged manner, starting with the lowest infecting dose (10 $TCID_{50}$) of a Good Manufacturing Practice (GMP) manufactured virus. Administration will only occur 2 weeks after the same dose has been safely given to three seronegative individuals in the parallel study by hVIVO. If, as expected, the tolerability of each dose is comparable in seropositive and seronegative individuals, we will commence subsequent doses at the same time as hVIVO.
ii) Inoculation will occur via intranasal deposition (avoiding direct instillation to the lung).
iii) a. The challenge study will be conducted in a fully equipped clinical study unit with appropriate containment conditions. Participants will be quarantined in rooms with appropriate ventilation to prevent the spread of infection (either negative pressure rooms or positive pressure ventilation lobbies) for a minimum of 14 days post challenge, under the care of experienced medical and nursing staff. Additionally, research volunteers will be continuously monitored using state of the art medical monitoring, including CT imaging, to sensitively identify changes in the lung. This will allow early recognition of features associated with progression to severe disease, including viral hyper-replication, and lower airway inflammation.

    b. If any participant develops symptoms beyond mild disease (i.e. that would be comfortably managed conservatively at home in the general population) they will be moved to an NHS inpatient bed and receive full access to best standard care by an experienced clinical team. Our trial team is made up of senior ITU, respiratory and infectious diseases consultants who will be monitoring volunteers in real time to ensure volunteers receive timely treatment and transfer as appropriate.
    c. Participants will be followed up for 12 months to monitor for prolonged symptoms and will be referred to appropriate specialist services if necessary. This study is fully indemnified if a participant becomes unexpectedly unwell.

iv. Participants in the dose escalation phase will be treated with either a targeted combined monoclonal therapy (REGN-COV2) or oral antiviral (paxlovid), depending on timing of enrolment in the study, if they develop any warning symptoms or signs of COVID-19 disease (see section 8.9). The need to continue this for our dose confirmation phase (Group 2 and Group 4) will be assessed after review by the DSMB. Full details regarding the two phases of the study can be found in section 6.1.

3.16 Our group is well placed to perform human respiratory pathogen challenge studies

The UK is world-class in having extensive recent experience using models of respiratory pathogen challenge. In 2016 our group in Oxford successfully developed the first aerosolised Bacille Calmette-Guérin (BCG) human challenge in healthy UK adults, to be used as a model of *Mycobacteria tuberculosis* infection (NCT02709278). To date we have safely challenged over 50 volunteers with this respiratory BCG infection This model is being used as a unique platform to investigate mucosal immune responses from point of infection (NCT03912207). Our co-investigator Professor Andrew Pollard has successfully developed a human Salmonella Typhi challenge which was instrumental in the development of the Typhoid conjugate vaccine [83, 84]. Our partners at Imperial College London, Chris Chiu and Peter Openshaw, who will be running the SARs-COV-2 challenge in naïve volunteers and with whom we are working closely, began conducting experimental human infections with RSV in 2010. In 2015, the influenza challenge model was established at Imperial and together these models have been shown to be safe and well-tolerated as well as providing unique platforms for the study of immunity and immunopathogenesis during acute infection [85-87]. Additionally, in developing this protocol, we have drawn on the expertise of a large group of UK experts with expertise in the ethics, regulation, virology, clinical implementation and interpretation of human infection challenges studies. These include Robert Read (Southampton), whose extensive experience of initiating and developing new respiratory tract human infection challenge models, including genetically modified *Neisseria lactamica* and wild type *Bordetella pertussis* [88], and mitigation of risks against potentially hazardous challenge pathogens has been invaluable in study development.

3.17 Hypothesis

1. SARS-CoV-2 infection in experimentally infected healthy adult volunteers with a documented history of previous SARS-CoV-2 infection and/or vaccination is safe and well tolerated.
2. SARS-CoV-2 infection allows early viral replication to be measured, enables monitoring of innate and adaptive immune responses and radiological changes which act as surrogates of protection and disease.
3. Markers of adaptive immunity will correlate with detectable viral load.

4 OBJECTIVES AND ENDPOINTS

This is a dose escalation study in which healthy adults (18-30 years) will be experimentally infected with SARS-CoV-2, with the aim of causing PCR-confirmed upper respiratory infection with minimal or no illness. The number of volunteers in this study has been chosen to generate sufficient data to meet the objectives described below, whilst minimising the number of volunteers exposed to wild type SARS-CoV-2 intranasal challenge.

Sample analysis for the completion of exploratory endpoints may be performed under the ethically approved OVC Biobank protocol.

	Outcome Measure	Time point
**Co-Primary**<br>To assess safety and human clinical response to wild type SARS-CoV-2 intranasal challenge in both previously infected (vaccinated or unvaccinated) and uninfected vaccinated volunteers.	Presence of solicited and unsolicited adverse events and other objective parameters including physical examinations, smell test, cognitive tests, vital signs, pulmonary CT, pulmonary function tests, ECG, cardiovascular imaging and clinical laboratory results.	AEs collected on electronic diary until D84 and additionally at each scheduled follow up visit.<br>Objective parameters will be undertaken as per SoA.
**Co-Primary**<br>Selection of the SARS-CoV-2 dose(s) required to induce upper respiratory tract infection in 50% (+/-10%) of previously SARS-CoV-2 infected (vaccinated or unvaccinated) and uninfected vaccinated healthy volunteers following intranasal challenge.	Defined by laboratory identification of SARS-CoV-2, from nasal-oropharyngeal swab, using qPCR and/or quantitative live viral detection at two consecutive 12-hourly time points starting 24 hours post-inoculation and up to discharge from quarantine.	Twice daily, during quarantine period (D0 – D14/ until discharge criteria met).
**Secondary**<br>To assess the SARS-CoV-2 viral dynamics in upper respiratory samples from previously infected and uninfected vaccinated individuals including; determination of the incubation period, peak viral load and the mean duration of infectious viral shedding.	From quantitative virology measured using qPCR and/or live viral detection on nasal-oropharyngeal samples.	Twice daily, during quarantine period (D0-D14/ until discharge criteria met) and additionally at each follow up visit.
To identify laboratory markers of the immune response that	Laboratory markers of innate and adaptive immunity, including but not limited to ex-vivo ELISpot, flow cytometry, ELISAs, transcriptomic analysis, neutralising antibody	D-2, D2, D5, D7, D11, D14 during quarantine stay and at each follow up visit.

correlate with the levels of viral load.	analysis and cytokine analysis performed on peripheral blood and nasal and pharyngeal samples.	
**Exploratory**		
To explore alternate measures of viral shedding.	e.g. Quantitation and detection of virus in exhaled breath using facemask insert technology; other methods of live virus detection; viral sequencing; antigen detection via CE marked lateral flow tests	Face masks: Once daily during quarantine period with a pre-challenge baseline measurement (D-1 to D14+/ until discharge criteria met). Other methods of virus detection will occur on selected samples.
Other exploratory immune endpoints.	e.g. HLA and whole genome sequencing on peripheral blood	
Sample analysis for the completion of exploratory endpoints may be performed under the OVC Biobank research tissue bank protocol (REC: 16/SC/0141)		

5 COMMITTEES

5.1 Trial Steering Committee (also known as Medical Oversight Committee)

A Trial Steering Committee (TSC) will be convened to provide overall guidance for the study on behalf of the Study Sponsor (University of Oxford) and to ensure the study is conducted to the rigorous standards set out in the Department of Health's Research Governance Framework for Health and Social Care and the Guidelines for Good Clinical Practice. It should be noted that the day to day management of the trial is the responsibility of the Chief Investigator (Helen McShane). The Chief Investigator in conjunction with the study team is responsible for overseeing Trial management and progress. The TSC will comprise at least 3 independent members (including the Chair). The TSC will operate in accordance with the study-specific charter, which will be established before recruitment starts.

5.2 Data Safety Monitoring Board

An independent Data Safety Monitoring Board (DSMB) will be appointed to provide real-time safety oversight. There will be a minimum of 5 appropriately qualified committee members of whom one will be the designated chair. The DSMB will operate in accordance with the study-specific charter, which will be established before recruitment starts. In order for real time and transparent sharing of safety information, the members of this board will be the same as the DSMB committee overseeing the parallel naïve, unvaccinated SARS-Cov-2 challenge trial at ICL. The DSMB will be notified within 24 hours of the

investigators' being aware of the occurrence of SAEs in either challenge trial that are deemed possibly, probably or definitely related to challenge agent and/or study procedures. The board has the power to place the study on hold if deemed necessary following a study intervention-related SAE. All correspondence between the investigators and DSMB will be conveyed by the investigator to the study sponsor or their delegate. The Chair of the DSMB will be contacted for advice and independent review by the investigator or study sponsor in the following situations:

- Following any SAE deemed to be possibly, probably, or definitely related to the challenge agent or study procedures.
- After each dosing group to assess the safety of progressing to the next group at the same dose, lower dose or higher dose
- Review of data from a minimum of 4 unvaccinated individuals prior to dose escalation of unvaccinated individuals.
- After the dose finding Group 1 is complete and prior to Group 2 enrolment to determine the dose for Group 2 and the use of the following in groups 2, 3, and 4 as appropriate as detailed in this protocol:
    - Rescue therapy
    - CMR scans
    - Routine use of CT scans at D5 and 11 post infection

- After the first 10 volunteers have been enrolled into Group 2, and again (if applicable) after 20 volunteers, to determine if further enrolment is required into Group 2
- After the dose finding Group 3 is complete and prior to Group 4 enrolment to determine the dose for Group 4 and the use of the following, if not already confirmed following review of Group 1:
    - Rescue therapy
    - CMR scans
    - Routine use of CT scans at D5 and 11 post infection

- Any other situation where the investigator or study sponsor feels independent advice or review is important

The DSMB is expected to meet as per the above or more frequently if required.

6 STUDY DESIGN

This is a phase I dose escalation challenge study in which increasing titres of wild-type SARS-CoV-2 ($1x10^1$ $TCID_{50}$, $1x10^2$ $TCID_{50}$, $1x10^3$ $TCID_{50}$,$1x10^4$ $TCID_{50}$ and $1x10^5$ $TCID_{50}$) will be administered intranasally to different groups of volunteers in order to achieve a 50% (+/-10%) attack rate as determined by quantitative live viral detection and/or qPCR detection in nasal-oropharyngeal secretions at two consecutive 12 hourly time points (at least 24 hours after inoculation). A Data Safety Monitoring Board (DSMB) will review safety and quantitative virology at each dose level and will recommend continuation, dose escalation or de-escalation based on emergent data. Dose escalation will be capped at 10^5 TCID50 and we will proceed to dose confirmation, following DSMB safety review, even if we do not meet our target attack rate of 50% +/- 10%, to enable a larger sample size to assess the dynamic range of protection at that dose and ensure confidence in the negative infection rate at that dose. The DSMB committee members are the same as those overseeing the naïve SARS-CoV-2 challenge study being performed in

parallel at Imperial (See section 8.9 Dose escalation, de-escalation and confirmation for further information). If a higher dose is advised by DSMB in order to reach our target attack rate, approval will be sought with the REC. This would be submitted as a substantial amendment.

Rescue treatment with a 5 day course of oral antiviral tablets (paxlovid) will commence immediately after any warning symptoms or signs of COVID-19 disease beyond mild disease*. Once the optimal dose of wildtype SARS-CoV-2 has been identified for previously infected volunteers and uninfected vaccinated volunteers (dose escalation groups 1 and 3), further challenge infections in groups 2 and 4 may proceed without Paxlovid treatment (following DSMB review of infection rate, viral load and clinical data).

Volunteers will remain in isolation rooms within the clinical trials unit for a minimum of 14 days post inoculation and until demonstration of the absence of live virus in two sequential samples as per section 8.10.

All 4 groups will together enrol up to 132 volunteers. This study will be carried out in close collaboration with the parallel hVIVO dose escalation in seronegative volunteers.

* Note, prior to availability of paxlovid, treatment was a single infusion of 1200mg intravenous monoclonal antibody cocktail (REGN COV2).

6.1 Study groups

Volunteers will be recruited who are able and willing to attend regular screening and outpatient follow up visits at the Clinical Centre for Vaccinology and Tropical Medicine. They will be challenged and admitted for a quarantine period within rooms with appropriate ventilation to prevent spread of infection (negative pressure rooms or positive pressure ventilation lobby) located at the Experimental Medicine Clinical Research Facility (EMCRF) depending on bed availability. It is planned that a total of up to 132 participants will be enrolled in this study (Table 4, Table 5)*. The study will consist of 4 groups:

- Group 1: Dose escalation group to find the lowest possible safe dose that causes either asymptomatic or mild infection in 50% (+/-10%) of up to 8 participants (i.e. 3 or more participants) of previously SARS-CoV-2 infected individuals.
- Group 2: A dose confirmation group using the dose identified in group 1, to allow a larger cohort for confirmation of group 1 findings and data for end points. If a dose meeting our target attack rate is not found in group 1, this group will continue at the last highest safe dose identified after discussion with the DSMB, to enable a larger sample size to assess the dynamic range of protective immunity at that dose.
- Group 3: Dose escalation group to find the lowest possible safe dose that causes either asymptomatic or mild infection in 50% (+/-10%) of up to 8 participants (i.e. 3 or more participants) in previously uninfected vaccinated individuals.
- Group 4: A dose confirmation group using the dose identified in group 3, to allow a larger cohort for confirmation of group 3 findings and data for end points. If a dose meeting our target attack rate is not found in group 3, this group will continue at the last highest safe dose identified after discussion with the DSMB, to enable a larger sample size to assess the dynamic range of protective immunity at that dose.

For detailed information on the dose escalation process please see section 8.8.

Table 4: Study groups: Previously SARS CoV-2 infected participants

Group	Number of Volunteers	Dose	Route
1A	6-8	$1x10^1$ TCID50	Intranasal viral challenge
1B^	6-8^	$1x10^2$ TCID50	Intranasal viral challenge
1C^	6-8^	$1x10^3$ TCID50	Intranasal viral challenge
1D	4-8^	$1x10^4$ TCID50	Intranasal viral challenge
1E	4-8^	$1x10^5$ TCID50	Intranasal viral challenge
2	10-20 (with option of additional 10)*	Dose determined by group 1	Intranasal viral challenge

^ If unvaccinated volunteers are enrolled; a minimum of 4 unvaccinated individuals will need to be enrolled at each dose with none having evidence of infection at that dose prior to dose escalation for any unvaccinated volunteers. This may mean enrolling unvaccinated volunteers at a lower dose group after dose escalation has occurred in vaccinated volunteers. If an unvaccinated volunteer did develop infection then we would create an unvaccinated subgroup at that dose and dose escalation in unvaccinated individuals would only be able to occur after confirmation that the target attack rate of 50% (+/-10%) in up to 8 participants will not be met. If an unvaccinated subgroup is created for the sake of clarity we will amend the protocol accordingly to reflect this.

*Up to an additional 10 volunteers may be enrolled into Group 2 if a dose that meets our target attack rate is found. This would be decided after statistical review if larger sampling sizes were felt necessary to reach study objectives. See Section 8.8 and 11 for more details.

Table 5. Study groups: Uninfected SARS CoV-2 vaccinated individuals

Group	Number of Volunteers	Dose	Route
3A	4-8[x]	$1x10^2$ TCID50	Intranasal viral challenge
3B	4-8[x]	$1x10^3$ TCID50	Intranasal viral challenge
3C	4-8[x,^]	$1x10^4$ TCID50	Intranasal viral challenge
3D	4-8^	$1x10^5$ TCID50	Intranasal viral challenge

4	10-20<br>(with option of additional 10)*	Dose determined by group	Intranasal viral challenge

[x] A minimum of 4 individuals will need to be enrolled at each dose with none having been infected at that dose prior to dose escalation. If 1 or more of the first 4 volunteers develops infection, the group will be expanded.

*Up to an additional 10 volunteers may be enrolled into Group 4 if a dose that meets our target attack rate is found. This would be decided after statistical review if larger sampling sizes were felt necessary to reach study objectives. See Section 8.8 and 11 for more details.

6.2 Study volunteers

Study population will be males and non-pregnant or lactating females between 18 and 30 years with no underlying co-morbidities. Participants for groups 1 and 2 will need evidence of previous SARS-CoV-2 infection . Participants in groups 3 and 4 will need written or electronic evidence of vaccination against SARS-CoV-2. Eligibility will depend on review of medical and social history, physical examination, and the results of comprehensive testing including; laboratory tests, ECG, smell tests, chest x ray and pulmonary function testing. In addition, a QCOVID risk score will be calculated and the risk discussed with the volunteer prior to enrolment. Although an absolute cut-off will not be used to determine eligibility it will be used as part of a holistic assessment by study investigators when considering suitability of participants. Volunteers will be considered enrolled immediately after receiving the intranasal viral challenge.

6.3 Definition of Start and End of Study

The start of the study is defined as the date the first volunteer is recruited into the study (i.e. the date the first volunteer provides informed consent). The end of the study is the date of the Last Volunteer Last Visit (LVLV).

6.4 Potential Risks for volunteers

The potential risks to participants in this study are those associated with the SARS-CoV-2 infection and related complications and other study procedures.

6.4.1 SARS-CoV-2 challenge

The risk associated with this challenge are those related to developing COVID-19 disease and its associated complications. The natural course of SARS-CoV 2 infection and risk factors are outlined in the study background. As discussed, the risk in our study population is generally considered low. Furthermore, this study has been designed with the aim of developing an infection model with minimal or no disease.

Nevertheless, we will be prepared for participants to develop symptoms. COVID-19 disease can present as a spectrum of severity from mild to life threatening. Mild-moderate symptoms can include cough, fever (>37.8 ^{0}) C, loss or change in normal sense of taste or smell (anosmia & ageusia), myalgia, malaise, headache and sore throat. Some individuals also experience gastrointestinal symptoms including diarrhoea, nausea and vomiting.

Severe SARS-CoV-2 infections are known to occur rarely in both infants and adults. In adult populations, multiple factors including older age, are independently associated with severe SARS-CoV-2 complications including chronic co-morbidities and significant immune compromise. Severe infection typically results in development of COVID-19 associated pneumonia with associated dyspnoea, tachypnoea and hypoxia. In life threatening cases this can result in sepsis (and septic shock) and acute respiratory distress syndrome (ARDS).

Other rare but serious COVID-19 related sequelae are outlined in the study background but include myocarditis (inflammation of heart) and coagulopathy (including increased risk of thromboembolic events such as pulmonary embolism, deep vein thrombosis and acute strokes). These sequelae can be seen with any viral or bacterial infection. The risk of them occurring in COVID-19 disease is low but not negligible.

6.4.2 Chest radiography and lung CT scan

Each volunteer enrolled in the study will have a single chest X-ray at screening, both for research purposes to act as a baseline for further imaging that may be necessary and to ensure that no significant pulmonary infection or other respiratory pathology is present which would make them ineligible for the study. The estimated dose will be 0.02 mSv which is approximately equivalent to 6.8 days natural background radiation and carries risk of inducing a cancer of approximately 1:100,000-1,000,000 based on risk factors for a healthy adult. This is classified as a trivial risk level (ICRP 62).

A CT of the chest will be acquired in all participants at day 5. An additional CT may occur at day 11 in infected volunteers if deemed necessary by the investigators e.g. due to sequential positive PCR, ongoing live viral culture or ongoing symptoms, signs or investigations suspicious for SARS-CoV-2 infection. In order to limit unnecessary radiation exposure, scans will not be conducted in well volunteers with isolated non-consecutive PCR positivity who have demonstrated a normal CT scan at day 5. A low dose unenhanced volumetric CT scan will be performed for both CT scans. The patient will be positioned supine with their arms above their head and the scan performed with the patient at held full inspiration. The scan will be acquired from the lung apices to the costophrenic sulci to obtain full coverage of the lung parenchyma using a 0.625 mm section thickness and reconstructed using a lung algorithm. The estimated dose will be no more than 1.3mSv or less for each low dose scan. This equates to six months of natural background radiation, and a minimally increased cancer risk of 1 in 14,000 in the lifetime of the participant.

The total protocol dose (X-ray and up to two CT scans) is 2.62mSv. This is equivalent to 1 year of average background radiation in the UK. The risk of developing cancer as a consequence of taking part in this study is estimated as 0.01%, when the lifetime (all cause) risk of developing cancer is approximately 50%. This is classified as minor to intermediate risk (ICRP 62).

.

The likelihood of identifying clinically significant abnormalities in young, healthy individuals with normal pulmonary function tests prior to inoculation is low. We will therefore only perform CT post challenge and

use a screening chest x ray to look for evidence of underlying pulmonary disease prior to enrolment to further minimize any radiation dose.

6.4.3 Cardiovascular Magnetic Resonance (CMR) imaging and Gadolinium contrast use

MRI is a safe and non-invasive technique with no known risk when appropriately supervised. It does not involve ionising radiation. Potential participants with ferromagnetic objects in their bodies, or with implanted devices which can be damaged by the CMR magnet will be excluded from a CMR scan, but will instead get an echocardiogram to screen for structural heart disease. All participants entering the scanner room are screened for such objects. Gadolinium contrast is widely used for clinical indications in CMR and is safe. Occasionally it may cause a mild headache, nausea, itching and very rarely (1 in 5000) a more severe allergic reaction. It is cleared within hours by the body. Availability to treatment for anaphylaxis is readily available in the unit where these procedures are carried out and participants will be accompanied by staff appropriately trained in the management of anaphylaxis.

The requirement for continuation of CMR was reviewed by the DSMB following Group 1 In agreement with DSMB review, CMR will be ceased in all groups following SA009.

Known risks are outlined in Tables 5 and 6, however, there may be unexpected or unforeseen side effects (e.g. a severe allergy). Every effort will be made to monitor the health of the participants to ensure that such risks are minimised.

Table 6. Risks and mitigation strategies related to SARs-CoV-2 challenge

Potential risk	Rationale	Mitigation Strategy
**Inoculation of SARS-CoV-2**		
**SARS-CoV-2 Infection**	Typical SAR-CoV-2 illness: abrupt onset of fever, malaise, myalgia (muscle aches), alteration or loss of smell, cough and sore throat.	The safety profile of the SARS-CoV-2 in young healthy adults has been described in the Background to Research. SARS-CoV-2 infection in healthy young adults usually resolves without treatment within 11 to 14 days. Fatigue and loss of smell may be protracted but in almost all cases resolve completely. Participants will be continuously monitored by clinically trained study staff and will have access to appropriate symptomatic treatment (such as paracetamol) and onward referral to appropriate healthcare professionals if clinically necessary.
	Severe SARS-CoV-2 infections are known to occur rarely in both infants and adults. In adult populations, multiple factors including older age, are independently associated with severe SARS-CoV-2 complications including chronic co-morbidities and significant immune compromise. Severe infection typically results in development of	Strict inclusion and exclusion criteria, combined with stringent health screening, will apply to ensure only healthy young adults with no known risk factors for severe COVID-19 are enrolled in this study. Use of QCOVID living risk prediction tool as a guide to inform risk determination.<br>The study risk in the study population is generally considered low. The study has been designed with the

	COVID-19 associated pneumonia with associated dyspnoea, tachypnoea and hypoxia (low oxygen levels). In life threatening cases this can result in sepsis (and septic shock) and acute respiratory distress syndrome (ARDS).<br><br>Antibody dependent enhancement (ADE) in individuals who have previously been infected with SARS-CoV-2 resulting in more severe disease is a theoretical risk.	aim of developing an infection model with minimal or no disease.<br><br>Dose finding will occur in a cautious and staged manner, starting with the lowest infecting dose (10 TCID50). Dose escalations will only occur after a review of safety data with the DSMB. In the event that unvaccinated volunteers participate, caution will be applied to the dose group recruited to as detailed in section 6.1 in recognition of the fact unvaccinated individuals will have differing immunity to vaccinated individuals.<br><br>Participants will be continuously monitored at an inpatient quarantine unit to allow early recognition of features associated with progression to severe disease. All participants in Group 1 who develop warning symptoms or signs of COVID-19 disease in the dose escalation phase will be treated with a targeted combined monoclonal therapy (REGN COV2) or, once approved as part of SA007, paxlovid. If deemed necessary after safety review by the DSMB, paxlovid will also be given to volunteers in Group 2.<br><br>Trial investigators include senior ITU, infectious diseases and respiratory consultants who will be closely monitoring volunteers and making appropriate timely clinical decisions to ensure volunteer welfare.
	Transient increase in alanine aminotransferase (ALT) or aspartate aminotransferase (AST) without clinical presentation, with a good prognosis upon improvement of infection.	Strict inclusion and exclusion criteria, combined with stringent health screening, will apply to ensure no history of liver disease.<br>Liver function tests (LFTs) will be monitored throughout the study as per the SoA.<br>In the very rare case of any indication of liver damage/failure, the participant will be transferred promptly to NHS care.
	The study virus, like many viruses, can cause more substantial health issues such as myocarditis (inflammation or damage to the heart muscle). However, the chance of this resulting in serious or permanent changes is rare, as most cases are minor and resolve without any lasting changes.	Participants will be continuously monitored by qualified medical staff in the quarantine unit for at least 14 days post challenge.<br><br>ECG and cardiac enzyme lab testing will be performed regularly as per the SoA and at additional time points if clinically indicated.<br><br>Routine cardiac MRI/echocardiogram will be ceased following SA09, in agreement with DSMB.
	In rare circumstances there may be renal damage. Normally this gets better but permanent kidney failure may require dialysis or renal transplantation.	Strict inclusion and exclusion criteria, combined with stringent health screening, will apply to ensure no history of renal disease. Urine will be tested for protein, blood and glucose at screening and during the study as

		per the SoA. Urea and electrolytes (U&Es) will be monitored throughout the study as per the SoA.<br>In the very rare case of acute kidney injury, the participant will be transferred promptly for NHS care.
	Risk of coagulopathy (including increased risk of thromboembolic events such as pulmonary embolism, deep vein thrombosis and acute strokes). These sequelae can be seen with any viral or bacterial infection. The risk of them occurring in COVID 19 disease is low but not negligible.	Full blood count and coagulation profile checked at screening and participants with existing coagulopathies will be excluded. These parameters will continue to be closely monitored during the study as per the SoA.<br><br>Thromboembolus deterrent (TED) stockings will be fitted for all volunteers from D0 of quarantine. In addition, if VTE risk considered sufficiently high risk, daily prophylactic dose of subcutaneous dalteparin (Fragmin) injection will be administered. (see Section 8.4.2 for more details)<br><br>Volunteers will be encouraged to carry out daily gentle exercise during the quarantine period (e.g. walking around the isolation room; yoga, stretch exercises etc.)<br><br>Participants will be continuously monitored on the quarantine unit and transferred for prompt NHS care if necessary.
	Develop painful swelling of toes ("COVID toes"). This usually lasts 3 to 4 weeks.	Usually COVID toes resolves without treatment, In some cases, to reduce pain or itching a hydrocortisone cream can be applied to the affected area. If this fails to bring relief or if symptoms worsen, the participant may be referred for specialist care e.g dermatologist.
	Inflammation of the epididymis /testicles (Epididymo-orchitis) has been reported, more commonly in males who have severe disease	This inflammation is temporary and generally resolves with analgesics and antibiotics. If this developed in our volunteers, appropriate treatment would be given as per advise of an NHS specialist. It is not yet known if this would have any long term affect on male fertility if it did occur with SARS CoV-2 infection. In the case of mumps, approximately 25% of males who get mumps infection after puberty get testicular inflammation, and approximately 1 in 10 of those who get testicular swelling have a drop in sperm count. This is rarely enough to cause infertility.
**Transmission of SAR-COV-2 to participants' close contacts**	SARS-CoV-2 virus in nasal secretions can cause infection in close contacts.	Viable virus will be shown to be absent from the nose by the time participants are discharged from quarantine. This will be confirmed by at least two consecutive negative nasal samples to determine participants' suitability for departure.
	Passing the SARS-CoV-2 Challenge Virus to others, including vulnerable people*	To reduce the risk, participants will be excluded from the study if they live with anyone considered clinically vulnerable to SARS-CoV-2 infection.
	Passing the SARS-CoV-2 Challenge Virus to study staff.	Clinic staff in contact with participants in the quarantine unit will be required to wear Personal Protective Equipment (PPE) as per local SOPs, to both

		avoid transmission of the SARS-CoV-2 Challenge Virus to study staff and prevent cross-contamination of participants.<br><br>Robust infection control measures will be implemented to prevent transmission, including disinfection of equipment, sample dispatch, infection control instructions to volunteer concerning their belongings.

*Note for the purposes of this study "vulnerable people" are defined as anyone defined as extremely clinically vulnerable to COVID-19 disease (ie previously shielding under Public Health England (now UK HSA) guidelines).

Table 7. Risks associated with other Study procedures

**Study Procedures**		
**Venepuncture/ blood sampling**	Localised bruising and discomfort at venepuncture site	Venepuncture performed by trained professionals. Inadequate venous access for requirements of study is an exclusion criterion.
	Syncope (fainting) can occur with venepuncture or even prior to needle insertion (pyschogenic response).	Blood samples obtained by trained professionals. Will be undertaken in appropriate setting to manage syncope if does occur.
	Anaemia – from blood collection.	Total volume of blood collected over 12 months is 838.5ml (+up to an additional maximum total of 125ml if longer than 17 days in quarantine and/or any unscheduled visits; see SoA). This should not compromise these otherwise healthy volunteers, as they would donate 470ml during a single blood donation for the National Blood transfusion Service over a 3-4 month period. Volunteers will be asked to refrain from blood donation for the duration of their involvement in the study. Participants with anaemia at screening will not be eligible to take part. Anaemia will be monitored for during the study and the decision to take research bloods will always be considered secondary to participant safety.
**Mid turbinate, nasopharyngeal and oropharyngeal swabs for viral detection and transcriptomics**	**Oropharyngeal** swab can be uncomfortable and cause gagging. **Nasopharyngeal and mid turbinate** swab procedures can cause discomfort, eye watering, sneezing, nasal irritation and nose bleeds.	Procedure will be performed by trained study staff. Individuals with significant nasal abnormalities or increased bleeding risk will not be eligible for enrolment.
**Nasosorption**	Collection of nasal strips samples may cause discomfort, sneezing, eye watering, or more rarely nasal irritation or nose bleeding.	Procedure will be performed by trained study staff. Individuals with significant nasal abnormalities will not be eligible for enrolment.

**Study Quarantine**	There is a risk that participants become anxious and/or depressed during the isolation period.	Strict inclusion and exclusion criteria, combined with stringent health screening makes this scenario less likely.<br>Patient Health Questionnaire (PHQ-9) and Generalised Anxiety Disorder (GAD-7) Questionnaire carried out prior to enrolment and at regular intervals throughout the quarantine period as per SoA.<br>Before enrolment into the study, staff will highlight the mental strain the study and quarantine period in particular may place on a participants mental health status and will encourage volunteers to consider this prior to agreeing to participate. Staff will discuss with volunteers their plans for activities during quarantine and strategies to promote mental well-being during the quarantine period. Suggested free online activities and resources (e.g. virtual museum exhibitions, online learning) will be shared with the volunteer including links to wellbeing and mental health resources. Volunteers will be strongly encouraged to exercise during quarantine and will be allowed to bring in basic exercise equipment such as mats and hand weights.<br>Participants will be visited frequently by the study staff to carry out the daily procedures.<br>Participants will have a diary showing what procedures are being performed each day, so they know what to expect and so that they can plan their daily routines.<br>Participants can bring their own mobile phone and laptop, and watch TV and films.<br>There will be access to a good WIFI and participants will be able to contact their friends/relatives via video call or similar.<br>Mental health questionnaires will be used to monitor mental health status and enable more general discussions about participants concerns and wellbeing.<br><br>If during the quarantine period the mental health status of the participant becomes of clinical concern, the study team will consider the need for further mitigating action for safety purposes (e.g. modification to procedures, safe withdrawal from trial and referral to specialist mental health assessment if in participants best interest.
	Risk of coagulopathy due to prolonged isolation in one room. Risk is increased if volunteers develop an infection (such as COVID-19)	Full blood count and coagulation profile checked at screening and during the study as per the SoA.<br><br>Thromboembolus deterrent (TED) stockings will be fitted for all volunteers from D0 of quarantine. In addition, if VTE risk considered sufficiently high risk, daily prophylactic dose of subcutaneous dalteparin (Fragmin) injection will be administered. (see Section 8.4.2 for more details)

		Participant will be encouraged to carry out daily gentle exercise during the quarantine period (e.g. walking around the isolation room; yoga, stretch exercises etc.)<br><br>Participants will be continuously monitored on the quarantine unit and transferred for prompt NHS care if necessary.
**Study therapeutics**		
**Intravenous REGN-COV2 rescue therapy**<br><br>**Note this is based on known adverse reactions as per IB version 5 safety data cut off date 27 January 2021. For up to date adverse reactions to this product please refer to the latest SmPC found at Summary of Product Characteristics for Ronapreve - GOV.UK (www.gov.uk) [1,2]**	Local signs and symptoms associated with the cannula insertion (peripheral access) and drug administration may include erythema, swelling/induration, phlebitis, haematoma and pain/tenderness at the insertion site.	Local reactions will be monitored but are generally short-term and do not require treatment. Aseptic technique used for cannula insertion and hypoallergenic dressings.
	Systemic hypersensitivity reactions & acute infusion reactions; symptoms can include fever, chills, nausea, vomiting, flushing, abdominal pain, chest tightness, headache, dyspnoea (shortness of breath), hypotension, angioedema, throat irritation, urticarial rash, pruritus, myalgia, and/or dizziness.<br><br>Infusion & hypersensitivity reactions in published data are rare (1.8% with 2.4g dose, 3.1% in 8g dose).	A study clinician will be present for administration. Emergency equipment and medication for the treatment of acute infusion/ hypersensitivity reactions (e.g., antihistamines, bronchodilators, IV fluids, corticosteroids, paracetamol and adrenaline) will be available for immediate use. In addition, immediate assistance will be available from the NHS on-site emergency clinical team in the small likelihood that emergency treatment is required.<br><br>If any signs or symptoms of a possible hypersensitivity reaction are noted the infusion will be stopped and participant managed symptomatically. Once symptoms have resolved and at the clinician's discretion infusions may be restarted at 50% the original rate as described in the REGN COV2 IB.<br><br>Infusions will be terminated completely if any of the following are encountered: anaphylaxis, laryngeal/pharyngeal oedema, severe bronchospasm, chest pain, seizure, severe hypotension, other neurological symptoms (e.g. confusion, loss of consciousness, paraesthesia, paralysis, etc.).
	Lack of reproductive and developmental toxicology studies in males or females. Theoretical risk that mABs will cross placental barrier (human IgG1 antibodies do). It is therefore not known whether this would provide a treatment benefit or risk to a developing foetus.	Females who are pregnant, lactating or trying to conceive will not be eligible to take part in the study. Participants will be advised to use an appropriate (highly effective) contraceptive method for the duration of the study (females) or for 6 months from Regeneron dose (males). Women of child bearing potential will have a pregnancy test at both screening visit and prior to SARS-CoV-2 infection.
	Potential risk of immunogenicity with an impact on efficacy/safety (as with all protein therapeutics)	Analysis from stored serum if required. (Note this serum is already planned for collection as per SoA as part of exploratory immunology).

**Oral Paxlovid rescue therapy**<br>**For up to date adverse reactions to this product please refer to the latest SmPC found at Summary of Product Characteristics for Paxlovid - GOV.UK (www.gov.uk)** [1,2]	Paxlovid inhibits the CYP3A4 metabolic pathway; associated risk of interaction with medicinal products that are dependent on CYP3A for clearance.<br>Use of these products is contraindicated with paxlovid due to potential reactions from elevated plasma concentrations.<br><br>Additional risk of interaction with medicinal products that are potent CYP3A inducers, thus inducing Paxlovid's metabolic pathway.<br>Use of these products is contraindicated with paxlovid due to risk of significantly reduced plasma PF-07321332/ritonavir concentrations, rendering the rescue therapy less effective.	Volunteers who are on many of the contraindicated medications will not be eligible to take part in the study or where appropriate will have stopped taking these with an appropriate washout period.<br>Any women of child bearing potential taking the combined oral contraceptive pill will be required to use additional appropriate barrier contraception with spermicide for 30 days after stopping treatment.<br>All acute medication prescriptions commenced during the quarantine period should have potential interactions checked using the Liverpool COVID-19 drug interactions checker.
	Potential risk of adverse reaction the excipients in oral paxlovid tablets, particularly lactose.	Volunteers will be asked ahead of treatment if they have any known allergies,<br>If any signs or symptoms of a possible hypersensitivity reaction are noted, the treatment will be stopped and participant managed symptomatically.
	Adverse reactions may include dysgeusia, diarrhea and vomiting	If any signs or symptoms of a possible reaction are noted, the treatment will be stopped and participant managed symptomatically.
	Lack of reproductive and developmental toxicology studies in males or females. It is therefore not known whether this would provide a treatment benefit or risk to a developing foetus.	Females who are pregnant, lactating or trying to conceive will not be eligible to take part in the study. Participants will be advised to use an appropriate (highly effective) contraceptive method for the duration of the study (females) and, where Paxlovid rescue therapy is used, must agree to also use a barrier method of contraception with spermicide for 30 days after stopping Paxlovid. Women of child bearing potential will have a pregnancy test at both screening visit and prior to SARS-CoV-2 infection.
**Prophylactic anticoagulation with low molecular weight**	This can commonly cause mild bruising and discomfort at subcutaneous injection site.	Subcutaneous LMWH will be administered by trained professionals.
	Haemorrhage	Risk of haemorrhage is low with prophylactic dose in otherwise healthy individuals. Risk of serious haemorrhage is very uncommon. Participants will only

**heparin (LMWH) – (to be used if required in a subset of symptomatic volunteers see Section 8.4.2)**		receive LMWH on quarantine unit where they are monitored continuously. If bleeding occurs – it is usually sufficient to stop LMWH for it to resolve. However, LMWH can also be partially reversed by protamine sulphate.
	Heparin Induced thrombocytopenia – 50% decrease in platelet count approximately 5-10 days after starting heparin due to heparin-dependent, platelet-activating IgG antibodies. Promotes a prothrombotic state - occurs in 1 in 5000 hospitalised patients. Uncommon.	Uncommon side effect, particularly in otherwise well individuals. Safety bloods will be monitored during quarantine phase of study and if thrombocytopenia noted LMWH would be stopped. Further action undertaken depending on clinical necessity.
**Thrombo-embolus deterrent stockings (TEDS)**	Pressure damage if stockings are too tight – signs of this include numbness, tingling, pins & needles, pain or soreness in the foot or leg; pale/cool/discoloured foot or leg.	Participant will be measured for the correct size of TED stockings and will be instructed in the correct use and potential problems related to TED stockings. Clinical team to observe for any complications daily.
	Contact allergy to the elastic fibres in the stockings. Immediate reactions. (e.g., urticaria) or delayed reactions (contact eczema) to the materials commonly used today in compression stockings, elastane and polyamide are rare. Other components such as cotton and natural rubber latex are used increasingly less often in compression stockings.	History of known allergies will be collected at screening. In case of allergy to the elastic fibres in the stockings, an alternative product (TED stocking) will be requested on case-by-case basis

1. If the SmPC is updated during the study then it will be reviewed to determine whether the protocol needs revising (for example, there may be the need to change eligibility criteria, con meds etc) and/or the risk:benefit for the study has changed.

2, Once paxlovid is fully approved following SA007, REGN COV2 will be discontinued as rescue therapy

6.5 Known Potential Benefits

Volunteers are not expected to benefit directly from participation in this study. Volunteers will gain some information about their general health as a result of the screening, examination, blood tests, urine tests, ECG, and chest imaging (Chest x-ray and/or CT if required).

It is hoped that their contribution will further understanding about protective immunity against SARS-CoV-2 infection. Furthermore, such a model can also be used to evaluate novel candidate vaccines, diagnostics and therapeutics.

7 RECRUITMENT AND WITHDRAWAL OF STUDY VOLUNTEERS

7.1 Identification of Study Volunteers

Volunteers will be recruited by use of an advertisement +/- registration form formally approved by the ethics committee(s) and distributed or posted in the following places:

- In public places, including buses and trains, with the agreement of the owner/ proprietor
- In literature for circulation
- Banner adverts online (e.g. Google advertising)
- Postage mailouts, including directly from GP practices where appropriate and agreed
- Text messaging services to potentially eligible participants when agreed with the owner of the database (i.e. local GP practices)
- On a website or social media site operated by our group or with the agreement of the owner or operator (including on-line recruitment through our website)
- By e-mail distribution to a group or list only with the express agreement of the network administrator or with equivalent authorisation
- By email distribution to individuals who have already expressed an interest in taking part in any clinical trial at the Oxford Vaccine Centre
- On stalls or stands at exhibitions or fairs
- Via presentations (e.g. presentations at lectures or invited seminars)
- Oxford Vaccine Centre databases: We may contact individuals from databases within the CCVTM (including the Oxford Vaccine Centre database) of previous trial participants who have expressed an interest in receiving information about all future studies for which they may be eligible
- The above methods may also be used within GP practices in England, where appropriate

Potential volunteers who express an interest in the study will be asked to fill in an online pre-screening questionnaire (further details in Clinical Procedures-Study visits) and may be contacted by phone or email by a member of the study team (clinician or nurse) prior to being formally invited for a screening visit.

7.2 Informed consent

In order to allow sufficient time for prospective participants to fully consider the PIS and what participation in the study entails the pre-screening and screening phase will be split across several encounters fully described in sections 8.6.1 and 8.6.2. After completing a pre-screening questionnaire (detailed in section 8.6.1) participants will be sent study participant information sheets and pre-recorded video and allowed at least 72 hours to read and consider these. They will then have a video call appointment with a study clinician (with only audio recorded), where the participant is able to ask any questions. Following this, individuals who remain interested in the study will be invited to an in-person screening visit a minimum of 48 hours after the video pre-screening appointment.

The final informed consent process will take place at the CCVTM, Oxford. Volunteers will be given additional opportunity to discuss the study and ask any further questions.

Across both the pre-screening video appointment and the screening visit the volunteers will be fully informed of all aspects of the study, the known side effects, potential risks and their obligations. The following general principles will be emphasised:

- Participation in the study is entirely voluntary
- Refusal to participate involves no penalty or loss of medical benefits

- The volunteers may withdraw from the study at any time, although we would strongly advise against leaving the quarantine facility prematurely both for their personal safety and to minimise risk of infection to household contacts.
- If a volunteer does leave the quarantine facility prior to clearance of live virus they will be advised to self-isolate as per current government guidance.
- The volunteers are free to ask questions at any time to allow them to understand the purpose of the study and the procedures involved
- There is no direct benefit to the volunteer from participating
- The volunteer will be registered on the TOPS database (The Over-volunteering Prevention System; www.tops.org.uk)
- The volunteer's GP will be contacted to corroborate their medical history. Volunteers will only be enrolled in the study if written information regarding the volunteer's medical history is obtained from the GP. This can either be via the study team accessing patient's electronic care summaries from local systems, by contacting the GP or volunteers bringing their medical care summaries from the GP to the study clinicians.
- Additionally, confirmation of prior SARS-CoV-2 infection or vaccination will need to be obtained. We may contact UK HSA to corroborate this or obtain further information.
- If agreed to, samples taken as part of the study may be sent outside of the UK and Europe to laboratories in collaboration with the University of Oxford. These will be pseudo-anonymised using a participant number which can be linked back to the participant's identifiable details by study staff only and not by any other agencies.
- Volunteers will undergo a separate, optional consent process (under the Oxford Vaccine Centre Biobank protocol) regarding indefinite storage of any leftover samples for use in other ethically approved research.
- Agree to be tested for SARS-CoV-2 infection and understand that results will need to be notified to the relevant public health authority.
- Agree to spend an inpatient quarantine period (at least 17 days) in an isolation room after the intranasal challenge, where the study team will be able to monitor any COVID-19 symptoms.

The aims of the study and all tests to be carried out will be explained. The volunteer will be given the opportunity to ask about details of the study, and will then have time to consider whether or not to participate. The study team will conduct a short questionnaire to make sure the volunteers have understood the study rationale, procedures and risks. Incorrect answers will prompt re-review of the relevant informed consent sections with the volunteer. Volunteers will then be asked to repeat the questionnaire. If volunteers do not answer all questions correctly on the second attempt they will be excluded (informed consent not obtainable). If they do decide to participate written Informed Consent will then be obtained by means of participant-dated signature and dated signature of the person who presented and obtained the Informed Consent. The person who obtained the consent must be suitably qualified and experienced and have been authorised to do so by the chief/Principal Investigator. A copy of the signed Informed Consent will be given to the participant. The original signed form will be retained at the study site in the case report form (CRF). Additionally, volunteers will be asked to initial the sections of the information sheet to confirm all details have been read, understood and that they have had the opportunity to ask questions. A copy of the signed information sheet will be retained by study staff at the study site in the case report form (CRF).

All volunteers will personally sign and date the informed consent form before any study specific procedures are performed. The participant will be allowed as much time as wished to consider the information, and the opportunity to question the Investigator, their GP or other independent parties to decide whether they will participate in the study.

7.3 Inclusion and exclusion criteria

This study will be conducted in healthy adults aged 18-30 years at the time of enrolment, who meet the following inclusion and exclusion criteria:

7.3.1 Inclusion Criteria

The volunteer must satisfy all the following criteria to be eligible for the study:

1) Aged 18-30 years on proposed date of enrolment.
2) Body Mass Index (BMI) ≥18.5 kg/m^2 and ≤28 kg/m^2.
3) In good health with no history of clinically significant medical conditions (as described in Exclusion criteria) that would interfere with subject safety, as defined by medical history, physical examination, routine laboratory tests, ECG, pulmonary function tests and Chest X-Ray as determined by the Investigator at a screening evaluation.
4) Volunteer is willing and able to give written informed consent for participation in the study
5) Willing to allow the investigators to discuss the volunteer's medical history with their General Practitioner or any relevant health authority
6) Allow the investigator to register volunteer details with a confidential database (The Over-volunteering Prevention Service) to prevent concurrent entry into clinical studies/trials
7) Agreement to refrain from blood donation during the course of the study
8) a. For women of child bearing potential (WOCBP), a willingness to practice continuous effective contraception (see below) during the study and, a negative pregnancy test on the day(s) of screening and challenge
   b. For women of child bearing potential (WOCBP) taking the combined oral contraceptive pill, a willingness to use barrier contraception with spermicide during treatment with Paxlovid and for 30 days after completing Paxlovid treatment (should they receive it).
9) Able and willing (in the investigator's opinion) to comply with all study requirements
10) No clinically relevant findings in medical history or on physical examination in the opinion of a clinically qualified investigator, in discussion with the CI if needed
11) For Groups 1 & 2: Previous microbiological confirmation of SARS-CoV-2 infection > 3 months prior to enrolment (Proof of positive PCR or lateral flow antigen test confirmed via medical notes/ or UK HSA or a history from a volunteer consistent with SARS-CoV-2 infection with other evidence of this infection such as a photograph of a positive lateral flow test on the volunteer's phone or similar). OR serological confirmation such as positive anti-nucleocapsid IgG serology (unless this is explainable by prior vaccination) with the most recent history of symptoms or exposure likely to represent SARS-CoV-2 infection having occurred > 3 months prior to enrolment*.
12) For Groups 3 & 4: Written or electronic evidence of at least one vaccination against SARS-CoV-2 >21 days prior to enrolment (proof required would include written or electronic evidence from GP/ medical records or electronic NHS COVID pass).

*Where no history of symptoms or exposure can be identified to determine the timing of SARS-CoV-2 infection, volunteers may be enrolled >7 weeks from the identification of anti-nucleocapsid positivity and >3 months from their last negative anti-nucleocapsid antibody test

7.3.2 Exclusion Criteria

The volunteer may not enter the study if any of the following apply:

1) History or evidence of any clinically significant or currently active cardiovascular, (including thromboembolic events), respiratory (excluding SARS-CoV-2 infection), dermatological, gastrointestinal, endocrine, haematological, hepatic, immunological, rheumatological, metabolic, urological, renal, neurological or psychiatric illness. Specifically:

   a) Volunteers with any history of physician diagnosed and/or objective test confirmed asthma, chronic obstructive pulmonary disease, pulmonary hypertension, reactive airway disease, or chronic lung condition of any aetiology or who have experienced:

vii) Significant/severe wheeze in the past
viii) Clinically significant respiratory symptoms, including wheeze, which has ever resulted in hospitalisation
ix) Known bronchial hyper reactivity to viruses

   b) History of thromboembolic, cardiovascular or cerebrovascular disease
   c) History or evidence of diabetes mellitus (Type I or Type II)
   d) Any concurrent serious illness including history of malignancy that could interfere with the aims of the study or a subject completing the study. Basal cell carcinoma within 5 years of treatment or with evidence of recurrence is also an exclusion.
   e) Migraine with associated neurological symptoms such as hemiplegia or vision loss. Cluster headache/migraine or prophylactic treatment for migraine
   f) History or evidence of clinically significant autoimmune disease or known immunodeficiency of any cause (including HIV).
   g) History of severe psychiatric illness at any time (e.g. inpatient stay, psychosis) or current significant active symptoms of anxiety and/or depression or significant claustrophobia. Consider exclusion in the following cases*:
      i) Volunteers with history of anxiety-related symptoms of any severity within the last 2 years if the Generalized Anxiety Disorder-7 score is ≥5
      ii) Volunteers with a history of depression of any severity within the last 2 years if the Patient Health Questionnaire-9 score is ≥4
      iii) Significant claustrophobia
   h) Bleeding disorder (e.g. factor deficiency, coagulopathy or platelet disorder), or prior history of significant bleeding or bruising following injections or venepuncture.

i) Other major disease that, in the opinion of the Investigator, could interfere with a subject completing the study and necessary investigations.

2) Clinically significant smoking history. Defined as: Current smoker (any smoking including e-cigarettes in the last 3 months) or > 2 pack year smoking history at any time (2 pack years is equivalent to 20 cigarettes daily for 2 years), or use of any nicotine containing products on more than one occasion within the last 3 months.
3) History or presence of alcohol addiction, or excessive use of alcohol (average weekly intake in excess of 28 units alcohol; one unit being a half glass of beer, a small glass of wine or a measure of spirits)
4) Clinically significant history of use of drugs of misuse, with evidence of a negative drugs of misuse urine test required at screening and quarantine admission
5) History of anaphylaxis or any allergy likely to be worsened by any component of the study agent or proposed treatment regime.
6) Clinically active rhinitis (including hay fever) or history of moderate to severe rhinitis, or history of seasonal allergic rhinitis likely to be active at time of inclusion into the study and/or requiring regular nasal corticosteroids on at least weekly basis, within 30 days of admission to quarantine.
7) Any significant abnormality altering the anatomy of the nose or nasopharynx, clinically significant history of epistaxis (nose bleeds) or any nasal or sinus surgery within six months of inoculation
8) Clinical, radiological, or laboratory evidence of current active TB disease or latent TB infection
9) Previous VZV pneumonia
10) Positive HBsAg, HCV or HIV antibodies
11) Concurrent use of oral, inhaled or systemic steroid medication or use within the last 6 months (steroids used as a cream or ointment are permissible), or the use of other immunosuppressive agents concurrently or within the last 6 months.
12) Concurrent use of medication contraindicated for use with Paxlovid rescue therapy
13) Administration of immunoglobulins and/or any blood products within the three months preceding the planned study challenge date
14) Current use of any medication or other drug taken through the nasal or inhaled route including cocaine or other recreational drugs
15) Plans to receive a live vaccination 30 days prior to enrolment, or any vaccination (i.e. non-live, including a SARS-CoV-2 vaccine) 21 days prior to enrolment and/or plans to take any vaccination 30 days following enrolment
16) Current pregnancy or pregnancy within the last 6 months, lactation or intention to become pregnant during study period
17) Shares a household with someone with clinically significant immunodeficiency (due to underlying medical condition, medication or pregnancy); or who is extremely clinically vulnerable (previously shielding under Public Health England (now UK HSA) guidelines).

18) Concurrent participation in any other research trial which involves receipt of an investigational product or drawing of blood. Additionally participants must not have had an investigational product within the 30 days preceding enrolment into COV-CHIM01.
19) Laboratory confirmed (PCR or lateral flow antigen test) SARS-Cov-2 infection, evidence of viral pneumonitis on chest radiograph or a high clinical suspicion of COVID-19 disease in the 3 months preceding enrolment.
20) Post COVID-19 symptoms that have not resolved by 1 month prior to enrolment.
21) Previous hospitalisation with COVID-19 disease or related complications e.g. pulmonary fibrosis on chest x ray
22) Family history of 1st degree relative aged 50 years or less with sudden cardiac or unexplained death
23) Family history of severe COVID-19 disease or response to any other viral disease e.g. Guillain-Barré
24) Family history unavailable or in opinion of investigators not sufficient to assess criteria 22 and 23.
25) Clinically significant abnormality on screening chest radiograph
26) Clinically significant abnormality of lung function testing
27) Any clinically significant abnormality of screening blood or urine tests
28) Any other significant disease, disorder, or finding, which, in the opinion of the investigator, may either put the volunteer at risk, affect the volunteer's ability to participate in the study or impair interpretation of the study data
29) Venous access deemed inadequate for the phlebotomy and cannulation demands of the study.

For Groups 3 & 4:

30) Previous positive test for SARS CoV-2 infection (PCR or lateral flow antigen test) or a high clinical suspicion of COVID-19 disease at any time.
31) Positive Anti-nucleocapsid IgG at screening, unless explainable by vaccination

Volunteers who are excluded from the study because they have been discovered during screening procedures to be suffering from a previously undiagnosed condition thought to require further medical attention will, with their consent, be referred appropriately to their GP or an NHS specialist service for further investigation and treatment.

** Potential participants may be included with higher PHQ-9 or GAD-7 scores if there is an alternative explanation for these scores and study clinician and participant believe that their mental health will not be significantly worsened by quarantine. E.g. a participant with ADHD who has symptoms of hyperactivity but feels their mood is good and has previously tolerated isolation well. Where the scores exceed those outlined in the exclusion criteria a detailed explanation of why the participant is suitable for enrolment will be recorded.*

7.3.3 Effective contraception for volunteers

Female volunteers

Women of child bearing potential (WOCBP)* are required to practice a highly effective form of contraception during the course of the study. Acceptable forms of contraception for female volunteers include:

a. Established use of oral, injected or implanted hormonal methods of contraception (established for a minimum of 4 weeks prior to first inpatient quarantine).
b. Placement of an intrauterine device (IUD) or intrauterine system (IUS)
c. Male sterilisation, if the vasectomised partner is the sole partner for the participant and appropriate post vasectomy documentation of sterilisation success is available.
d. Same sex intercourse only
e. True abstinence from heterosexual intercourse, when this is in line with the preferred and usual lifestyle of the participant (Periodic abstinence and withdrawal are not acceptable methods of contraception). Careful history and documentation regarding the reliability of this method must be recorded.

* WOCBP are defined as women who are fertile following menarche until becoming postmenopausal, unless permanently sterile. A postmenopausal state is defined as no menses for 12 months without an alternative medical cause.

Permanent sterility includes total abdominal hysterectomy, bilateral salpingectomy (removal of both the Fallopian tubes) bilateral oophorectomy (removal of both the ovaries) laparoscopic sterilisation / tubal occlusion.

In addition to the contraceptive requirements above, WOCBP using combined oral contraceptives as their primary method of contraception must agree that they will also use a barrier method of contraception with spermicide should they receive paxlovid rescue therapy. The barrier method will be used for 30 days after stopping Paxlovid.

7.3.4 Challenge Postponement Criteria

Challenge will not proceed on the scheduled day in any of the following situations:

- The volunteer has had a recent upper respiratory tract infection (must be symptom-free for a full 7 days)
- Nasopharyngeal colonisation of respiratory pathogen at admission (as detected on BioFire test)
- The volunteer has a temperature > 37.5^{o}C
- The investigator judges the volunteer to have an acute moderate or severe illness (whether febrile or not)
- The volunteer has been notified of a significant exposure to a SARS-CoV-2 positive individual and should be self-isolating as per current government guidance.

- The volunteer has received a live vaccine within the preceding 30 days or a non-live vaccine (including SARS-CoV-2 vaccine) within the preceding 21 days or has plans to receive a vaccine in the following 30 days.
- The investigator has any other concern that challenge may not be in the volunteer's best interests
- Evidence of recent SARS CoV-2 infection (<3 months) based on a suggestive clinical history or investigations such as repeat serology, lateral flow antigen tests or PCR. Volunteers in group 3 & 4 could be re-enrolled, if appropriate, into group 1 or 2 as per the inclusion criteria.

In these cases, the volunteer may be challenged at a later date or withdrawn from the study at the discretion of the investigator.

In addition critical care capacity in the local NHS hospital (OUH) will be confirmed prior to enrolment. The CI has been granted access to the regional ICU network data for Thames Valley and Wessex. If this demonstrates concern regarding capacity issues (i.e. Critcon above usual winter pressure levels) then she will be in contact with both the Network Manager (Adult Critical Care), currently Kujan Paramanantham, regarding regional capacity and the Oxford ICU Senior Clinical and Nursing team to confirm capacity prior to enrolment. The CI, or someone from her team will assess capacity at 7 days and 2-3 days prior to enrolment. Volunteers will be deferred if there is not sufficient capacity. This is a precautionary measure in the unlikely event a participant requires hospitalisation due to a severe unforeseen adverse reaction. Bed capacity is not anticipated to be a recurring issue however this policy has been put in place as a safe guarding measure due to the current fluctuating bed pressures with the pandemic.

7.4 Prevention of 'Over Volunteering'

Volunteers will be excluded from the study if they are participating in any other research trial which involves receipt of an investigational product or drawing of blood or if they have received an investigational product within the 30 days preceding enrolment into COV-CHIM01. In order to ensure this, volunteers will be asked to provide their National Insurance or Passport number (if they are not entitled to a NI number) and will be registered on a national database of participants in clinical trials (www.tops.org.uk). They will not be enrolled if found to be actively registered on another trial.

7.5 Withdrawal of Volunteers

Every reasonable effort will be made to maintain protocol compliance and participation in the study.

Any subject can withdraw from the study at any time if they wish. Participants will be counselled that early withdrawal from the viral challenge phase of the study is strongly discouraged, as it may pose a risk both to the participant and their contacts. In the event of a participant insisting on early withdrawal during the challenge quarantine period, the participant will be encouraged to stay in the facility for isolation purposes and would be advised of the potential risks of carrying SARS-CoV-2 infection into the community, and to vulnerable groups in particular. If they consent to continued isolation at the quarantine facility after withdrawal from the study then any further procedures for research purposes will not occur, however, any procedures performed for safety purposes will continue to be offered.

However, if withdrawal occurs and the participant wants to leave the facility after virus inoculation prior to confirmation of minimal infectiousness, every effort will be made to manage their withdrawal and do the following:

- The participant will be counselled about the risk of onward transmission of SARS-CoV-2 and advised to self-isolate according to UK HSA and government guidance at the time.
- The patient will be counselled about any risks due to the withdrawal and specifically any risks due to missed safety monitoring that cannot be performed at home
- The participant will be reminded about infection control procedures and receive re-training if necessary, in handwashing and isolation rules
- The participant will be informed that they will not be able to receive the rescue therapy if they do not stay in the quarantine unit (if withdrawal is prior to this and eligible for the treatment as per protocol) and the potential pros and cons of this.
- The participant will be transported home in private transport with appropriate PPE, and in accordance with national guidance.
- Daily follow-up calls will be proffered to check on participant's health and remind them of self-isolation requirements until the end of the quarantine period and until study investigators are satisfied that daily follow up can end.

In accordance with the principles of the current revision of the Declaration of Helsinki and any other applicable regulations, a volunteer has the right to withdraw from the study at any time and for any reason, and is not obliged to give his or her reasons for doing so. The investigator may withdraw the volunteer at any time in the interests of the volunteer's health and well-being (including on the advice of the DSMB). In addition, the volunteer may withdraw/be withdrawn for any of the following reasons:

- Administrative decision by the investigator
- Ineligibility (either arising during the study or retrospectively, having been overlooked at screening)
- Significant protocol deviation
- Volunteer non-compliance with study requirements
- An AE, which requires discontinuation of the study involvement or results in inability to continue to comply with study procedures
- Confirmed pregnancy during the study

The reason for withdrawal will be recorded in the Case Report Form (CRF). If withdrawal is due to an AE, appropriate follow-up visits or medical care will be arranged, with the agreement of the volunteer, until the AE has resolved, stabilised or a non-study related causality has been assigned. Any volunteer who is withdrawn from the study may be replaced at the decision of the investigator. The DSMB may recommend withdrawal of volunteers. Any volunteer who fails to engage with study follow up will be deemed to have withdrawn from the study e.g. failure to attend consecutive follow-up visits, uncontactable despite active attempts to contact volunteer by study team (including contact via their next of kin).

If a volunteer withdraws from the study, samples and data collected before their withdrawal from the study will be used/stored unless the volunteer specifically requests otherwise. Long-term safety data collection will continue as appropriate if a volunteer has received a challenge dose.

7.6 Compliance with Dosing Regime

The volunteers will receive a single infection dose with wild-type SARS-CoV-2 only at enrolment (challenge day).

7.7 Pregnancy

Should a volunteer become pregnant during the study, she will be followed up for clinical safety assessment with her ongoing consent and in addition will be followed until pregnancy outcome is determined. We would not routinely perform venepuncture on a pregnant volunteer unless there is clinical need. Additionally, further investigations will be at the discretion of the investigator depending on clinical need. For example, CT imaging would not routinely be performed on pregnant volunteers unless benefits were felt to outweigh risks.

Abnormal pregnancy outcomes (e.g., spontaneous abortion, foetal death, stillbirth, congenital anomalies, ectopic pregnancy) are considered SAEs.

8. CLINICAL PROCEDURES

This section describes the clinical procedures for evaluating study participants and follow-up after administration of the challenge agent.

8.1 Schedule of Attendance

All volunteers will have the same schedule of clinic attendances and procedures as indicated in section 8.13. The total volume of blood donated during the study will be around 838.5ml with up to 120mls extra taken at unscheduled visits as detailed in the SoA. Additional visits or procedures may be performed at the discretion of the investigators, e.g. further medical history and physical examination, blood tests in event of blood AE, urine microscopy in the event of positive urinalysis and imaging studies based on symptoms/ abnormal tests

8.2 Observations

Pulse, blood pressure, oxygen saturations, temperature and respiratory rate will be measured at the time-points indicated in the SoA and may also be measured as part of a physical examination if indicated at other time-points.

8.3 Study Procedures

8.3.1 Urinalysis:

Near-patient testing on urine will be performed at the study site. Urine will be tested for protein, blood and glucose at screening. Urine will additionally be tested for evidence of drugs of misuse (including but not limited to amphetamines, barbiturates, cocaine, opiates, cannabinoids and benzodiazepines) and nicotine use at screening and prior to SARS-CoV-2 challenge.

For WOCBP only, urine will be tested for beta-human chorionic gonadotrophin (β-HCG) at screening and serum β-HCG prior to SARS-CoV-2 challenge.

8.3.2 Mid turbinate (nose) and Oropharyngeal (throat) swabs for virus detection:

Combined mid turbinate nose and oropharyngeal throat swabs will be taken for SARS-CoV-2 viral detection and quantitation using study SOPs as detailed in the SoA. Briefly, qPCR will be performed on each swab and, to fulfil discharge criteria, immunofluorescence for detection of live virus will be performed on pre-discharge samples. In addition, to meet the secondary objectives describing the SARS-CoV-2 viral dynamics in upper respiratory samples, further live culture analysis of swab elution will occur on selected samples collected over the course of the study. Antigen detection (CE marked lateral flow tests) may also be performed on selected samples as part of our exploratory end points. Sequencing for variant typing (requiring an additional swab) may also be carried out at suspected COVID/COVID positive visits.

8.3.3 Nasopharyngeal (nose) for respiratory pathogen detection:

At admission to the quarantine unit a nasopharyngeal swab will be taken to screen for respiratory pathogens including SARS-CoV-2 prior to enrolment. The minimum following pathogens will be screened for:

VIRUSES:

- Adenovirus
- Coronavirus HKU1, NL63, 229E, OC43
- Severe Acute Respiratory Syndrome Coronavirus 2 (SARS-CoV-2)
- Human Metapneumovirus
- Human Rhinovirus/Enterovirus
- Influenza A & B
- Parainfluenza Virus 1-4
- Respiratory Syncytial Virus

BACTERIA:

- Bordetella parapertussis
- Bordetella pertussis
- Chlamydia pneumonia
- Mycoplasma pneumoniae

Additional swabs may be performed at both scheduled follow up visits and at unscheduled assessment of volunteers with suspected or confirmed COVID if deemed necessary by study clinicians based on any symptoms experienced to identify the cause of symptoms and also assess for possible co-infection.

8.3.4 Nasopharyngeal (nose) for RNA-sequencing

Two nasopharyngeal swabs will be taken on the same days that immunology bloods are taken for RNA-sequencing, as detailed in the SoA. Swabs will be taken on all volunteers during the quarantine period but only in individuals who have demonstrated swab positivity during the study at post quarantine follow up visits.

8.3.5 Nasosorption (immune assays):

Up to four strips of synthetic absorptive matrix (SAM™) will be used (1 to 2 per nostril) for 1 minute each to obtain repeated samples of neat nasal epithelial lining fluid. This is a painless minimally invasive procedure that will not require any local anaesthetic. The SAM strip is inserted in the nostril and laid against the inferior turbinate. The participant is then asked to pinch their nostril or a nose clip is positioned.

8.3.6 Safety blood tests:

Blood will be drawn for the following laboratory tests and processed at contractually agreed NHS Trust laboratories using NHS standard procedures. The full list of safety bloods in the study is outlined below. The tests to be done at each safety blood time point (see SoA) will be outlined in the Clinical Study Plan.

- Haematology: Full Blood Count including differential
- Biochemistry: Sodium, Potassium, Urea, Creatinine, Liver Function Tests (Bilirubin, ALT, ALP, Albumin), ferritin, LDH, HbA1C, Troponin-I or T, NT-pro-BNP, C-reactive protein, beta-human chorionic gonadotrophin
- Coagulation screen: Prothrombin time, APTT, D-dimer
- Diagnostic serology; HBsAg, HCV antibodies, HIV antibodies (specific consent will be gained prior to testing blood for these blood-borne viruses)
- Immunology: Interferon Gamma Release Assays (IGRA) to aid as a diagnostic tool for latent or active tuberculosis at screening.
- SARS-CoV-2 antibody (anti-S and/or anti-N protein) (prior to enrolment)

8.3.7 Blood samples for immunology:

- Immunogenicity will be assessed by a variety of immunological assays to meet primary, secondary and exploratory endpoints as outlined in section 8.13.
- ELISA for SARS-COV-2 taken at baseline and throughout the study as an immune endpoint

8.3.8 Pulmonary function tests (PFTs):

Spirometry and Transfer factor of the Lung for Carbon Monoxide (TLCO) will be performed according to standard operating procedures. Height at screening will be used as the baseline measurement for all spirometry assessments.

Spirometry and transfer factor will be repeated at the day 28 visit and only if the participant is no longer considered infectious. If there is a significant drop in FEV1, FVC or transfer factor (>15% from baseline or fall to below 80% predicted), then this will be repeated at subsequent outpatient follow up visits.

Following SA09 PFTs will not be performed in all participants but will be performed in participants who demonstrate PCR positivity beyond D1 and may be performed optionally in volunteers who do not demonstrate evidence of PCR positivity beyond D1 at clinician discretion.

8.3.9 Radiology:

Chest X-rays and CT scans will be carried out in the hospital X-ray department associated with the clinical research unit according to local NHS SOPs. A chest x-ray will be performed during the screening phase. Where possible, this should be acquired with the patient erect, taking a full inspiration and using a PA projection.

A low dose CT of the chest will be acquired at day 5 and additionally may occur at day 11 in individuals with evidence of significant SARS-CoV-2 infection (at clinical discretion of investigator due to sequential positive PCR after the day 5 timepoint, ongoing live viral culture or ongoing symptoms, signs or investigations suspicious for SARS CoV-2 infection). The scanner will have a minimum of 16 detector rows and a standard diagnostic scan will be acquired with patient specific radiation dose optimisation techniques used. The patient should be positioned supine with their arms above their head and the scan performed with the patient at held full inspiration. A spiral scan will be acquired from the lung apices to the costophrenic sulci to obtain full coverage of the lung parenchyma and 1mm imaging slices reconstructed using a lung algorithm. The radiation dose of this examination makes it difficult to justify performing an additional routine CT chest in the screening phase of the study, particularly given that the likelihood of finding abnormalities prior to inoculation is very low. All scans will be reported by a consultant radiologist.

After completion of group 1 the DSMB will review the need for routine CT scans in all participants for groups 2, 3 and 4. The DSMB will consider the safety data as a whole including whether routine CT scans have demonstrated evidence of clinically significant lower respiratory tract involvement and/or altered the management of any participants enrolled in the study. If it is felt that the risks of radiation exposure outweigh the potential benefits of CT scans they will no longer be offered routinely. Instead, a low dose CT chest will be carried out at day 5 and/or day 11* in any participant with COVID-19 infection and any of the following:

- Ongoing severe and persistent cough – Grade 3 reports of coughing via symptom diary cards that is largely persistent over 48 hours (Grade 3: Significant discomfort with marked limitation in activity).
- Any event of confirmed hypoxia (≤ 94%, usually confirmed over a 1-hour period)
- Ongoing signs of lower respiratory tract involvement on auscultation (e.g. crepitations)
- At CI discretion

*Capacity for CT scanning will be pre-arranged for D5 and D11 with the local radiology department. However, if a participant meets the criteria outlined above and service capacity permits, a CT scan may be performed at another timepoint during the quarantine stay.

Importantly CI discretion will be used at all times in the decision to carry out a CT chest. If a participant meets the criteria for CT chest but is felt to be unwell enough to require referral for hospital care, CT chest will not be performed within the study in order to avoid delaying admission and/or treatment. Where a participant undergoes a CT chest at D5 this may or may not be repeated at D11 at CI discretion.

After completion of group 1 the DSMB will advise from the following options:

- Continue use of routine CT scans throughout the study with the option for re-review after enrolment of further participants
- Discontinue use of routine CT scans in all groups (2,3 and 4)
- Discontinue use of routine CT scans in group 2 with continued use of routine CT scans in group 3 and re-review of the use of CT scans prior to progression to group 4

This change will take effect immediately after the DSMB have provided their opinion.

Following DSMB review on 31st October 2022 routine CT scans were discontinued for remaining participants in groups 2, 3 & 4 and instead restricted to participants with symptoms or signs of lower respiratory tract involvement as above

8.3.10 Cardiovascular Magnetic Resonance (CMR):

CMR will be performed at baseline (before being confined) in all volunteers, and at discharge from the clinical research unit, only in individuals who demonstrate PCR positivity after 24 hours during the quarantine stay (brief PCR positivity in first 24 hours is considered to represent residual inoculum rather than true infection). The CMR protocol will include assessment of cardiac structure, function and myocardial tissue characterisation. If intravenous access can be obtained, intravenous injection of a gadolinium-based contrast agent (e.g. Dotarem or Gadovist) per clinically-accepted CMR protocols, will be performed for late gadolinium enhanced imaging (LGE). Participants will be screened for magnetic safety and any contraindications to CMR. Participants who successfully complete a baseline CMR scan will be invited for a follow-up CMR at or soon after discharge from the quarantine unit, for both safety and exploratory purposes. All scans will be reported based on cine, +/- LGE imaging where available, by an appropriately qualified healthcare professional.

The CMR scan will include parametric mapping (e.g. T1/T2/Extracellular volume), which will not be included in the formal clinical report, but rather will form part of our exploratory research endpoints. Images will be used for research purposes by both the study team and Professor Keith Channon group, as part of a collaboration within the framework of the Oxford Acute Myocardial Infarction (OxAMI) Study.

Following SA09, and in agreement with the DSMB, CMR will no longer be carried out at screening or discharge in all participants.

8.3.11 Echocardiography:

For patients who have contraindications to a baseline CMR (e.g. metallic implants, allergy to gadolonium-based contrast), a transthoracic echocardiogram may instead be performed by a trained echocardiographer to screen for structural heart disease before enrolment. An additional Echocardiogram will be obtained when they are discharged from the quarantine unit if CMR cannot be performed (again, only in individuals who demonstrate PCR positivity during the quarantine stay, from 24 hours after inoculation)

8.3.12 Electrocardiogram:

ECGs will be obtained to evaluate the electrical activity of the heart. ECGs will be read on site by an appropriately qualified Investigator.

8.3.13 Patient Health Questionnaire (PHQ-9) and Generalised Anxiety Disorder (GAD-7) Questionnaire

PHQ-9 is a multipurpose instrument for screening, diagnosing, monitoring and measuring the severity of depression. The GAD-7 questionnaire is a screening tool used for diagnosis of generalised anxiety disorder. These will be used at screening to assess participants' eligibility in terms of ability to tolerate isolation in the quarantine unit as per the exclusion criteria. Absolute cut-offs in PHQ-9 and GAD-7 scores will not be used to determine eligibility. However, the scores listed in the exclusion criteria (PHQ-9 ≥4 or GAD-7 ≥5) will be used as a guide. Potential participants may be included with higher scores if there is an alternative explanation for these scores and study clinician and participant believe that their mental health will not be significantly worsened by quarantine. E.g. a participant with ADHD who has symptoms of hyperactivity but feels their mood is good and has previously tolerated isolation well. Where the scores exceed those outlined in the exclusion criteria a detailed explanation of why the participant is suitable for enrolment will be recorded. In addition, an adapted version of these questionnaires will continue to be performed at regular intervals during the study (as per the SoA) as a crude marker of response to the study procedures and quarantine period and as an enabler for discussions between the study team and participant regarding their mental health status.

Both the overall score and individual questions will be reviewed by study staff. Concerning scores or individual questions will prompt timely review by a study clinician. If necessary, the study clinician should discuss the need for further mitigating action for safety purposes (e.g. modification to procedures, safe withdrawal from trial and referral to specialist mental health assessment).

8.3.14 Cognitive tests

To assess changes in cognition associated with infection, a computerised system for repeated assessment of cognitive function will be used. This has been developed by Dr Adam Hampshire (Imperial College London) and has been successfully used in healthy individuals and clinical populations inclusive of traumatic brain injury. Participants will be provided with a study tablet computer assigned to them for from the time of their admission to the quarantine unit until discharge that will contain a pre-loaded app (CogAsses) and factory-provided software only. This app will provide a brief battery of computerised tasks and questionnaires to track speed of information processing, memory, attention, executive function and sleep. The battery of tests will last for ~20-30 minutes. Participants will be asked to complete the cognitive assessment every day around the same time of day during their admission within the quarantine unit. In addition, a standardised questionnaire about the quality of sleep from the preceding night will be included in the app. The cognitive tasks included within this battery of tests are:

1. Motor Control

2. Object memory-Immediate recall

3. Simple Reaction Time

4. Choice reaction time

5. 2D Manipulations

6. Allocentric towers

7. Spatial span

8. Target detection

9. Tower of London

10. Verbal analogies

11. Object memory-Delayed recall

Participants will also be invited to complete this assessment battery at each follow-up visit. All data from the cognitive assessments will be stored in a remote web server hosted within Amazon Web Services. The servers are behind a firewall in a secure cloud computing facility, with access to the data processor requiring a two factor authentication method. These measures represent a high-level of data security that are the standard for any website. Remote storage of performance data will be in a pseudonymised format and will be linked across tasks using unique specific study number. Identifiable information will be stored in an encrypted database 'key' that is separate from the test scores.

8.3.15 University of Pennsylvania Smell Identification Test (UPSIT)

The UPSIT is a well-validated and reliable (test-retest r = 0.94) test that employs microencapsulated "scratch and sniff" odorants. It is provided as booklets containing a series of cards that the participants scratch. They then are asked to identify the scents, which have been validated in an English population. The test provides an index of absolute dysfunction (ie, anosmia, severe microsmia, moderate microsmia, mild microsmia, normosmia, factitious), as well as relative dysfunction based upon age and gender-adjusted normative percentile ranks. The total number of odorant stimuli out of 40 that is correctly identified serves as the test measure. Scores on this test correlate well with other types of olfactory tests, including threshold tests. The UPSIT is designed to be self-administered after explanation of the test by study staff and will be performed at baseline, every three days during quarantine, at the day 28 follow up visit and any suspected Covid-19 visit. We will also perform the smell test at any study visit if clinically indicated e.g. if UPSIT abnormal at previous timepoint or the volunteer reports subjective symptoms of anosmia/parosmia.

8.3.16 Mask wearing

As an additional assessment of virus shedding, volunteers will be asked to wear a single use face mask that has been fitted with a polyvinyl alcohol sampling matrix insert, capable of capturing virus in exhaled

breath (details in section 9.4). They will do this on a daily basis after challenge - once a day for 30 minutes on each occasion, with an additional baseline test prior to challenge (on admission to the quarantine unit). No hazards have been identified by the manufacturer of the PVA material and these face masks have been validated for the detection of *M. tuberculosis* in infected participants [89]. They have recently also been successfully demonstrated to detect SARS-CoV-2 in exhaled breath [90].Up to five PVA strips will be harvested from exposed masks and analysed for virus and virus related signals.

8.3.17 Sample processing and leftover samples

All initial investigations will be outlined in the study-specific laboratory plan and conducted according to local SOPs.

Samples will be retained for a maximum of one year from the end of the study to allow for analysis of protocol-defined laboratory endpoints relating to primary and secondary objectives. Following this period, any leftover samples will be transferred to the OVC biobank if consent has been obtained for this. Any exploratory immunology assays will then be performed under the OVC Biobank ethical approval. If volunteers have not consented to storage and future use of leftover samples, they will be discarded.

Participants will be informed that there may be leftover samples (after all testing for this study is complete), and that such samples may be stored indefinitely for possible future research. Participants will be able to decide if they will permit such future use of any leftover samples. With the volunteers' informed consent, any leftover samples will be frozen indefinitely for future analysis of COVID-specific or vaccine-related responses. If a participant elects not to permit this, all of that participant's leftover samples will be discarded after the required period of storage to meet Good Clinical Practice (GCP) and regulatory requirements.

Samples that are to be stored for future research will be transferred to the OVC Biobank (REC 16/SC/0141).

8.4 Treatments administered

8.4.1 Ronapreve (Regeneron monoclonal antibody cocktail) or Paxlovid (oral antiviral tablets)

Prior to paxlovid availability, treatment with a single intravenous infusion of 1200mg REGN-COV2 will be given to volunteers confirmed to be infected and who demonstrate any warning features of COVID-19 disease beyond mild signs and symptoms that are confined to the upper respiratory tract. (See Section 8.10 for further information).

Once approved as part of SA007, Paxlovid will replace REGN COV2 as rescue therapy. Volunteers confirmed to be infected and who demonstrate any warning features of COVID-19 disease beyond mild signs and symptoms that are confined to the upper respiratory tract will be given two 150mg PF 072321332 (nirmatrelvir) and one 100mg ritonavir tablet (together paxlovid) to take orally twice daily for 5 days. (See Section 8.10 for further information).

8.4.2 Low molecular weight heparin (Dalteparin)

To minimise risk of venous thromboembolism during the extended period of reduced activity in isolation rooms, volunteers will be fitted with TED stockings to wear during quarantine.

In addition, due to the increased risk of VTE in COVID-19 disease, a daily prophylactic subcutaneous injection of dalteparin will be administered if VTE risk is felt to be sufficiently high. As per current OUH policy, LMWH is recommended if patients have reduced mobility AND a significant medical comorbidity (such as an acute infectious disease). Hence we will commence LMWH if:

1. A volunteer develops a "significant medical comorbidity from COVID-19 infection" which for the purposes of this study we define as any of:
   - Evidence of pulmonary infiltrates on CT;
   - ≥ Grade 2 respiratory or systemic symptoms such as shortness of breath or chest pain;
   - Laboratory evidence of pro-thrombotic state e.g. ≥ Grade 2 inflammatory markers or D-dimer;
   - Any other symptoms or signs deemed by the study team to increase risk of VTE

AND

2. The volunteer has a resultant reduction in mobility.

The decision to commence LMWH will be based on clinical discretion by the study clinicians in consultation with the CI. Dosing will be based on recommended prophylactic doses based on volunteer screening body weight as per Table 8:

Table 8. Dalteparin dose per body weight

Weight	Dose
<50kg	2500 units once daily
50-99kg	5000 units once daily
100-150kg	7500 units once daily

8.4.3 Prior and Concomitant Medication

Permitted Medication

- The use of concomitant medications other than those listed in Table 9 is prohibited from D-2 through to D28 unless approved by the CI, including all prescription drugs, herbal preparations, over-the-counter medications, vitamins and minerals.
- Use of all permitted therapies during quarantine has to be documented and agreed at the screening visit.

- The Investigator is to be informed as soon as possible about any medication taken by a subject from the time of screening until the completion of the follow-up visit on Day 28. Agreed concomitant medications taken during the quarantine phase will be stored, prescribed and administered in line with their label-specific requirements and full accountability will be maintained. Use of all concomitant medications will be recorded in the case report form (CRF) throughout the study.

Table 9. Permitted medication and restrictions during quarantine period until Day 28 visit

**Permitted medication**	**Restrictions**
Oral, injected or implanted contraceptives or hormone replacement therapies	Recommended dosing
Simple analgesia and anti-emetics	Recommended dosing
Mild potency topical steroids	Recommended dosing
Over-the-counter creams and topical treatments	Recommended dosing

8.4.4 Prohibited Medication

All medications (including over the counter and herbal supplements), other than those noted above are to be stopped before the planned date of viral challenge unless in the opinion of the Chief Investigator/ Lead Clinician the medication will not interfere with the study procedures or compromise subject safety. Certain medications requiring a specific washout period before a subject is eligible to enter the study; details are provided in Table 10.

As the SARS-CoV-2 vaccination is now being offered to our target population as part of the national roll out, it would be unethical to require volunteers to withhold vaccination in order to participate.

As our study aims to define protective immunity, the study aims remain intact, with or without vaccination. SARS-CoV-2 vaccination will be permitted in the groups with prior infection (groups 1 and 2) as long as it is at least 3 weeks prior and 30 days post enrolment (as per UK HSA guidelines to avoid confusing the differential diagnosis if patients become unwell after vaccination; see section 8.4.5). Vaccination will be noted as a concomitant medication to allow appropriate post hoc analysis on exploratory immunology. We will be able to define non-spike immunogenicity to evaluate immunity induced by infection, as well as measure vaccine and infection induced immunogenicity against the spike antigen. The addition of groups focussing on uninfected vaccinated volunteers will further allow us to assess the full dynamic range of protective immunity induced by vaccination and contrast this with the protective immunity conferred by natural infection.

It is becoming increasingly unlikely with high vaccine uptake in the UK, that significant numbers of unvaccinated volunteers will enrol in the study. However, there is an increasing body of evidence demonstrating the immune benefit conferred by vaccination of convalescent individuals over natural immunity alone [19-22]. We have therefore adjusted our dose escalation policy accordingly to ensure that should unvaccinated individuals wish to enrol they will not be dose escalated without sufficient evidence of safety at prior dosing schedules (As per section 6.1 and 8.9).

Table 10. Prohibited medication

**Prohibited medication**	**Washout required**
Systemic corticosteroid (oral and parenteral) therapy	6 months before the planned date of viral challenge
Systemic (oral and parenteral) antiviral drugs	6 months before the planned date of viral challenge
Vaccinations	30 days either side of Challenge
Short and long-acting anti-histamines	7 days before study-specific screening
Any medication or product (prescription or over-the-counter), for symptoms of nasal congestion or respiratory tract infections including nasal steroids	30 days before the planned date of viral challenge
Any medication with significant interaction with Paxlovid	30 days before the planned date of viral challenge

8.4.5 Vaccination considerations

To date there have been no safety concerns vaccinating individuals with detectable COVID-19 antibody.[91] Current UK COVID-19 vaccine guidelines advise deferring a COVID-19 vaccine to 4 weeks after COVID-19 symptom onset or the first confirmed positive specimen. This is to avoid confusing the differential diagnosis if patients become unwell after vaccination.[91] In this study, all volunteers must defer any COVID-19 vaccine offered to them for a minimum of 30 days from enrolment. If infection develops, volunteers will be advised to follow UK HSA COVID-19 vaccine guidance, currently advising patients to wait for 30 days from symptom onset or from positive PCR nasal-oropharyngeal swab.

An additional consideration for those who receive REGN-COV2 is that the presence of passively acquired circulating SARs-COV-2 antibodies may result in a sub-optimal immune response to the vaccine. The half –life of REGN-COV2 is 25 to 37 days.[92] For this reason, if offered a SARS-CoV-2 vaccine as part of the UK government roll out, volunteers who receive REGN-COV2 will be advised that they should wait a minimum of 40 days from REGN-COV2 therapy before receiving the vaccine (up to 50 days from enrolment).

Additionally, volunteers will be advised that waiting 90 days from REGN-COV2 therapy for first dose of vaccination may further reduce risk of REGN-COV2 interfering with vaccine response. This timeline has been decided after careful consideration, weighing up the current unknown theoretical interaction between vaccination and REGN-COV-2 in people who have pre-existing immunity, and the disadvantages to the volunteer of deferring the first vaccine dose, such as the potential impact on restrictions to travel and work. As this is a changing landscape, study clinicians will discuss with each volunteer based on their personal circumstances and current information, the benefits or disadvantages of deferring vaccination beyond the minimum 40 days.

No additional consideration is required for those who receive Paxlovid.

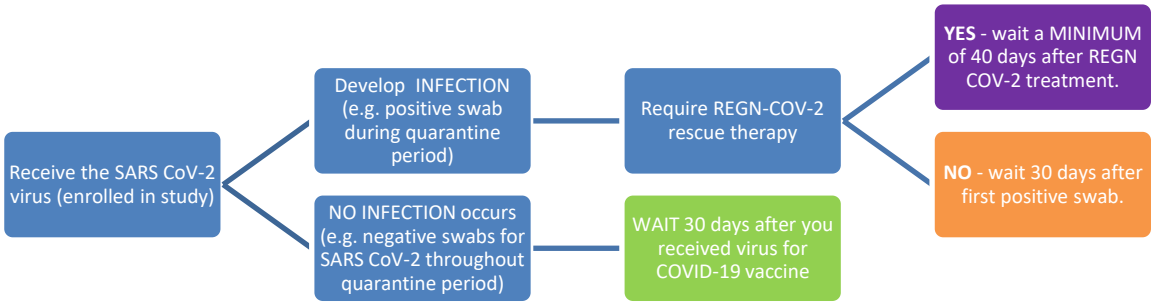

Figure 5: Summary flowchart of COVID-19 vaccination guidance after enrolment

8.4.6 Blood donation

All volunteers must avoid blood donation throughout the course of the study (12 months from enrolment) to reduce risk of developing anaemia. In addition, volunteers will be advised that the NHS blood bank will not accept blood donations from volunteers for a period of 12 months after receiving REGN-COV2.

8.5 Diary card

Following the SARS-CoV-2 challenge, volunteers will be asked to complete an electronic diary. Volunteers will be asked to record any specific solicited and unsolicited AEs twice daily whilst on the quarantine unit. From discharge to day 28 this will continue once daily. From 29 - 84 days post challenge, to capture any "long COVID" symptoms, volunteers will be sent a weekly electronic diary card asking a "Yes/No" question regarding the presence of symptoms. If they have any ongoing symptoms (i.e. select "Yes") further detail on solicited and unsolicited symptoms will be collected. Electronic diary cards will be reviewed with volunteers during quarantine and at the post-challenge follow up visits

8.6 Study visits

The study is divided into outpatient and inpatient/confinement phases. Volunteers will stay overnight for a period of at least 17 days in total, from the morning 2 days before the viral challenge, to at least the 14th day after viral challenge (see Section 8.11 Discharge from confinement). This period of confinement has been chosen to eliminate the possibility of participants in the study transmitting the virus to anyone not involved in the study (i.e. family, household contacts, and the wider community). During the confinement period, all study procedures will take place in the confinement facility. This will be the Experimental Medicine Clinical Research Facility (EMCRF). The exception to this is the Day 5 and 11 CT scan which may take place outside the confinement facility. In this case volunteers will be transported as per standard OUH infection control protocol. The outpatient phase involves screening and follow-up visits which will take place at the CCVTM, Oxford with chest x ray taking place at Oxford University Hospitals NHS trust. Each visit is assigned a time-point and a window period, within which the visit will be conducted.

8.6.1. Pre-screening phase

In order to exclude volunteers who are clearly not eligible, all volunteers expressing an interest in the study will be required to fill out an online pre-screening questionnaire. This questionnaire requests basic information including volunteer age, previous COVID-19 infection and co-morbidities. Consent to solicit this information will be implied by volunteers submitting their answers. If volunteers are not eligible for the study, all information collected from this questionnaire will be destroyed unless consent is given to store for future studies.

To allow sufficient time for prospective participants to fully consider the PIS and what participation in the study entails, the screening and consent process will be split across a minimum 5-day period as follows:

1. Prospective participant completes pre-screening questionnaire and no obvious exclusion criteria met as detailed above.
2. Participant will be sent study participant information sheets and directed to pre-recorded video with a detailed explanation of study information sheets. The structure of the video is guided by the information in the documents.
3. Screening video call arranged a minimum of 72 hours after documents sent.
4. A pre-screening video appointment will be arranged with the study doctor (recorded, audio only). Prospective participants will be asked to confirm that they have read and seen the PIS and video and will be given ample opportunity to ask questions if any aspect of the study remains unclear. Study procedures for eligibility assessment will not formally be undertaken at this call, but if during the discussion the participant volunteer's information that makes them clearly ineligible to take part, they will be thanked for their time and interest but told they are ineligible for the study. Similarly, answers volunteered on the pre-screening questionnaire may be clarified during this call with the purpose of ensuring no clear exclusion criteria are met.
5. If after discussion the participant remains interested in the study then an in-person screening visit and study consent will be arranged at the CCVTM, Oxford a minimum of 48 hours after the pre-screening video appointment. This allows the volunteer time to reflect on the

discussion with the study clinician and also to discuss with their support network should they wish.

The pre-screening video appointment between the participant and study clinician will be recorded with the participant's prior consent. This will be sought in written format on the pre-screening registration questionnaire and verbally at the start of the pre-screening video call. The call will be audio-recorded only with no video details using a secure internet platform. For data management purposes, the participants personally identifiable details will not recorded and it will be saved under a screening number. If volunteers are not eligible for the study or decide they do not wish to consent to the study then this recording will be destroyed. Otherwise this data will be stored on secure University of Oxford servers (in accordance with data protection act 2018) for 7 years in order to act as a reference in the event of any questions about what was and was not discussed.

8.6.2 Consent & Screening Visit

This visit may take place up to 90 days prior to the challenge.

Informed consent will be taken before screening, as described in section 7.2. If consent is obtained, the procedures indicated in the schedule of attendances will be undertaken including a medical history, physical examination, ECG, lung function and blood tests.

The participant's general practitioner will be contacted with the written permission of the participant after screening to ascertain any significant medical history and as notification that the participant has volunteered for the study. During the screening, the volunteers will be asked to provide their national insurance or passport number so that this can be entered on to a national database which helps prevent volunteers from participating in more than one clinical trial simultaneously or over-volunteering for clinical trials (www.tops.org.uk).

Proof of prior SARS-CoV-2 infection (groups 1 & 2) will require:

- Confirmation of a prior positive test for SARS-CoV-2 virus (including date of test) via medical records, occupational health records or verification via the UK HSA (formerly PHE) test & trace system. Where official documentation such as the above is not available, other forms of evidence such as a picture of a positive lateral flow test on a volunteer's phone may be acceptable forms of proof if assessed as being genuine by the study team.

Written consent for access to UK HSA (formerly PHE) test results will be sought at screening.

Groups 3 & 4 will require:

- Written or electronic confirmation of SARS-CoV-2 vaccination via medical records or occupational health records
- Negative anti-nucleocapsid antibody test, unless explainable by vaccine received.
- No concerning history for prior COVID-19 exposure including history strongly suggestive of undiagnosed infection or strong exposure to SARS-CoV-2 e.g. household contact.

QCovid score will be calculated with the patient present and discussed as part of a holistic assessment of risk.

Cardiac assessment (CMR or echocardiogram) will occur, where required, at Oxford University Hospitals NHS trust at a second visit once results from the screening visit have been received and deemed eligible.

Abnormal blood tests following screening will be assessed according to site-specific laboratory adverse event grading tables which are listed in the CSP. Any abnormal test result deemed clinically significant may be repeated to ensure it is not a single occurrence.

If an abnormal finding discovered at screening is deemed to be clinically significant, the volunteer will be informed and referral for appropriate medical care arranged with the permission of the volunteer.

The eligibility of the volunteer will be reviewed at the end of the screening visit and again when all results from the screening visit and cardiac assessment have been considered. Decisions to exclude the volunteer from enrolling in the study or to withdraw a volunteer from the study will be at the discretion of the investigator. If eligible, an inpatient quarantine will be scheduled for the volunteer to receive the challenge and subsequent follow-up. This will be confirmed by a telephone call with a member of the study team who will additionally reiterate pre-quarantine requirements including permitted medications.

To minimise the risk of admitting a volunteer already incubating SARS-CoV-2, we will use the following approach, based on current NHS outpatient pre-operative guidelines and current epidemiological data indicating a median incubation period of 5 days [60, 93]. All volunteers will be reminded of the need to follow current government social distancing guidelines. Any volunteers who have symptoms suspicious of COVID-19 or who are isolating due to being a contact of someone with COVID-19 will be deferred until the study team is happy that the volunteer is not incubating SARS-CoV-2. In addition, 3 days before admission to the quarantine unit (5 days before challenge) all volunteers will be asked to attend clinic for a combined nasal mid turbinate and oropharyngeal swab for SARS-CoV-2 viral detection by PCR. They will then be required to self-isolate at home from this point up until admission to the quarantine unit. On admission they will have a nasopharyngeal swab for SARS-CoV-2 PCR viral detection. Both swabs must be negative for challenge to proceed at Day 0. If either swab is positive the volunteer will be deferred (or excluded depending on the study schedule and planned group enrolment), UK HSA will be informed and the volunteer will be advised to isolate as per the current UK HSA guidelines. While this approach does not completely eliminate the risk of a naturally acquired re-infection, we believe this balances the well-being of our volunteers while maintaining the integrity of the study. A more stringent approach with requirement for a full two weeks home isolation (along with household members) before a 17 day+ quarantine would be more impactful to volunteer's well-being than the theoretical minimal increased risk to the participant of a low dose SARS-Cov-2 inoculation if they were already incubating the virus. Additionally this is not in line with current NHS outpatient guidance. Viral sequencing during the study will enable us to identify any volunteers with a naturally acquired infection, though we believe, with the measures outlined above, the likelihood of this occurring would be low.

Furthermore, if there is clinical suspicion of asymptomatic infection (e.g. high rates of infection locally) an additional 5ml of blood may be taken for serology prior to enrolment, in order to detect changes in antibody titre or anti-nucleocapsid antibody positivity which may indicate asymptomatic infection between screening and enrolment. Evidence of asymptomatic infection prior to entering quarantine may inform a decision to defer (+/- change groups) for participants with possible recent infection. E.g.

participants in group 3 or 4 who are intended to be infection naïve or group 1 and 2 who are intended to have been infected > 3 months prior.

8.6.3 D-2 admission to quarantine unit (commencement of inpatient period) to enrolment D0

On the morning two days before viral challenge, participants will be admitted to the inpatient quarantine facility. Volunteers will be asked to travel to the quarantine unit by private transport (e.g. by a member of their own household), or if not possible, COVID-secure transport will be arranged by us.
On admission, they will be met by staff wearing PPE and will go directly into their own en-suite room. During the inpatient quarantine period care will be delivered in line with Oxford University Hospital NHS inpatient policies and study standard operating procedures.

A 48 hour quarantine period prior to receiving the challenge agent enables review of contraindications to challenge and allows a period of observation to ensure there is no active SARS-CoV-2 (or alternate viral infection) prior to enrolment. Prior to virus inoculation, history and medical examination to confirm ongoing fitness will be performed. Nasopharyngeal swabs will be taken to exclude coincidental respiratory viral infection and SARS-CoV-2 infection (biofire panel) and for transcriptomics, nasosorption sampling performed and blood will be taken (for repeat safety blood tests and immunology). An ECG will be taken to exclude baseline abnormalities. Vital signs (Pulse, blood pressure, oxygen saturations, respiratory rate and temperature) and weight will also be recorded. Urine screening for drugs of misuse and nicotine use will be performed on all volunteers. WOCBP will have a serum pregnancy test. If a participant is excluded on the basis of any of these tests, a previously-screened backup volunteer will be contacted to enter the study instead.

Baseline PHQ-9, GAD-7, cognitive tests, UPSIT and mask wearing will be performed as per SoA.

8.6.4 Day 0: Enrolment

Volunteers will be considered enrolled in to the study at the point of SARS-CoV-2 intranasal challenge.
On day of challenge, the eligibility of the volunteer will be reviewed. If necessary, a medical history and physical examination may be undertaken to determine need to postpone the challenge depending on criteria listed in section 7.3.4. SARS-CoV-2 challenge will be administered as described below.

8.6.5 Post Challenge inpatient period (D0 to D14+)

SARS-CoV-2 challenge will be administered intranasally on D0, according to the study-specific SOP, in an isolation room (negative pressure or positive pressure ventilated lobby). Volunteers will be infected with SARS-CoV-2 at the pre-defined dose using the minimum possible volume of challenge virus (up to a maximum 1mL) by intranasal drops divided between nostrils. After virus inoculation, participants will be closely observed for at least 60 minutes for adverse events as well as physiological observations. During the administration of SARS-CoV-2 virus, monitoring equipment, oxygen, medicines including bronchodilators and resuscitation equipment will be immediately available for the management of anaphylaxis and bronchospasm according to the study-specific SOP.

In order to minimise dissemination of the SARS-CoV-2 virus into the environment and to ensure the protection of staff, measures will be instituted during and following challenge to fully comply with local infection control and occupational health regulations. These will be detailed in the study specific SOP.

Following the challenge, volunteers will remain in their isolation room where they will be constantly monitored until they meet discharge criterion (see Section 8.11 Discharge from confinement). The volunteer will be required to fill a daily e-diary with specific solicited local and systemic AEs. There will be additional sections to collect information on unsolicited AEs and use of medication for symptoms. The electronic diary will be kept twice daily whilst in the quarantine unit.

Information will be collected on the following solicited AEs:

Table 11. Solicited Adverse Events

**Local (respiratory) solicited AEs**	**Systemic solicited AEs**
Cough (any cough)	Documented Fever (oral temp ≥37.8^{o}C)
Coughing up phlegm	Feverishness/chilliness
Coughing up blood	Joint pains (arthralgia)
Wheeze	Muscle pains (myalgia)
Shortness of breath	Fatigue
Sore throat	Headache
Tickly throat	Malaise/ tiredness
Chest tightness	Nausea
Chest pain	Vomiting
Stuffy nose (Nasal congestion)	Diarrhoea
Runny nose (Nasal discharge)	Abdominal Pain
Hoarse voice	Confusion
Sneezing	Anosmia/ hyposmia (loss or reduction in sense of smell)
	Parosmia (distortion in smell)
	Aguesia/ dysguesia (loss or altered sense of taste)
	Blisters
	Rashes
	Sore eyes
	Earache

Objective safety parameters and research procedures will be collected as per the schedule of attendance (section 8.13) including vital signs (four times a day post challenge), nasal mid turbinate, oropharyngeal and nasopharyngeal swabs, nasosorption, Pulmonary CT (day 5 +/- 11), regular ECG, mental health & cognitive assessments, smell testing, mask wearing & blood tests. Participants will wear TED stockings and

may receive a daily subcutaneous low molecular weight heparin injection (See section 8.4.2 for details on commencement criteria and dosing). Additional permitted medications/ over the counter therapy for symptom relief will be given as required (see section 8.4.4 Prohibited Medication).

8.7 Sequence of Enrolment and challenge of volunteers

Volunteers will be sequentially allocated into each group according to availability.

8.8 Blinding

In order to minimise volunteer adverse event reporting bias by volunteers, study participants will be kept blinded to swab results for as long as possible. Volunteers may be able to infer swab positivity if investigations such as CT scans are ordered, depending on their dose group. Where possible, blinding will be maintained.

Volunteers who require REGN COV-2 or paxlovid administration, LMWH or changes to treatment due to infection may become aware of their swab results earlier (i.e. at the point of treatment they will know their infection status).

In addition, where research investigations require interpretation such as the D5 CT scan, the reporting clinician will be blinded to infection status where possible (unless clinically indicated in the interest of patient safety), as will the senior immunologist processing research samples. This is to reduce bias that could be introduced in result interpretation and/or at the sample processing.

8.9 Dose escalation, de-escalation and confirmation

The study will involve dose escalation of SARS-CoV-2 to demonstrate safety and identify an optimum dose in participants with previous SARS-CoV-2 infection (+/-vaccination) (see Figure 6 below) and uninfected SARS-CoV-2 vaccinated individuals (see Figure 7).

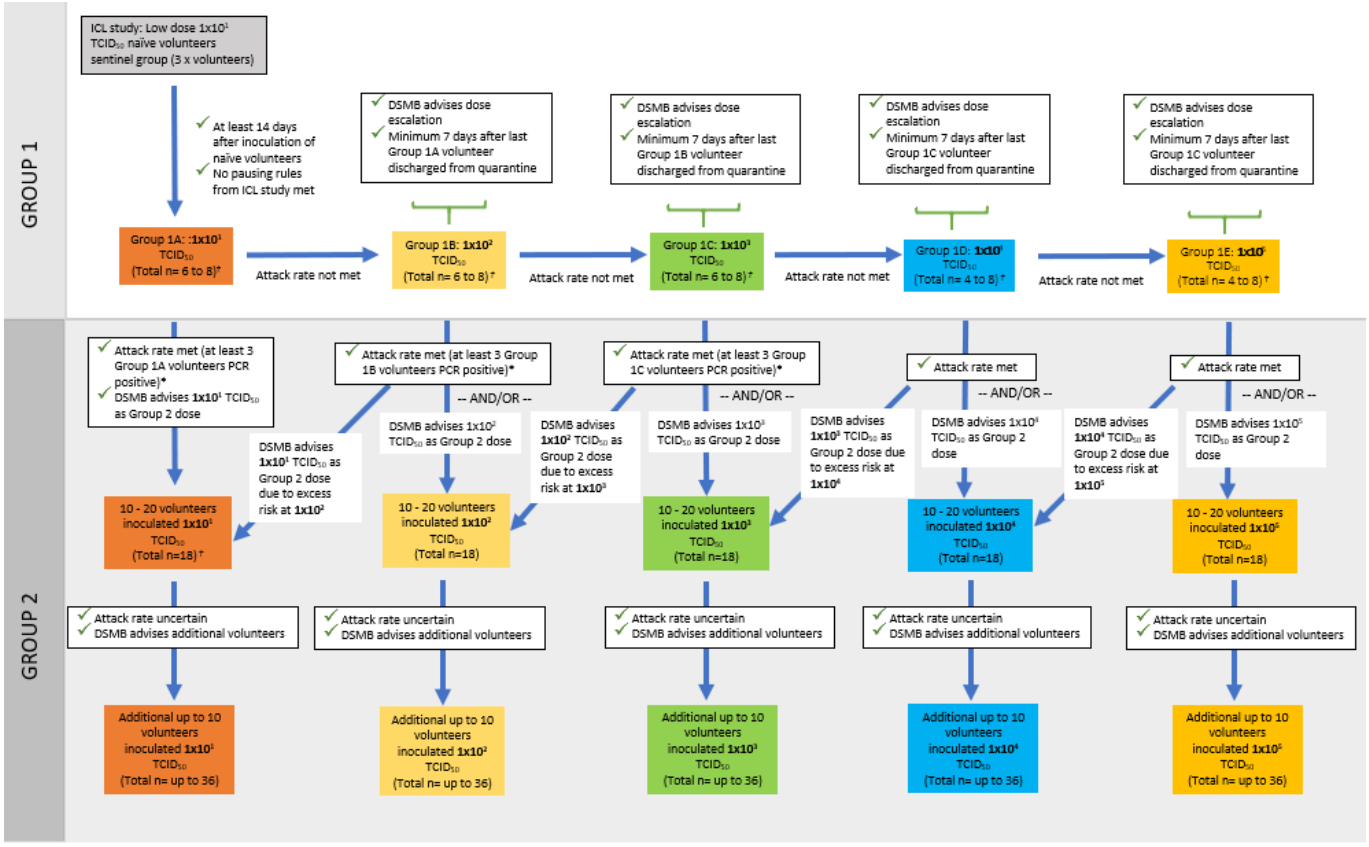

*Starting 24 hours post-inoculation and up to discharge from quarantine.
† We will not dose escalate in unvaccinated volunteers until at least 4 unvaccinated individuals have received the lower dose, this may increase the group size of groups 1A to 1C to up to 12. If an unvaccinated volunteer is successfully infected we will create a separate subgroup (n=6 to 8) for unvaccinated volunteers

Figure 6. Groups 1 and 2: Dose escalation and dose confirmation

Association with ICL study:

- Commencement of the trial will only occur after the first dose (group 1A) has been given to three volunteers in the naïve SARS-Cov-2 challenge study run by Imperial College London (ICL).

Inoculation of our first volunteer in group 1A will only occur after a minimum of 14 days from the inoculation of their third volunteer at the equivalent dose AND a satisfactory review by their DSMB that there have been no safety concerns warranting activation of a holding rule.

- If, as expected, the tolerability of each dose is comparable in seropositive and seronegative individuals, we will commence subsequent doses at the same time as ICL. Given the difference in study populations (seronegative versus seropositive individuals), ICL may reach their attack threshold at a lower dose. In this instance, if deemed safe by the DSMB we will continue to inoculate at the higher group doses as described below.

Group 1: Groups of up to 8 individuals will be challenged at each dose and dose escalation will proceed as follows.

- The starting dose for Group 1A (10^1 $TCID_{50}$) was selected as the lowest reliably quantifiable amount of virus. 6-8 participants in the group will be challenged at the group 1A dose (10^1 $TCID_{50}$).
- The attack rate threshold for up to 8 participants for any given Group 1 sub-group is 50% +/- 10%. i.e we would aim for 5 participants but a minimal threshold as low as 3 participants would be acceptable dependent on other factors such as clinical symptoms as outlined in the note below. Once this attack rate has been met we will move on to Group 2 (details below).
- If the number of infected participants in the first Group 1 sub-group does not meet this pre-defined attack rate threshold (50% +/- 10%), the next sub-group will be inoculated with a higher dose. ie if 2 or fewer volunteers in the subgroup develop infection after 14 days and no pausing rules are met (based on severity and frequency of adverse events, see Group Safety Holding Rules), the dose would be escalated to the next sub group (following DSMB review of data). If during the course of enrolment we become aware that we will not meet our target attack rate threshold i.e. 6 volunteers have been challenged with no evidence of infection then we will proceed to the next subgroup without inoculating all 8 volunteers.
- The next sub-group will be challenged at the earliest, 7 days after the last participant was discharged (i.e. minimum of 3 weeks between viral inoculations).
- If the dose of 10^4 TCID50 (Group 1D) is reached and the number of infected participants in the group does not meet the pre-defined attack rate threshold* no pausing rules are met, and the DSMB approves we will inoculate 4-8 participants at 10^5 TCID50 (group 1E).

Group 2. Dose confirmation.

- Once the number of infected participants in a group meets the pre-defined attack rate threshold* or if a minimum of 4 individuals have been safely inoculated in the final dose escalation group (Group 10^5 TCID50), a second group of 10-20 participants ("Group 2") will be challenged with the same dose level (i.e. resulting in 14-28 participants challenged at that dose level) to increase confidence for the attack rate at that dose level.
- Dose escalation will be capped at 10^5 TCID50 regardless of attack rate in Group 1E. Therefore, following inoculation of at least 4 individuals in this group (regardless of attack rate). The study may proceed to group 2 following safety review from the DSMB.
- In practice, the quarantine unit limits each round to 5 volunteers. Therefore, no more than 5 volunteers will be enrolled at any one time.
- An additional 10 participants in Group 2 may be challenged if review of clinical and virological outcome data suggest that a larger sample size is needed to establish confidence in the attack rate (see Statistics and Data Analysis). If following dose escalation, review of clinical and virological outcome data suggest a risk of participants being exposed to excess risk (due to extremely high

attack rates or high viral loads), then group 2 will be challenged with a previously tested lower dose (determined by discussion with DSMB) and confidence in the attack rate of that lower dose will be increased.

- If a dose meeting our target attack rate is not found in Group 1, then Group 2 will continue at the last highest safe dose identified after discussion with the DSMB, to enable a larger sample size to assess the dynamic range of protection at that dose and ensure confidence in the negative infection rate at that dose.

The planned sample size for Group 2 is 10-20 subjects. However, once 10 -20 subjects have been enrolled, we will carry out a statistical review of the immunology findings to determine if it is possible to draw conclusions about appropriate dose in the context of protective immunity from this study. If further data is needed to achieve this endpoint we will enrol up to a further 10 subjects in Group 2 depending on the results of that statistical review.

Unvaccinated individuals

- As the UK vaccination schedule progresses, it is unlikely we will be able to enrol unvaccinated volunteers into higher dosing groups. However, there is an increasing body of evidence demonstrating the immune benefit conferred by vaccination of convalescent individuals over natural immunity alone [19-22].
- If an unvaccinated volunteer is recruited to enrol in an intermediate or higher dosing group (i.e. 1B, 1C, 1D, or 1E) then they will only be inoculated at that dose if at least 4 unvaccinated individuals have already received the immediate lower dose and demonstrated no evidence of infection or safety concerns.
- This may mean enrolling unvaccinated volunteers at a lower dose group after dose escalation has occurred. If an unvaccinated volunteer did develop infection at a lower dose then we would create an unvaccinated cohort at that dose and dose escalation in unvaccinated individuals would only be able to occur after confirmation that the target attack rate of 50% (+/-10%) in up to 8 participants is not met and following safety review with the DSMB. If an unvaccinated subgroup is created for the sake of clarity we will amend the protocol accordingly to reflect this.

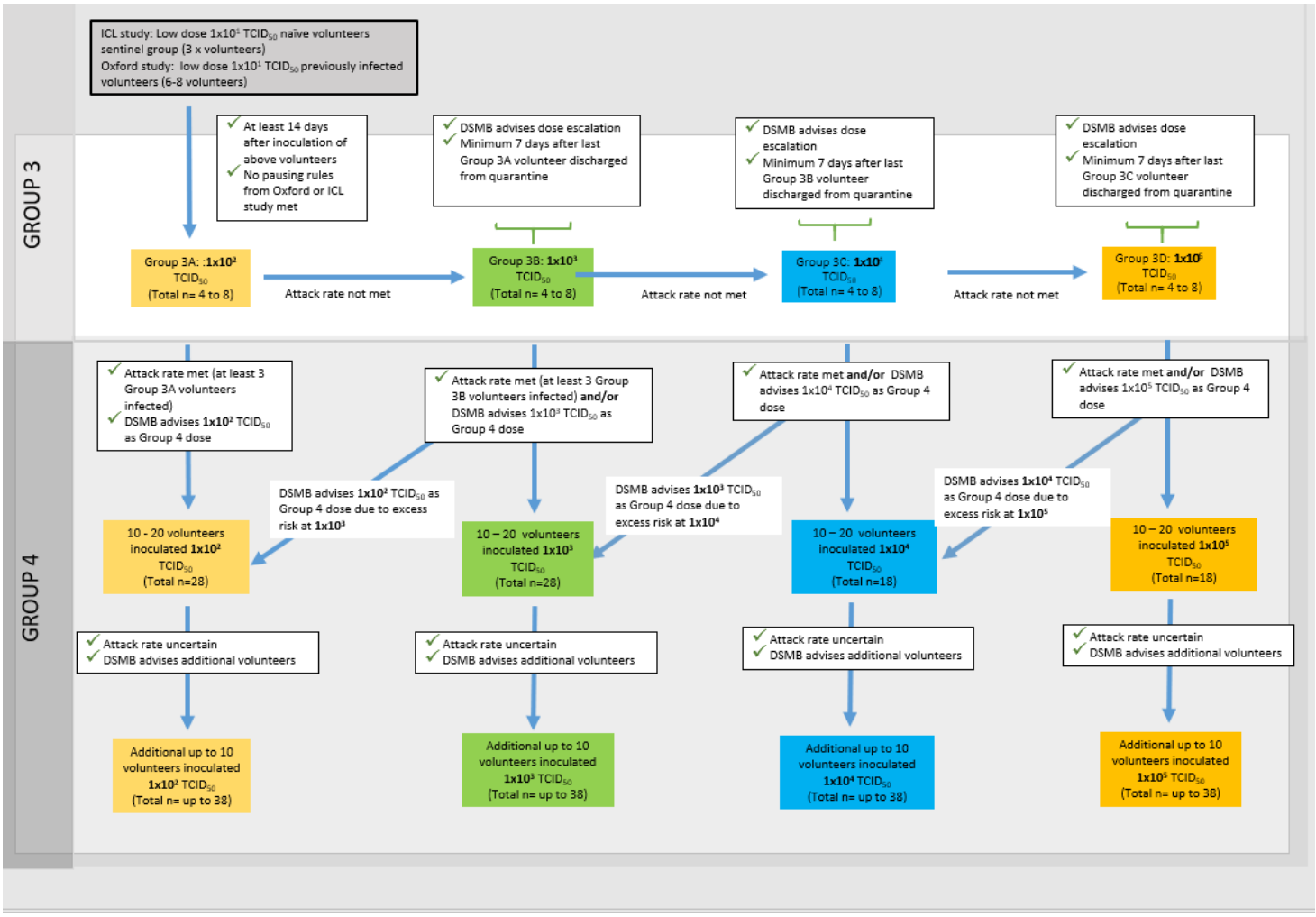

Figure 7. Groups 3 and 4: Dose escalation and dose confirmation

Group 3: Groups of up to 8 individuals will be challenged at each dose and dose escalation will proceed as follows.

- The higher starting dose for Group 3A (10^2 $TCID_{50}$) was selected based on our data from group 1A demonstrating that a target attack rate of 50% (+/-10%) was not achievable in unvaccinated individuals with a history of prior infection with SARS-CoV-2.
- The attack rate threshold for up to 8 participants for any given Group 3 sub-group is 50% +/- 10%. i.e we would aim for 5 participants but a minimal threshold as low as 3 participants would be acceptable dependent on other factors such as clinical symptoms as outlined in the note below. Once this attack rate has been met we will move on to Group 4 (details below).
- If the number of infected participants in the first Group 3 sub-group does not meet this pre-defined attack rate threshold (50% +/- 10%), the next sub-group will be inoculated with a higher dose. ie if 2 or fewer volunteers in the subgroup develop infection after 14 days and no pausing rules are met (based on severity and frequency of adverse events, see Group Safety Holding Rules), the dose would be escalated to the next sub group (following DSMB review of data).
- If, after enrolling a minimum of 4 volunteers we have not infected any volunteers we would dose escalate to the next subgroup (following DSMB review of data) The lower minimum number of 4

volunteers in groups 3A-D, as compared with the minimum of 6 volunteers in groups 1A-1C reflects the safety data accumulated from group 1.

- The next sub-group will be challenged at the earliest, 7 days after the last participant was discharged (i.e. minimum of 3 weeks between viral inoculations).
- If the dose of 10^4 $TCID_{50}$ (Group 3C) is reached and the number of infected participants in the group does not meet the pre-defined attack rate threshold,* no pausing rules are met and the DSMB approves we will inoculate 4-8 participants at 10^5 $TCID_{50}$ (group 3D).

Group 4. Dose confirmation.

- Once the number of infected participants in a group meets the pre-defined attack rate threshold* or if a minimum of 4 individuals have been safely inoculated in the final dose escalation group (Group 10^5 TCID50), a second group of 10-20 participants ("Group 4") will be challenged with the same dose level (i.e. resulting in 14-28 participants challenged at that dose level) to increase confidence for the attack rate at that dose level. An additional 10 participants may be challenged if review of clinical and virological outcome data suggest that a larger sample size is needed to establish confidence in the attack rate (see Statistics and Data Analysis).
- If following dose escalation, review of clinical and virological outcome data suggest a risk of participants being exposed to excess risk (due to extremely high attack rates or high viral loads), then group 4 will be challenged with a previously tested lower dose (determined by discussion with DSMB) and statistical confidence in the attack rate of that lower dose will be increased.
- If a dose meeting our target attack rate is not found in group 3, then group 4 will continue at the last highest safe dose identified after discussion with the DSMB, to enable a larger sample size to assess the dynamic range of protection at that dose and increase statistical confidence in the negative infection rate at that dose.
- Dose escalation will be capped at 10^5 TCID50 regardless of attack rate in Group 3D. Therefore, following inoculation of at least 4 individuals in this group (regardless of attack rate). The study may proceed to group 4 following safety review from the DSMB.

The planned sample size for Group 4 is 10-20 subjects. However, once 10 -20 subjects have been enrolled, we will carry out a statistical review of the immunology findings to determine if it is possible to draw conclusions about appropriate dose in the context of protective immunity from this study. If further data is needed to achieve this endpoint we will enrol up to a further 10 subjects in Group 4 depending on the results of that statistical review.

Given the evolving situation with Omicron it may become increasingly difficult to recruit to group 4 due to limited numbers of eligible participants who have not been previously infected. If it becomes clear that it is not feasible to recruit 10-20 subjects into group 4 we may combine groups 2 and 4 for analysis purposes aiming for up to 30 participants across both groups.

*Note: Dose escalations, de-escalations and confirmation group decisions will be made following discussion by the Investigators with the DSMB and TSC (as well as, prior to Group 2 and 4 enrolment, use of REGN-COV2 or paxlovid, and routine CT scans during quarantine - see relevant sections for more information). Decisions will be based not only on meeting the attack rate threshold but also take into account the quantity of viral load, symptomatology and other clinical factors to identify the dose that is

not only safe but also most appropriate for future use of the model for antibody protection studies and vaccine and therapeutics testing. Therefore the attack rate thresholds defined above will not be absolute criteria for group progression but will be considered together with these other data. For example, if at a given dose the attack rate does not reach the pre-determined threshold but is associated with high viral loads in those volunteers who are SARS-CoV-2 positive (such as a peak viral load of $>10^7$ $TCID_{50}$), it may be decided that a further group at the same dose is challenged rather than escalating to a higher dose level. Conversely, if an attack rate threshold is reached but the peak viral load by qPCR is low in all group participants (e.g. $<10^6$ $TCID_{50}$ and therefore unlikely to be culturable), dose escalation may still take place if deemed to be safe.

We have built in a range for our target attack rate (+/-10%) to establish a robust human challenge model that also accommodates our co-primary endpoint of safety and clinical symptoms. For example, an attack rate of 40% would be deemed acceptable to meet our objectives if volunteers are frequently reporting symptoms. Conversely, if dosing resulted in asymptomatic infection in the majority of volunteers we would aim for a final attack rate closer to 60%.

8.10 Rescue treatment with REGN COV2 (Ronapreve) or Paxlovid

Early "rescue" therapy, when backed by compelling evidence of efficacy and when available, is indicated for challenge model infections that demonstrate warning features beyond mild signs and symptoms that are confined to the upper respiratory tract. To limit symptomatic disease and the risk of lower respiratory tract involvement, and until such time a preferred rescue therapy is available and approved for use in the study, a monoclonal antibody REGN-COV2 will be administered to all participants challenged *in the dose escalation groups* with SARS-CoV-2 as follows.

A single 60 minute intravenous infusion of 1200mg of REGN COV2 will commence in any volunteer confirmed to be infected with SARS-CoV-2 and with any of the following:

- Persistent tachypnoea – Respiratory Rate ≥ 21 for ≥ 8 hours
- Persistent Fever – Fever (≥ 37.9) from a time point 5 days post symptom onset and present for at least once each day for ≥ 72 hours
- Severe and persistent cough – Grade 3 reports of coughing via symptom diary cards that is largely persistent over 48 hours (Grade 3: Significant discomfort with marked limitation in activity).
- For CT changes related to SARS CoV-2 infection, anything more than mild changes (based on the British Society of Thoracic Imaging COVID reporting scale) represents a standalone trigger and mild changes in combination with other concerning clinical features could also be considered.
- Any event of confirmed hypoxia (≤ 94%, usually confirmed over a 1-hour period)

Once approved as part of SA007, volunteers *in the dose escalation groups* meeting the above criteria for rescue therapy will be given Paxlovid in place of REGN-COV2.

Importantly CI discretion will be used at all times in the decision to start rescue therapy. Biochemical markers (e.g. elevated CRP/D-dimer) will not be used in isolation, though a combination of factors outside of the triggers above could still lead to a decision to start therapy. Once a decision to commence rescue therapy has been made by the CI/PI, the NHS Infectious Diseases team at the Oxford University

Hospital, will be made aware. The subject will remain within the quarantine unit to receive rescue therapy unless protocol stated criteria for transfer to NHS care are met.

A study doctor will be available for the administration period to monitor for any immediate reactions as per section 6.4. If any signs or symptoms of a possible hypersensitivity reaction are noted the treatment will be stopped and participant managed symptomatically. Once symptoms have resolved and at the clinician's discretion treatment may be restarted. Any signs of a serious reaction will result in complete termination of the treatment.

Emergency equipment and medication for the treatment of acute infusion/ hypersensitivity reactions (e.g., antihistamines, bronchodilators, IV fluids, corticosteroids, paracetamol and adrenaline) will be available for immediate use. In addition, immediate assistance will be available from the NHS on-site emergency clinical team in the small likelihood that emergency treatment is required.

After enrolment into Group 1 is completed, DSMB will review clinical data from group 1 participants. Following their advice, Group 2 will proceed without the use of rescue therapy unless the DSMB advises otherwise. Likewise, after enrolment into Group 3 is completed, the DSMB will review use of rescue therapy for Group 4.

8.11 Discharge from confinement

Participants will be quarantined for a minimum of 14 days after enrolment (17 days in total as admitted at day-2) and until discharge criteria for clearance of infection:

- 2 consecutive nose/throat swabs must show no viable virus on viral culture
- If the culture assay fails repeatedly, a downward trend in qPCR with a Ct value >35 in 2 consecutive swabs will be sufficient evidence of minimal infectiousness

Quarantine will be extended until these criteria are met.

If, on the 14th day of residence post challenge, all clinically significant symptoms have resolved and criteria for infection clearance has been met, participants will be discharged from the confinement facility at the discretion of the principle investigator..

If a participant wishes to withdraw after virus inoculation but before discharge criteria are met, they will be counselled regarding infection control measures and reminded of the government guidance to self-isolate within their own home. As part of the counselling, they will be advised to avoid contact with any household members to reduce risk of infection; that they may not receive rescue therapy in the community; and that they must remain in close contact with the study team to be monitored for any safety signals.

The Health Protection Unit local to the participant's home will be informed and any additional infection control measures recommended by them will be implemented.

8.12 Subsequent follow up visits: Day 28, 56, 84, 168 and 365

On discharge, the volunteer will be provided with a contact card providing 24/7 phone access to the research Doctor on the trial if required. Following discharge, participants will continue to complete a daily electronic Diary until Day +28. From day 29-84, they will then be required to complete a weekly electronic diary to assess for "long COVID symptoms." Diaries will be monitored remotely by the study team. The volunteer will be contacted for further assessment if they document anything deemed to be a clinical concern (i.e. any grade 3 symptoms, worsening symptoms or any symptoms of clinical concern such as, shortness of breath or chest pain). Follow-up visits will take place as documented in the visit schedule (section 8.14). Volunteers will be assessed for local and systemic adverse events, interim history, lung function, SARS-CoV-2 PCR test and blood tests at these time points as detailed in the schedule of attendances. Blood will also be taken for exploratory immunology. The subject will be asked to inform the study team as soon as possible if an episode of natural COVID-19 infection (see section 8.13), COVID-19 vaccination, medical consultation or hospitalisation occurs, after which the study team will obtain further details of the episode.

If participants experience adverse events (laboratory or clinical), which the investigator (physician), CI and/or DSMB determine necessary for further close observation, the volunteer may be transferred to an appropriate inpatient ward at Oxford University Hospitals NHS Foundation Trust for observation and further medical management under the care of the consultant on call.

Hospitalisation of any subject deemed possibly, probably or definitely related to the challenge agent or study procedures in any way will lead to immediate suspension of further enrolments into the trial. The DSMB will be convened to assess the clinical evidence in order to determine whether the study may proceed (see Data Safety Monitoring Board) as per section 10.10.

8.13 Unscheduled additional visits for individuals with possible or confirmed COVID-19 in post quarantine follow up period

Following discharge, participants will be asked to contact the study team should they develop any new* symptoms consistent with possible COVID-19 infection. They will be asked to undergo testing for SARS-CoV-2 via the usual routes available to them e.g. occupational health testing. Volunteers will also be provided with lateral flow tests by the study team to ensure they have access to SARS-CoV-2 testing. Any swab results positive for SARS-CoV-2 will prompt a follow up "Positive COVID-19 test visit" for clinical review, nasal-oropharyngeal swab(s) for e.g. qPCR +/- live viral assays +/- sequencing SARS CoV-2 PCR and live viral testing, deep nasopharyngeal swabs for RNA as well as possible assessment for co-infection and blood testing (safety bloods and exploratory immunology). In addition, volunteers with symptoms deemed by the investigator to be highly suspicious for COVID-19 (e.g. loss of sense of smell) may prompt a "suspected COVID-19 visit" which will be the same as outlined above. Suspected COVID-19 and positive COVID-19 test visits will occur within 5 days of symptom onset/ COVID positive result.

Staff will wear full PPE for this visit and the room cleaned down as per local SARS-CoV-2 infection control SOPs. Individuals will be advised to self-isolate at home whilst awaiting results. If evidence of SARS-CoV-2 virus is detected, (indicating a community acquired infection), individuals will be advised to follow current government self-isolation requirements for positive SARS-CoV-2 tests, self-isolate at home and UK HSA will be notified(or as per the current guidelines at the time).

*New symptoms that warrant SARS-CoV-2 testing will include the following:

- Fever >37.8°C (excluding post vaccination fevers)
- New onset persistent cough
- New onset change or loss in sense of smell and/or taste

Volunteers may also be asked to undertake a COVID test at clinician discretion (e.g. developing symptoms compatible with COVID not included in the above list).

Volunteers who have attended for a 'positive COVID-19' visit or a 'suspected COVID-19' visit where they were found to have been infected with SARS-CoV-2 in the community will attend for a further ' 4 weeks post COVID-19 infection' follow up visit at 4 weeks (-1 week/+2weeks) from their suspected/positive COVID-19 visit. We will perform repeat clinical review, nasal-oropharyngeal swab(s) for e.g. SARS CoV-2 qPCR +/- live viral assays +/- sequencing , nasosorption sampling, Deep nasopharyngeal swabs for RNA as well as possible assessment for co-infection and blood testing (exploratory immunology +/-safety bloods if clinically necessary). These visits will be organised where possible to coincide with pre-planned follow up visits. If this is the case samples will not be duplicated.

8.14 Schedule of Attendances

Visit number	Screening			1 (Quarantine)	Home	2	3	4	5	6	(+/-)	(+/-)
**Timeline (days)**	D-90 to D-5	D-14 to D-3	D-5	D-2 to D14+ See Quarantine schedule for daily breakdown	Discharge to D27	D28	D56	D84	D168	D365	Additional unscheduled suspected COVID/ COVID positive visit	Additional unscheduled 4 weeks post COVID infection visit
Time windows (days)			±1			±5	±7	±14	±21	±28	5	-1/+2 weeks
Inclusion/exclusion criteria	X			X								
Review contra-indications	X			X								
Informed consent	X			X								
Medical history	X			X		(X)	(X)	(X)	(X)	(X)	X	X
Physical examination	X			X		(X)	(X)	(X)	(X)	(X)	(X)	(X)
Vital signs	X			X		X	X	X	X	X	X	X
PHQ-9	X			X		X	(X)	(X)	(X)	(X)		
GAD-7	X			X		X	(X)	(X)	(X)	(X)		
Urinalysis	X			X		(X)	(X)	(X)	(X)	(X)		
β-HCG test (females only)	X (urine)			X								
Urine drugs of misuse & nicotine screen	X			X								

Visit number	Screening			1 (Quarantine)	Home	2	3	4	5	6	(+/-)	(+/-)
**Timeline (days)**	D-90 to D-5	D-14 to D-3	D-5	D-2 to D14+ See Quarantine schedule for daily breakdown	Discharge to D27	D28	D56	D84	D168	D365	Additional unscheduled suspected COVID/ COVID positive visit	Additional unscheduled 4 weeks post COVID infection visit
Time windows (days)			±1			±5	±7	±14	±21	±28	5	-1/+2 weeks
Chest X-ray	X											
Lung CT scan				X[f,q]								
PFTs[a]	X					X[a]	(X)	(X)	(X)	(X)		
ECG	X			X		(X)	(X)	(X)	(X)	(X)		
Biofire Respiratory pathogen Swab (including SARS-CoV-2)				X		(X)	(X)	(X)	(X)	(X)	(X)	(X)
Smell Test (UPSIT)	X			X		X	(X)	(X)	(X)	(X)	X	(X)
Cognitive Tests				X		X	X	X	X	X		
**Sars-Cov-2 intranasal challenge**				X								
**Rescue treatment (REGN-COV2 or Paxlovid)[p,q]**				(X)								
Telephone calls		X			(X)							
Local & systemic events/ reactions				X	X	X	X	X	X	X	X	X
VTE prophylaxis				X								
Daily diary card set up				X								

Visit number	Screening			1 (Quarantine)	Home	2	3	4	5	6	(+/-)	(+/-)
**Timeline (days)**	D-90 to D-5	D-14 to D-3	D-5	D-2 to D14+ See Quarantine schedule for daily breakdown	Discharge to D27	D28	D56	D84	D168	D365	Additional unscheduled suspected COVID/ COVID positive visit	Additional unscheduled 4 weeks post COVID infection visit
Time windows (days)			±1			±5	±7	±14	±21	±28	5	-1/+2 weeks
Daily diary card final review						X						
Weekly diary card set up						X						
Weekly diary card final review								X				
Combined mid-turbinate and oropharyngeal swab(s) for detection of SARS CoV-2 virus [b]			X	X		X	X	X	X	X	X	X
Nasopharyngeal swab for RNA (x2)[l]				X		(X)	(X)	(X)	(X)	(X)	(X)	(X)
Nasosorption – immunology				X		X	X	X	X	X	X	X
Mask wearing samples				X								
Biochemistry	5			30		5		5			(5)	(5)
Haematology	4			14		2		4			(2)	(2)
Coagulation	3			18		3		3			(3)	(3)

Visit number	Screening			1 (Quarantine)	Home	2	3	4	5	6	(+/-)	(+/-)
**Timeline (days)**	D-90 to D-5	D-14 to D-3	D-5	D-2 to D14+ See Quarantine schedule for daily breakdown	Discharge to D27	D28	D56	D84	D168	D365	Additional unscheduled suspected COVID/ COVID positive visit	Additional unscheduled 4 weeks post COVID infection visit
Time windows (days)			±1			±5	±7	±14	±21	±28	5	-1/+2 weeks
Serology (HIV, HBV, HCV & SARS-CoV-2 Antibodies)	5[o]											
HLA typing				4								
IGRA	6											
Exploratory immunology[i]				415[k]		62.5	62.5	62.5	62.5	62.5	(62.5)[h]	(62.5)
Blood vol per visit (mL)	23		0	481	0	72.5	62.5	74.5	62.5	62.5	(72.5)	(72.5)
Cumulative blood vol (mL)	23		23	504 [e,i,k]	504	576.5	639	713.5	776	838.5[h]		

Quarantine period (Visit 1)																		
**Timeline (days)**	**D-2**	**D-1**	**Challenge D0**	**D1**	**D2**	**D3**	**D4**	**D5**	**D6**	**D7**	**D8**	**D9**	**D10**	**D11**	**D12**	**D13**	**D14**	**D15+ to Discharge**	**Discharge**
Inclusion/exclusion criteria			X																
Review contra-indications	X	X	X	X	X	X	X	X	X	X	X	X	X	X	X	X	X	X	X
Informed consent			X																
Medical history	X		(X)	(X)	(X)	(X)	(X)	(X)	(X)	(X)	(X)	(X)	(X)	(X)	(X)	(X)	(X)	(X)	(X)
Physical examination	X		(X)	(X)	(X)	(X)	(X)	(X)	(X)	(X)	(X)	(X)	(X)	(X)	(X)	(X)	(X)	(X)	(X)
Vital signs[m]	X	X	X	X	X	X	X	X	X	X	X	X	X	X	X	X	X	X	X
PHQ-9	X		X			X			X			X			X		X	(X)	X
GAD-7	X		X			X			X			X			X		X	(X)	X
Urinalysis	X						X					X					X		
β-HCG test (females only)	X (serum)		X (urine)																
Urine drugs of misuse & nicotine screen	X																		
Lung CT scan[q]								(X)[f]						(X)[f]					
ECG	X			(X)	(X)	(X)	X	(X)	(X)	(X)	X	(X)	(X)	(X)	X	(X)	(X)	(X)	
Biofire Respiratory pathogen Swab (including SARS-CoV-2)	X																		
Smell Test (UPSIT)		X		(X)	X	(X)	(X)	X	(X)	(X)	X	(X)	(X)	X	(X)	(X)	X	(X)	
Cognitive Tests		X		X	X	X	X	X	X	X	X	X	X	X	X	X	X	X	
Weight	X	(X)	(X)	(X)	(X)	(X)	(X)	X	(X)	(X)	(X)	(X)	(X)	(X)	X	(X)	(X)	(X)	(X)
**Sars-Cov-2 intranasal challenge**			X																
**REGN-COV2 – one dose maximum[p]**					(X)	(X)	(X)	(X)	(X)	(X)	(X)	(X)	(X)	(X)	(X)	(X)	(X)	(X)	
**Paxlovid (one course maximum)[p]**					(X)	(X)	(X)	(X)	(X)	(X)	(X)	(X)	(X)	(X)	(X)	(X)	(X)	(X)	
Daily diary card			X	X	X	X	X	X	X	X	X	X	X	X	X	X	X	X	X
Local & systemic events/ reactions			X	X	X	X	X	X	X	X	X	X	X	X	X	X	X	X	
TED stockings +/- LMWH			X	X	X	X	X	X	X	X	X	X	X	X	X	X	X	X	

Combined mid-turbinate and oropharyngeal swab for qPCR and live viral assays	X[j]			X	X	X	X	X	X	X	X	X	X	X	X	X	X	X[j]	
Nasopharyngeal swab for RNA (x2)	X				X			X		X				X			X		
Nasosorption[d]		X	X	X	X	X		X		X				X			X	(X)	
Mask wearing samples		X		X	X	X	X	X	X	X	X	X	X	X	X	X	X	X	
Biochemistry	5[c]				5			5		5				5			5	X	
Haematology	4				2			2		2				2			2	X	
Coagulation	3				3			3		3				3			3	X	
HLA typing	4																		
Exploratory immunology[i]	102.5[k]	(X)[k]			62.5			62.5		62.5				62.5			62.5	(65)[e]	
Blood vol per visit (mL)[g]	118.5	0	0	0	72.5	0	0	72.5	0	72.5	0	0	0	72.5	0	0	72.5		
Cumulative blood vol (mL)	118.5	118.5	118.5	118.5	191	191	191	263.5	263.5	336	336	336	336	408.5	408.5	408.5	481	481[e]	481[e]

[a] if abnormality detected at D28 further PFTs may be performed with SARS-CoV-2 PCR swab 48 hours +/- 24 hours beforehand. D28 PFT performed if PCR+ beyond D1 or at clinician discretion following SA09.

[b] See further detail in Section 8.3.2 Mid turbinate swabs for virus detection

[c] Includes serum β-HCG

[d] Twice daily for first 3 days post challenge and Day-1, otherwise once daily

[e] Up to a total of 65mls additional immunology bloods may be taken if participant stays beyond 14 days during the rest of the quarantine period

[f] For group 1 participants or any participant enrolled before DSMB review on October 31st 2022 a CT scan will be performed on day 5, but for day 11 CT will only occur if evidence of infection (PCR positive swabs or symptoms, signs or investigations consistent with COVID-19). For participants enrolled into groups 2,3&4 after October 31st CT scans will be performed at day 5 and/or 11 if they meet criteria outlined in section 8.3.9. If a participant in groups 2,3&4 meets the criteria outlined in 8.3.9 and service capacity permits, a CT scan may be performed at another timepoint during the quarantine stay.

[g] Blood volumes for biochemistry, haematology, serology and coagulopathy may vary dependent on the requirements of minimum blood volume for local laboratories

[h] Any additional bleeds will not total more than an additional 125mls for immunology for the entire study duration

[i] Includes screening for evidence of anti-REGN-COV2 antibodies if required

[j] One swab only if at D-2 or on day of discharge

[k] If vein fails this blood volume may be taken over a course of two days (Day-2 and Day-1), and a minimum of 82.5ml may be taken if total 102.5ml is not possible, at clinical discretion.

(X) –procedure may occur on clinical discretion of investigator

[l] Nasopharyngeal swabs for RNA sequencing will only be performed at post quarantine follow up visits in volunteers who have demonstrated evidence of SARS-CoV-2 positivity at any point during the study

[m] Whilst every effort will be made to ensure vital signs are taken as close to a specified time point as possible minor deviations will be acceptable to allow for prioritisation of work load e.g. vitals taken 12 minutes post challenge where 10 minutes is the specified time point

[o] An additional 5mls of blood may be taken for serology for SARS-CoV-2 antibodies if clinically indicated between screening visit and enrolment

[p] Once Paxlovid oral antiviral tablets are approved as part of SA007, REGN-COV2 will no longer be used as rescue therapy

9. STUDY AGENTS AND DEVICES

9.1 SARS-CoV-2 virus

9.1.1 Identity of wild type SARS-CoV-2 challenge virus

The SARS-COV-2 challenge virus strain was originally obtained from a nose/throat swab taken from a patient who developed respiratory symptoms consistent with COVID-19. The isolate was plaque purified to obtain a 'single' virus entity (Professor Wendy Barclay lab, Imperial College). The selected plaque, B1, was subsequently manufactured in accordance with GMP at the Great Ormond Street manufacturing suite. As per communication with MHRA the virus is a non-CTIMP, therefore neither the master stock nor dilutions are subject to conditions set out in Article 13(1) Directive 2001/20/EC. The undiluted challenge virus (Master Virus Bank) has undergone extensive quality testing performed during manufacture (identity, appearance, sterility, infectivity and contaminants) according to pre-determined specifications. Selected individual inoculum vials that have been prepared at the various dilutions required for the study may also be tested. The specific manufacturing process and release testing of the SARS-CoV-2 challenge virus have been reviewed by the MHRA and confirmed suitable. This includes a thorough review by the MHRA of the validation documentation of each of the safety and release testing assays, all conducted in accordance with GMP that were used in the challenge virus testing. The challenge virus will be stored in a secure –80°C freezer (normal temperature range –60°C to –90°C). All challenge virus vials will be supplied by hVIVO.

9.1.2 Storage

The SARS-CoV-2 vials will be shipped to the CCVTM, University of Oxford, Churchill Hospital, in the presence of a temperature logger. The vials will be transported and packaged as per local SOPs approved following appropriate risk assessments by the University Health and Safety Office. The vials will be re-labelled for local use as per study SOPs. Stocks of challenge virus will be stored in a secure –80°C freezer (normal temperature range –60°C to –90°C) in a Schedule 5 compliant facility at the CCVTM. All movements of vials of the study agent in or out of the locked freezer will be documented. SARS-CoV-2 accountability, storage, shipment and handling will be in accordance with local SOPs and other relevant local forms. Vials will be thawed just before inoculum preparation. Once thawed, vials of stock virus will not be re-used for human challenge studies. The residual inoculum in the vials used for inoculating the volunteers with the challenge virus will be labelled with the date, corresponding volunteer number(s), time of thawing and time of inoculation. The vials will be sent to the lab for culture and/or frozen and stored in accordance with the lab analytical plan. All storage records will be maintained in full compliance with GCP.

9.1.3 Dispensing and administration

SARS-CoV-2 virus will be used for experimental infection of volunteers with full details of preparation and inoculation procedures outlined in the local SOP. On the day of inoculation (day 0), vials of challenge virus prepared with the appropriate dose/dilution (see above) will be removed from storage in the -80°C freezer and transferred to the quarantine unit on dry ice as per study transport SOP. Each volunteer will be inoculated in the high containment room in which they will subsequently remain quarantined by a

clinically-trained staff member. For safety purposes, staff present for inoculation will be limited to the minimum required for the procedure and essential training. Volunteers will then be inoculated as per local SOP. Briefly, a sign will be placed on the door of the room to indicate that inoculation is taking place and to prevent accidental entry of others. Staff will wear Level 2 PPE (including FFP3 mask or respirator; full waterproof gown; goggles or visor) when in the participant's room for the duration of the inoculum procedure. The inoculum dose will be rapidly defrosted by warming in the gloved hand. Lying down, volunteers will be inoculated with a maximum 200ul inoculum, depending on concentration of the vial, by 4 drops into the nostrils (maximum 100ul per naris) using filter tips and pipette. This will be done slowly with sufficient interval between each inoculation drop to ensure maximum contact time with the nasal and pharyngeal mucosa. Volunteers will be asked not to swallow during the procedure to ensure maximal contact.

To preserve stocks of SARS-CoV-2 inoculum it may be necessary to use a single vial to dose 2 or more volunteers. In this case the vial will be securely closed and labelled before being transferred to the next room. Once in the next room the vial will be opened and inoculation will proceed in the same way. The vial will then be re-closed and returned to a secure -80°C freezer. A more detailed explanation can be found in the SOP. Following inoculation, advice regarding infection control and hand hygiene will be given. After the inoculation procedure any further contact between the volunteer and staff members will require that staff wear PPE in line with local SOPs for care of suspected/ confirmed COVID-19 patients.

After inoculation, the vials of challenge virus (containing any residual remaining virus) will be labelled with the date and time of inoculation and the volunteer number. The vials will be stored in a secure –80°C freezer to allow for future batched quantification of virus. If more than one volunteer is inoculated from the same vial the volunteers' details will be recorded on the same vial.

9.2 REGN COV2 and Paxlovid

Once approved as part of SA007, Paxlovid will be used in place of REGN-COV2 as rescue therapy.

<u>REGN COV2</u>

REGN COV2 (combined REGN10933/casirivimab and REGN10987/imdevimab) is recently licensed in the UK (20th August 2021). Casirivimab and imdevimab will be supplied in 20R sterile glass vials of 11.1 mL withdrawable volume, at a concentration of 120mg/mL. Casirivimab and imdevimab will be released by Regeneron by a quality controller or nominated person, and sent to Oxford University Hospitals NHS Foundation Trust Clinical Trials pharmacy. All shipments will come with a temperature monitoring device (vials must remain at 2-8 degrees). The vials will then be receipted into stock and recorded onto the master accountability log as per local SOP. On receipt, vials will be stored in pharmacy in a secured temperature monitored fridge 2 – 8°C and protected from light. The vials will be labelled with the details of the clinical trial as per study SOPs. Once required as per study protocol, a prescription will be completed by a research clinician authorised to prescribe, as per study specific SOP. The research pharmacist will check, prepare and release the Ronapreve (combined casirivimab and imdevimab .All movements of vials in or out of the locked fridge will be documented.

The Ronapreve will be prepared by the pharmacist as per local risk assessment and study SOP. In brief, a dose of REGN COV2 is prepared by combining 600mg casirivimab and 600mg imdevimab (1.5 vials each) with 250mL of 0.9% sodium chloride in an intravenous (IV) infusion bag. Drug and volume checks will be

performed in accordance with OUH local practice. Once prepared the IV bag will be labelled with volunteer number, time of preparation, study name and the wording "For clinical study use only"; in addition to the labelling requirements of OUH NHS Trust for prepared IV infusions (e.g. volunteer name, DOB etc).

The volunteer will be monitored as per local OUH practice post infusion for a biological infusion (and see table 6 for REGN-COV2 specific considerations).

Following administration, used vials and infusion bag can be disposed of in clinical waste as per local OUH guidelines. Any unused vials will be returned to Regeneron on completion of the study.

Paxlovid

Paxlovid is licensed for use in the UK (31st Dec 2021), although will be used off-license for this study. It is produced by Pfizer in the form of two different film-coated tablets, each containing a different constituent of the active medications; Nirmatrelvir and Ritonavir. Each pink film-coated tablet contains 150mg of Nirmatrelvir and each white tablet contains 100mg of Ritonavir. The recommended course for treatment is 300mg Nirmatrelvir (two tablets) and 100mg of Ritonavir (one tablet), taken twice daily for 5 days.

All medication is taken orally. All three tablets should be taken at the same time, tablets should be swallowed whole and not chewed, broken or crushed. Paxlovid can be taken with or without food.

9.3 Dalteparin

Dalteparin is a licensed low molecular weight heparin used routinely to reduce the risk of the development of venous thromboembolism. If required, it will be obtained through OUH pharmacy, stored, prescribed, dispensed and administered in accordance with Oxford University Hospital NHS Trust policy for Venous Thromboembolism prevention and the SmPC.

9.4 Duckbill face mask

Each duckbill face mask (Integrity® 600-300) contains a collection matrix which consists of strips of Polyvinyl Alcohol (PVA) produced by 3D printing. This system allows the capture of aerosolized droplets from the lungs. These masks, which are categorised as "sample containers" under the MHRA in vitro diagnostics regulation, will be manufactured and supplied in bulk to the study team by Professor Michael Barer at the University of Leicester. Masks will be appropriately labelled to allow batch identification. Collection matrix samples at each time point will be sent to our collaborators at the University of Leicester for viral assays, for infected volunteers and uninfected volunteers where necessary (e.g. for negative controls).

All investigations using the PVA matrix will be conducted according to site specific SOPs. Evaluation and use of this non-CE marked technology in the study will be registered with the MHRA portal by the University of Leicester as manufacturers of the device. MHRA approval is not required.

10. ASSESSMENT OF SAFETY

Safety will be assessed by the frequency, incidence and nature of AEs and SAEs arising during the study. Recording and reporting of all AEs will take place as detailed in SOP VC027 Adverse Event Data Collection and Analysis.

All adverse event data (both solicited and unsolicited) reported by the volunteer will be graded (Table 13) and entered onto a volunteer's electronic diary card daily for one month from enrolment and then weekly to 84 days following the administration of SARS-CoV-2. The electronic diary will be provided by REDCap™. Outside the diary periods, respiratory and systemic AEs (Table 11. Solicited Adverse Events) will be specifically solicited at each visit, and graded by severity (as detailed in "Table 14. Severity grading criteria for local and systemic AEs.")

All AEs starting after the diary period(s), or persisting after this period, will be recorded in the AE line listing of the eCRF. In addition, all Grade 3 vital signs, Grade 3 safety blood tests, and any other investigation results considered clinically significant will be recorded on the AE line listing throughout the study (as per SOP VC027).
All AEs will be followed to the end of the participant's involvement in the study (if not resolved prior). If there is a causal relationship to SARS-COV-2, and it is not resolved by this point, the AE will be followed until resolution or stabilisation. In addition, all AEs that result in a volunteer's withdrawal from the study will be followed up until a satisfactory resolution occurs, or until a non-study related causality is assigned (if the volunteer consents to this). SAEs will be collected throughout the entire study period.

Data from the diary cards will be extracted following the last volunteer last visit (LVLV) or at any time prior to this in order to perform an interim safety analysis.

Photographs of AEs may be taken for documentation purposes and with volunteers' written informed consent. Examples would be an inoculation site reaction or rash. Any photographs would be stored pseudonymised using a unique specific study number and identifying details on the face would not be included.

10.1 Definitions

10.1.1 Adverse Event (AE)

An AE is any untoward medical occurrence in a volunteer, which may occur during or after administration of the SARS-CoV-2 virus and does not necessarily have a causal relationship with the intervention. An AE can therefore be any unfavourable and unintended sign (including any clinically significant abnormal laboratory finding or change from baseline), symptom or disease, whether or not considered related to the study intervention.

10.1.2 Adverse Reaction (AR)

An AR is any untoward or unintended response to the challenge agent (SARS-Cov2) or any of the research procedures. This means that a causal relationship between a study procedure(s) and an AE is at least a reasonable possibility, i.e., the relationship cannot be ruled out. All cases judged by the investigator as having a reasonable suspected causal relationship to the agent or other study procedure (i.e. possibly, probably or definitely related to it) will qualify as AR.

10.1.3 Serious Adverse Event (SAE)

An SAE is an AE that results in any of the following outcomes, whether or not considered related to the study intervention:

- Death

- Life-threatening event (i.e. the volunteer was, in the view of the investigator, at immediate risk of death from the event that occurred). This does not include an AE that, if it occurred in a more severe form, might have caused death.
- Persistent or significant disability or incapacity (i.e. substantial disruption of one's ability to carry out normal life functions).
- Hospitalisation or prolongation of existing hospitalisation regardless of length of stay, even if it is a precautionary measure for continued observation; other than as part of planned study procedures outlined in the protocol following initial viral inoculation. Hospitalisation (including inpatient or outpatient hospitalisation for an elective procedure) for a pre-existing condition that has not worsened unexpectedly does not constitute a serious AE.
- An important medical event (that may not cause death, be life threatening, or require hospitalisation) that may, based upon appropriate medical judgment, jeopardise the volunteer and/or require medical or surgical intervention to prevent one of the outcomes listed above. Examples of such medical events include allergic reaction requiring intensive treatment in an emergency room or clinic, blood dyscrasias, or convulsions that do not result in inpatient hospitalisation.
- Congenital anomaly or birth defect.

10.1.4 Serious Adverse Reaction (SAR)

An AE (expected or unexpected) that is both serious and, in the opinion of the investigator, believed to be possibly, probably or definitely due to the challenge agent or any other study procedure, based on the information provided.

10.2 Foreseeable adverse reactions

In this study, foreseeable adverse reactions (expectedness) for the challenge agent, SARS-CoV-2, are defined as the solicited adverse events described in "Table 11. Solicited Adverse Events."

The expected reactions to REGN COV-2 and Paxlovid rescue therapies are defined as those outlined in the SmPC (www.gov.uk).

All other foreseeable adverse reactions to study procedures are outlined in "Table 7. Risks associated with other Study procedures".

10.3 Unforeseen Serious Adverse Reactions

A serious adverse reaction to the challenge agent or study procedures, the nature and severity of which is not consistent with known information on the challenge agent and/or study procedures.

NB: To avoid confusion or misunderstanding the following note of clarification is provided: "Severe" is often used to describe intensity of a specific event, which may be of relatively minor medical significance. "Seriousness" is the regulatory definition supplied above.

With regard to use of the challenge agent, there are many known complications of COVID-19 disease that could meet the definition for "serious." However, this study has been designed with the intention of

asymptomatic or mild disease only. Therefore, there are no expected serious adverse reactions to SARS-Cov-2 inoculation and any such event will be defined as an "unforeseen serious adverse reaction".

All unforeseen SARs will be reported to the REC as outlined in Section 10.6, and assessed to determine if any actions are needed, for example urgent safety measures or requests for substantial amendments.

10.4 Causality

For every AE, an assessment of the relationship of the event to the administration of the challenge agent SARS-CoV-2, REGN-COV2, Paxlovid, or other study procedure, will be undertaken by the CI or delegated clinician. An interpretation of the causal relationship of the intervention to the AE in question will be made, based on the type of event; the relationship of the event to the time of SARS-CoV-2 challenge or other study procedure; the known biology of SARS-Cov2; the known side effects from study interventions (Table 12). Alternative causes of the AE, such as the natural history of pre-existing medical conditions, concomitant therapy, or other risk factors will be considered and investigated. Causality assessment will take place during planned safety reviews, interim analyses (e.g. if a holding or stopping rule is activated) and at the final safety analysis, except for SAEs which will have causality assigned at time of reporting by a medically qualified study member as outlined below. If necessary, advice may be sought from Regeneron for causality assessment of SAEs to REGN-COV2.

Table 12. Guidelines for assessing the relationship of study intervention to an AE.

0	**No Relationship**	No temporal relationship to study intervention ***and***<br>Alternate aetiology (clinical state, environmental or other interventions); ***and***<br>Does not follow known pattern of response to study intervention
1	**Unlikely**	Unlikely temporal relationship to study intervention ***and***<br>Alternate aetiology likely (clinical state, environmental or other interventions) ***and*** does not follow known typical or plausible pattern of response to study intervention.
2	**Possible**	Reasonable temporal relationship to study intervention; ***or***<br>Event not readily produced by clinical state, environmental or other interventions; ***or***<br>Similar pattern of response to that seen with SARS-COV-2 (or study intervention under question)
3	**Probable**	Reasonable temporal relationship to study intervention; ***and***<br>Event not readily produced by clinical state, environment, or other interventions ***or***<br>Known pattern of response seen with SARS-COV-2 (or study intervention under question)

4	**Definite**	Reasonable temporal relationship to study intervention; ***and***<br>Event not readily produced by clinical state, environment, or other interventions; ***and***<br>Known pattern of response seen with SARS-COV-2 (or study intervention under question)

10.5 Assessment of severity

The severity of clinical adverse events will be assessed according to the scales in Table 13 and Table 14. Grading of laboratory adverse events can be found in the site SOP.

Table 13. Severity grading criteria for physical observations.

	**Grade 1 (mild)**	**Grade 2 (moderate)**	**Grade 3 (severe)**	**Grade 4 (Potentially life threatening)**
Fever (oral)	37.8°C - 38.4°C	38.5°C – 38.9°C	>39.0°C - 40°C	>40°C
Pulse oximetry	95-96%	93-94%	≤92%	Intubation/ Imminent respiratory arrest
Respiratory Rate	17-20	21-25	>25	Intubation
Tachycardia (bpm)*	101 - 115	116 – 130	>130	A&E visit or hospitalisation for arrhythmia
Bradycardia (bpm)**	50 – 54	40 – 49	<40	A&E visit or hospitalisation for arrhythmia
Systolic hypertension (mmHg)	141 - 150	151 – 155	≥155	A&E visit or hospitalisation for malignant hypertension
Diastolic hypertension (mmHg)	91 – 95	96 – 100	≥100	A&E visit or hospitalisation for malignant hypertension
Systolic hypotension (mmHg)***	85 – 89	80 – 84	<80	A&E visit or hospitalisation for hypotensive shock

*Taken after ≥10 minutes at rest **When resting heart rate is between 60 – 100 beats per minute. Use clinical judgement when characterising bradycardia among some healthy participant populations, for example, conditioned athletes. ***Only if symptomatic (e.g. dizzy/ light-headed)

Table 14. Severity grading criteria for local and systemic AEs.

**GRADE 0**	None
**GRADE 1**	Mild: Transient or mild discomfort ; no medical intervention/therapy required, does not interfere with regular activities
**GRADE 2**	Moderate level of discomfort: Mild to moderate limitation in activity – some assistance may be needed; no or minimal medical intervention/therapy required
**GRADE 3**	Severe: Significant discomfort with marked limitation in activity, some assistance usually required; medical intervention/therapy required, hospitalisation possible

Any changes from baseline results for other study procedures (non-laboratory e.g. ECG, urinalysis, smell testing) during the study will be assessed for their clinical significance by the CI or an appropriately qualified delegate. Clinically significant changes will be reported as AEs, whether or not deemed attributable to the challenge agent. The CI or delegate will assess non-clinically significant changes to determine whether they should be recorded.

10.6 Reporting Procedures for Serious AEs

In order to comply with current regulations on serious adverse event reporting the event will be documented accurately and notification deadlines respected. SAEs will be reported to the CI (as the Sponsors representative) immediately (within 24 hours) of the investigators being aware of their occurrence. The safety monitoring committee (DSMB) chair will be notified of SAEs deemed possibly, probably or definitely related to study interventions immediately (within 24 hours) of the investigators' being aware of their occurrence.

A serious adverse event (SAE) occurring to a participant should be reported, using the non-CTIMP Serious Adverse Events Reporting Form, to the REC, that gave a favourable opinion of the study, where in the opinion of the Chief Investigator the event was 'related' (resulted from administration of any of the research procedures) and 'unexpected' in relation to those procedures. Reports of related and unexpected SAEs should be submitted within 15 working days of the Chief Investigator becoming aware of the event.

10.7 Reporting of events related to REGN-COV2

Any SAEs deemed to be related to REGN-COV2 will be reported within 24 hours of investigator awareness to Regeneron and to the MHRA via Dr Kirsty Wyndebach by email (covid.clinicaltrials@mhra.gov.uk) as per agreement with the MHRA (Private correspondence with CI Helen McShane).

Furthermore; we will report to Regeneron the following adverse events of special interest (AESI) related to REGN-COV-2.:

Grade ≥2 infusion-related reaction or Grade ≥2 hypersensitivity reaction. Where:

i. Infusion-related reactions are defined as any relevant adverse events that occurs during the infusion or up to day 4.
ii. Hypersensitivity reactions are defined as any relevant adverse event that occurs during the infusion or up to study day 29.

Additionally, although not an AE, identification of pregnancy in a female participant within 6 months of receipt of REGN-COV2 will be reported within 24 hours to Regeneron via a standardised reporting form. Optional consent for reporting of pregnancy in female partners of male participants within 6 months of receipt of REGN-COV2 will also be sought. Outcomes for all participant pregnancies (and consented partner pregnancies) will be reported to Regeneron.

The only data that will be transferred to Regeneron will be restricted solely to safety information as outlined above.

10.8 Procedures to be followed in the event of abnormal findings

Eligibility for enrolment in the study in terms of laboratory findings will be assessed as detailed in SOP OVC027. Abnormal clinical findings from medical history, examination or investigations will be assessed as to their clinical significance throughout the study. Laboratory AEs will be assessed using the site-specific tables in SOP OVC027. If a test is deemed clinically significant, it may be repeated, to ensure it is not a single occurrence. If a test remains clinically significant, the volunteer will be informed by a study doctor and appropriate medical care arranged as appropriate, such as referral to the volunteer's GP or NHS specialist, and with the permission of the volunteer. Decisions to exclude the volunteer from enrolling in the study or to withdraw a volunteer from the study will be at the discretion of the chief investigator or relevant investigators.

10.9 Interim Safety Reviews

The safety profile of intranasal SARS-CoV-2 will be assessed on an on-going basis by the investigators with communication to the DSMB as necessary. The CI and relevant investigators (as per the study delegation log) will also review safety issues and SAEs as they arise.

For the dose escalations in groups 1 and 3, six to eight volunteers will receive a new dose and the study investigators will wait a minimum of 14 days and until live virus negative before a safety review by the CI. If less than three volunteers have developed infection then we will refer to the DSMB for consideration of dose escalation. The next group of six to eight volunteers at the next higher dose of SARS-CoV2, will be challenged only following favourable review by the DSMB, and a minimum of 7 days after the last volunteer has been discharged.

If an unvaccinated volunteer is to be recruited into an intermediate or higher dose group the DSMB will also have to review data from at least 4 unvaccinated volunteers at the preceding dose.

The DSMB will review safety data accumulated after each group and evaluate frequency of events, safety and infection rate data prior to starting a new dose group. The DSMB will make recommendations concerning the conduct, continuation or modification of the study.

10.10 Safety Group Holding Rules

Safety holding rules have been developed to take into account the fact that this is a first-in-human dose escalation study using SARS-CoV-2 virus. Solicited AEs are those listed as foreseeable AEs in Table 11. Solicited Adverse Events" of the protocol. Unsolicited adverse events are adverse events other than these foreseeable AEs.

10.10.1 Group holding rules

The following would act as a trigger to place the study on hold and the DSMB would need to be consulted prior to recommencement:

- Solicited adverse events: If more than one volunteer develops the same grade 3 solicited AE beginning within 2 days after challenge (day of challenge and one subsequent day) and persisting at Grade 3 for >48 hours.
- Unsolicited adverse events: If more than one volunteer develops a grade 3 unsolicited AE (that is considered possibly, probably or definitely related to challenge or study procedures) and persists at grade 3 for >48 hours.
- Laboratory adverse event: If more than one volunteer develops the same grade 3 laboratory AE considered possibly, probably or definitely related to challenge agent or study procedures that persists at grade 3 for >72 hours
- Any serious adverse event considered possibly, probably or definitely related to the challenge agent or study procedures

In addition to these pre-defined criteria, the study can be put on hold upon advice of the Chief Investigator, Study Sponsor, regulatory authority, Ethical Committee(s) or DSMB, for any single event or combination of multiple events which, in their professional opinion, jeopardise the safety of the volunteers or the reliability of the data.

If a holding rule has been met and, following an internal safety review by the DSMB it is deemed appropriate to restart dosing, a request to restart dosing with pertinent data will be submitted to the REC. The internal safety review will consider:

- The relationship of the AE or SAE to the challenge agent or study procedures.
- The relationship of the AE or SAE to the challenge dose
- If appropriate an amendment will be submitted, for example with additional screening or laboratory testing for other volunteers to identify those who may develop similar symptoms and alterations to the current Participant Information Sheet (PIS) if required.

If ICL (parallel SARS-CoV-2 naïve controlled human infection model), activate a safety group holding rule this will be reviewed by the DSMB and they will advise us accordingly.

The local ethics committee and sponsor will be notified if a holding rule is activated or released.

All enrolled volunteers will be followed for safety until resolution or stabilisation (if determined to be chronic sequelae) of their AEs, providing they consent to this.

10.10.2 Individual stopping rules (will apply to all challenged individuals)

Separate individual stopping rules will not apply in this study, as each volunteer will only receive one challenge dose of SARS-Cov2.

11 STATISTICS

This is primarily an exploratory study to identify a safe and infectious dose of wild type SARS-CoV-2 in healthy volunteers, suitable for future intervention studies. No formal sample size calculation has been performed for this early stage dose finding study. However, a sample size of up to a total of 132 participants felt sufficient to meet the primary objective of escalating/expanding the dose in a safe manner whilst providing information on the attack rate. Our previous experience other clinical studies suggests that this sample size is a feasible number to recruit, screen, enrol, and follow up in practical terms, whilst also allowing the evaluation of the human response to the challenge agent.
The planned sample size for Group 2 and 4 is 10-20 subjects. However, once 10 and then 20 subjects have been enrolled, we will carry out a statistical review of the immunology findings to determine if it is possible to revise this sample size in order to draw conclusions about appropriate dose in the context of protective immunity from this study. We will therefore enrol up to a further 10 subjects in Group 2 and 4 depending on the results of that statistical review.
It is expected that the amount of missing, unused or spurious data will be insignificant. Unused and spurious data will be listed separately and excluded from the statistical analysis. Missing data will be excluded from the statistical analysis.
Statistical support will be provided by an ongoing collaboration with the statistical team at the Nuffield Department of Primary Care Health Sciences Clinical trials Unit.

12 DATA MANAGEMENT

12.1 Data Handling

The Chief Investigator will be responsible for all data that accrues from the study.

The data will be captured directly into the volunteers' electronic CRFs or transferred from a paper source in to the eCRF created on the study specific database designed using REDCap™ (an open source clinical trial software for Electronic Data Capture (EDC)). Electronic data will be stored on secure servers, which are outsourced by REDCap™. Data will be entered directly into the study database via a web browser using encrypted (https) transfer. REDCap™ meets FDA part 11B standards. This includes safety data, laboratory data and outcome data.

The participants will be identified by a unique study specific number. The name and any other identifying detail will NOT be included in any study data electronic file with the following exceptions:

- For safety reasons radiological investigations will have volunteer's identifying details as per OUH trust policy.
- In addition, during the inpatient quarantine period, volunteer identifiers will be recorded as part of standard of care as per OUH protocols. e.g. blood tests

Audio recorded files captured during the pre-screening video call will be pseudo-anonymised by saving under a unique study number. Volunteers will not be referred to by their personally identifiable details in

the recorded call. Audio files will be saved on University of Oxford secure servers and stored for those who consent to partake in the study. For individuals, who do not sign an informed consent form these files will be destroyed.

12.2 Record Keeping

The Investigators will maintain appropriate medical and research records for this study, in compliance with GCP and regulatory and institutional requirements for the protection of confidentiality of volunteers. The Chief Investigator, co-Investigators and clinical research nurses will have access to records. Direct access will be granted to authorised representatives from the Sponsor, host institution and the regulatory authorities to permit trial-related monitoring, audits and inspections.

With the volunteers' consent, we will keep their contact details after participation in the study is complete, so we may inform them of opportunities to participate in future vaccine related research. This will be entirely optional and participation in this study will not be affected by their decision to allow or not allow storage of their contact details beyond participation in this study. Details will be stored electronically on a secure server and only authorised individuals at the CCVTM will have access to it. We will not, under any circumstances, share their contact details with any third party institutions without their permission. Volunteers will be informed that being contacted does not oblige them to agree to take part in future research and they can ask us to have their contact details removed from our database at any time.
All original source records will be kept on site for the duration of the study. Following the closure of the study, all records will be transferred to a secure archiving facility for a minimum of 3 years and maximum of 7 years. Subsequently a data base will be maintained with de-identified data for 25 years.

12.3 Source Data and Case Report Forms (CRFs)

All protocol-required information will be collected in CRFs designed by the investigator. All source documents will be filed in the CRF. Source documents are original documents, data, and records from which the volunteer's CRF data are obtained. For this study, these will include, but are not limited to, volunteer consent form, blood and microbiology results, radiology report, GP response letters, laboratory records, diaries and correspondence. In the majority of cases, CRF entries will be considered source data as the CRF is the site of the original recording (i.e. there is no other written or electronic record of data). In this study this will include, but is not limited to medical history, medication records, vital signs, physical examination records, urine assessments, blood and nasal-oropharyngeal swab results, adverse event data and details of SARS-CoV-2 administration. All source data and volunteer CRFs will be stored securely.

On all study-specific documents, other than the contact form, signed consent, radiology reports, SARS-CoV-2 nasal-pharyngeal swabs (if required by UK HSA), GP/medical correspondence, the participant will be referred to by the unique specific study number, not by name.

12.4 Data Protection

The study will comply with the General Data Protection Regulation (GDPR) and Data Protection Act 2018, which require data to be anonymised as soon as it is practical to do so. The processing of the personal data of participants will be minimised by making use of a unique participant study number only on all study documents and any electronic database(s). All documents will be stored securely and only accessible by study staff and authorised personnel. The study staff will safeguard the privacy of participants' personal

data. No information concerning the study or the data will be released to any unauthorised third party, without prior written approval of the Sponsor.

12.5 Data Quality

Data collection tools will undergo appropriate validation to ensure that data are collected accurately and completely. Datasets provided for analysis will be participant to quality control processes to ensure analysed data is a true reflection of the source data.
Study data will be managed in compliance with local data management SOPs (including the overarching SOP OVC007 Data and Database Management).

13 QUALITY CONTROL AND QUALITY ASSURANCE PROCEDURES

13.1 Investigator procedures

Approved site-specific standard operating procedures (SOPs) will be used at all clinical and laboratory sites.

13.2 Monitoring

Regular monitoring will be performed by The University of Oxford Clinical Trials & Research Governance team according to the trial specific Monitoring Plan. Data will be evaluated for compliance with the protocol and accuracy in relation to source documents as these are defined in the trial specific Monitoring Plan. Following written standard operating procedures, the monitors will verify that the clinical trial is conducted and data are generated, documented and reported in compliance with the protocol, GCP and the applicable regulatory requirements.

13.3 Protocol deviation

Any deviations from the protocol will be documented in a protocol deviation form and filed in the trial master file. Each deviation will be assessed as to its impact on volunteer safety and study conduct. Significant protocol deviations will be listed in the end of study report.

13.4 Audit & inspection

The QA manager conducts systems based internal audits to check that trials are being conducted according to local procedures and in compliance with GCP and applicable regulations.

The Sponsor, trial sites, and ethical committee(s) may carry out audit to ensure compliance with the protocol, GCP and appropriate regulations.

14 SERIOUS BREACHES

A serious breach is defined as “A breach of GCP or the study protocol which is likely to affect to a significant degree:

- the safety or physical or mental integrity of the participants of the study
- the scientific value of the study”

In the event that a serious breach is suspected, the Sponsor will contact the REC within 7 days of becoming aware of the breach of GCP.

15 ETHICS AND REGULATORY CONSIDERATIONS

15.1 Declaration of Helsinki

The Investigators will ensure that this study is conducted according to the principles of the current revision of the Declaration of Helsinki.

15.2 Guidelines for Good Clinical Practice

The Investigator will ensure that this study is conducted in accordance with relevant regulations and with Good Clinical Practice.

15.3 Ethical Approvals

The protocol, informed consent form, participant information sheet and any proposed advertising material will be submitted to an appropriate Research Ethics Committee (REC), HRA and host institution(s) for written approval.

No substantial amendments to this protocol will be made without consultation with, and agreement of, the sponsor. Any substantial amendments that appear necessary during the course of the study must be discussed by the investigator and sponsor concurrently. If agreement is reached concerning the need for an amendment, it will be produced in writing by the chief investigator (or delegate) and will be made a formal part of the protocol following ethical approval.

The Investigator is responsible for ensuring that changes to an approved study, during the period for which ethical committee(s)' approval has already been given, are not initiated without ethical committee(s)' review and approval except to eliminate apparent immediate hazards to the participant (i.e as an Urgent Safety Measure).

The Investigators will notify deviations from the protocol or SAEs occurring at the site to the Sponsor and will notify the REC of these in accordance with local procedures.

15.4 Volunteer Confidentiality

All data will be pseudonymised whereby volunteer data will be identified by a unique study number in the CRF and database. A separate confidential file containing identifiable information will be stored in a secured location at the local trial site only in accordance with the Data Protection Act 2018. Only the sponsor representative, investigators, the clinical monitor and the REC will have access to all the records. In addition, we will be sharing pseudoanonymised data with Regeneron and the MHRA on SAEs deemed related to REGN COV2. This may include pseudoanonymised information from the volunteer's medical notes. Optional written informed consent will be sought from the volunteer to take photographs if required to document an AE as per section 10, or for other purposes such as media communication. These photographs will not include the volunteer's face and will be identified by the date, trial code and participant's unique identifier. Once developed, photographs will be stored pseudonymised, as above. This material may be shown to other professional staff, used for educational purposes, media

communication or included in a scientific publication. Photos may be taken which include identifiable features e.g. for media communication, but if so, a separate written consent for this will be sought from the volunteer.

16 FINANCING AND INSURANCE

16.1 Financing

This study will be funded by the Wellcome Trust and the Department of Health and Social Care (DHSC). The follow-up CMR scans will be fund by Professor Keith Channon via Oxford BRC (OxAMI study).

16.2 Insurance

The University has a specialist negligent harm insurance policy in place which would operate in the event of any participant suffering harm as a result of their involvement in the research (Newline Underwriting Management Ltd, at Lloyd's of London). In addition, Oxford University Hospitals NHS Foundation Trust holds negligent harm insurance policies which apply to this study.

16.3 Contractual Arrangements

Appropriate contractual arrangements will be put in place with all third parties

16.4 Compensation

Volunteers will be compensated for their time, inconvenience and their travel expenses. The maximum total amount compensated will be £4845 for full study participation . Additional compensation will be provided for any additional days in quarantine beyond day 17, COVID positive visits and any unscheduled additional visits.

Should the volunteer decide to withdraw from the study before it is completed, payment will be pro rata.

17 PUBLICATION POLICY

The Investigators will be involved in reviewing drafts of the manuscripts, abstracts, press releases and any other publications arising from the study. Data from the study may also be used as part of a thesis for a PhD or MD. When the study is complete, a manuscript describing the primary study results will be written and published in a peer-reviewed, open access journal. International guidelines will be followed regarding authorship. There may also be secondary publications on more exploratory results. A summary of published reports will be sent to all trial participants for information purposes.

References

1. Van Elslande, J., et al., *Longitudinal follow-up of IgG anti-nucleocapsid antibodies in SARS-CoV-2 infected patients up to eight months after infection.* J Clin Virol, 2021. **136**: p. 104765.
2. *Immune responses and immunity to SARS-CoV-2: European Centre for Disease Prevention and Control*. 2021.

3. Levine, M.M., et al., *Viewpoint of a WHO Advisory Group Tasked to consider establishing a closely-monitored challenge model of coronavirus disease 2019 (COVID-19) in healthy volunteers.* Clinical Infectious Diseases, 2021. **72**(11): p. 2035-2041.
4. Lee, F.E., et al., *Experimental infection of humans with A2 respiratory syncytial virus.* Antiviral Res, 2004. **63**(3): p. 191-6.
5. Memoli, M.J., et al., *Validation of the wild-type influenza A human challenge model H1N1pdMIST: an A(H1N1)pdm09 dose-finding investigational new drug study.* Clin Infect Dis, 2015. **60**(5): p. 693-702.
6. Pickering, S., et al., *Comparative performance of SARS-CoV-2 lateral flow antigen tests and association with detection of infectious virus in clinical specimens: a single-centre laboratory evaluation study.* Lancet Microbe, 2021.
7. Agency, M.H.P.R., *Summary of Product Characteristics for Ronapreve*. 2021, MHRA, GOV.UK: https://www.gov.uk/government/publications/regulatory-approval-of-ronapreve/summary-of-product-characteristics-for-ronapreve.
8. Baum, A., et al., *REGN-COV2 antibodies prevent and treat SARS-CoV-2 infection in rhesus macaques and hamsters.* Science, 2020. **370**(6520): p. 1110-1115.
9. Weinreich, D.M., et al., *REGN-COV2, a Neutralizing Antibody Cocktail, in Outpatients with Covid-19.* N Engl J Med, 2020.
10. Government, U. *Coronavirus (COVID-19) in the UK: https://coronavirus.data.gov.uk/*. [Website] 2022 [accessed 9th November 2022].
11. Wibmer, C.K., et al., *SARS-CoV-2 501Y.V2 escapes neutralization by South African COVID-19 donor plasma.* bioRxiv, 2021: p. 2021.01.18.427166.
12. Hall, V., et al., *Do antibody positive healthcare workers have lower SARS-CoV-2 infection rates than antibody negative healthcare workers? Large multi-centre prospective cohort study (the SIREN study), England: June to November 2020.* medRxiv, 2021: p. 2021.01.13.21249642.
13. Hall V, F.S., , Charlett A, Atti A, Monk EJM, Simmons R, Wellington E, Cole MJ, Saei A, Oguti B, Munro K, Wallace S, Kirwan PD, Shrotri M, Vusirikala A ,Rokadiya S, Kall M, Zambon M, Ramsay M, Brooks T, Brown CS , Chand MA and H.S.S.s. group, *Do antibody positive healthcare workers have lower SARS-CoV-2 infection rates than antibody negative healthcare workers? Large multi-centre prospective cohort study (the SIREN study), England: 4 June to November 2020* MedRxiv (pre-print), 2021.
14. Lumley, S.F., et al., *The duration, dynamics and determinants of SARS-CoV-2 antibody responses in individual healthcare workers.* Clin Infect Dis, 2021.
15. Rogers, T.F., et al., *Isolation of potent SARS-CoV-2 neutralizing antibodies and protection from disease in a small animal model.* Science, 2020. **369**(6506): p. 956-963.
16. Brouwer, P.J.M., et al., *Potent neutralizing antibodies from COVID-19 patients define multiple targets of vulnerability.* Science, 2020. **369**(6504): p. 643-650.
17. Quinti, I., et al., *A possible role for B cells in COVID-19? Lesson from patients with agammaglobulinemia.* J Allergy Clin Immunol, 2020. **146**(1): p. 211-213.e4.
18. Sahin, U., et al., *BNT162b2 induces SARS-CoV-2-neutralising antibodies and T cells in humans.* medRxiv, 2020: p. 2020.12.09.20245175.
19. Ebinger, J.E., et al., *Prior COVID-19 Infection and Antibody Response to Single Versus Double Dose mRNA SARS-CoV-2 Vaccination.* medRxiv : the preprint server for health sciences, 2021: p. 2021.02.23.21252230.
20. Planas, D., et al., *Reduced sensitivity of SARS-CoV-2 variant Delta to antibody neutralization.* Nature, 2021. **596**(7871): p. 276-280.
21. Wang, Z., et al., *Naturally enhanced neutralizing breadth against SARS-CoV-2 one year after infection.* Nature, 2021. **595**(7867): p. 426-431.
22. Zollner, A., et al., *B and T cell response to SARS-CoV-2 vaccination in health care professionals with and without previous COVID-19.* EBioMedicine, 2021. **70**: p. 103539-103539.

23. Nadesalingam, A., et al., *Paucity and discordance of neutralising antibody responses to SARS-CoV-2 VOCs in vaccinated immunodeficient patients and health-care workers in the UK.* The Lancet Microbe, 2021. **2**(9): p. e416-e418.
24. Sterlin, D., et al., *IgA dominates the early neutralizing antibody response to SARS-CoV-2.* Science translational medicine, 2021. **13**(577): p. eabd2223.
25. Roberts, A., et al., *Animal models and vaccines for SARS-CoV infection.* Virus Res, 2008. **133**(1): p. 20-32.
26. Watson, J.M., et al., *Characterisation of a wild-type influenza (A/H1N1) virus strain as an experimental challenge agent in humans.* Virology journal, 2015. **12**: p. 13-13.
27. Wölfel, R., et al., *Virological assessment of hospitalized patients with COVID-2019.* Nature, 2020. **581**(7809): p. 465-469.
28. van Kampen, J.J.A., et al., *Shedding of infectious virus in hospitalized patients with coronavirus disease-2019 (COVID-19): duration and key determinants.* medRxiv, 2020: p. 2020.06.08.20125310.
29. Consortium, C.U.G., *COG-UK report on SARS-CoV-2 Spike mutations of interest in the UK 15th January 2021*. 2021: https://www.cogconsortium.uk/wp-content/uploads/2021/01/Report-2_COG-UK_SARS-CoV-2-Mutations.pdf.
30. PHE, *Technical briefing 5: Investigation of novel SARS-CoV-2 variant; Variant of Concern 202012/01*. 2020: https://assets.publishing.service.gov.uk/government/uploads/system/uploads/attachment_data/file/957504/Variant_of_Concern_VOC_202012_01_Technical_Briefing_5_England.pdf.
31. Novovax, I., *Novavax COVID-19 Vaccine Demonstrates 89.3% Efficacy in UK Phase 3 Trial*. 2021: https://ir.novavax.com/news-releases/news-release-details/novavax-covid-19-vaccine-demonstrates-893-efficacy-uk-phase-3.
32. Moderna, I., *Moderna COVID-19 Vaccine Retains Neutralizing Activity Against Emerging Variants First Identified in the U.K. and the Republic of South Africa*. https://investors.modernatx.com/news-releases/news-release-details/moderna-covid-19-vaccine-retains-neutralizing-activity-against.
33. Xie, X., et al., *Neutralization of SARS-CoV-2 spike 69/70 deletion, E484K, and N501Y variants by BNT162b2 vaccine-elicited sera.* bioRxiv, 2021: p. 2021.01.27.427998.
34. Johnson, J., *Johnson & Johnson Announces Single-Shot Janssen COVID-19 Vaccine Candidate Met Primary Endpoints in Interim Analysis of its Phase 3 ENSEMBLE Trial*. 2021, Johnson & Johnson: https://www.janssen.com/uk/sites/www_janssen_com_uk/files/final_covid-19_ph3toplinedata_final.pdf.
35. Voysey, M.e.a., *Single Dose Administration, And The Influence Of The Timing Of The Booster Dose On Immunogenicity and Efficacy Of ChAdOx1 nCoV-19 (AZD1222) Vaccine.* Lancet Pre-prints; https://papers.ssrn.com/sol3/papers.cfm?abstract_id=3777268, 2021.
36. Times, F. *Oxford/AstraZeneca jab fails to prevent mild and moderate Covid from S African strain, study shows*. 2021 [cited 2021 11/02/2021].
37. Cevik, M., et al., *SARS-CoV-2, SARS-CoV, and MERS-CoV viral load dynamics, duration of viral shedding, and infectiousness: a systematic review and meta-analysis.* The Lancet Microbe, 2021. **2**(1): p. e13-e22.
38. Tabatabaeizadeh, S.A., *Airborne transmission of COVID-19 and the role of face mask to prevent it: a systematic review and meta-analysis.* Eur J Med Res, 2021. **26**(1): p. 1.
39. van Doremalen, N., et al., *Aerosol and Surface Stability of SARS-CoV-2 as Compared with SARS-CoV-1.* N Engl J Med, 2020. **382**(16): p. 1564-1567.
40. Kasloff, S.B., et al., *Stability of SARS-CoV-2 on critical personal protective equipment.* Sci Rep, 2021. **11**(1): p. 984.
41. Abu-Raddad, L.J., et al., *Assessment of the risk of SARS-CoV-2 reinfection in an intense re-exposure setting.* medRxiv, 2020: p. 2020.08.24.20179457.

42. Lumley, S.F., et al., *Antibodies to SARS-CoV-2 are associated with protection against reinfection.* medRxiv, 2020: p. 2020.11.18.20234369.
43. Deng, W., et al., *Primary exposure to SARS-CoV-2 protects against reinfection in rhesus macaques.* Science, 2020. **369**(6505): p. 818-823.
44. Iwasaki, A. and Y. Yang, *The potential danger of suboptimal antibody responses in COVID-19.* Nat Rev Immunol, 2020. **20**(6): p. 339-341.
45. E.C.D.C. *European Centre for Disease Prevention and Control. Reinfection with SARS-CoV: considerations for public health response*. 2020; Available from: https://www.ecdc.europa.eu/en/publications-data/threat-assessment-brief-reinfection-sars-cov-2.
46. Tillett, R.L., et al., *Genomic evidence for reinfection with SARS-CoV-2: a case study.* Lancet Infect Dis, 2021. **21**(1): p. 52-58.
47. Spiegelhalter, D., *Use of "normal" risk to improve understanding of dangers of covid-19.* BMJ, 2020. **370**: p. m3259.
48. Salje, H., et al., *Estimating the burden of SARS-CoV-2 in France.* Science, 2020. **369**(6500): p. 208-211.
49. Sakurai, A., et al., *Natural History of Asymptomatic SARS-CoV-2 Infection.* The New England journal of medicine, 2020. **383**(9): p. 885-886.
50. Kuiper, V.P., et al., *Assessment of risks associated with SARS-CoV-2 experimental human infection studies.* Clinical Infectious Diseases, 2020.
51. England, N. *COVID 19 NHS statistics*. 2021.
52. Williamson, B.N., et al., *Clinical benefit of remdesivir in rhesus macaques infected with SARS-CoV-2.* bioRxiv, 2020: p. 2020.04.15.043166.
53. Harrison, E.M.e.a., *Ethnicity and Outcomes from COVID-19: The ISARIC CCP-UK Prospective Observational Cohort Study of Hospitalised Patients.* The Lancet, 2020.
54. Clift, A.K., et al., *Living risk prediction algorithm (QCOVID) for risk of hospital admission and mortality from coronavirus 19 in adults: national derivation and validation cohort study.* Bmj, 2020. **371**: p. m3731.
55. Polack, F.P., et al., *Safety and Efficacy of the BNT162b2 mRNA Covid-19 Vaccine.* New England Journal of Medicine, 2020.
56. Statistics, O.f.N. *Updating ethnic contrasts in deaths involving the coronavirus (COVID-19), England and Wales: deaths occurring 2 March to 28 July 2020*. 2020 [cited 2020.
57. He, X., et al., *Temporal dynamics in viral shedding and transmissibility of COVID-19.* Nature Medicine, 2020. **26**(5): p. 672-675.
58. Tenforde, M.W., *Symptom Duration and Risk Factors for Delayed Return to Usual Health Among Outpatients with COVID-19 in a Multistate Health Care Systems Network — United States, March–June 2020.* MMWR Morb. Mortal. Wkly. Rep, 2020. **69**.
59. Berlin, D.A., R.M. Gulick, and F.J. Martinez, *Severe Covid-19.* N Engl J Med, 2020.
60. Docherty, A.B., et al., *Features of 20 133 UK patients in hospital with covid-19 using the ISARIC WHO Clinical Characterisation Protocol: prospective observational cohort study.* BMJ, 2020. **369**: p. m1985.
61. Long, Q.-X., et al., *Clinical and immunological assessment of asymptomatic SARS-CoV-2 infections.* Nature Medicine, 2020. **26**(8): p. 1200-1204.
62. Whittaker, E., et al., *Clinical Characteristics of 58 Children With a Pediatric Inflammatory Multisystem Syndrome Temporally Associated With SARS-CoV-2.* Jama, 2020. **324**(3): p. 259-269.
63. Jiang, L., et al., *COVID-19 and multisystem inflammatory syndrome in children and adolescents.* Lancet Infect Dis, 2020. **20**(11): p. e276-e288.
64. Horby, P., et al., *Dexamethasone in Hospitalized Patients with Covid-19 - Preliminary Report.* N Engl J Med, 2020.

65. Beigel, J.H., et al., *Remdesivir for the Treatment of Covid-19 - Final Report.* The New England journal of medicine, 2020: p. NEJMoa2007764.
66. WHO_Solidarity_Trial_Consortium, *Repurposed Antiviral Drugs for Covid-19 — Interim WHO Solidarity Trial Results (Dec).* New England Journal of Medicine, 2020.
67. MHRA, *Interim Clinical Commissioning Policy: Tocilizumab or Sarilumab for critically ill patients with COVID-19 pneumonia (adults)*. 2021: https://www.cas.mhra.gov.uk/ViewandAcknowledgment/ViewAlert.aspx?AlertID=103138.
68. Hansen, J., et al., *Studies in humanized mice and convalescent humans yield a SARS-CoV-2 antibody cocktail.* Science, 2020. **369**(6506): p. 1010-1014.
69. Baum, A., et al., *Antibody cocktail to SARS-CoV-2 spike protein prevents rapid mutational escape seen with individual antibodies.* Science, 2020. **369**(6506): p. 1014-1018.
70. WHO., *Human challenge trials for vaccine development: regulatory considerations*. 2016.
71. Owen, D.R., et al., *An oral SARS-CoV-2 M(pro) inhibitor clinical candidate for the treatment of COVID-19.* Science, 2021. **374**(6575): p. 1586-1593.
72. Sevrioukova, I.F. and T.L. Poulos, *Structure and mechanism of the complex between cytochrome P4503A4 and ritonavir.* Proc Natl Acad Sci U S A, 2010. **107**(43): p. 18422-7.
73. Hammond, J., et al., *Oral Nirmatrelvir for High-Risk, Nonhospitalized Adults with Covid-19.* N Engl J Med, 2022. **386**(15): p. 1397-1408.
74. *Casirivimab and imdevimab in patients admitted to hospital with COVID-19 (RECOVERY): a randomised, controlled, open-label, platform trial.* Lancet, 2022. **399**(10325): p. 665-676.
75. Weinreich, D.M., et al., *REGEN-COV Antibody Combination and Outcomes in Outpatients with Covid-19.* N Engl J Med, 2021. **385**(23): p. e81.
76. *Pfizer Announces Additional Phase 2/3 Study Results Confirming Robust Efficacy of Novel COVID-19 Oral Antiviral Treatment Candidate in Reducing Risk of Hospitalization or Death*. [cited 2022 09/05/2022].
77. Ngai, J.C., et al., *The long-term impact of severe acute respiratory syndrome on pulmonary function, exercise capacity and health status.* Respirology, 2010. **15**(3): p. 543-50.
78. Lam, M.H., et al., *Mental morbidities and chronic fatigue in severe acute respiratory syndrome survivors: long-term follow-up.* Arch Intern Med, 2009. **169**(22): p. 2142-7.
79. Maxwell, *Living with Covid19.* 2020.
80. Nabavi, N., *Long covid: How to define it and how to manage it.* BMJ, 2020. **370**: p. m3489.
81. Sudre, C.H., et al., *Attributes and predictors of Long-COVID: analysis of COVID cases and their symptoms collected by the Covid Symptoms Study App.* medRxiv, 2020: p. 2020.10.19.20214494.
82. Antonelli, M., et al., *Risk factors and disease profile of post-vaccination SARS-CoV-2 infection in UK users of the COVID Symptom Study app: a prospective, community-based, nested, case-control study.* Lancet Infect Dis, 2021.
83. Gibani, M.M., et al., *Homologous and heterologous re-challenge with Salmonella Typhi and Salmonella Paratyphi A in a randomised controlled human infection model.* PLOS Neglected Tropical Diseases, 2020. **14**(10): p. e0008783.
84. Neuzil, K.M., et al., *Early Insights From Clinical Trials of Typhoid Conjugate Vaccine.* Clin Infect Dis, 2020. **71**(Supplement_2): p. S155-s159.
85. Guvenel, A., et al., *Epitope-specific airway-resident CD4+ T cell dynamics during experimental human RSV infection.* J Clin Invest, 2020. **130**(1): p. 523-538.
86. Jozwik, A., et al., *RSV-specific airway resident memory CD8+ T cells and differential disease severity after experimental human infection.* Nat Commun, 2015. **6**: p. 10224.
87. Habibi, M.S., et al., *Neutrophilic inflammation in the respiratory mucosa predisposes to RSV infection.* Science, 2020. **370**(6513).
88. de Graaf, H., et al., *Controlled Human Infection With Bordetella pertussis Induces Asymptomatic, Immunizing Colonization.* Clin Infect Dis, 2020. **71**(2): p. 403-411.

89. Williams, C.M., et al., *Exhaled Mycobacterium tuberculosis output and detection of subclinical disease by face-mask sampling: prospective observational studies.* Lancet Infect Dis, 2020. **20**(5): p. 607-617.

90. Williams, C.M., et al., *COVID-19: Exhaled virus detected by Face Mask Sampling provides new links to disease severity and potential infectivity.* medRxiv, 2020: p. 2020.08.18.20176693.

91. England, P.H., *Green Book Chapter 14a=COVID-19-SARS-CoV-2*. 2021.

92. Inc, R.P., *Investigator's Brochure REGN10933+REGN10987(REGN-COV2)*. November 2020.

93. CPOC, *FAQs for patients having an operation during the COVID-19 (coronavirus) pandemic by the Centre for Perioperative Care*, C.f.P. Care, Editor. 2020: https://cpoc.org.uk/sites/cpoc/files/documents/2020-11/UPDATED%20NOV%202020-%20CPOC-FAQ%20For%20patients%20having%20an%20operation%20during%20the%20pandemic.pdf.
